# Synthesis, Antimicrobial Activities, and Model of Action of Novel Tetralone Derivatives Containing Aminoguanidinium Moiety

**DOI:** 10.3390/ijms26135980

**Published:** 2025-06-21

**Authors:** Qing-Jie Zhang, Yu-Xi Li, Wen-Bo Ge, Li-Xia Bai, Xiao Xu, Ya-Jun Yang, Xi-Wang Liu, Jian-Yong Li

**Affiliations:** Key Lab of New Animal Drug of Gansu Province, Key Lab of Veterinary Pharmaceutical Development of Ministry of Agriculture and Rural Affairs, Lanzhou Institute of Husbandry and Pharmaceutical Sciences of Chinese Academy of Agricultural Sciences, Lanzhou 730050, China

**Keywords:** aminoguanidine, tetralone, synthesis, antibacterial activity, ESKAPE pathogens, *S. aureus*, MRSA, structure–activity relationship, dihydrofolate reductase, membrane disruption, in vivo antibacterial efficacy, drug development

## Abstract

The objectives of this study were to design, synthesize, and evaluate the antibacterial activity of a series of novel aminoguanidine-tetralone derivatives. Thirty-four new compounds were effectively synthesized through nucleophilic substitution reaction and guanidinylation reaction. Chemical structures of all the desired compounds were identified by NMR and HR-MS spectroscopy. Most of the synthesized compounds showed significant antibacterial activity against ESKAPE pathogens and clinically resistant *Staphylococcus aureus* (*S. aureus*) isolates. *S. aureus* is an important pathogen that has the capacity to cause a variety of diseases, including skin infections, pneumonia, and sepsis. The most active compound, **2D**, showed rapid bactericidal activity against *S. aureus* ATCC 29213 and MRSA-2 with MIC/MBC values of 0.5/4 µg/mL and 1/4 µg/mL, respectively. The hemolytic activity and cytotoxicity of **2D** was low, with HC_50_ and IC_50(HEK 293-T)_ values of 50.65 µg/mL and 13.09 µg/mL, respectively. Compound **2D** induced the depolarization of the bacterial membrane and disrupted bacterial membrane integrity, ultimately leading to death. Molecular docking revealed that dihydrofolate reductase (DHFR) may be a potential target for **2D**. In the mouse skin abscess model caused by MRSA-2, **2D** reduced the abscess volume, decreased bacterial load, and alleviated tissue pathological damage at doses of 5 and 10 mg/kg. Therefore, compound **2D** may be a promising drug candidate for antibacterial purposes against *S. aureus*.

## 1. Introduction

The emergence and dissemination of antimicrobial resistance represents a significant global public health concern [1]. The resistance of the six pathogens (*Enterococcus faecium*, *Staphylococcus aureus*, *Klebsiella pneumoniae*, *Acinetobacter baumannii*, *Pseudomonas aeruginosa*, and *Escherichia coli*) known as ESKAPE pathogens has been particularly serious [2,3,4]. *Staphylococcus aureus* (*S. aureus*) is a widely distributed opportunistic pathogen that has the capacity to cause a variety of diseases, including skin infections, pneumonia, and sepsis [5]. As a significant clinical pathogen, drug-resistant *Staphylococcus aureus* (*S. aureus*) has attracted widespread attention [1,2,3,4,5]. In particular, methicillin-resistant *S. aureus* (MRSA) has attracted much attention since it was first identification in 1961 [6]. MRSA can be resistant to multiple antibiotics [7], and its recurrent bloodstream infections are associated with high mortality rates [8]. In addition to the rational use of existing antimicrobial agents, novel antimicrobial agents should be actively developed to combat *S. aureus* resistance.

Tetralone and tetralone derivatives, as crucial structural scaffolds of potential novel drugs, are normally found in several natural compounds and can be used as parental scaffolds and/or intermediates for the synthesis of a series of pharmacologically active compounds with a broad spectrum of bioactivities including antibacterial, antitumor, CNS effect, and so on [9]. Tetralone derivatives have attracted wide interest due to their antimicrobial properties. Ampicillin-tetralone derivatives have been shown to overcome drug resistance and demonstrate effective activity against *S. aureus* [10]. The natural product 4-hydroxy-α-tetralone and its derivatives can reverse the resistance of multi-drug resistant *Escherichia coli* by inhibiting the ATP-dependent efflux pump [11]. Furthermore, tetralone derivatives have shown antiviral activity [12,13] and antifungal activity [14]. Since tetralone derivatives exhibit excellent antibacterial potential [15], the molecules containing this scaffold are likely to be promising antibacterial drugs in the future.

Antimicrobials have been shown to cross the membrane, subsequently interacting with the target of action [16], a process which poses a significant challenge to many compounds [17]. Study has also shown that amphiphilic and rigid small molecules containing amines with low sphericity were most likely to accumulate to effect concentrations [18]. Aminoguanidine, a group with strongly alkaline and multiple nitrogen atoms, has attracted significant attention in the field of antimicrobial agents [19,20,21,22]. Quinolone-tethered aminoguanidine molecules have demonstrated noteworthy anti-tuberculosis activity [23]. Aminoguanidine derivatives with 1,2,4-triazol moieties exhibited substantial antibacterial activity against both bacteria and Candida albicans [24]. Diphenylurea derivatives bearing aminoguanidine moiety exhibited potent anti-staphylococcal activity [25]. Consequently, aminoguanidine has emerged as a promising antibacterial pharmacophore.

In this study, a series of tetralone indole derivatives (**1A**–**1X**, **2A**–**2J**) were first designed and synthesized, including 6-hydroxy-1-tetralone with various substituted benzyl bromides or bromoalkane and aminoguanidine to produce the target compound. Then, their structures were characterized. Antibacterial activity against both Gram-positive and Gram-negative bacteria was assessed. The role of the active molecule **2D** in disrupting cell membrane integrity was investigated. A molecular docking study was performed with **2D** and *S. aureus* dihydrofolate reductase (DHFR) to find the potential antibacterial target and elucidate the binding mechanism. Furthermore, the hemolytic activity and cytotoxicity of **2D** were also examined. This study provides a class of compounds for the development of novel antibacterial agents.

## 2. Results and Discussion

### 2.1. Chemistry

**Scheme 1:**
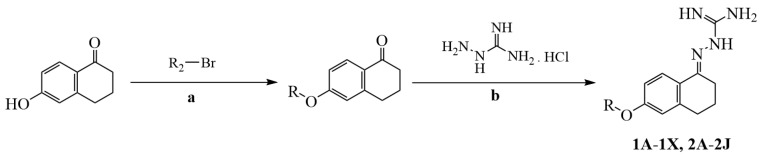
The synthesis routes of compounds **1A**–**1X** and **2A**–**2J**. (**a**) Dry acetonitrile, K_2_CO_3_, KI, 80 °C, 0.5–12 h; (**b**) ethanol absolute, 15 drops of concentrated HCl, 80 °C, 0.5–1 h.

The synthetic route for the preparation of the aminoguanidine tetralone derivatives (**1A**–**1X**, **2A**–**2J**) and intermediates is shown in Scheme 1. The key intermediates were prepared from 6-hydroxy-1-tetralone and substituted benzyl bromides or brominated alkanes or brominated alkenes under alkaline conditions. With the key intermediate in hand, all the target derivatives (Table 1) were prepared directly in the presence of aminoguanidine hydrochloride and concentrated hydrochloric acid by stirring in an oil bath at 80 °C. Finally, the structures of the desired compounds were characterized by ^1^H NMR, ^13^C NMR and high-resolution mass spectrometry (HR-MS) (Supplementary Materials).

### 2.2. Evaluation of In Vitro Antimicrobial Activity

The minimum inhibitory concentrations (MICs) and minimum bactericidal concentrations (MBCs) of the synthesized compounds effective against ESKAPE strains, *S. aureus* clinical isolates, and MRSA were investigated by the broth dilution method. Levofloxacin is a broad-spectrum antibacterial agent that is widely used in the treatment of ESKAPE infections [26]. Therefore, levofloxacin was selected as the positive control for the preliminary activity screening. DMSO (0.1%) was selected as the negative control.

Initial screening results are presented in Table 2. In general, most of the compounds showed strong activity against ESKAPE strains, with MICs ranging from 0.5 μg/mL to 32 μg/mL. Most of the compounds showed higher antibacterial activity against Gram-positive bacteria, with MICs in the ranging from 0.5 μg/mL to 4 μg/mL. For Gram-negative bacteria, the compounds showed better activity against *Escherichia coli* and *Acinetobacter baumannii* in comparison to *Klebsiella pneumoniae* and *Pseudomonas aeruginosa*. Compound **2D** exhibited the highest level of activity against *S. aureus*. Compared with the aminoguanidine derivatives synthesized by Ping Yang et al. (MIC*_S._* _*aureus*_ = 2–128 μg/mL) [27], Wolfgang Dohle et al. (MIC*_S._* _*aureus*_ = 1–32 μg/mL) [28] and Xueqian Bai et al. (MIC*_S._* _*aureus*_ = 4–64 μg/mL) [24], respectively, compound **2D** (MIC*_S._* _*aureus*_ = 0.5 μg/mL) showed superior activity against *S. aureus*.

An analysis of the structure–activity relationship reveals the following: In the case of R being a substituted benzyl group, the antibacterial activity of compounds containing one or two halogens (fluorine, chlorine, bromine) or one trifluoromethyl group was similar. In contrast, the activity of compounds containing two trifluoromethyl groups against Gram-positive bacteria decreased by 2–16 times, and the activity against Gram-negative bacteria was lost. In the case of the R being an alkyl group, compounds with more than nine carbon atoms exhibited a loss of all activity against Gram-negative bacteria, while displaying minimal effect on the activity of Gram-positive bacteria. Among all compounds, those comprising medium-chain alkane groups exhibit better activity in comparison to those with halogenated benzyl groups, as well as short- and long-chain alkane compounds. It is speculated that medium-chain compounds possess a more optimal configuration and lipid–water partition coefficient (LogP).

In view of the excellent anti-*Staphylococcus aureus* activity of the compounds, we further tested the activity of the compounds against four *Staphylococcus aureus* clinical isolates and two MRSA strains (Table 3). Vancomycin is currently the first-line drug against MRSA infections [29], so it was selected as a positive control. The results showed that the synthesized compounds also showed significant activity against the clinical isolates and the MRSA, with MICs ranging from 1 μg/mL to 32 μg/mL. The activity of **1L**, **1M**, **1S**, **1U**, **1X**, **2A**, **2D**–**2F,** and **2I** against MRSA was analogous to that of vancomycin, with MICs ranging from 1 µg/mL to 2 µg/mL. Furthermore, the MBC values of the compounds were detected. The MBC values of most compounds were 1–4 times higher than the MIC values, indicating bactericidal properties of those compounds.

**Figure 1:**
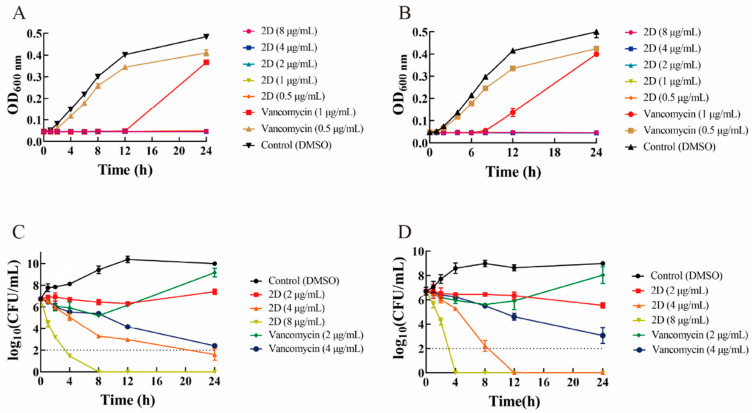
The result of the growth curve and bactericidal time-kill kinetics assay. (**A**) Growth curve of ATCC 29213 in the presence of **2D** (0.5, 1, 2, 4 and 8 µg/mL) and vancomycin (0.5 and 1 µg/mL). (**B**) Growth curve of MRSA-2 in the presence of **2D** (0.5, 1, 2, 4 and 8 µg/mL) and vancomycin (0.5 and 1 µg/mL). (**C**) Time-kill curve of ATCC 29213 in the presence of **2D** (2, 4 and 8 µg/mL) and vancomycin (2 and 4 µg/mL). (**D**) Time-kill curve of MRSA-2 in the presence of **2D** (2, 4 and 8 µg/mL) and vancomycin (2 and 4 µg/mL).

As the most active compound, the bacteriostatic and time-killing ability of **2D** was investigated in vitro. **2D** was able to suppress the growth of ATCC 29213 and MRSA-2 at concentrations 0.5, 1, 2, 4, and 8 µg/mL. Vancomycin at 0.5 μg/mL and 1 μg/mL failed to completely inhibit the growth of ATCC 29213 and MRSA-2 (Figure 1A,B). The time-kill assay showed that **2D** exhibited a concentration-dependent and time-dependent bactericidal response (Figure 1C,D).

### 2.3. **2D** Exhibits Potent Anti-Biofilm Properties

**Figure 2:**
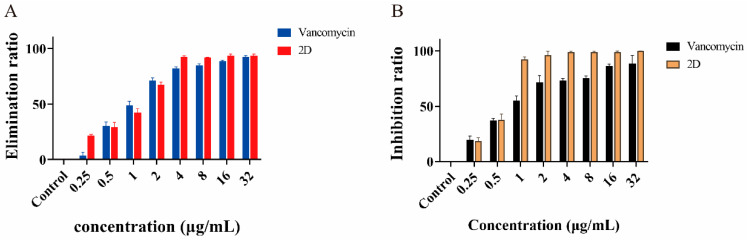
Elimination and inhibition of biofilm. (**A**) Elimination rates of biofilm by different concentrations of **2D** and vancomycin against MRSA-2. (**B**) Inhibition rates of biofilm by different concentrations of **2D** and vancomycin against MRSA-2.

The biofilm is a highly structured microbial community that protects bacterial cells from the external environment and resists antibiotics [30]. Therefore, good anti-biofilm activity is an important ability of new antimicrobial agents [31]. The results showed that the biofilm elimination rate of **2D** (0.25 μg/mL) was 21.4%, which was significantly superior to that of vancomycin at 0.25 μg/mL (3.63%) (Figure 2A). In the concentration range of 4–32 μg/mL, **2D** exhibited a biofilm elimination rate of 91.8% to 93.7%, which was more effective than vancomycin. However, at the test concentration, vancomycin was unable to completely eliminate the biofilm, a finding that is consistent with the existing research [32].

The ability of **2D** and vancomycin to inhibit biofilm formation at 0.25 μg/mL and 0.5 μg/mL (1/4MIC and 1/2MIC) was similar, with an inhibition rate of 18.5% and 37.8% (Figure 2B). **2D** at 1 μg/mL (1MIC) reduced biofilm formation by more than 92%. Furthermore, the **2D** concentrations of 2–32 μg/mL (2-32MIC) almost completely inhibited the formation of biofilm.

### 2.4. **2D** Destroys the Bacterial Structure

**Figure 3:**
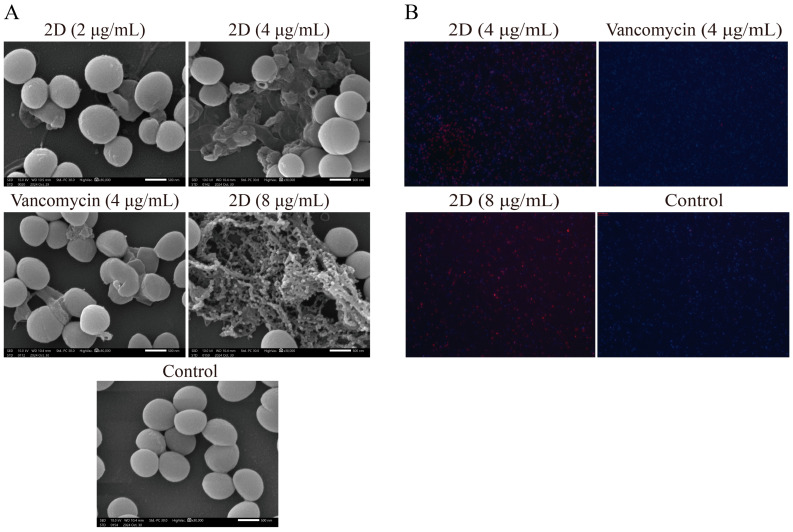
(**A**) The results of scanning electron microscopy assay. The strain used was MRSA-2. Scale bar, 500 nm. (**B**) The results of fluorescence microscopy assay. The strain used was MRSA-2.

The primary antibacterial mechanism of guanidine involves the destruction of bacterial membranes [33]. Therefore, scanning electron microscopy (SEM) was used to observe the effect of **2D** on MRSA cell membranes. As shown in Figure 3, the cell membrane of MRSA-2 was intact in the control group. Following treatment with vancomycin (4 μg/mL) and **2D** (2 μg/mL), a small number of bacteria exhibited rupture folds. However, after treatment with **2D** at concentrations of 4 and 8 μg/mL, a substantial number of bacteria membranes were broken. These results suggest that **2D** can destroy the membrane structure of MRSA-2, and this effect is concentration-dependent.

### 2.5. Fluorescence Microscopy

DAPI has been shown to stain living cells blue. PI has the ability to stain dead cells with damaged cell membranes a red color. The results of the fluorescence microscopy analysis showed that the control group exhibited almost no red fluorescence, with only significant blue fluorescence being observed (Figure 3B). The vancomycin (4 µg/mL) group exhibited a very small amount of red fluorescence. The **2D** (4 and 8 μg/mL) group exhibited a marked tendency to emit red fluorescence. The findings indicated that **2D** exerted a substantial destructive impact on the cell membrane. This finding aligns with the results obtained from the SEM analysis.

### 2.6. Membrane Permeabilization and Depolarization

**Figure 4:**
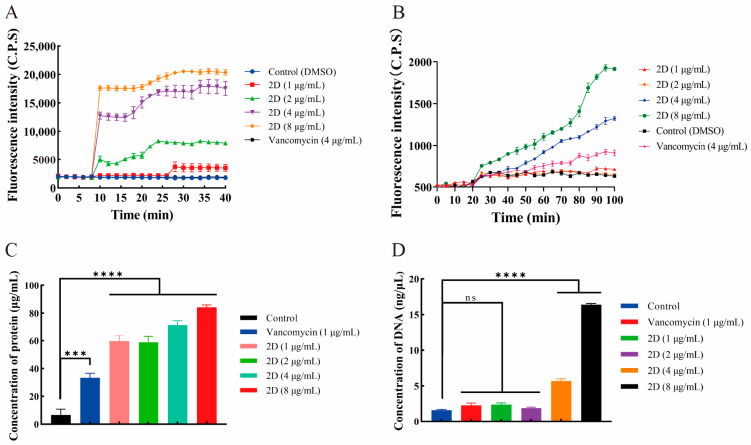
(**A**) Cytoplasmic membrane depolarization of **2D** using DiSC3(5) assays. (**B**) The **2D** increased the permeability of the inner membrane. (**C**) Protein leakage caused by the action of **2D** on MRSA-2. (**D**) DNA leakage resulting from the action of **2D** on MRSA-2. “ns” is not significant; *** *p* < 0.001; **** *p* < 0.0001.

Since compound **2D** significantly destroyed the membrane structure of bacteria from SEM and fluorescence microscopy, the effect of **2D** on bacterial plasma membranes was further studied by fluorescent probes DiSC3(5) and PI. DiSC3(5) localizes in the bacterial membrane depending on an intact membrane potential gradient, where self-quenching occurs [34]. After adding **2D** (2, 4, and 8 μg/mL), the fluorescence intensity increased instantaneously (at 10 min), and the final fluorescence intensity was significantly higher than that of the control group (Figure 4A). This finding indicates that **2D** has the capacity to depolarize the bacterial cell membrane. The PI enters the bacteria only through the damaged membrane and emits strong fluorescence when combined with DNA [35]. After treatment with **2D** (4 and 8 μg/mL), the fluorescence intensity of MRSA-2 was significantly enhanced (Figure 4B). The results showed that **2D** could destroy the integrity of the inner membrane of MRSA-2. Concurrently, the effect of **2D** on MRSA-2 cell membranes exhibited a concentration-dependent response.

### 2.7. Leakage of Protein and DNA

The disruption of the bacterial cell membrane can result in the efflux of intracellular substances [36]. As shown in Figure 4C,D, **2D** significantly increased the content of extracellular protein and DNA compared with the control group. These results indicated that **2D**-induced membrane damage in MRSA-2 can lead to intracellular protein and DNA leakage.

### 2.8. Docking Analysis

**Figure 5:**
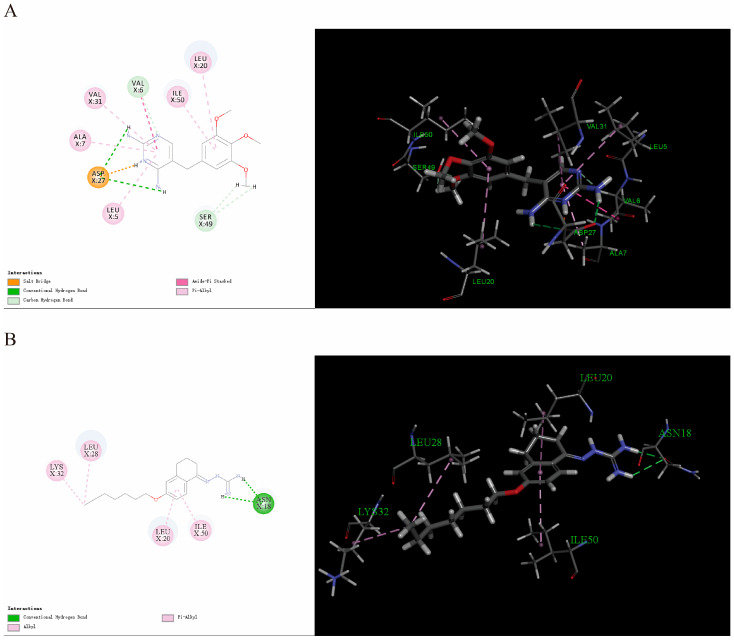
(**A**) Interaction of trimethoprim with the *S. aureus* DHFR protein. (**B**) Interaction of **2D** with the *S. aureus* DHFR protein.

Guanidine has been demonstrated to exhibit a variety of antibacterial mechanisms [37]. As previous studies have shown, aminoguanidine carbazole derivatives [38] and aminoguanidine indole derivatives [39] have strong binding ability with dihydrofolate reductase (DHFR). DHFR is an important antibacterial target [40]. Therefore, the binding capacity of the interaction between **2D** and the *S. aureus* DHFR protein was investigated. The -CDOCKER₋ENERGY of the dihydrofolate reductase inhibitor trimethoprim was determined to be 32.9555. As shown in the docking results, the docked pose of **2D** with DHFR had a -CDOCKER₋ENERGY of 33.7945, suggesting that **2D** can bind to potential binding sites in the DHFR. Five active site residues (ASN18, LEU20, LEU28, LYS32, and ILE50) were involved in **2D** recognition (Figure 5). In DHFR, the ASN18 residue formed a conventional hydrogen bond interaction with the guanidine of the **2D**. The side chains of LEU20, LEU28, LYS32, and ILE50 formed alkyl stacking with **2D**. The tetralin ring and alkyl moieties enhanced the hydrophobic interaction between **2D** and DHFR.

### 2.9. Hemolytic Activity and Cytotoxicity

The hemolysis of compound **2D** was determined by its ability to lyse sheep red blood cells. The results were expressed as the HC_50_ value (the concentration at which 50% of red blood cells were lysed). The HC_50_ value of **2D** was 50.65 μg/mL, indicating that the compound exhibited selective toxicity to bacterial cells (SI = 101.30) (Table 4).

In addition, the cytotoxicity of **2D** in four mammalian cell lines was also detected (Table 4). The selectivity index (SI) of **2D** was determined to be 26.18 for *S. aureus* ATCC 29213 and the normal human cell line HEK 293-T. In accordance with SI > 10 [41], the results showed that **2D** was less toxic to mammalian cells.

### 2.10. In Vivo Efficacy

**Figure 6:**
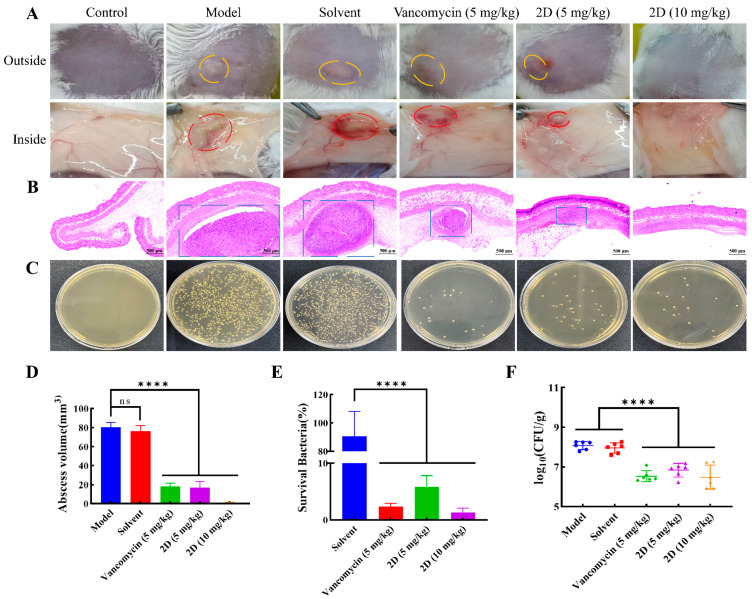
**2D** can treat MRSA infection in mice. (**A**) Photographs of MRSA-2 infection sites with various treatments after infection. Skin abscesses were marked by the orange circles (outside) and red circles (inside). (**B**) Histological photographs of infected skin that received various treatments. Suppurative lesions were marked by the blue sqaures. Scale bar, 500 μm. (**C**) Images of MRSA-2 colonies cultured from the homogenate of infected skin after appropriate dilution. (**D**) Size of abscess at MRSA-2 infection site under different treatments. (**E**) Bacterial survival in the skin of MRSA-2-infected mice of each group treated with **2D** and vancomycin. (**F**) The tissue bacteria load of MRSA-2-infected mice skin in each group treated with **2D** (5 and 10 mg/kg) and vancomycin (5 mg/kg). The data are presented as the means ± SD from six independent experiments; “ns” is not significant; **** *p* < 0.0001.

**Figure 7:**
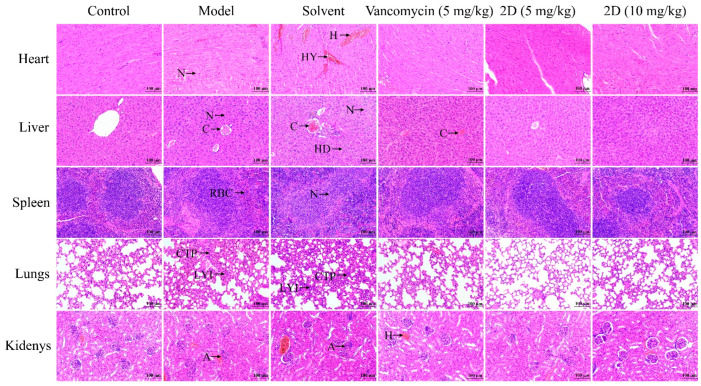
Histopathological analysis of different tissues using hematoxylin-eosin (H&E) staining. A: atrophy; C: congestion; CTP: connective tissue proliferation; H: hemorrhage; HD: hepatocyte degeneration; HY: hyperemia; LYI: lymphocyte infiltration; N: necrosis; RBC: red blood cell. Scale bar, 100 μm.

As **2D** has exhibited potent antibacterial activity in vitro, its antibacterial effect in vivo was assessed by a mouse skin abscess model. After a 48 h period of infection, the dorsal skin of mice in both the model group and the solvent group displayed significant swelling, accompanied by an increase in the volume of subcutaneous abscesses. The abscesses in the vancomycin group and the **2D** (5 mg/kg) group exhibited a reduced volume compared to the model group and the solvent group, although complete resolution was not observed. The dorsal skin of mice treated with **2D** (10 mg/kg) displayed a smooth appearance and was devoid of any discernible abscesses. **2D** (5 and 10 mg/kg) exhibited superiority over vancomycin in terms of reducing the volume of skin abscesses (Figure 6A,D). The results of the histopathological examination of the skin indicated that the mice in the model group developed a suppurative lesion with a distinct boundary with the surrounding tissues, primarily comprising neutrophils and lymphocytes, which was characterized as suppurative dermatitis. A minimal degree of inflammatory cell infiltration was observed beneath the skin of mice treated with **2D** (10 mg/kg), with the presence of almost no purulent foci noted. The purulent lesions at the infection site of mice in the vancomycin (5 mg/kg) and **2D** (5 mg/kg) groups were significantly smaller (Figure 6B). The results of the tissue bacterial load analysis showed that the bacterial load of the skin at the infected site was significantly reduced after vancomycin and **2D** treatment, compared with the model group and the solvent group (Figure 6C,E,F). The results of the histopathological examination of the major organs showed that the major organs of the model group and the solvent group exhibited significant pathological alterations, in comparison with the blank control group. These alterations were characterized by congestion of the central vein of the liver, indistinct boundaries of the marginal area of the spleen, diffuse necrosis of parenchymal cells, glomerular degeneration and atrophy, and local hemorrhage. After the vancomycin and **2D** treatment, no discernible lesions were observed in the major organs (Figure 7).

In general, the aminoguanidine tetralone derivative **2D** exhibited a favorable therapeutic effect in a mouse model of MRSA-infected skin abscesses, suggesting its potential as a treatment for MRSA infection.

## 3. Materials and Methods

### 3.1. Chemistry

#### 3.1.1. General

All reagents were commercially available and were used without further purification. The reaction process was monitored by Thin Layer Chromatography (TLC). The ^1^H and ^13^C NMR spectra were recorded on an Agilent DD2-600MHz NMR spectrometer at room temperature, operating at 600 MHz for ^1^H NMR and 151 MHz for ^13^C NMR by using *DMSO-d6* as solvent. The mass spectra were obtained at Agilent 6530 QTOF.

#### 3.1.2. Synthesis of Intermediates

A quantity of 1 mmol (163 mg) of 6-hydroxy-1-tetralone was dissolved in 5 mL of dry acetonitrile. Subsequently, 4 mmol (554 mg) of anhydrous potassium carbonate and 0.1 mmol (16.6 mg) of potassium iodide were added to the solution. The mixture was stirred in an oil bath at 80 °C for 10 min. Then, 1 mmol of substituted benzyl bromide or bromoalkane or bromoalkene was added, and the reaction was stirred at 80 °C for 0.5–12 h. After the completion of the reaction, 50 mL of water was added, filtered or extracted with dichloromethane (15 mL × 3, the dichloromethane layer was washed twice with saturated salt water, anhydrous sodium sulfate was used to remove water, and dichloromethane was removed by vacuum distillation). The obtained product was used for the next reactions without further treatment after drying.

#### 3.1.3. General Procedure for the Synthesis of Compounds **1A**–**1X** and **2A**–**2J**

The intermediate (1 mmol) was meticulously suspended in 5 mL of anhydrous ethanol, and 15 drops of concentrated hydrochloric acid were added to the suspension. Subsequently, aminoguanidine hydrochloride (1.2 mmol, 132.7 mg) was added to the mixture and stirred at 80 °C for 0.5–1 h. After the completion of the reaction, the solution was cooled to room temperature. In instances where a substantial quantity of solid precipitation was present, the collected solid was subjected to three cycles of washing with anhydrous ethanol, followed by three additional cycles of washing with water. In the absence of solid precipitation, a small amount of ethyl acetate was added to the reaction solution to precipitate a large amount of solid. The precipitated solids were subsequently collected and washed thrice with ethyl acetate and water, respectively. After solids were dry, the target compounds **1A**–**1X** and **2A**–**2J** were obtained.

The LogP of the compounds was predicted by ADMETlab 3.0 (https://admetlab3.scbdd.com./server/evaluationCal (accessed on 25 April 2025)). The spectral data of the novel compounds are listed below.

(*E*)-2-(6-((2-fluorobenzyl)oxy)-3,4-dihydronaphthalen-1(2*H*)-ylidene)hydrazine-1-carboximidamide(**1A**). Yield: 56.0%, 0.177 g; ^1^H NMR (600 MHz, *DMSO-d6*) δ 11.14 (s, 1H), 8.30–8.26 (m, 1H), 7.95–7.67 (br s, 3H), 7.56 (td, *J* = 7.5, 1.4 Hz, 1H), 7.45–7.40 (m, 1H), 7.28–7.21 (m, 2H), 6.90–6.86 (m, 2H), 5.17 (s, 2H), 2.74 (t, *J* = 6.0 Hz, 1H), 2.66 (t, *J* = 6.5 Hz, 2H), 1.84 (p, *J* = 6.3 Hz, 2H); ^13^C NMR (151 MHz, *DMSO-d6*) δ 160.39 (d, *J*_CF_ = 246.3 Hz), 159.19, 155.96, 151.12, 142.11, 130.76 (d, *J*_CF_ = 4.0 Hz), 130.47 (d, *J*_CF_ = 8.2 Hz), 127.39, 124.53 (d, *J*_CF_ = 3.4 Hz), 124.41, 123.60 (d, *J*_CF_ = 14.6 Hz), 115.39 (d, *J*_CF_ = 20.9 Hz), 113.61, 113.25, 63.54 (d, *J*_CF_ = 3.5 Hz), 28.99, 26.43, 21.18; HRMS (ESI+): *m*/*z* calcd for C_18_H_19_FN_4_O [M + H]^+^ 327.1615, found 327.1628.

(*E*)-2-(6-((3-fluorobenzyl)oxy)-3,4-dihydronaphthalen-1(2*H*)-ylidene)hydrazine-1-carboximidamide(**1B**). Yield: 89.3%, 0.263 g; ^1^H NMR (600 MHz, *DMSO-d_6_*) δ 11.14 (s, 1H), 8.28 (d, *J* = 8.7 Hz, 1H), 7.94–7.64 (br s, 3H), 7.44 (td, *J* = 8.0, 6.0 Hz, 1H), 7.30–7.26 (m, 2H), 7.18–7.14 (m, 1H), 6.89–6.85 (m, 2H), 5.17 (s, 2H), 2.73 (t, *J* = 6.0 Hz, 2H), 2.65 (t, *J* = 6.5 Hz, 2H), 1.84 (p, *J* = 6.3 Hz, 2H); ^13^C NMR (151 MHz, *DMSO-d_6_)* δ 162.18 (d, *J*_CF_ = 243.6 Hz), 159.14, 155.97, 151.12, 142.08, 139.84 (d, *J*_CF_ = 7.5 Hz), 130.48 (d, *J*_CF_ = 8.6 Hz), 127.39, 124.39, 123.52 (d, *J*_CF_ = 2.8 Hz), 114.60 (d, *J*_CF_ = 21.1 Hz), 114.22 (d, *J*_CF_ = 21.6 Hz), 113.70, 113.42, 68.33 (d, *J*_CF_ = 2.1 Hz), 28.99, 26.43, 21.18; HRMS (ESI^+^): *m*/*z* calcd for C_18_H_19_FN_4_O [M + H]^+^ 327.1615, found 327.1625.

(*E*)-2-(6-((4-fluorobenzyl)oxy)-3,4-dihydronaphthalen-1(2*H*)-ylidene)hydrazine-1-carboximidamide(**1C**). Yield: 67.5%, 0.207 g; ^1^H NMR (600 MHz, *DMSO-d_6_*) δ 11.14 (s, 1H), 8.29–8.26 (m, 1H), 7.95–7.66 (br s, 3H), 7.52–7.48 (m, 2H), 7.24–7.19 (m, 2H), 6.86 (t, *J* = 7.9 Hz, 2H), 5.12 (s, 2H), 2.73 (t, *J* = 6.0 Hz, 2H), 2.65 (t, *J* = 6.5 Hz, 2H), 1.83 (p, *J* = 6.3 Hz, 2H), ^13^C NMR (151 MHz, *DMSO-d_6_*) δ 161.77 (d, *J*_CF_ = 243.9 Hz), 159.28, 155.97, 151.15, 142.06, 133.09 (d, *J*_CF_ = 2.9 Hz), 129.98 (d, *J*_CF_ = 8.2 Hz, 2C), 127.37, 124.27, 115.25 (d, *J*_CF_ = 21.4 Hz, 2C), 113.72, 113.37, 68.49, 29.00, 26.44, 21.19; HRMS (ESI^+^): *m*/*z* calcd for C_18_H_19_FN_4_O [M + H]^+^ 327.1615, found 327.1624.

(*E*)-2-(6-((2,4-difluorobenzyl)oxy)-3,4-dihydronaphthalen-1(2*H*)-ylidene)hydrazine-1-carboximidamide(**1D**). Yield: 71.4%, 0.231 g; ^1^H NMR (600 MHz, *DMSO-d_6_*) δ 11.14 (s, 1H), 8.30–8.27 (m, 1H), 8.05–7.67 (br s, 3H), 7.63 (td, *J* = 8.6, 6.6 Hz, 1H), 7.30 (ddd, *J* = 10.3, 9.3, 2.6 Hz, 1H), 7.17–7.10 (m, 1H), 6.88 (t, *J* = 7.6 Hz, 2H), 5.14 (s, 2H), 2.74 (t, *J* = 6.0 Hz, 2H), 2.66 (t, *J* = 6.5 Hz, 2H), 1.84 (p, *J* = 6.3 Hz, 2H); ^13^C NMR (151 MHz, *DMSO-d_6_*) δ 162.32 (dd, *J*_CF_ = 254.6, 12.5 Hz), 160.68 (dd, *J*_CF_ = 256.7, 12.4 Hz), 159.12, 155.97, 151.12, 142.13, 132.23 (dd, *J*_CF_ = 9.9, 5.6 Hz), 127.41, 124.47, 120.11 (dd, *J*_CF_ = 14.9, 3.5 Hz), 113.63, 113.27, 111.61 (dd, *J*_CF_ = 21.3, 3.5 Hz), 104.06 (t, *J*_CF_ = 25.7 Hz), 63.11 (d, *J*_CF_ = 3.0 Hz), 28.99, 26.43, 21.18; HRMS (ESI^+^): *m*/*z* calcd for C_18_H_18_F_2_N_4_O [M + H]^+^ 345.1521, found 345.1526.

(*E*)-2-(6-((2,5-difluorobenzyl)oxy)-3,4-dihydronaphthalen-1(2*H*)-ylidene)hydrazine-1-carboximidamide(**1E**). Yield: 80.2%, 0.254 g; ^1^H NMR (600 MHz, *DMSO-d_6_*) δ 11.14 (s, 1H), 8.31-8.27 (m, 1H), 7.97–7.61 (br s, 3H), 7.43 (ddd, *J* = 8.8, 5.6, 3.2 Hz, 1H), 7.32 (td, *J* = 9.2, 4.5 Hz, 1H), 7.27 (td, *J* = 8.8, 8.3, 4.5 Hz, 1H), 6.92–6.88 (m, 2H), 5.16 (s, 2H), 2.74 (t, *J* = 6.0 Hz, 2H), 2.66 (t, *J* = 6.5 Hz, 2H), 1.84 (p, *J* = 6.3 Hz, 2H); ^13^C NMR (151 MHz, *DMSO-d_6_*) δ 158.95, 158.05 (dd, *J*_CF_ = 240.4, 2.1 Hz), 156.35 (dd, *J*_CF_ = 242.3, 2.4 Hz), 155.96, 151.08, 142.14, 127.42, 125.68 (dd, *J*_CF_ = 17.4, 8.1 Hz), 124.58, 117.00 (dd, *J*_CF_ = 24.2, 9.0 Hz), 116.70 (dd, *J*_CF_ = 24.7, 4.4 Hz), 116.61 (dd, *J*_CF_ = 24.2, 8.7 Hz), 113.63, 113.31, 63.06 (d, *J*_CF_ = 2.5 Hz), 28.98, 26.43, 21.17; HRMS (ESI^+^): *m*/*z* calcd for C_18_H_18_F_2_N_4_O [M + H]^+^ 345.1521, found 345.1530.

(*E*)-2-(6-((2,6-difluorobenzyl)oxy)-3,4-dihydronaphthalen-1(2*H*)-ylidene)hydrazine-1-carboximidamide(**1F**). Yield: 50.9%, 0.143 g; ^1^H NMR (600 MHz, *DMSO-d_6_*) δ 11.17 (s, 1H), 8.30 (d, *J* = 8.6 Hz, 1H), 8.10–7.60 (br s, 3H), 7.53 (tt, *J* = 8.4, 6.6 Hz, 1H), 7.18 (t, *J* = 8.0 Hz, 2H), 6.96–6.80 (m, 2H), 5.14 (s, 2H), 2.74 (t, *J* = 6.0 Hz, 2H), 2.66 (t, *J* = 6.5 Hz, 2H), 1.84 (p, *J* = 6.3 Hz, 2H). ^13^C NMR (151 MHz, *DMSO-d_6_*) δ 161.19 (d, *J*_CF_ = 256.8 Hz), 161.19 (d, *J*_CF_ = 241.6 Hz), 159.09, 156.01, 151.07, 142.16, 131.81 (t, *J*_CF_= 10.4 Hz), 127.45, 124.63, 113.47, 113.22, 112.06 (t, *J*_CF_ = 19.4 Hz), 111.88 (d, *J*_CF_ = 4.6 Hz), 111.74 (d, *J*_CF_ = 4.5 Hz), 57.61 (t, *J*_CF_ = 3.6 Hz), 29.00, 26.46, 21.17. HRMS (ESI^+^): *m*/*z* calcd for C_18_H_18_F_2_N_4_O [M + H]^+^ 345.1521, found 345.1526.

(*E*)-2-(6-((3,5-difluorobenzyl)oxy)-3,4-dihydronaphthalen-1(2*H*)-ylidene)hydrazine-1-carboximidamide(**1G**). Yield: 86.4%, 0.257 g; ^1^H NMR (600 MHz, *DMSO-d6*) δ 11.13 (s, 1H), 8.28 (d, *J* = 8.8 Hz, 1H), 8.10–7.50 (br s, 3H), 7.23–7.15 (m, 3H), 6.92–6.83 (m, 2H), 5.18 (s, 2H), 2.73 (t, *J* = 6.0 Hz, 2H), 2.65 (t, *J* = 6.5 Hz, 2H), 1.84 (p, *J* = 6.3 Hz, 2H); ^13^C NMR (151 MHz, *DMSO-d_6_*) δ 162.45 (d, *J*_CF_ = 246.4 Hz), 162.36 (d, *J*_CF_ = 246.6 Hz), 158.90, 155.97, 151.09, 142.11, 141.64 (t, *J*_CF_ = 9.3 Hz), 127.43, 124.56, 113.68, 113.46, 110.48 (d, *J*_CF_ = 5.2 Hz), 110.34 (d, *J*_CF_ = 5.2 Hz), 103.19 (t, *J*_CF_ = 25.7 Hz), 67.78, 28.98, 26.41, 21.16; HRMS (ESI^+^): *m*/*z* calcd for C_18_H_18_F_2_N_4_O [M + H]^+^ 345.1521, found 345.1527.

(*E*)-2-(6-((3,4-difluorobenzyl)oxy)-3,4-dihydronaphthalen-1(2*H*)-ylidene)hydrazine-1-carboximidamide(**1H**). Yield: 80.0%, 0.262 g; ^1^H NMR (600 MHz, *DMSO-d_6_*) δ 11.13 (s, 1H), 8.28 (d, *J* = 8.7 Hz, 1H), 7.95–7.64 (br s, 3H), 7.53 (ddd, *J* = 11.6, 7.9, 2.2 Hz, 1H), 7.45 (dt, *J* = 10.8, 8.4 Hz, 1H), 7.34–7.29 (m, 1H), 6.93–6.82 (m, 2H), 5.13 (s, 2H), 2.73 (t, *J* = 5.9 Hz, 2H), 2.65 (t, *J* = 6.5 Hz, 2H), 1.84 (p, *J* = 6.3 Hz, 2H); ^13^C NMR (151 MHz, *DMSO-d_6_*) δ 159.07, 155.97, 151.12, 149.34 (dd, *J*_CF_ = 245.7, 12.7 Hz), 149.00 (dd, *J*_CF_ = 245.6, 12.6 Hz), 142.09, 134.70 (dd, *J*_CF_ = 5.8, 3.5 Hz), 127.40, 124.60 (dd, *J*_CF_ = 6.8, 3.4 Hz), 124.44, 117.55 (d, *J*_CF_ = 17.2 Hz), 116.80 (d, *J*_CF_ = 17.6 Hz), 113.71, 113.41, 67.88, 28.99, 26.43, 21.18; HRMS (ESI^+^): *m*/*z* calcd for C_18_H_18_F_2_N_4_O [M + H]^+^ 345.1521, found 345.1526.

(*E*)-2-(6-((3-(trifluoromethyl)benzyl)oxy)-3,4-dihydronaphthalen-1(2*H*)-ylidene)hydrazine-1-carboximidamide(**1I**). Yield: 72.6%, 0.251 g; ^1^H NMR (600 MHz, *DMSO-d_6_*) δ 11.13 (s, 1H), 8.31-8.26 (m, 1H), 8.01–7.79 (br s, 3H), 7.82 (d, *J* = 2.0 Hz, 1H), 7.77 (d, *J* = 8.0, 1.7 Hz, 1H), 7.70 (dd, *J* = 8.0, 1.7 Hz, 1H), 7.64 (t, *J* = 7.7 Hz, 1H), 6.90 (t, *J* = 8.0 Hz, 2H), 5.25 (s, 2H), 2.74 (t, *J* = 6.0 Hz, 2H), 2.66 (t, *J* = 6.5 Hz, 2H), 1.84 (p, *J* = 6.3 Hz, 2H); ^13^C NMR (151 MHz, *DMSO-d_6_*) δ 159.11, 155.97, 151.11, 142.10, 138.44, 131.67, 129.59, 129.18 (d, *J*_CF_ = 31.7 Hz), 127.42, 124.58 (q, *J*_CF_ = 3.8 Hz), 124.47, 124.17 (d, *J*_CF_ = 272.3 Hz), 124.03 (q, *J*_CF_ = 3.8 Hz), 113.66, 113.47, 68.30, 28.99, 26.42, 21.18; HRMS (ESI^+^): *m*/*z* calcd for C_19_H_19_F_3_N_4_O [M + H]^+^ 377.1583, found 377.1587.

(*E*)-2-(6-((4-(trifluoromethyl)benzyl)oxy)-3,4-dihydronaphthalen-1(2*H*)-ylidene)hydrazine-1-carboximidamide(**1J**). Yield: 60.8%, 0.205 g; ^1^H NMR (600 MHz, *DMSO-d_6_*) δ 8.21 (d, *J* = 8.5 Hz, 1H), 7.76 (d, *J* = 8.0 Hz, 2H), 7.67 (d, *J* = 8.0 Hz, 2H), 7.50–6.85 (br s, 4H), 6.87–6.81 (m, 2H), 5.25 (s, 2H), 2.71 (t, *J* = 5.9 Hz, 2H), 2.66 (t, *J* = 6.5 Hz, 2H), 1.80 (p, *J* = 6.2 Hz, 2H); ^13^C NMR (151 MHz, *DMSO-d_6_*) δ 158.45, 157.07, 149.89, 141.93, 141.33, 128.30 (d, *J*_CF_ = 31.7 Hz), 127.98 (2C), 126.75, 125.73, 125.32 (q, *J*_CF_ = 3.9 Hz, 2C), 124.23 (d, *J*_CF_ = 271.9 Hz), 113.51, 113.44, 68.24, 29.31, 26.31, 21.47; HRMS (ESI^+^): *m*/*z* calcd for C_19_H_19_F_3_N_4_O [M + H]^+^ 377.1583, found 377.1587.

(*E*)-2-(6-((3,5-bis(trifluoromethyl)benzyl)oxy)-3,4-dihydronaphthalen-1(2*H*)-ylidene)hydrazine-1-carboximidamide(**1K**). Yield: 82.7%, 0.332 g; ^1^H NMR (600 MHz, *DMSO-d_6_*) δ 11.13 (s, 1H), 8.30 (d, *J* = 8.6 Hz, 1H), 8.18 (d, *J* = 1.8 Hz, 2H), 8.09 (s, 1H), 7.62–7.97 (br s, 3H), 6.95–6.90 (m, 2H), 5.35 (s, 2H), 2.75 (t, *J* = 5.9 Hz, 2H), 2.66 (t, *J* = 6.5 Hz, 2H), 1.85 (p, *J* = 6.4 Hz, 2H); ^13^C NMR (151 MHz, *DMSO-d_6_*) δ 158.85, 155.96, 151.05, 142.13, 140.53, 130.35 (q, *J*_CF_ = 32.9 Hz, 2C), 128.24 (d, *J*_CF_ = 3.4 Hz, 2C), 127.47, 124.69, 123.28 (q, *J*_CF_ = 272.8 Hz, 2C), 121.68–121.52 (m), 113.60, 113.52, 67.58, 28.98, 26.41, 21.17; HRMS (ESI^+^): *m*/*z* calcd for C_20_H_18_F_6_N_4_O [M + H]^+^ 445.1457, found 445.1458.

(*E*)-2-(6-((2-chlorobenzyl)oxy)-3,4-dihydro-1(2*H*)-naphthalenylidene)hydrazine-1-carboxamide(**1L**); Yield: 87.6%, 0.300 g; ^1^H NMR (600 MHz, *DMSO-d_6_*) δ 11.06 (s, 1H), 8.26 (d, *J* = 8.7 Hz, 1H), 8.1–7.6 (br s, 3H), 7.60–7.56 (m, 1H), 7.52–7.47 (m, 1H), 7.41–7.34 (m, 2H), 6.88–6.84 (m, 2H), 5.17 (s, 2H), 2.75–2.70 (m, 2H), 2.63 (t, *J* = 6.5 Hz, 2H), 1.85–1.79 (m, 2H); ^13^C NMR (151 MHz, *DMSO-d_6_*) δ 159.65, 156.35, 151.59, 142.60, 134.54, 133.14, 130.74, 130.42, 129.85, 127.86, 127.83, 124.92, 114.10, 113.69, 67.31, 29.43, 26.84, 21.62; HRMS (ESI^+^): *m*/*z* calcd for C_18_H_19_ClN_4_O [M + H]^+^ 343.1320, found 343.1332.

(*E*)-2-(6-((3-chlorobenzyl)oxy)-3,4-dihydro-1(2*H*)-naphthalenylidene)hydrazine-1-carboxamide(**1M**). Yield: 82.2%, 0.282 g; ^1^H NMR (600 MHz, *DMSO-d_6_*) δ 11.05 (s, 1H), 8.25 (d, *J* = 8.6 Hz, 1H), 8.13–7.52 (br s, 3H), 7.50 (s, 1H), 7.42–7.36 (m, 3H), 6.87–6.83 (m, 2H), 5.14 (s, 2H), 2.73–2.69 (m, 2H), 2.62 (t, *J* = 6.5 Hz, 2H), 1.85–1.78 (m, 2H); ^13^C NMR (151 MHz, *DMSO-d_6_*) δ 159.55, 156.35, 151.58, 142.53, 139.93, 133.54, 130.82, 128.23, 127.83, 127.75, 126.63, 124.83, 114.13, 113.87, 68.69, 29.43, 26.82, 21.62. HRMS (ESI^+^): *m*/*z* calcd for C_18_H_19_ClN_4_O [M + H]^+^ 343.1320, found 343.1330.

(*E*)-2-(6-((4-chlorobenzyl)oxy)-3,4-dihydro-1(2*H*)-naphthalenylidene)hydrazine-1-carboxamide(**1N**). Yield: 57.9%, 0.198 g; ^1^H NMR (600 MHz, *DMSO-d_6_*) δ 11.05 (s, 1H), 8.24 (d, *J* = 8.8 Hz, 1H), 8.11–7.50 (br s, 3H), 7.47–7.42 (m, 4H), 6.85–6.82 (m, 2H), 5.12 (s, 2H), 2.73–2.68 (m, 2H), 2.62 (t, *J* = 6.5 Hz, 2H), 1.85-1.78 (m, 2H); ^13^C NMR (151 MHz, *DMSO-d_6_*) δ 159.63, 156.34, 151.59, 142.52, 136.38, 132.88, 129.95 (2C), 128.88 (2C), 127.81, 124.77, 114.16, 113.85, 68.79, 29.44, 26.82, 21.62; HRMS (ESI^+^): *m*/*z* calcd for C_18_H_19_ClN_4_O [M + H]^+^ 343.1320, found 343.1328.

(*E*)-2-(6-((2,4-dichlorobenzyl)oxy)-3,4-dihydro-1(2*H*)-naphthalenylidene)hydrazine-1-carboxamide(**1O**). Yield: 86.1%, 0.325 g; ^1^H NMR (600 MHz, *DMSO-d_6_*) δ 11.05 (s, 1H), 8.28–8.25 (m, 1H), 8.07–7.68 (br s, 3H), 7.67 (d, *J* = 2.1 Hz, 1H), 7.60 (d, *J* = 8.3 Hz, 1H), 7.46 (dd, *J* = 8.3, 2.1 Hz, 1H), 6.87–6.84 (m, 2H), 5.15 (s, 2H), 2.75–2.70 (m, 2H), 2.63 (t, *J* = 6.5 Hz, 2H), 1.85–1.79 (m, 2H); ^13^C NMR (151 MHz, *DMSO-d_6_*) δ 159.46, 156.34, 151.56, 142.61, 134.10, 134.05, 133.79, 131.92, 129.38, 128.02, 127.87, 125.04, 114.10, 113.73, 66.72, 29.42, 26.82, 21.60; HRMS (ESI^+^): *m*/*z* calcd for C_18_H_18_Cl_2_N_4_O [M + H]^+^ 377.0930, found 377.0935.

(*E*)-2-(6-((3,4-dichlorobenzyl)oxy)-3,4-dihydro-1(2*H*)-naphthalenylidene)hydrazine-1-carboxamide(**1P**). Yield: 80.3%, 0.303 g; ^1^H NMR (600 MHz, *DMSO-d_6_*) δ 11.04 (s, 1H), 8.25 (d, *J* = 8.7 Hz, 1H), 8.12–7.60 (br s,3H), 7.70 (d, *J* = 1.8 Hz, 1H), 7.64 (d, *J* = 8.3 Hz, 1H), 7.42 (dd, *J* = 8.3, 1.9 Hz, 1H), 6.85 (dt, *J* = 5.6, 2.4 Hz, 2H), 5.14 (s, 2H), 2.74–2.68 (m, 2H), 2.62 (t, *J* = 6.5 Hz, 2H), 1.85–1.78 (m, 2H); ^13^C NMR (151 MHz, *DMSO-d_6_*) δ 159.41, 156.33, 151.55, 142.55, 138.60, 131.53, 131.14, 130.83, 129.91, 128.27, 127.85, 124.92, 114.13, 113.89, 68.05, 29.42, 26.81, 21.60; HRMS (ESI^+^): *m*/*z* calcd for C_18_H_18_Cl_2_N_4_O [M + H]^+^ 377.0930, found 377.0937.

(*E*)-2-(6-((2-bromobenzyl)oxy)-3,4-dihydronaphthalen-1(2*H*)-ylidene)hydrazine-1-carboximidamide(**1Q**). Yield: 80.2%, 0.312 g; ^1^H NMR (600 MHz, *DMSO-d6*) δ 11.13 (s, 1H), 8.30–8.28 (m, 1H), 8.08–7.72 (br s, 3H), 7.68 (dd, *J* = 8.1, 1.2 Hz, 1H), 7.59 (dd, *J* = 7.6, 1.7 Hz, 1H), 7.43 (td, *J* = 7.5, 1.2 Hz, 1H), 7.32 (td, *J* = 7.7, 1.8 Hz, 1H), 6.87 (t, *J* = 7.5 Hz, 2H), 5.15 (s, 2H), 2.75 (t, *J* = 6.0 Hz, 2H), 2.66 (t, *J* = 6.5 Hz, 2H), 1.84 (p, *J* = 6.3 Hz, 2H); ^13^C NMR (151 MHz, *DMSO-d_6_*) δ 159.18, 155.96, 151.12, 142.14, 135.68, 132.65, 130.41, 130.22, 127.93, 127.42, 124.51, 122.95, 113.64, 113.25, 69.07, 28.98, 26.41, 21.17; HRMS (ESI^+^): *m*/*z* calcd for C_18_H_19_BrN_4_O [M + H]^+^ 387.0815, found 387.0810.

(*E*)-2-(6-((3-bromobenzyl)oxy)-3,4-dihydronaphthalen-1(2*H*)-ylidene)hydrazine-1-carboximidamide(**1R**). Yield: 71.5%, 0.255 g; ^1^H NMR (600 MHz, *DMSO-d_6_*) δ 11.12 (s, 1H), 8.28 (d, *J* = 8.6 Hz, 1H), 8.00–7.69 (br s, 3H), 7.66 (t, *J* = 1.8 Hz, 1H), 7.53 (ddd, *J* = 8.0, 2.0, 1.0 Hz, 1H), 7.46 (dt, *J* = 7.6, 1.2 Hz, 1H), 7.36 (t, *J* = 7.8 Hz, 1H), 6.90–6.83 (m, 2H), 5.15 (s, 2H), 2.73 (t, *J* = 6.0 Hz, 2H), 2.65 (t, *J* = 6.5 Hz, 2H), 1.84 (p, *J* = 6.4 Hz, 2H); ^13^C NMR (151 MHz, *DMSO-d_6_*) δ 159.11, 155.95, 151.12, 142.08, 139.73, 130.69, 130.67, 130.19, 127.40, 126.58, 124.40, 121.69, 113.68, 113.43, 68.19, 28.99, 26.41, 21.17; HRMS (ESI^+^): *m*/*z* calcd for C_18_H_19_BrN_4_O [M + H]^+^ 387.0815, found 387.0806.

(*E*)-2-(6-((4-bromobenzyl)oxy)-3,4-dihydronaphthalen-1(2*H*)-ylidene)hydrazine-1-carboximidamide(**1S**). Yield: 95.6%, 0.342 g; ^1^H NMR (600 MHz, *DMSO-d_6_*) δ 11.13 (s, 1H), 8.27 (d, *J* = 8.5 Hz, 1H), 7.98–7.68 (br s, 3H), 7.63–7.54 (m, 2H), 7.44–7.37 (m, 2H), 6.90–6.82 (m, 2H), 5.12 (s, 2H), 2.72 (t, *J* = 6.0 Hz, 2H), 2.65 (t, *J* = 6.5 Hz, 2H), 1.83 (p, *J* = 6.3 Hz, 2H); ^13^C NMR (151 MHz, *DMSO-d_6_*) δ 159.16, 155.96, 151.13, 142.07, 136.36, 131.36(2C), 129.80(2C), 127.38, 124.35, 120.97, 113.71, 113.42, 68.37, 28.99, 26.42, 21.17; HRMS(ESI^+^): *m*/*z* calcd for C_18_H_19_BrN_4_O [M + H]^+^ 387.0815, found 387.0811.

(*E*)-2-(6-((2-chloro-4-fluorobenzyl)oxy)-3,4-dihydronaphthalen-1(2*H*)-ylidene)hydrazine-1-carboximidamide(**1T**). Yield: 99.8%, 0.360 g; ^1^H NMR (600 MHz, *DMSO-d_6_*) δ 11.14 (s, 1H), 8.33–8.23 (m, 1H), 8.03–7.70 (br s, 3H), 7.66 (dd, *J* = 8.7, 6.2 Hz, 1H), 7.52 (dd, *J* = 8.8, 2.6 Hz, 1H), 7.28 (td, *J* = 8.5, 2.6 Hz, 1H), 6.88 (t, *J* = 7.8 Hz, 2H), 5.15 (s, 2H), 2.74 (t, *J* = 6.0 Hz, 2H), 2.66 (t, *J* = 6.5 Hz, 2H), 1.84 (p, *J* = 6.3 Hz, 2H); ^13^C NMR (151 MHz, *DMSO-d_6_*) δ 161.76 (d, *J*_CF_ = 248.4 Hz), 159.14, 155.97, 151.11, 142.15, 133.83 (d, *J*_CF_ = 10.6 Hz), 132.05 (d, *J*_CF_ = 9.2 Hz), 130.61 (d, *J*_CF_ = 3.4 Hz), 127.42, 124.53, 116.82 (d, *J*_CF_ = 25.3 Hz), 114.53 (d, *J*_CF_ = 21.0 Hz), 113.66, 113.25, 66.35, 28.99, 26.43, 21.17; HRMS (ESI^+^): *m*/*z* calcd for C_18_H_18_ClFN_4_O [M + H]^+^ 361.1225, found 361.1221.

(*E*)-2-(6-((3-chloro-4-fluorobenzyl)oxy)-3,4-dihydronaphthalen-1(2*H*)-ylidene)hydrazine-1-carboximidamide(**1U**). Yield:70.7%, 0.255 g; ^1^H NMR (600 MHz, *DMSO-d_6_*) δ 11.13 (s, 1H), 8.28 (d, *J* = 8.7 Hz, 1H), 8.00–7.71 (br s, 3H), 7.68 (dd, *J* = 7.2, 2.1 Hz, 1H), 7.50–7.40 (m, 2H), 6.90–6.84 (m, 2H), 5.13 (s, 2H), 2.73 (t, *J* = 6.0 Hz, 2H), 2.65 (t, *J* = 6.5 Hz, 2H), 1.84 (p, *J* = 6.3 Hz, 2H); ^13^C NMR (151 MHz, *DMSO-d_6_*) δ 159.04, 156.76 (d, *J*_CF_ = 246.7 Hz), 155.97, 151.09, 142.08, 134.87 (d, *J*_CF_ = 3.8 Hz), 129.82, 128.47 (d, *J*_CF_ = 7.5 Hz), 127.40, 124.45, 119.43 (d, *J*_CF_ = 17.9 Hz), 116.95 (d, *J*_CF_ = 20.9 Hz), 113.69, 113.42, 67.74, 28.99, 26.42, 21.18; HRMS(ESI^+^): *m*/*z* calcd for C_18_H_18_ClFN_4_O [M + H]^+^ 361.1225, found 361.1221.

(*E*)-2-(6-((4-cyanobenzyl)oxy)-3,4-dihydronaphthalen-1(2*H*)-ylidene)hydrazine-1-carboximidamide(**1V**). Yield: 90.6%, 0.302 g; ^1^H NMR (600 MHz, *DMSO-d6*) δ 11.14 (s, 1H), 8.28 (d, *J* = 8.6 Hz, 1H), 8.05–7.70 (br s, 3H), 7.88–7.84 (m, 2H), 7.66–7.59 (m, 2H), 6.90–6.84 (m, 2H), 5.26 (s, 2H), 2.73 (t, *J* = 6.0 Hz, 2H), 2.65 (t, *J* = 6.5 Hz, 2H), 1.83 (p, *J* = 6.3 Hz, 2H); ^13^C NMR (151 MHz, *DMSO-d_6_*) δ 158.97, 155.97, 151.08, 142.72, 142.11, 132.42(2C), 128.06(2C), 127.43, 124.53, 118.73, 113.68, 113.45, 110.50, 68.22, 28.98, 26.41, 21.16; HRMS (ESI^+^): *m*/*z* calcd for C_19_H_19_N_5_O [M + H]^+^ 334.1662, found 334.1659.

(*E*)-2-(6-([1,1’-biphenyl]-3-ylmethoxy)-3,4-dihydronaphthalen-1(2*H*)-ylidene)hydrazine-1-carboximidamide(**1W**). Yield: 96.2%, 0.370 g; ^1^H NMR (600 MHz, *DMSO-d6*) δ 11.14 (s, 1H), 8.28 (d, *J* = 8.5 Hz, 1H), 8.00–7.75 (br s, 3H), 7.75 (s, 1H), 7.68–7.65 (m, 2H), 7.62 (dt, *J* = 7.6, 1.6 Hz, 1H), 7.52–7.43 (m, 4H), 7.39–7.36 (m, 1H), 6.93–6.88 (m, 2H), 5.22 (s, 2H), 2.73 (t, *J* = 6.0 Hz, 2H), 2.65 (t, *J* = 6.5 Hz, 2H), 1.84 (p, *J* = 6.3 Hz, 2H); ^13^C NMR (151 MHz, *DMSO-d_6_*) δ 159.39, 155.96, 151.17, 142.07, 140.34, 139.90, 137.61, 129.11, 128.97(2C), 127.57, 127.38, 126.77, 126.71(2C), 126.23, 126.06, 124.26, 113.73, 113.46, 69.17, 29.01, 26.43, 21.19; HRMS (ESI^+^): *m*/*z* calcd for C_24_H_24_N_4_O [M + H]^+^ 385.2022, found 385.2016.

(*E*)-2-(6-(4-isopropylphenoxy)-3,4-dihydronaphthalen-1(2*H*)-ylidene)hydrazine-1-carboximidamide(**1X**). Yield: 83.6%, 0.271 g; ^1^H NMR (600 MHz, *DMSO-d6*) δ 8.07 (d, *J* = 8.6 Hz, 1H), 7.35 (d, *J* = 7.6 Hz, 2H), 7.25 (d, *J* = 7.7 Hz, 2H), 6.81–6.70 (m, 2H), 5.78 (s, 2H), 5.32 (s, 2H), 5.03 (s, 2H), 2.88 (p, *J* = 6.9 Hz, 1H), 2.68 (dt, *J* = 13.4, 6.2 Hz, 4H), 1.73 (p, *J* = 6.2 Hz, 2H), 1.20 (d, *J* = 6.9 Hz, 6H); ^13^C NMR (151 MHz, *DMSO-d6*) δ 159.03, 157.71, 148.00, 147.76, 139.81, 134.56, 127.84(2C), 127.69, 126.29(2C), 125.41, 113.30, 113.21, 68.97, 33.18, 29.92, 26.25, 23.87(2C), 22.07; HRMS (ESI^+^): *m*/*z* calcd for C_21_H_26_N_4_O [M + H]^+^ 351.2179, found 351.2172.

(*E*)-2-(6-butoxy-3,4-dihydronaphthalen-1(2*H*)-ylidene)hydrazine-1-carboximidamide(**2A**). Yield: 89.2%, 0.245 g; ^1^H NMR (600 MHz, *DMSO-d_6_*) δ 11.14 (s, 1H), 8.25 (d, *J* = 8.8 Hz, 1H), 7.95–7.63 (br s, 3H), 6.80–6.73 (m, 2H), 3.98 (t, *J* = 6.5 Hz, 2H), 2.72 (t, *J* = 6.0 Hz, 2H), 2.65 (t, *J* = 6.5 Hz, 2H), 1.83 (p, *J* = 6.3 Hz, 2H), 1.71–1.64 (m, 2H), 1.46–1.38 (m, 2H), 0.92 (t, *J* = 7.4 Hz, 3H); ^13^C NMR (151 MHz, *DMSO-d_6_*) δ 159.77, 155.98, 151.25, 142.06, 127.32, 123.86, 113.50, 112.80, 67.15, 30.70, 29.01, 26.47, 21.21, 18.69, 13.67; HRMS (ESI^+^): *m*/*z* calcd for C_15_H_22_N_4_O [M + H]^+^ 275.1866, found 275.1864.

(*E*)-2-(6-(pentyloxy)-3,4-dihydronaphthalen-1(2*H*)-ylidene)hydrazine-1-carboximidamide(**2B**). Yield: 78.7%, 0.267 g; ^1^H NMR (600 MHz, *DMSO-d_6_*) δ 11.13 (s, 1H), 8.24 (d, *J* = 8.8 Hz, 1H), 7.95–7.64 (br s, 3H), 6.80–6.71 (m, 2H), 3.97 (t, *J* = 6.5 Hz, 2H), 2.72 (t, *J* = 6.0 Hz, 2H), 2.65 (t, *J* = 6.5 Hz, 2H), 1.83 (p, *J* = 6.3 Hz, 2H), 1.74–1.66 (m, 2H), 1.42–1.30 (m, 4H), 0.89 (t, *J* = 7.1 Hz, 3H); ^13^C NMR (151 MHz, *DMSO-d6*) δ 159.76, 155.96, 151.25, 142.06, 127.32, 123.85, 113.48, 112.80, 67.44, 29.00, 28.32, 27.67, 26.46, 21.86, 21.21, 13.89; HRMS (ESI^+^): *m*/*z* calcd for C_16_H_24_N_4_O [M + H]^+^ 289.2022, found 289.2014.

(*E*)-2-(6-(hexyloxy)-3,4-dihydronaphthalen-1(2*H*)-ylidene)hydrazine-1-carboximidamide(**2C**). Yield: 83.7%, 0.253 g; ^1^H NMR (600 MHz, *DMSO-d_6_*) δ 11.13 (s, 1H), 8.24 (d, *J* = 8.7 Hz, 1H), 7.96–7.64 (br s, 3H), 6.79–6.72 (m, 2H), 3.97 (t, *J* = 6.5 Hz, 2H), 2.72 (t, *J* = 5.9 Hz, 2H), 2.65 (t, *J* = 6.5 Hz, 2H), 1.83 (p, *J* = 6.3 Hz, 2H), 1.72–1.66 (m, 2H), 1.43–1.36 (m, 2H), 1.29 (h, *J* = 3.8 Hz, 4H), 0.91–0.82 (m, 3H); ^13^C NMR (151 MHz, *DMSO-d6*) δ 159.76, 155.96, 151.25, 142.06, 127.31, 123.85, 113.49, 112.80, 67.45, 30.97, 29.00, 28.60, 26.46, 25.15, 22.05, 21.21, 13.89; HRMS (ESI^+^): *m*/*z* calcd for C_17_H_26_N_4_O [M + H]^+^ 303.2179, found 303.2176.

(*E*)-2-(6-(heptyloxy)-3,4-dihydronaphthalen-1(2*H*)-ylidene)hydrazine-1-carboximidamide(**2D**). Yield: 88.9%, 0.281 g; ^1^H NMR (600 MHz, *DMSO-d_6_*) δ 11.11 (s, 1H), 8.24 (d, *J* = 8.8 Hz, 1H), 7.94–7.62 (br s, 3H), 6.78–6.72 (m, 2H), 3.97 (t, *J* = 6.5 Hz, 2H), 2.72 (t, *J* = 6.0 Hz, 2H), 2.64 (t, *J* = 6.5 Hz, 2H), 1.83 (p, *J* = 6.3 Hz, 2H), 1.69 (p, *J* = 6.7 Hz, 2H), 1.43–1.35 (m, 2H), 1.34–1.20 (m, 6H), 0.86 (t, *J* = 6.9 Hz, 3H); ^13^C NMR (151 MHz, *DMSO-d6*) δ 159.77, 155.95, 151.27, 142.07, 127.32, 123.85, 113.50, 112.80, 67.45, 31.23, 29.01, 28.64, 28.43, 26.45, 25.46, 22.04, 21.22, 13.93; HRMS (ESI^+^): *m*/*z* calcd for C_18_H_28_N_4_O [M + H]^+^ 317.2335, found 317.2330.

(*E*)-2-(6-(octyloxy)-3,4-dihydronaphthalen-1(2*H*)-ylidene)hydrazine-1-carboximidamide(**2E**). Yield:75.0%, 0.248 g; ^1^H NMR (600 MHz, *DMSO-d_6_*) δ 11.13 (s, 1H), 8.24 (d, *J* = 8.8 Hz, 1H), 7.95–7.62 (br s, 3H), 6.78–6.72 (m, 2H), 3.97 (t, *J* = 6.5 Hz, 2H), 2.72 (t, *J* = 6.0 Hz, 2H), 2.65 (t, *J* = 6.5 Hz, 2H), 1.83 (p, *J* = 6.3 Hz, 2H), 1.69 (p, *J* = 6.7 Hz, 2H), 1.42–1.35 (m, 2H), 1.33–1.19 (m, 8H), 0.88–0.82 (m, 3H); ^13^C NMR (151 MHz, *DMSO-d6*) δ 159.76, 155.96, 151.24, 142.04, 127.31, 123.84, 113.49, 112.79, 67.44, 31.23, 29.01, 28.72, 28.65, 28.63, 26.46, 25.49, 22.07, 21.21, 13.93; HRMS (ESI^+^): *m*/*z* calcd for C_19_H_30_N_4_O [M + H]^+^ 331.2492, found 331.2483.

(*E*)-2-(6-(nonyloxy)-3,4-dihydronaphthalen-1(2*H*)-ylidene)hydrazine-1-carboximidamide(**2F**). Yield: 30.0%, 0.103 g; ^1^H NMR (600 MHz, *DMSO-d6*) δ 11.11 (s, 1H), 8.24 (d, *J* = 8.8 Hz, 1H), 7.94–7.60 (br s, 3H), 6.77 (dd, J = 8.8, 2.6 Hz, 1H), 6.74 (d, J = 2.6 Hz, 1H), 3.97 (t, *J* = 6.5 Hz, 2H), 2.72 (t, *J* = 6.0 Hz, 2H), 2.64 (t, *J* = 6.5 Hz, 2H), 1.83 (p, *J* = 6.2 Hz, 2H), 1.69 (p, *J* = 6.7 Hz, 2H), 1.42–1.35 (m, 2H), 1.33–1.19 (m, 10H), 0.87–0.82 (m, 3H); ^13^C NMR (151 MHz, *DMSO-d6*) δ 159.77, 155.95, 151.25, 142.05, 127.32, 123.85, 113.49, 112.79, 67.44, 31.27, 29.01, 28.96, 28.76, 28.65, 28.63, 26.46, 25.48, 22.09, 21.22, 13.93; HRMS (ESI^+^): *m*/*z* calcd for C_20_H_32_N_4_O [M + H]^+^ 345.2648, found 345.2639.

(*E*)-2-(6-(decyloxy)-3,4-dihydronaphthalen-1(2*H*)-ylidene)hydrazine-1-carboximidamide(**2G**). Yield: 82.7%, 0.296 g; ^1^H NMR (600 MHz, *DMSO-d_6_*) δ 11.10 (s, 1H), 8.24 (d, *J* = 8.7 Hz, 1H), 7.94–7.63 (br s, 3H), 6.78–6.72 (m, 2H), 3.97 (t, *J* = 6.5 Hz, 2H), 2.72 (t, *J* = 6.0 Hz, 2H), 2.64 (t, *J* = 6.5 Hz, 2H), 1.83 (p, *J* = 6.3 Hz, 2H), 1.69 (p, *J* = 6.7 Hz, 2H), 1.42–1.35 (m, 2H), 1.33–1.19 (m, 12H), 0.84 (t, 3H); ^13^C NMR (151 MHz, *DMSO-d_6_*) δ 159.77, 155.95, 151.25, 142.05, 127.32, 123.85, 113.49, 112.79, 67.44, 31.29, 29.02, 29.00, 28.95, 28.75, 28.69, 28.63, 26.45, 25.48, 22.09, 21.22, 13.93; HRMS (ESI^+^): *m*/*z* calcd for C_21_H_34_N_4_O [M + H]^+^ 359.2805, found 359.2791.

(*E*)-2-(6-(undecyloxy)-3,4-dihydronaphthalen-1(2*H*)-ylidene)hydrazine-1-carboximidamide(**2H**). Yield: 71.5%, 0.266 g; ^1^H NMR (600 MHz, *DMSO-d_6_*) δ 11.11 (s, 1H), 8.24 (d, *J* = 8.8 Hz, 1H), 7.95–7.63 (br s, 3H), 6.79–6.71 (m, 2H), 3.97 (t, *J* = 6.5 Hz, 2H), 2.72 (t, *J* = 6.0 Hz, 2H), 2.64 (t, *J* = 6.5 Hz, 2H), 1.83 (p, *J* = 6.3 Hz, 2H), 1.69 (p, J = 6.7 Hz, 2H), 1.38 (td, *J* = 9.3, 8.5, 4.8 Hz, 2H), 1.33–1.18 (m, 14H), 0.84 (t, *J* = 7.0 Hz, 3H); ^13^C NMR (151 MHz, *DMSO-d_6_*) δ 159.77, 155.95, 151.24, 142.04, 127.32, 123.85, 113.49, 112.79, 67.44, 31.30, 29.02, 28.99 (3C), 28.75, 28.71, 28.63, 26.45, 25.48, 22.09, 21.22, 13.93; HRMS (ESI^+^): *m*/*z* calcd for C_22_H_36_N_4_O [M + H]^+^ 373.2961, found 373.2946.

(*E*)-2-(6-((3-methylbut-2-en-1-yl)oxy)-3,4-dihydronaphthalen-1(2*H*)-ylidene)hydrazine-1-carboximidamide(**2I**). Yield: 22.8%, 0.065 g; ^1^H NMR (600 MHz, *DMSO-d_6_*) δ 11.13 (s, 1H), 8.24 (d, *J* = 8.6 Hz, 1H), 7.93–7.63 (br s, 3H), 6.80–6.75 (m, 2H), 5.42 (ddt, *J* = 6.7, 5.2, 1.4 Hz, 1H), 4.54 (d, *J* = 6.7 Hz, 2H), 2.72 (t, *J* = 6.0 Hz, 2H), 2.65 (t, *J* = 6.5 Hz, 2H), 1.83 (p, *J* = 6.4 Hz, 2H), 1.72 (dd, *J* = 19.9, 1.4 Hz, 6H); ^13^C NMR (151 MHz, *DMSO-d_6_*) δ 159.54, 155.96, 151.25, 142.02, 137.27, 127.28, 123.88, 119.74, 113.66, 113.01, 64.32, 29.03, 26.45, 25.41, 21.22, 18.01; HRMS (ESI^+^): *m*/*z* calcd for C_16_H_22_N_4_O [M + H]^+^ 287.1866, found 287.1859.

2-((*E*)-6-(((*E*)-3,7-dimethylocta-2,6-dien-1-yl)oxy)-3,4-dihydronaphthalen-1(2*H*)-ylidene)hydrazine-1-carboximidamide(**2J**). Yield: 86.0%, 0.305 g; ^1^H NMR (600 MHz, *DMSO-d_6_*) δ 8.05 (d, *J* = 8.7 Hz, 1H), 6.70 (dd, *J* = 8.7, 2.7 Hz, 1H), 6.66 (d, *J* = 2.7 Hz, 1H), 6.10–5.60 (br s, 2H), 5.56–5.30 (m, 3H), 5.07 (t, *J* = 6.9 Hz, 1H), 4.52 (d, *J* = 6.5 Hz, 2H), 2.68 (dt, *J* = 12.1, 6.1 Hz, 4H), 2.08 (q, *J* = 7.2 Hz, 2H), 2.03 (dd, *J* = 8.8, 6.2 Hz, 2H), 1.72 (d, *J* = 22.6 Hz, 5H), 1.63 (s, 3H), 1.56 (s, 3H); ^13^C NMR (151 MHz, *DMSO-d_6_*) δ 158.89, 157.83, 147.99, 139.94, 139.84, 131.00, 127.30, 125.43, 123.78, 119.88, 113.23, 113.03, 64.21, 38.94, 29.92, 26.26, 25.82, 25.46, 22.06, 17.55, 16.33; HRMS (ESI^+^): *m*/*z* calcd for C_21_H_30_N_4_O [M + H]^+^ 355.2492, found 355.2479.

### 3.2. Antibacterial Evaluation

#### 3.2.1. MIC and MBC Testing

The minimum inhibitory concentration (MIC) of all synthesized compounds was tested by the broth microdilution method [42]. In brief, compounds dissolved in DMSO were diluted twice continuously in a 96-well plate with MH broth. The ESKAPE pathogens (*E. coli* ATCC 25922, *S. aureus* ATCC 29213, *K. pneumoniae* ATCC 700603, *A. baumannii* ATCC 19606, *P. aeruginosa* ATCC 27853, and *E. faecium* ATCC 35667), clinical *S. aureus* isolates (LMY45, LMY46, LMY47, and LMY48) and methicillin-resistant *Staphylococcus aureus* (MRSA-1 and MRSA-2) were cultured in MH broth to logarithmic growth phase and then diluted to 1 × 10^6^ CFU/mL in MH broth. Subsequently, the diluted bacterial solution was mixed with the diluted compound solution in a 96-well plate and incubated at 37 °C for a duration of 18 h. The MIC of the compounds was recorded as the lowest concentration at which no visible growth was observed. Subsequently, the MBC values were also determined (only for the compounds with MIC values of ≤32 µg/mL). The levofloxacin or vancomycin was used as a positive control and DMSO (0.1%) was used as a negative control. Each concentration was replicated thrice.

#### 3.2.2. Growth Curve and Bactericidal Time-Kill Kinetics Assay

The growth inhibition and time-dependent killing abilities of **2D** were evaluated against *S. aureus* (ATCC 29213) and MRSA-2. After culturing the strains in MH broth until the logarithmic growth phase, they were diluted with MH broth to 1 × 10^6^ CFU/mL. Subsequently, different final concentrations of **2D** (0.5, 1, 2, 4, and 8 µg/mL) and vancomycin (0.5 and 1 µg/mL) were added to a diluted bacterial suspension and incubated at 37 °C, respectively. OD_600_ values were measured at predetermined time intervals (0, 1, 2, 4, 6, 8, 10, 12, and 24 h).

*S. aureus* (ATCC 29213) and MRSA-2 were cultivated in MH broth until the logarithmic growth phase and then diluted to 1 × 10^7^ CFU/mL, respectively. Subsequently, different final concentrations of **2D** (2, 4, and 8 µg/mL) and vancomycin (2 and 4 µg/mL) were added to a diluted bacterial suspension and incubated at 37 °C. After a specified time interval (0, 1, 2, 4, 8, 12, and 24 h), the culture suspension was serially diluted in 0.9% saline over a concentration range from 10^−1^ to 10^−9^. A volume of 100 μL of the obtained diluent and culture solution was dispensed onto a sterile MH agar plate. The plate was subsequently cultured at 37 °C for a duration of 24 h. The total number of colonies was enumerated and expressed as log_10_(CFU/mL).

#### 3.2.3. Biofilm Formation Inhibition and Elimination Assay

The effect of **2D** on the biofilm formation of MRSA was evaluated. MRSA-2 was cultured in MH broth for a period of 3–5 h, after which it was diluted to a density of 1 × 10^6^ CFU/mL with MH broth. In 96-well plates, different concentrations of **2D** and vancomycin were mixed with bacterial suspension and subsequently incubated at 37 °C for 24 h. After the removal of the suspended bacteria, the well plates were washed thrice with PBS. Then, 150 μL of methanol was added and fixed for 30 min. Subsequent to air-drying, 150 μL of a 0.1% crystal violet aqueous solution was added. Following a 15 min staining period, the excess crystal violet was removed by rinsing with flowing water. Finally, 150 μL of anhydrous ethanol was added and the mixture was incubated for 20 min. The optical density at 575 nm was detected.

The ability of **2D** to eliminate biofilm was evaluated. MRSA-2 (1 × 10^6^ CFU/mL) was incubated in 96-well plates for 24 h until biofilm formation. The planktonic bacteria were discarded and washed thrice with PBS. Subsequently, various concentrations of **2D** and vancomycin were added and cultivated at 37 °C for 24 h. According to the above method, the OD_575 nm_ value was detected after the following steps: PBS washing, methanol fixation, crystal violet staining, and ethanol incubation.

### 3.3. Mode of Action Study

#### 3.3.1. Scanning Electron Microscopy (SEM) Analysis

MRSA-2 was cultivated to reach the logarithmic growth phase, washed thrice with PBS (pH = 7.2–7.4), and then resuspended to a bacterial concentration of 1×10^8^ CFU/mL. The bacterial suspension was treated with **2D** (2, 4, and 8 μg/mL) and vancomycin (4 μg/mL), followed by incubation at 37 °C for 3 h. Subsequently, the bacterial precipitate was collected via centrifugation and washed. The bacterial precipitate was incubated in 2.5% glutaraldehyde fixative at room temperature for 2 h, and then transferred to 4 °C overnight in the dark. Then, the samples were processed according to the following steps: washed with PBS, fixed with 1% osmium tetraoxide, and dehydrated with ethanol. After spraying with gold, the bacteria were observed by electron microscope.

#### 3.3.2. Membrane Depolarization Assay

MRSA-2 was cultivated in MH broth, with a final concentration of 1 × 10^8^ CFU/mL. After centrifugation, the supernatant was discarded. The bacterial precipitate was washed thrice and resuspended with PBS. Subsequently, the bacterial resuspension (180 μL) and the DiSC5 (10 μL, 3 μM) were incubated in the dark for 30 min. The fluorescence intensity was measured at 2 min intervals for a period of 10 min. After the fluorescence intensity was stabilized, 10 μL of **2D** (final concentrations of 1, 2, 4, and 8 μg/mL) was added, respectively. The fluorescence intensity was measured at 2 min intervals for a total duration of 30 min. The excitation/emission wavelength was 622/670 nm.

#### 3.3.3. Fluorescence Microscopy Assay

MRSA-2 was cultivated to the logarithmic growth phase, then washed with PBS and resuspended in PBS until the concentration of bacterial suspension reached 1 × 10^8^ CFU/mL. The compound **2D** (final concentration of 4 and 8 μg/mL) and vancomycin (4 μg/mL) were mixed with the bacterial suspension and incubated at 37 °C for 2 h. Thereafter, the samples were incubated with DAPI (10 μM) and PI (10 μM) in the dark for 15 min. The bacterial cells were then subjected to observation under a fluorescence microscope. DMSO served as the negative controls.

#### 3.3.4. Inner Membrane Permeabilization Assay

MRSA-2 bacterial precipitates in the logarithmic growth phase were collected by centrifugation, washed, and resuspended in PBS to a density of 1 × 10^6^ CFU/mL. PI (10 μM, 40 μL) was added to a black 96-well plate along with 150 μL of bacterial suspension. After an incubation period of 30 min, fluorescence was detected at excitation wavelengths of 535 nm and emission wavelengths of 617 nm at 5 min intervals until a stable state was reached. Various concentrations of **2D** and vancomycin were then added and the fluorescence intensity was immediately detected every 5 min.

#### 3.3.5. Bacterial DNA and Protein Leakage Experiment

MRSA-2 was cultured to logarithmic growth phase. The bacterial precipitate was collected, washed twice with PBS (pH = 7.2–7.4), and resuspended in PBS to 1 × 10^7^ CFU/mL. **2D** (1, 2, 4, and 8 μg/mL) and vancomycin (1 μg/mL) were subsequently added and incubated for 4 h at 37 °C. After centrifugation of 1 mL of the suspension, the supernatant was collected. The protein level was measured by BCA protein concentration kits (Servicebio, Hubei, China), and the DNA level was detected by microspectrophotometer.

#### 3.3.6. Predicted Binding Mode of **2D** in DHFR

Preliminary docking was performed to evaluate whether binding to DHFR might account for the bactericidal effect of the compound **2D**. All docking studies were carried out using Discovery Studio 2019 Client (Accelrys, San Diego, CA, USA). The crystal structure data were obtained from the protein data bank (https://www.rcsb.org/structure/6P9Z (accessed on 6 May 2025)). Enzyme structures were checked for missing atoms, bonds, and contacts. Hydrogen atoms were added to the enzyme structure. Water molecules and bound ligands were manually deleted. The structures of compounds were sketched in 2D (2 Dimensions) and converted into 3D (3 Dimensions) using the DS molecule editor. Automated docking studies were carried out to investigate the binding mode of **2D** utilizing DS-CDOCKER protocol. The pose with the top-CDOCKER_ENERGY was chosen for analyzing the binding features of **2D** with DHFR. Dihydrofolate reductase inhibitor trimethoprim was used as a control.

### 3.4. Safety Assay

The sterile defibrillated sheep blood cells were centrifuged (5000 rpm, 5 min) and then washed three times with 0.9% saline. Subsequently, 1 μL of different concentrations of **2D** (0.5–128 μg/mL) was mixed with 999 μL of red blood cell suspension. After incubation at 37 °C for 1 h, the mixed solution was centrifuged. The absorbance of the supernatant at 570 nm was measured. Triton X-100 (0.1%) was used as a positive control, and DMSO was used as a negative control. The hemolysis rate was calculated according to the following formula:

Hemolysis rate (%) = (OD_Treatment_ − OD_Negative_)/(OD_Positive_ − OD_Negative_) × 100% (1)

The CCK-8 method was used to evaluate the toxicity of **2D** to four cell lines (lung cancer cell line (A549), human liver cell line (HepG2), human embryonic kidney 293T cells (HEK 293-T), and human colon adenocarcinoma cell line (Caco-2)). Cells with a density of 1 × 10^5^ cells/well were added to 96-well plates and then incubated for 24 h at 5% CO_2_ and 37 °C. Subsequently, after treatment with different concentrations of **2D** for 24 h, the CCK-8 kit was added according to the prescribed procedure. Following an incubation at 37 °C for 0.5 h, the absorbances at 450 nm were detected. The hemolysis rate was calculated according to the following formula:

Cytotoxic (%) = (A_test_/A_control_) × 100% (2)

### 3.5. In Vivo Infection Model

The MRSA-induced mouse skin abscess model was used to assess the in vivo antibacterial effects of **2D**. Female KM mice, aged 4–6 weeks and weighing 20 ± 2 g, were purchased from the Laboratory Animal Center, Lanzhou Institute of Veterinary Medicine, Chinese Academy of Agricultural Sciences. The animal experiments were approved by the Ethics Committee of Research Involving Animals of the Lanzhou Institute of Husbandry and Pharmaceutical Science of CAAS (Approval number: 2025-03) and performed in accordance with the guidelines of the Ethics Committee for Animal Experiments. Prior to the formal experiment, all mice were acclimated for 5 days. The mice were kept in a pathogen-free environment with a 12 h light/12 h dark cycle, 50% ± 10% humidity, and a temperature of 24 °C ± 2 °C. After removing the dorsal hair, the mice were randomly divided into six groups, with six mice in each group: a control group with no MRSA infection and no treatment, a model group with MRSA infection only, a solvent group with MRSA infection and solvent (75% normal saline, 10% DMSO, 5% Tween-80, 10% PEG 300), a vancomycin group with 5 mg/kg doses, and a **2D** group with 5 mg/kg and 10 mg/kg doses. The depilation method employed was as follows: depilatory paste was applied to the designated area for 10 min, after which it was thoroughly washed with 0.9% saline. The mice were semi-anesthetized by intraperitoneal injection with sodium Ulatan (TCI, 20%, 100 μL). Subcutaneous abscesses were induced by injecting 60 μL of MRSA-2 bacterial suspension (1 × 10^8^ CFU/mL) into the back of anesthetized mice, followed by an injection of 60 μL of **2D**, vancomycin, or solvent 2 h postinfection. After a 24 h interval, the administration was repeated, and 24 h after the last administration, the volume of abscess was measured and calculated. Then, the skin of the infected site and main organs were collected for tissue bacterial load and pathological detection.

### 3.6. Statistical Analysis

The results of all experiments are presented as the mean ± SD. Statistical significance was analyzed by GraphPad Prism 9 software (GraphPad Software, San Diego, CA, USA) with unpaired Student’s t-test or non-parametric one-way ANOVA. Significant values are represented by an asterisk: * p < 0.05, ** p < 0.01, *** p < 0.001, **** p < 0.0001.

## 4. Conclusions

A total of 34 novel aminoguanidine indole derivatives were designed, synthesized, and identified. The synthesized compounds showed better activity against Gram-positive bacteria than Gram-negative bacteria. Compounds **1L**, **1M**, **1S**, **1U**, **1X**, **2A**, **2D**–**2F**, and **2I** demonstrated strong antibacterial activity against *S. aureus*, including MRSA, with MIC values ranging from 0.5 to 4 μg/mL. Compound **2D**, with the most antibacterial activity, demonstrated a favorable anti-MRSA effect in vivo, similar to that of vancomycin. The main antibacterial mechanisms of **2D** may be related to the disruption of the membrane integrity and inhibition of dihydrofolate reductase. Furthermore, the hemolytic activity and cytotoxicity of **2D** were acceptable. Therefore, compound **2D**, as an aminoguanidine tetralone derivative, may be a promising candidate for a novel broad-spectrum antibacterial agent.

## Figures and Tables

**Table 1:** Chemical structures of target compounds.

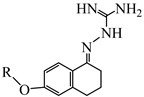							
Compounds	R	LogP	MW	Compounds	R	LogP	MW
**1A**	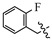	2.560	326.15	**1R**	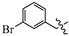	3.018	386.07
**1B**	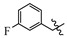	2.542	326.15	**1S**	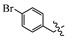	2.987	386.07
**1C**	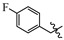	2.341	326.15	**1T**	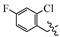	3.513	360.12
**1D**	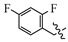	2.561	344.14	**1U**	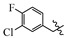	3.351	360.12
**1E**	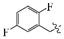	2.599	344.14	**1V**	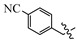	1.168	333.16
**1F**	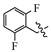	2.542	344.14	**1W**	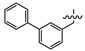	3.394	384.20
**1G**	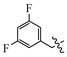	2.400	344.14	**1X**	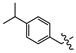	3.776	350.21
**1H**	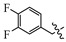	2.419	344.14	**2A**	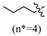	2.494	274.18
**1I**	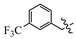	3.59	376.15	**2B**	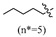	2.845	288.20
**1J**	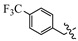	3.654	376.15	**2C**	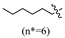	3.123	302.21
**1K**	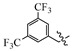	4.097	444.14	**2D**	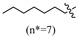	3.360	316.23
**1L**	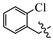	3.03	342.12	**2E**	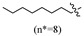	3.654	330.24
**1M**	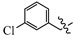	3.036	342.12	**2F**	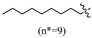	3.972	344.26
**1N**	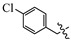	2.82	342.12	**2G**	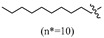	4.295	358.27
**1O**	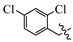	3.825	376.09	**2H**	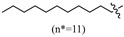	4.608	372.29
**1P**	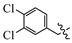	3.859	376.09	**2I**	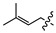	3.008	286.18
**1Q**	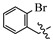	2.934	386.07	**2J**	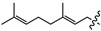	4.345	354.24

MW: molecular weight. n*: The numbers of C atoms.

**Table 2:** The MICs and MBCs of target compounds against ESKAPE pathogens.

Compounds	Strains (MIC/MBC, µg/mL)					
	*E. faecium* ATCC 35667	*S. aureus* ATCC 29213	*K. pneumoniae* ATCC 700603	*A. baumannii* ATCC 19606	*P. aeruginosa* ATCC 27853	*E. coli* ATCC 25922
**1A**	4/16	4/16	16/16	8/16	32/>32	8/8
**1B**	4/16	4/16	16/16	8/16	32/32	8/8
**1C**	4/16	4/16	16/16	8/8	32/32	8/8
**1D**	4/16	2/8	16/16	8/8	32/32	8/8
**1E**	4/16	2/16	32/32	8/8	>32/-	8/8
**1F**	8/16	4/16	32/32	16/16	>32/-	16/16
**1G**	4/8	2/8	16/16	8/8	32/-	8/16
**1H**	4/16	2/8	16/32	8/8	32/32	4/16
**1I**	4/8	2/16	32/32	8/8	32/32	4/4
**1J**	2/4	2/16	16/32	8/8	16/>32	4/4
**1K**	4/16	32/-	>32/-	>32/-	>32/-	>32/-
**1L**	4/8	2/8	16/32	4/4	32/>32	8/8
**1M**	2/8	2/16	16/>32	4/16	16/32	8/8
**1N**	2/8	2/4	16/32	4/16	16/32	4/8
**1O**	2/16	4/8	>32/-	16/16	>32/-	8/16
**1P**	2/8	2/8	32/32	8/16	>32/-	8/16
**1Q**	2/8	4/8	32/32	8/8	>32/-	4/4
**1R**	2/8	2/16	8/8	4/4	>32/-	8/8
**1S**	4/8	2/8	16/16	8/16	>32/-	4/8
**1T**	2/16	2/32	>32/-	16/32	>32/-	8/>32
**1U**	2/16	2/4	8/32	4/16	16/>32	4/16
**1V**	16/>32	8/32	32/-	16/32	>32/-	16/32
**1W**	2/16	2/16	>32/-	32/32	>32/-	32/32
**1X**	2/4	2/4	>32	4/8	32/>32	4/8
**2A**	8/32	4/>32	16/>32	8/16	32/32	16/>32
**2B**	4/16	1/8	8/8	8/8	16/32	4/8
**2C**	2/8	1/8	8/8	4/4	8/16	4/4
**2D**	2/4	0.5/4	16/32	4/16	16/32	4/8
**2E**	2/4	2/2	>32/-	8/32	32/>32	4/16
**2F**	2/4	2/8	>32/-	>32/-	>32/-	>32/-
**2G**	2/8	2/8	>32/-	>32/-	>32/-	>32/-
**2H**	4/8	>32/-	>32/-	>32/-	>32/-	>32/-
**2I**	8/32	4/32	32/>32	16/32	32/32	16/32
**2J**	2/8	4/8	>32/-	8/16	32/32	8/16
Levofloxacin	≤1/2	≤1/≤1	≤1/4	≤1/2	≤1/2	≤1/≤1
Vancomycin	2/16	1/4	-	-	-	-

-: Not detected.

**Table 3:** The MICs and MBCs of target compounds against *S. aureus* clinical isolates and MRSA.

Compounds	Strains (MIC/MBC, µg/mL)					
	LMY45	LMY46	LMY47	LMY48	MRSA-1	MRSA-2
**1A**	2/4	2/8	2/8	2/4	2/16	2/8
**1B**	2/4	2/16	2/8	4/4	2/8	2/8
**1C**	2/4	2/8	2/8	4/4	2/16	4/8
**1D**	2/4	4/32	2/8	2/4	2/32	2/32
**1E**	2/4	2/8	2/8	2/2	2/32	2/8
**1F**	4/8	4/>32	4/8	4/16	4/32	4/16
**1G**	2/4	2/8	2/8	2/2	2/16	2/16
**1H**	2/4	2/>32	2/8	2/4	2/32	2/8
**1I**	1/4	2/4	2/2	2/2	2/16	2/4
**1J**	2/2	2/16	2/4	2/2	1/8	2/32
**1K**	4/16	4/>32	4/8	4/8	16/-	8/32
**1L**	1/2	1/8	1/4	2/2	1/32	1/4
**1M**	2/2	2/4	2/4	2/8	1/32	1/4
**1N**	2/4	4/8	2/2	2/16	2/4	2/4
**1O**	2/16	4/8	≤1/4	≤1/2	1/16	2/8
**1P**	2/4	2/8	2/4	≤1/≤1	1/16	2/4
**1Q**	2/4	2/4	≤1/4	2/2	1/8	2/8
**1R**	2/4	2/4	2/8	2/16	2/16	2/4
**1S**	2/4	1/4	2/4	4/8	1/4	1/4
**1T**	1/4	1/4	1/4	4/4	1/8	2/8
**1U**	2/2	1/4	2/4	4/8	2/32	1/4
**1V**	4/8	4/32	8/32	8/8	8/16	8/8
**1W**	2/8	2/8	2/8	2/4	4/32	2/4
**1X**	2/4	2/2	2/8	2/8	4/8	1/4
**2A**	2/4	2/4	2/2	1/2	2/2	1/2
**2B**	8/16	8/16	4/8	2/2	32/32	4/4
**2C**	8/32	8/16	2/8	4/32	4/16	2/4
**2D**	2/2	4/32	1/4	2/2	1/16	1/4
**2E**	1/4	1/4	2/4	1/2	1/8	1/2
**2F**	2/4	2/4	2/8	1/2	2/2	1/2
**2G**	4/16	4/8	2/2	2/4	4/16	4/4
**2H**	4/8	2/16	2/16	4/4	4/16	4/16
**2I**	2/4	2/2	2/8	1/2	2/16	1/2
**2J**	4/16	2/>32	2/16	4/8	2/16	2/16
Vancomycin	0.5/2	1/8	1/2	1/2	1/4	1/2

-: Not detected.

**Table 4:** The hemolysis and the cytotoxicity of **2D**.

Compound	HC_50_ (µg/mL)	Cell Lines (IC_50_, µg/mL)			
		A549	HepG2	HEK 293-T	CaCo-2
**2D**	50.65	3.57	6.00	13.09	9.86
SI	101.30	7.14	12.00	26.18	19.72

Selectivity index (SI), SI = HC_50_/MIC_ATCC 29213_ or SI = IC_50_/MIC_ATCC 29213_. HC_50_ = the concentration at which 50% of the red blood cells are lysed. IC_50_ = the concentration that reduced cell viability by 50%.

## Data Availability

The raw data supporting the conclusion of this study will be made available by the authors.

## Supplementary Materials

The following supporting information can be downloaded at https://www.mdpi.com/article/10.3390/ijms26135980/s1.

## Abbreviations

MRSA, methicillin-resistant *Staphylococcus aureus*; HRMS, high-resolution mass spectrometry; NMR, nuclear magnetic resonance; MH, Mueller−Hinton; MIC, minimum inhibitory concentration; DMSO, dimethyl sulfoxide; CFU, colony forming units; PBS, phosphate-buffered saline; DAPI, 4′,6-diamidino-2-phenylindole; PI, propidium iodide; DiSC3(5), 3,3′-dipropylthiadicarbocyanine iodide; CCK-8, cell counting kit-8; H&E, hematoxylin and eosin; RBCs, red blood cells; SEM, scanning electron microscopy; SI, selectivity index; TLC, thin-layer chromatography.
