# Supplementary material for: Synthesis, Antimicrobial Activities, and Model of Action of Novel Tetralone Derivatives Containing Aminoguanidinium Moiety
Source: Int J Mol Sci. 2025 Jun 21;26(13):5980. doi: 10.3390/ijms26135980 (PMC12250302; doi:10.3390/ijms26135980)
Supplement: Supplementary file 1 [file ijms-26-05980-s001.zip › ijms-3679249-supplementary.pdf]

Supplementary Information

Synthesis, Antimicrobial Activities, and Model of Action of Novel Tetralone Derivatives Containing Aminoguanidinium Moiety

Qing-Jie Zhang[†], Yu-Xi Li[†], Wen-Bo Ge, Li-Xia Bai, Xiao Xu, Ya-Jun Yang, Xi-Wang Liu[*], Jian-Yong Li[*]

Key Lab of New Animal Drug of Gansu Province, Key Lab of Veterinary Pharmaceutical Development of Ministry of Agriculture and Rural Affairs, Lanzhou Institute of Husbandry and Pharmaceutical Sciences of Chinese Academy of Agricultural Sciences, Lanzhou 730050, China.

† These authors contributed equally to this work.

* Corresponding email: xiwangliu@126.com, lijy1971@163.com.

Corresponding author: Tel.: +86 931 2115290; fax: +86 931 2115290. E-mail address: xiwangliu@126.com (Xi-Wang Liu); lijy1971@163.com (Jian-Yong Li).

Author: Tel. +86 132 2814 0097; fax: +86 931 2115290. E-mail address: zhangqingjie0097@163.com (Qing-Jie Zhang).

Address: Lanzhou Institute of Husbandry and Pharmaceutical Sciences of CAAS, No. 335, Qilihe District, Lanzhou, 730050, P.R. China.

Funding information

This study was supported by grants from the National Key R&D Program of China (2021YFD1800900), and Science-Technology Innovation Engineering of CAAS (25-LZIHPS-02).

Supplemental Table of Contents

1. The mass errors of all compounds.
2. HRMS, ^{1}H NMR and ^{13}C NMR spectrum of the compounds.

1. The mass errors of all compounds

The mass errors of all compounds are within ± 5 ppm (Table 1).

Table 1. The mass error of the compounds.

Compounds	Calculated	Found	Error (ppm)
**1A**	327.1615	327.1628	3.97
**1B**	327.1615	327.1625	3.06
**1C**	327.1615	327.1624	2.75
**1D**	345.1521	345.1526	1.45
**1E**	345.1521	345.1530	2.61
**1F**	345.1521	345.1526	1.45
**1G**	345.1521	345.1527	1.74
**1H**	345.1521	345.1526	1.45
**1I**	377.1583	377.1587	1.06
**1J**	377.1583	377.1587	1.06
**1K**	445.1457	445.1458	0.22
**1L**	343.1320	343.1332	3.50
**1M**	343.1320	343.1330	2.91
**1N**	343.1320	343.1328	2.33
**1O**	377.0930	377.0935	1.33
**1P**	377.0930	377.0937	1.86
**1Q**	387.0815	387.0810	-1.29
**1R**	387.0815	387.0806	-2.33
**1S**	387.0815	387.0811	-1.03
**1T**	361.1225	361.1221	-1.11
**1U**	361.1225	361.1221	-1.11
**1V**	334.1662	334.1659	-0.90
**1W**	385.2022	385.2016	-1.56
**1X**	351.2179	351.2172	-1.99
**2A**	275.1866	275.1864	-0.73
**2B**	289.2022	289.2014	-2.77
**2C**	303.2179	303.2176	-0.99
**2D**	317.2335	317.2330	-1.58
**2E**	331.2492	331.2483	-2.72
**2F**	345.2648	345.2639	-2.61
**2G**	359.2805	359.2791	-3.90
**2H**	373.2961	373.2946	-4.02
**2I**	287.1866	287.1859	-2.44
**2J**	355.2492	355.2479	-3.66

ppm: parts per million.

2. HRMS, ^{1}H NMR and ^{13}C NMR spectrum of the compounds

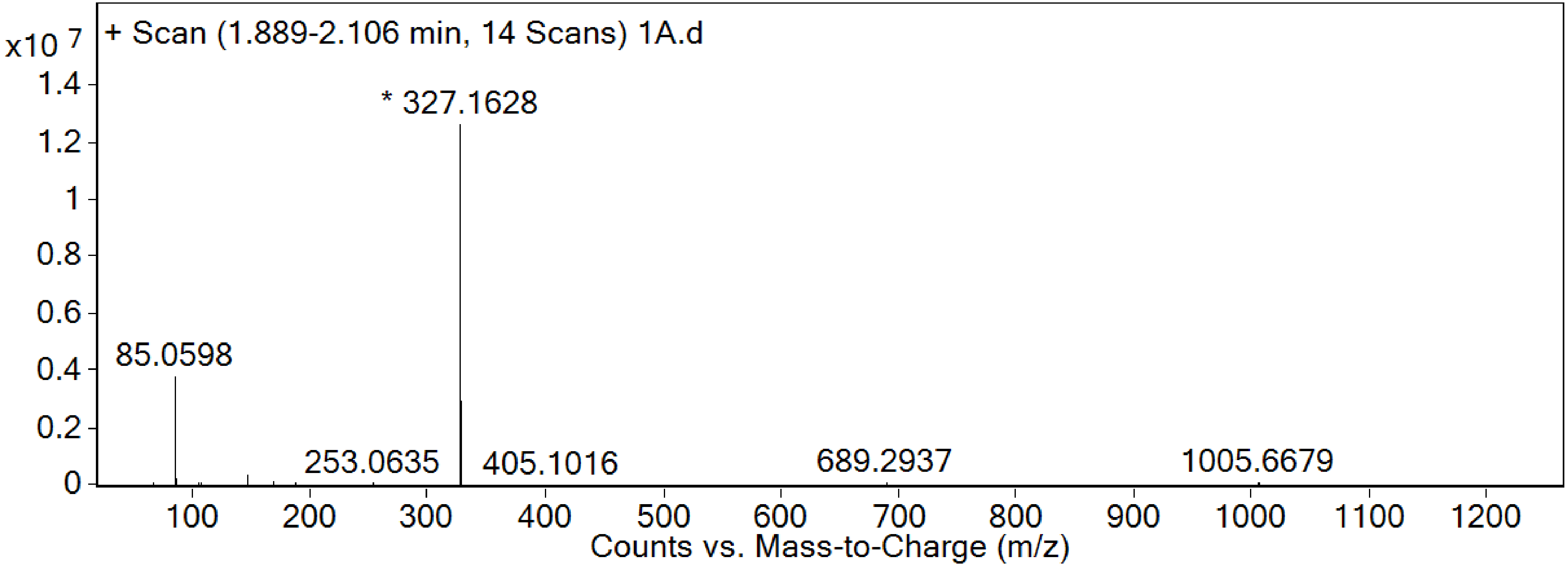

x10 7
+ Scan (1.889-2.106 min, 14 Scans) 1A.d
1.4
1.2
1
0.8
0.6
0.4
0.2
0
* 327.1628
85.0598
253.0635
405.1016
689.2937
1005.6679
100
200
300
400
500
600
700
800
900
1000
1100
1200
Counts vs. Mass-to-Charge (m/z)

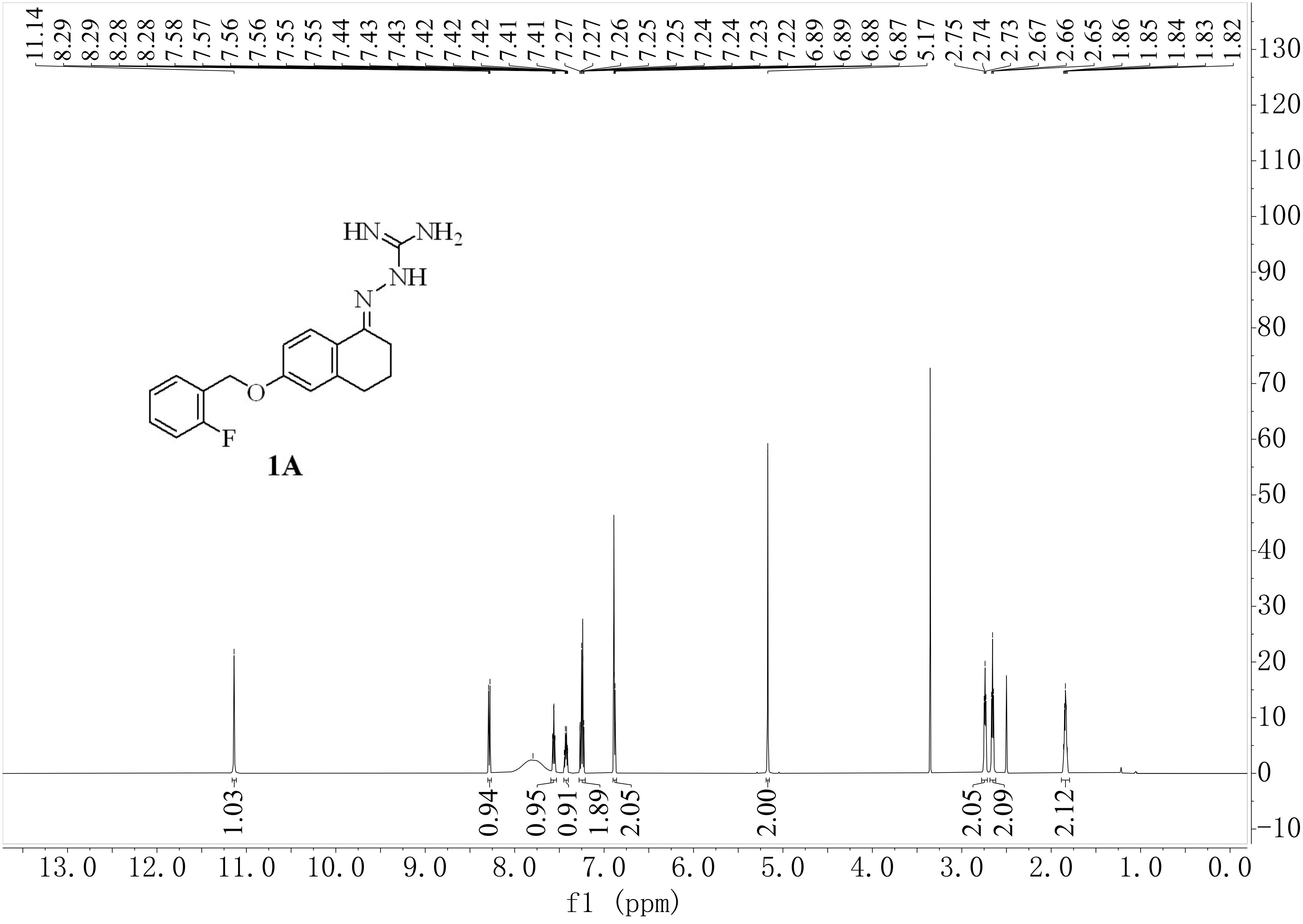

11.14
8.29
8.29
8.28
8.28
7.58
7.57
7.56
7.56
7.55
7.55
7.44
7.43
7.43
7.42
7.42
7.42
7.41
7.41
7.27
7.27
7.26
7.25
7.25
7.24
7.24
7.23
7.22
6.89
6.89
6.88
6.87
5.17
2.75
2.74
2.73
2.67
2.66
2.65
1.86
1.85
1.84
1.83
1.82
HN
NH2
NH
N
O
F
1A
1.03
0.94
0.95
0.91
1.89
2.05
2.00
2.05
2.09
2.12
13.0
12.0
11.0
10.0
9.0
8.0
7.0
6.0
5.0
4.0
3.0
2.0
1.0
0.0
f1 (ppm)
130
120
110
100
90
80
70
60
50
40
30
20
10
0
−10

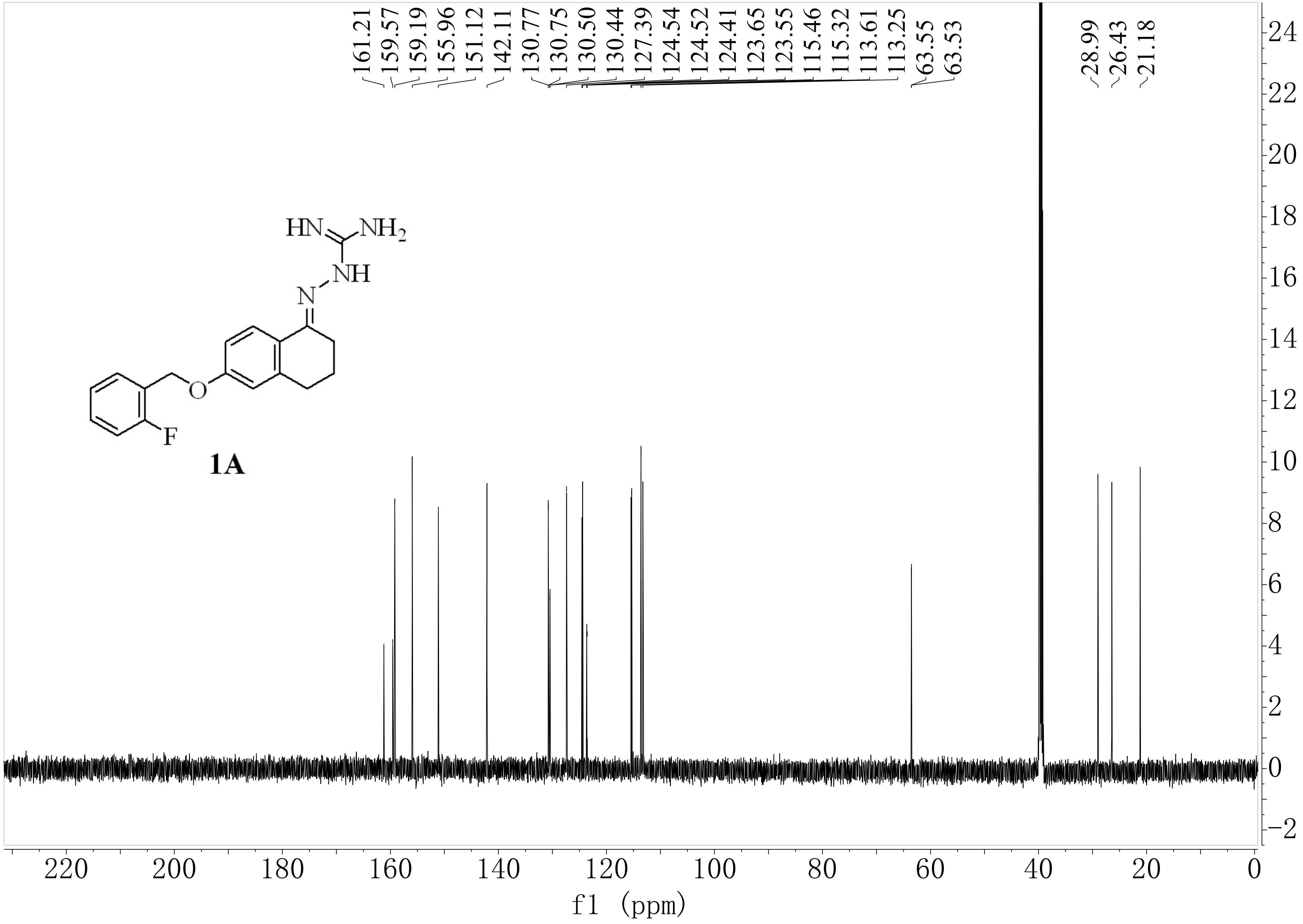

161.21
159.57
159.19
155.96
151.12
142.11
130.77
130.75
130.50
130.44
127.39
124.54
124.52
124.41
123.65
123.55
115.46
115.32
113.61
113.25
63.55
63.53
28.99
26.43
21.18
HN
NH2
NH
N
O
F
1A
220
200
180
160
140
120
100
80
60
40
20
0
f1 (ppm)
24
22
20
18
16
14
12
10
8
6
4
2
0
−2

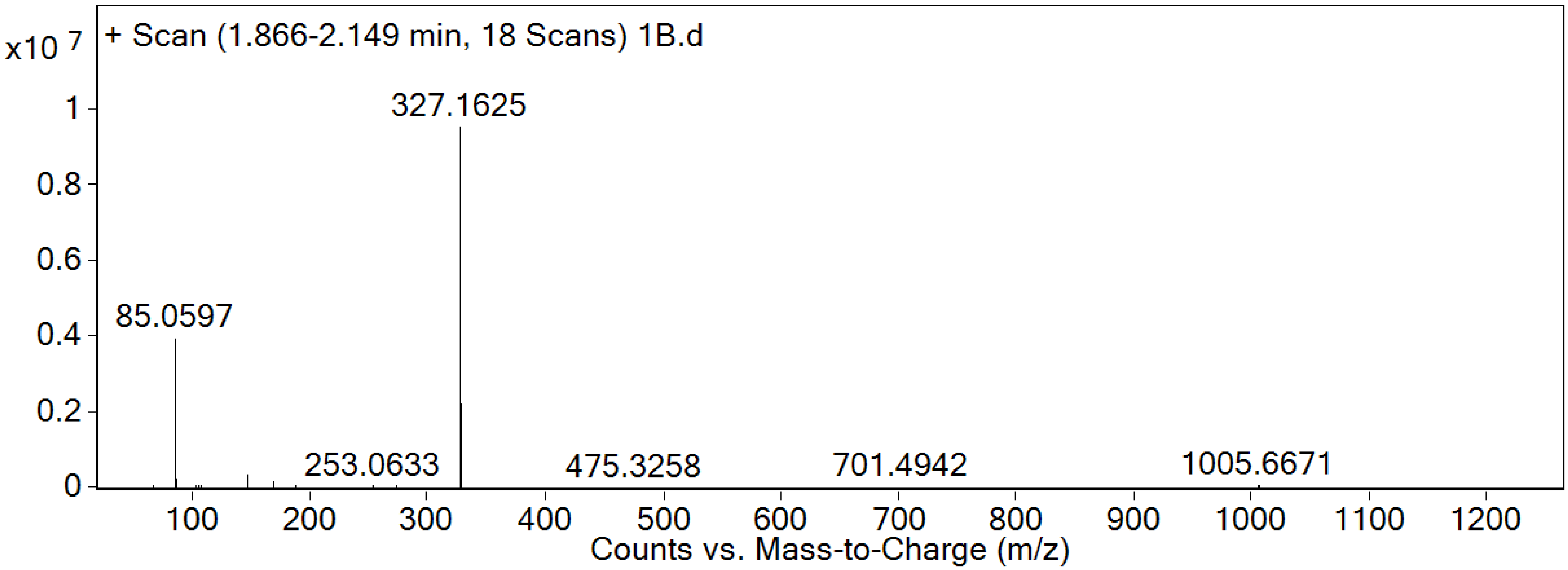

x10 7
+ Scan (1.866-2.149 min, 18 Scans) 1B.d
1
0.8
0.6
0.4
0.2
0
327.1625
85.0597
253.0633
475.3258
701.4942
1005.6671
100
200
300
400
500
600
700
800
900
1000
1100
1200
Counts vs. Mass-to-Charge (m/z)

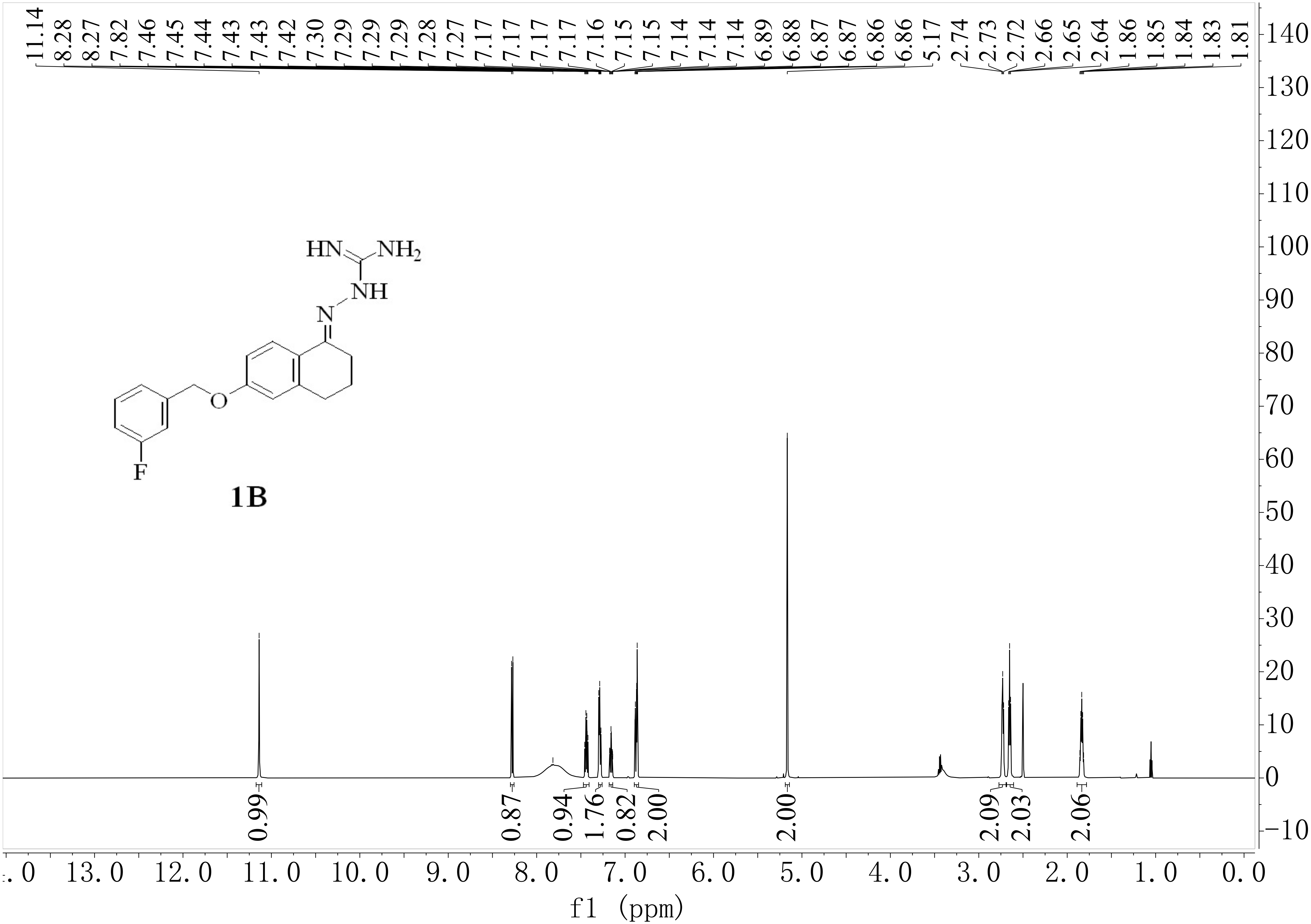

11.14
8.28
8.27
7.82
7.46
7.45
7.44
7.43
7.43
7.42
7.30
7.29
7.29
7.29
7.28
7.27
7.17
7.17
7.17
7.17
7.16
7.15
7.15
7.14
7.14
7.14
6.89
6.88
6.87
6.87
6.86
6.86
5.17
2.74
2.73
2.72
2.66
2.65
2.64
1.86
1.85
1.84
1.83
1.81
HN
NH2
NH
N
O
F
1B
0.99
0.87
0.94
1.76
0.82
2.00
2.00
2.09
2.03
2.06
13.0
12.0
11.0
10.0
9.0
8.0
7.0
6.0
5.0
4.0
3.0
2.0
1.0
0.0
f1 (ppm)

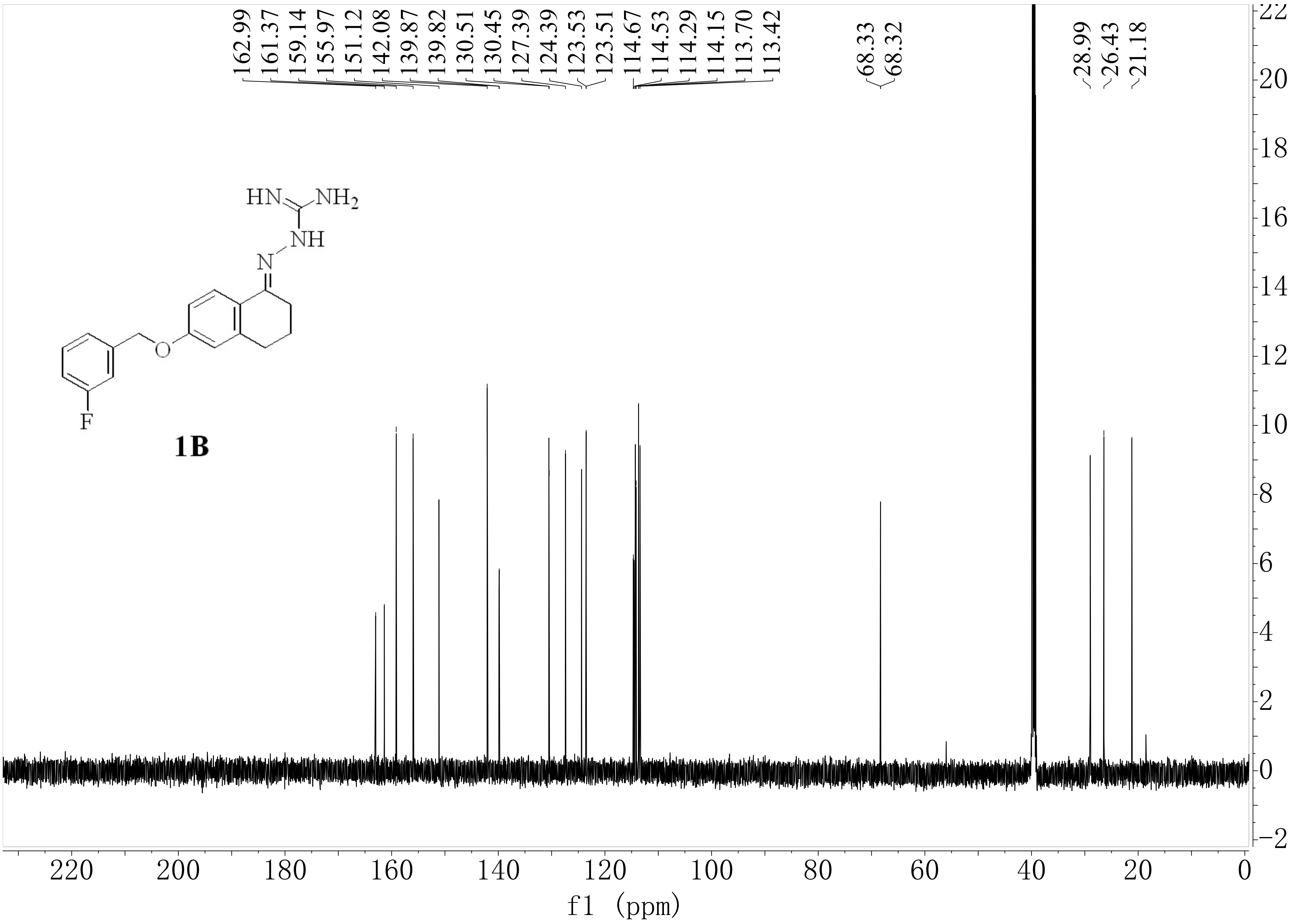

162.99
161.37
159.14
155.97
151.12
142.08
139.87
139.82
130.51
130.45
127.39
124.39
123.53
123.51
114.67
114.53
114.29
114.15
113.70
113.42
68.33
68.32
28.99
26.43
21.18
HN
NH2
NH
N
O
F
1B
220
200
180
160
140
120
100
80
60
40
20
0
f1 (ppm)
22
20
18
16
14
12
10
8
6
4
2
0
−2

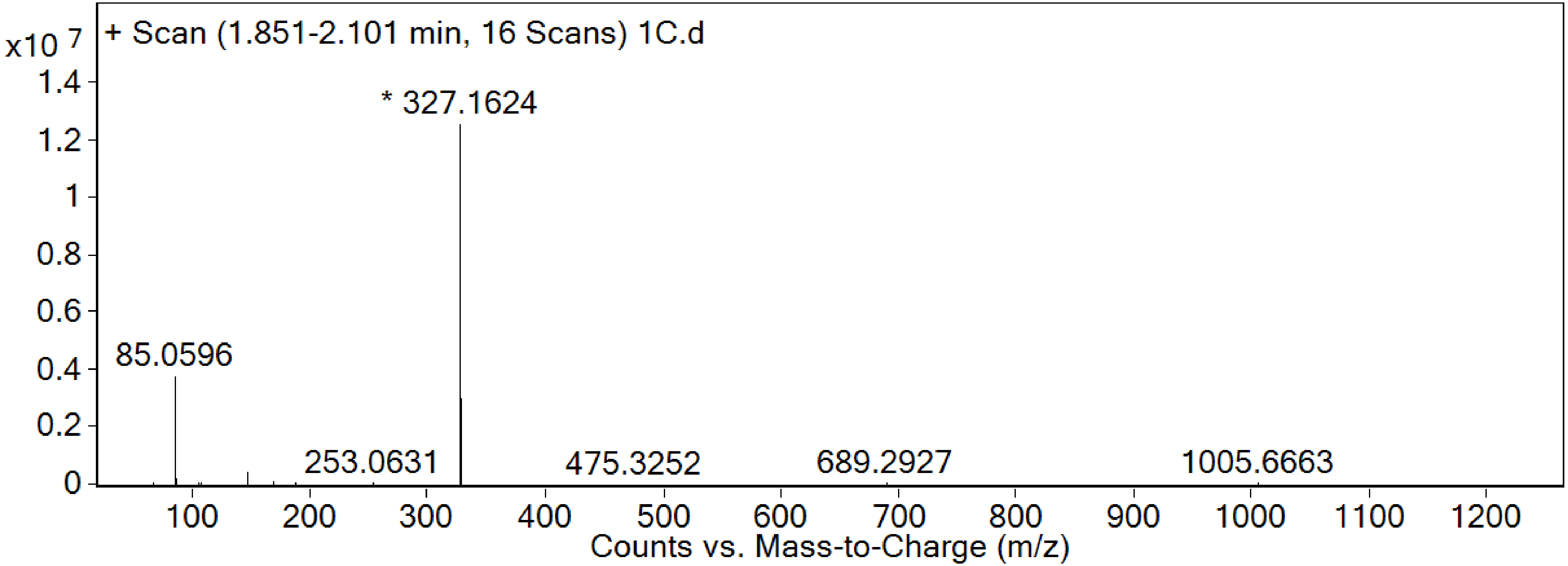

+ Scan (1.851-2.101 min, 16 Scans) 1C.d
x10 7
1.4
1.2
1
0.8
0.6
0.4
0.2
0
* 327.1624
85.0596
253.0631
475.3252
689.2927
1005.6663
100
200
300
400
500
600
700
800
900
1000
1100
1200
Counts vs. Mass-to-Charge (m/z)

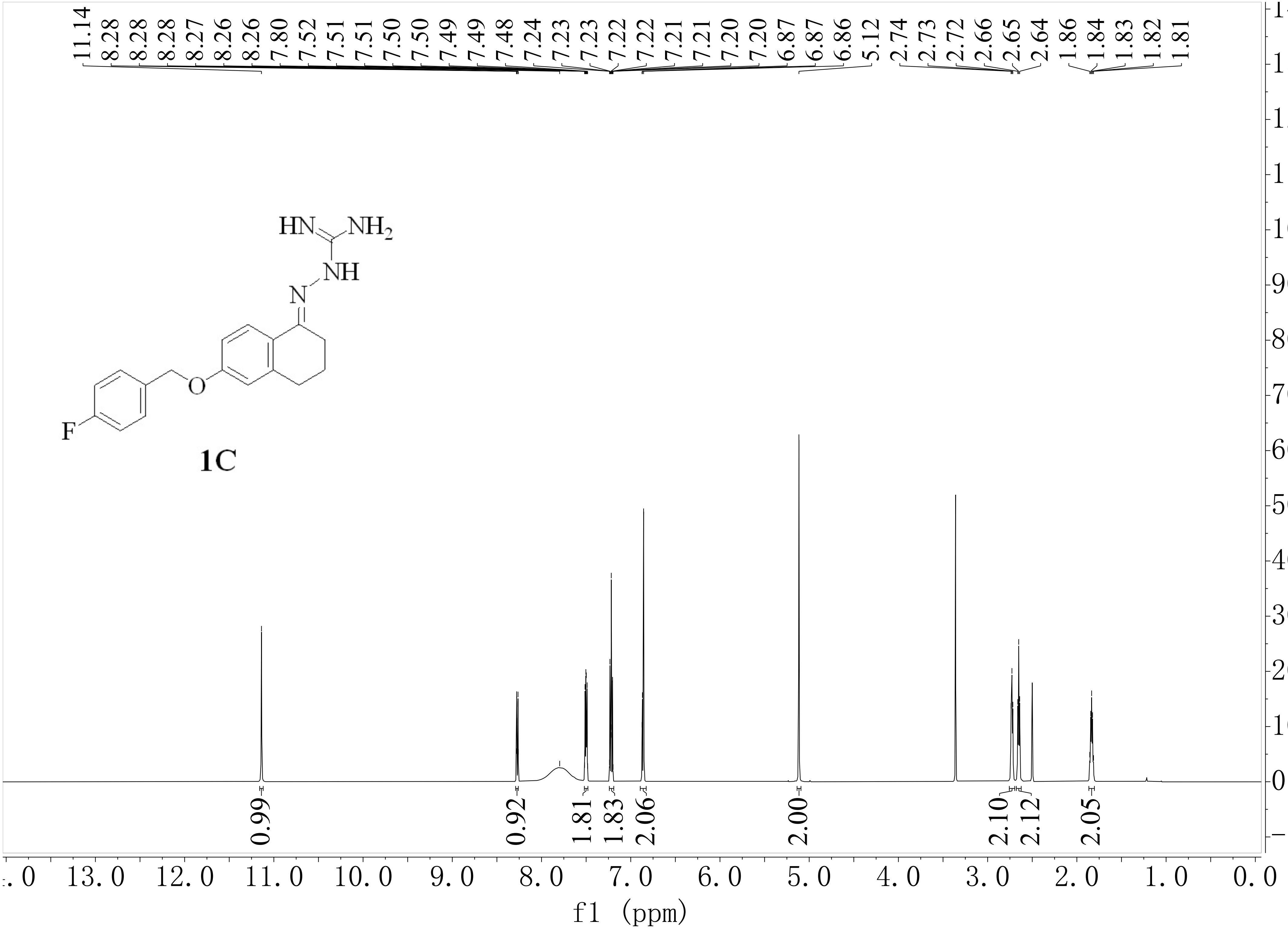
11.14
8.28
8.28
8.28
8.27
8.26
8.26
7.80
7.52
7.51
7.51
7.50
7.50
7.49
7.49
7.48
7.24
7.23
7.23
7.22
7.22
7.21
7.21
7.20
7.20
6.87
6.87
6.86
5.12
2.74
2.73
2.72
2.66
2.65
2.64
1.86
1.84
1.83
1.82
1.81
HN
NH2
NH
N
O
F
0.99
0.92
1.81
1.83
2.06
2.00
2.10
2.12
2.05
f1 (ppm)

1C

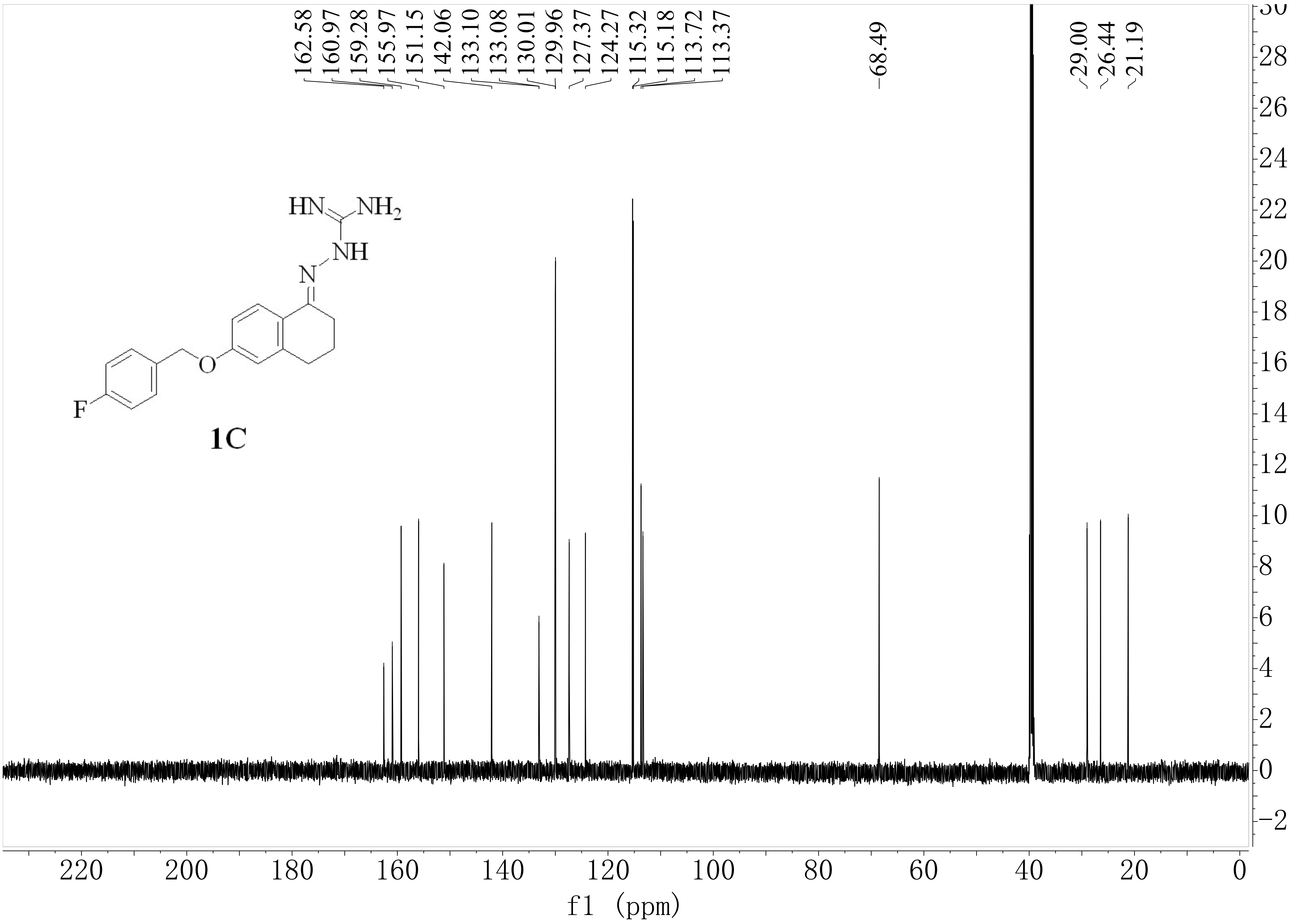
162.58
160.97
159.28
155.97
151.15
142.06
133.10
133.08
130.01
129.96
127.37
124.27
115.32
115.18
113.72
113.37
68.49
29.00
26.44
21.19
HN
NH2
NH
N
O
F
1C
f1 (ppm)

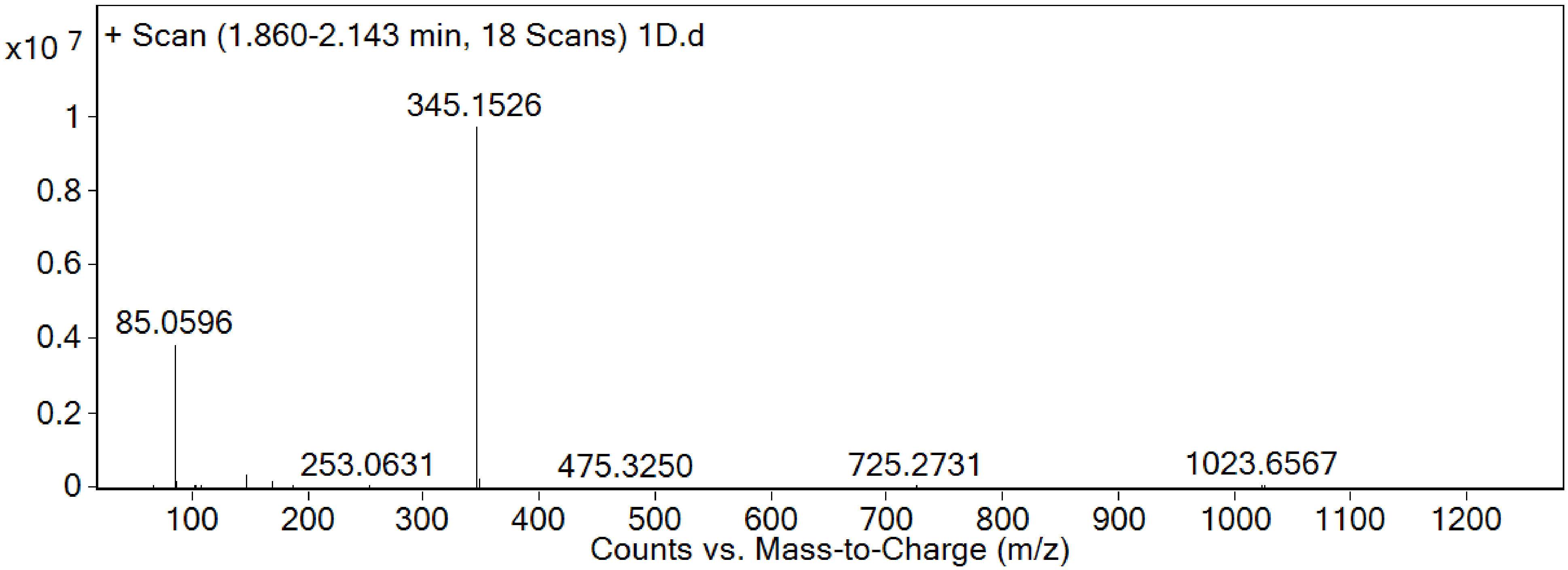

x10 7
+ Scan (1.860-2.143 min, 18 Scans) 1D.d
345.1526
85.0596
253.0631
475.3250
725.2731
1023.6567
1
0.8
0.6
0.4
0.2
0
100
200
300
400
500
600
700
800
900
1000
1100
1200
Counts vs. Mass-to-Charge (m/z)

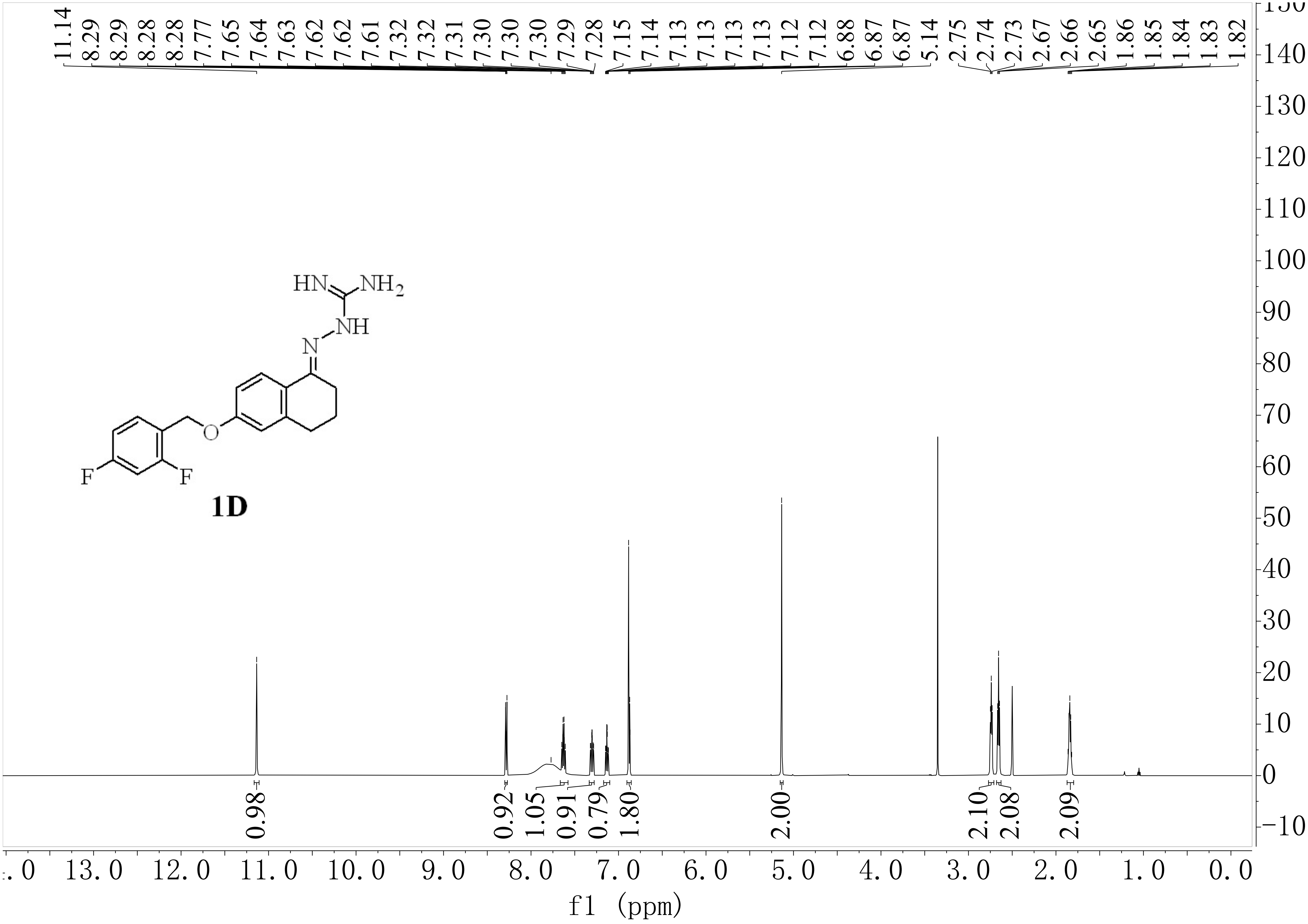
11.14 8.29 8.29 8.28 8.28 7.77 7.65 7.64 7.63 7.62 7.62 7.61 7.32 7.32 7.31 7.30 7.30 7.30 7.29 7.28 7.15 7.14 7.13 7.13 7.13 7.13 7.12 7.12 6.88 6.87 6.87 5.14 2.75 2.74 2.73 2.67 2.66 2.65 1.86 1.85 1.84 1.83 1.82
HN
NH2
NH
N
O
F
F
1D
0.98
0.92
1.05
0.91
0.79
1.80
2.00
2.10
2.08
2.09
f1 (ppm)

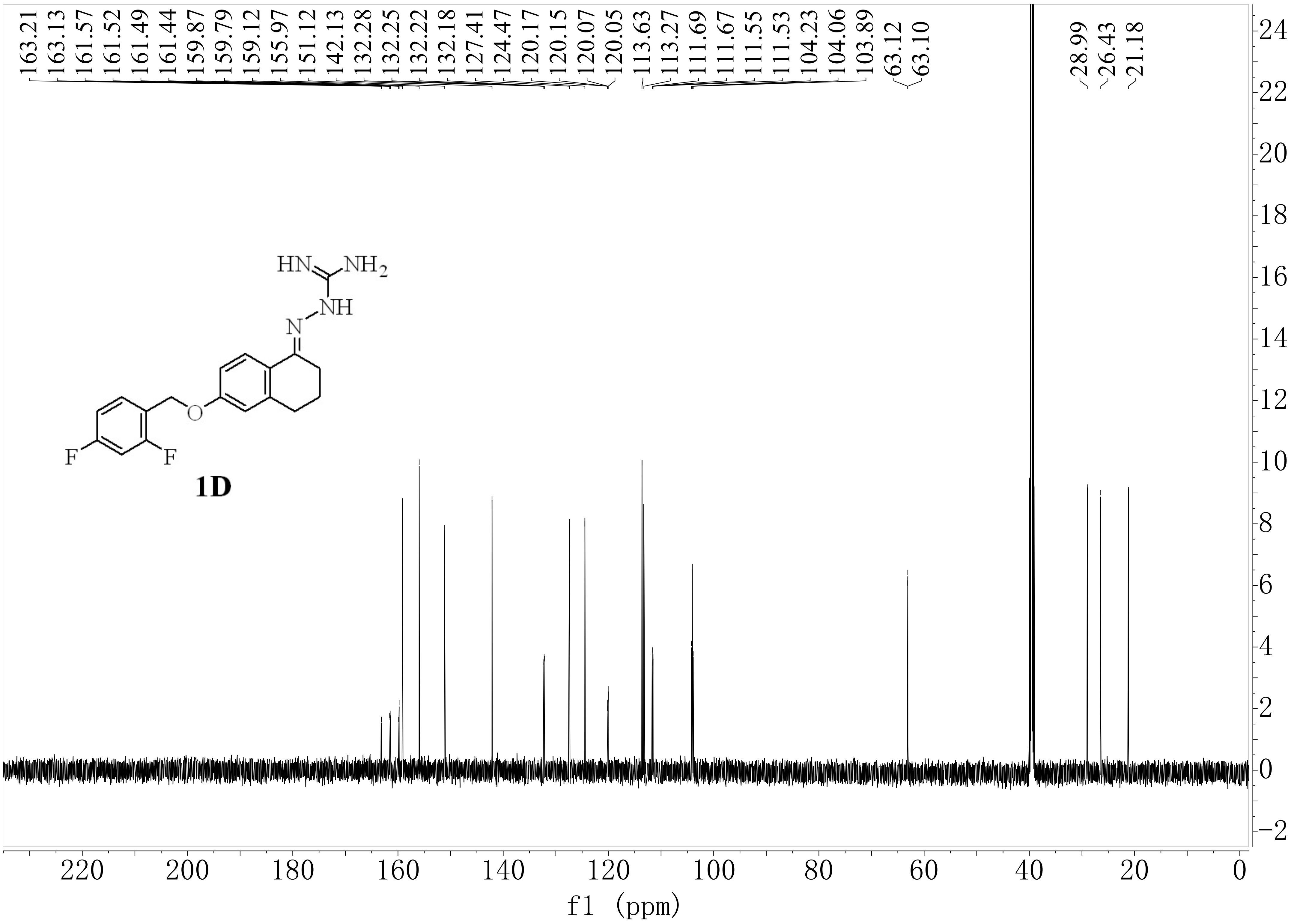
163.21
163.13
161.57
161.52
161.49
161.44
159.87
159.79
159.12
155.97
151.12
142.13
132.28
132.25
132.22
132.18
127.41
124.47
120.17
120.15
120.07
120.05
113.63
113.27
111.69
111.67
111.55
111.53
104.23
104.06
103.89
63.12
63.10
28.99
26.43
21.18
HN
NH2
NH
N
O
F
F
1D
220
200
180
160
140
120
100
80
60
40
20
0
f1 (ppm)
24
22
20
18
16
14
12
10
8
6
4
2
0
-2

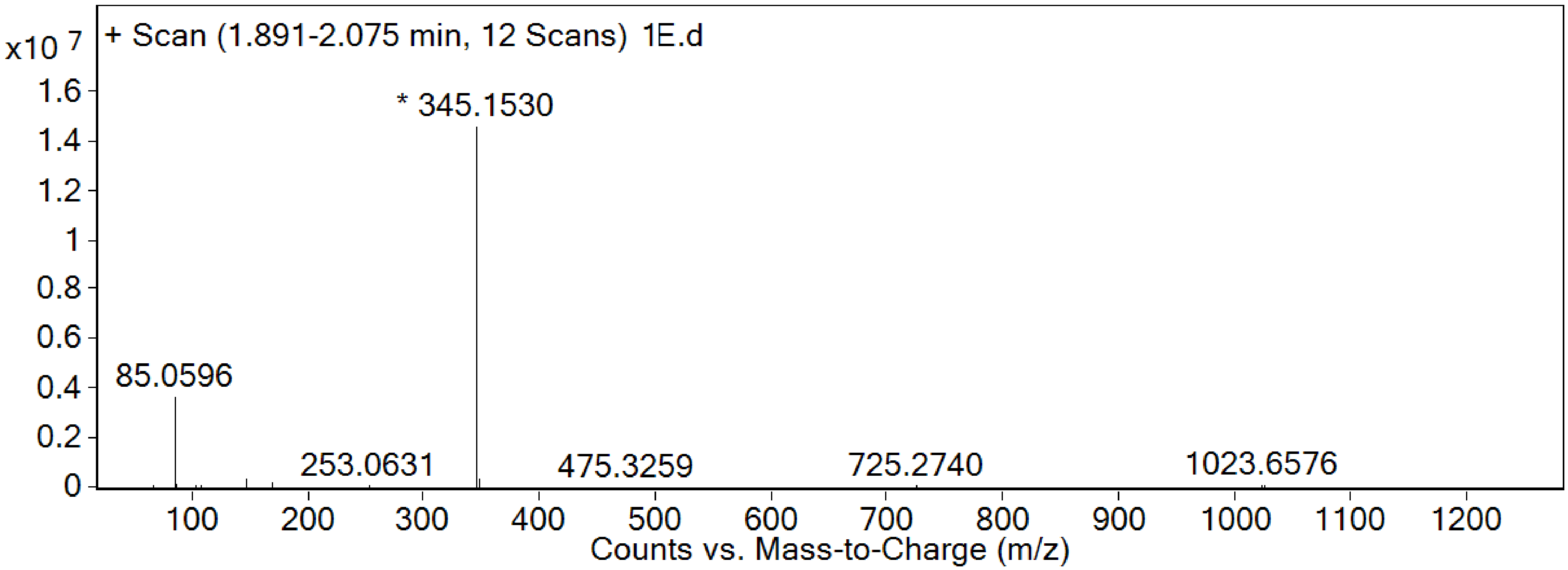

x10 7
+ Scan (1.891-2.075 min, 12 Scans) 1E.d
1.6
1.4
1.2
1
0.8
0.6
0.4
0.2
0
* 345.1530
85.0596
253.0631
475.3259
725.2740
1023.6576
100
200
300
400
500
600
700
800
900
1000
1100
1200
Counts vs. Mass-to-Charge (m/z)

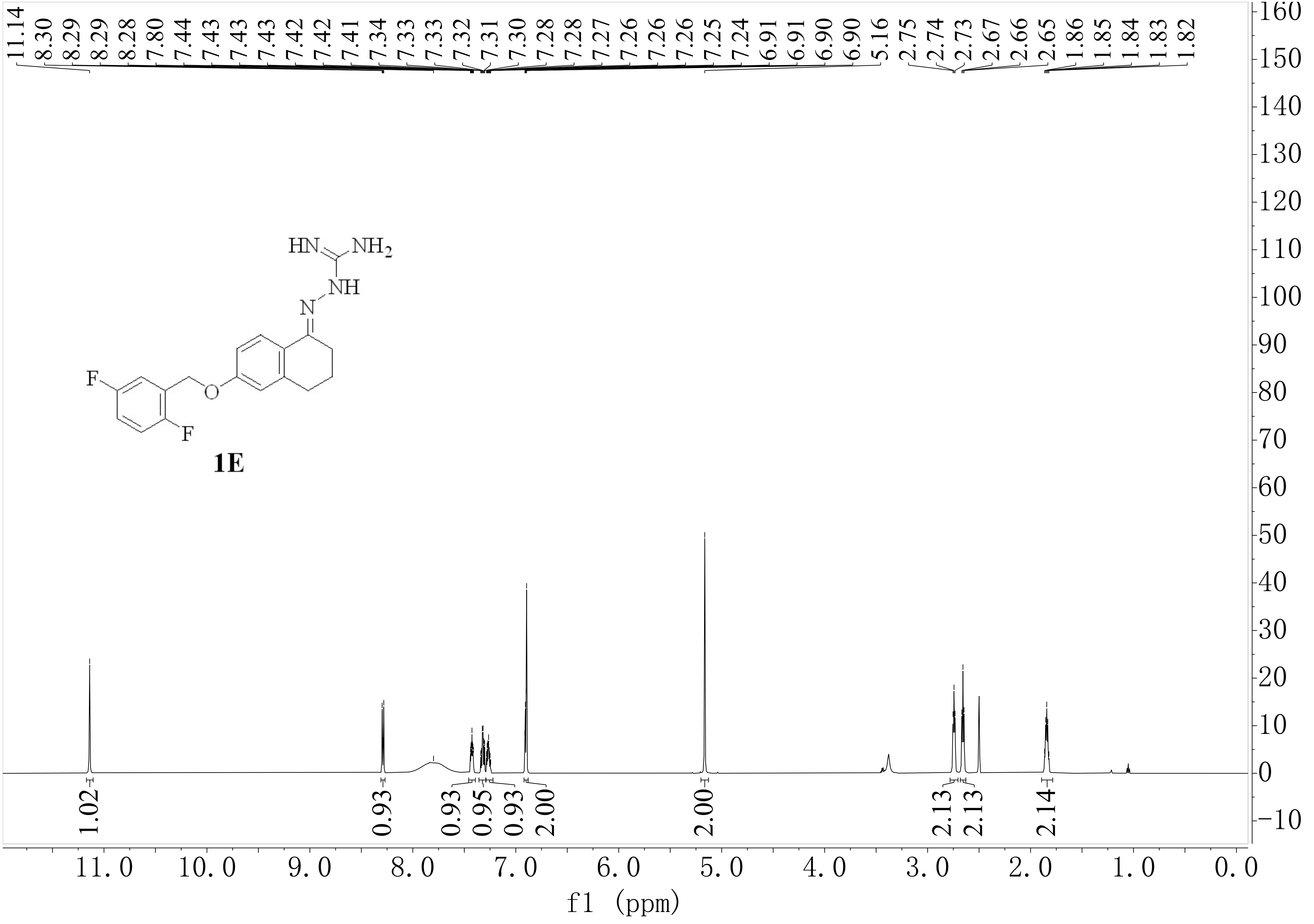

11.14
8.30
8.29
8.29
8.28
7.80
7.44
7.43
7.43
7.43
7.42
7.42
7.41
7.34
7.33
7.33
7.32
7.31
7.30
7.28
7.28
7.27
7.26
7.26
7.26
7.25
7.24
6.91
6.91
6.90
6.90
5.16
2.75
2.74
2.73
2.67
2.66
2.65
1.86
1.85
1.84
1.83
1.82
HN
NH2
NH
N
O
F
F
1E
1.02
0.93
0.93
0.95
0.93
2.00
2.00
2.13
2.13
2.14
11.0
10.0
9.0
8.0
7.0
6.0
5.0
4.0
3.0
2.0
1.0
0.0
f1 (ppm)
160
150
140
130
120
110
100
90
80
70
60
50
40
30
20
10
0
−10

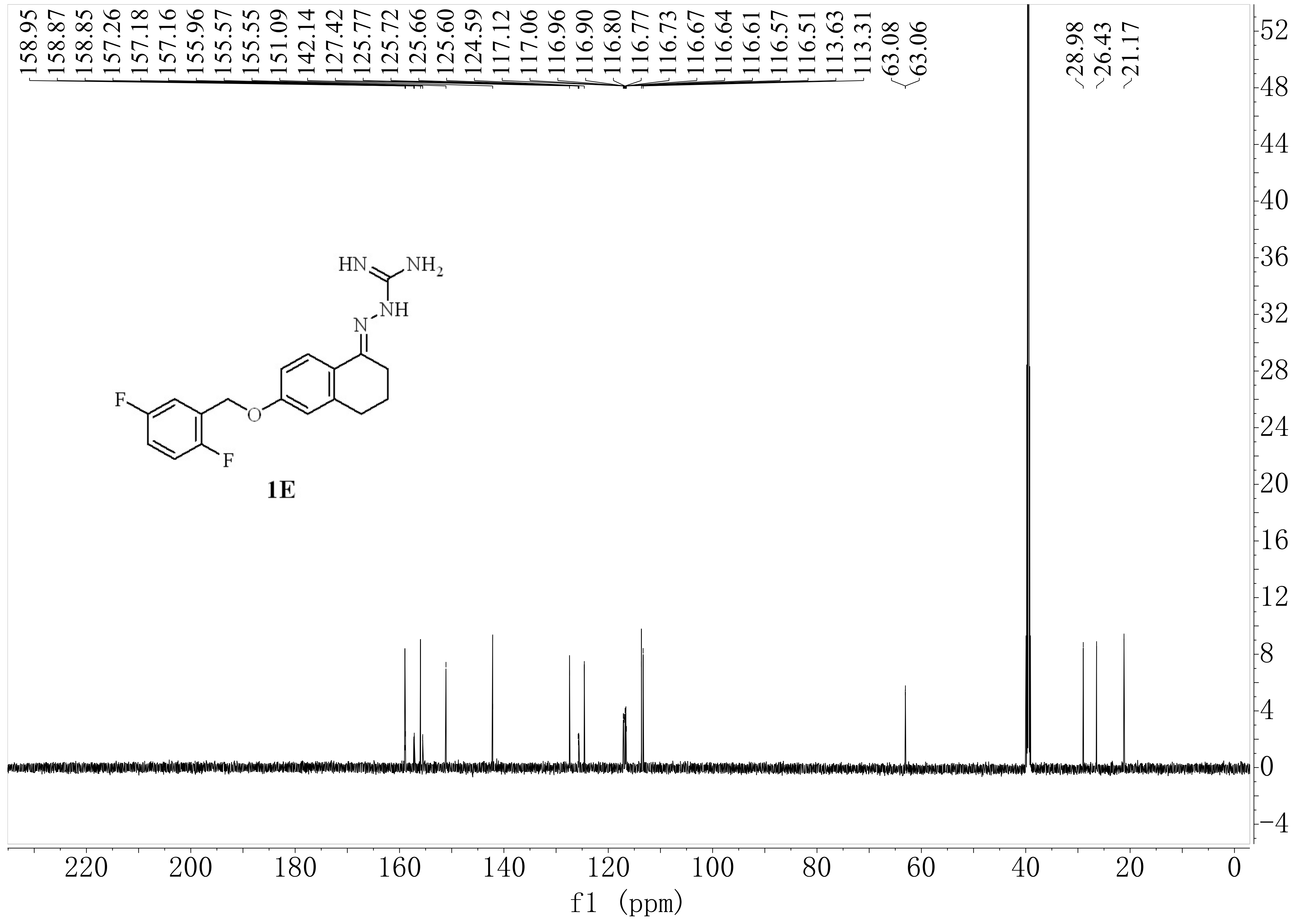

158.95
158.87
158.85
157.26
157.18
157.16
155.96
155.57
155.55
151.09
142.14
127.42
125.77
125.72
125.66
125.60
124.59
117.12
117.06
116.96
116.90
116.80
116.77
116.73
116.67
116.64
116.61
116.57
116.51
113.63
113.31
63.08
63.06
28.98
26.43
21.17
HN
NH2
NH
N
F
O
F
1E
220
200
180
160
140
120
100
80
60
40
20
0
f1 (ppm)
52
48
44
40
36
32
28
24
20
16
12
8
4
0
−4

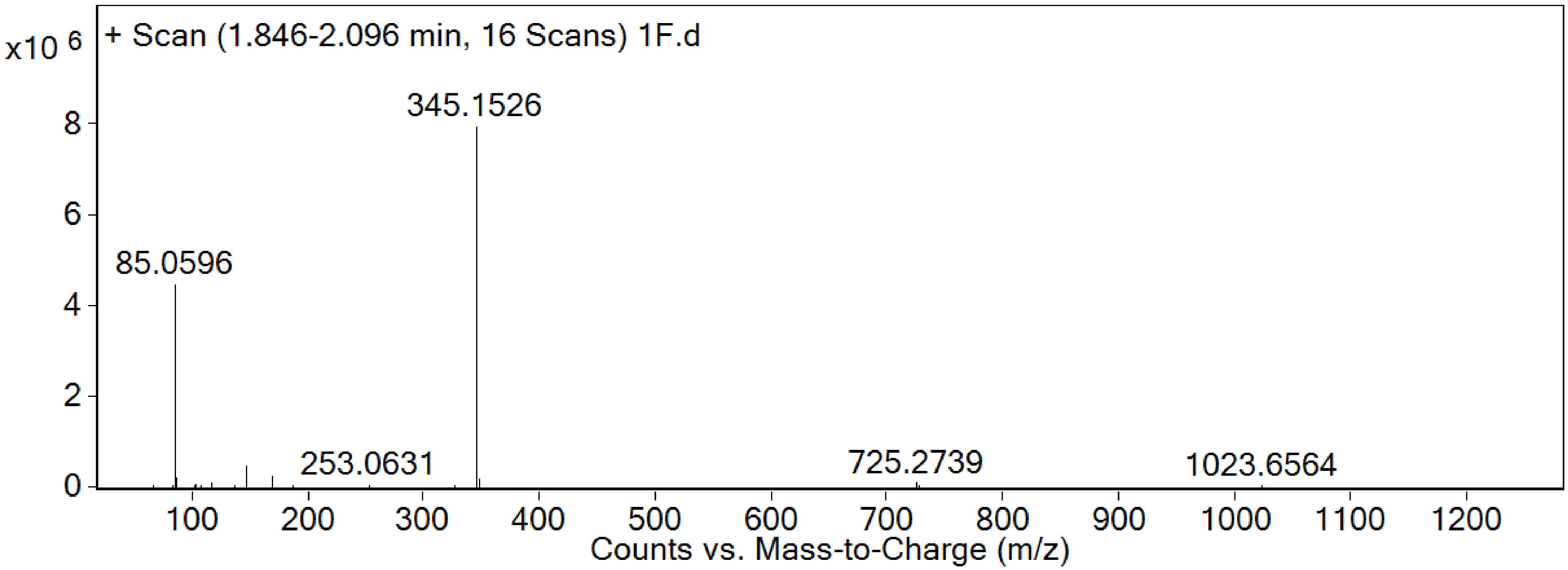
x10 6
+ Scan (1.846-2.096 min, 16 Scans) 1F.d
345.1526
85.0596
253.0631
725.2739
1023.6564
0
2
4
6
8
100
200
300
400
500
600
700
800
900
1000
1100
1200
Counts vs. Mass-to-Charge (m/z)

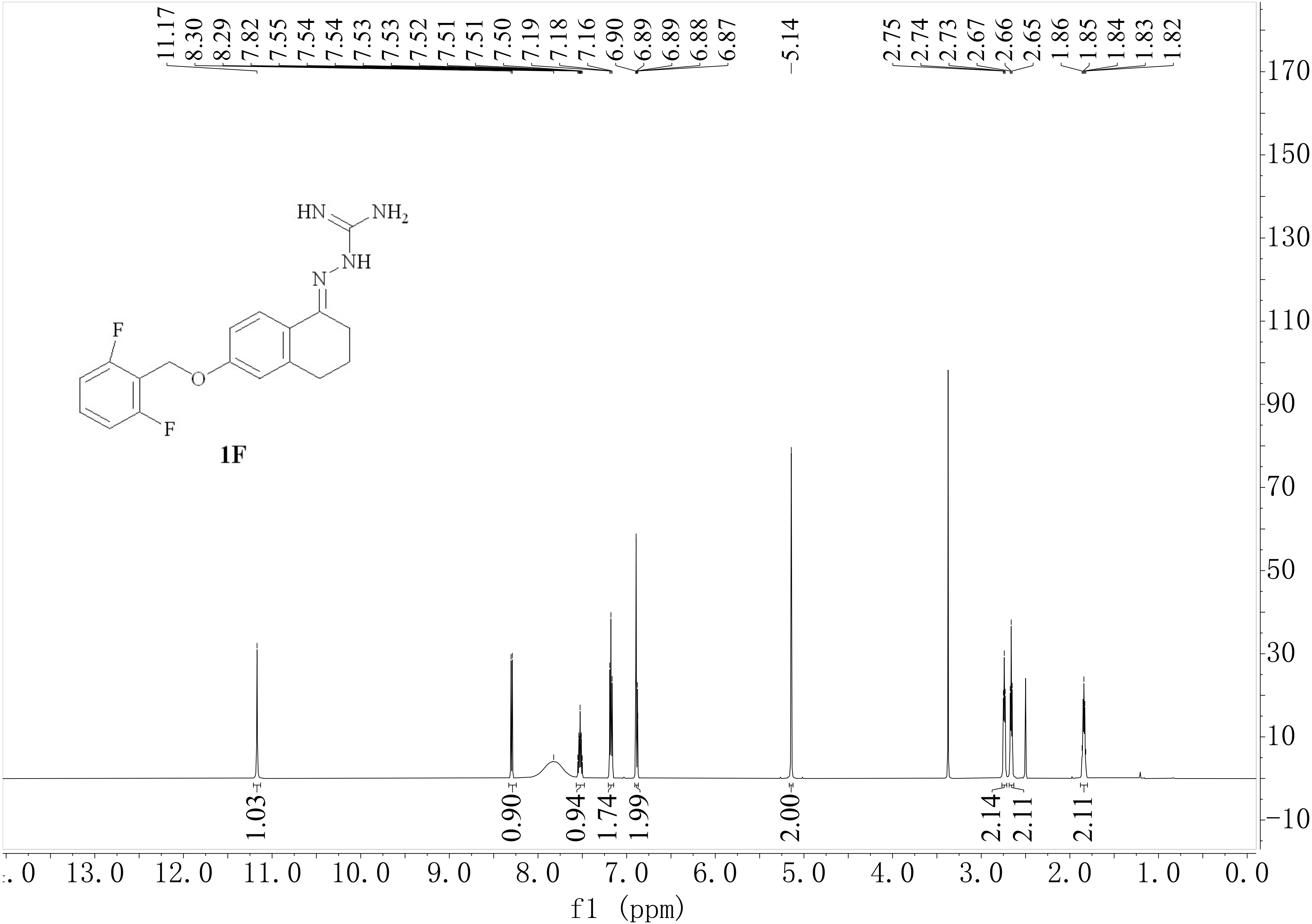
11.17 8.30 8.29 7.82 7.55 7.54 7.54 7.53 7.53 7.52 7.51 7.51 7.50 7.19 7.18 7.16 6.90 6.89 6.89 6.88 6.87
5.14
2.75 2.74 2.73 2.67 2.66 2.65 1.86 1.85 1.84 1.83 1.82
HN
NH2
NH
N
F
O
F
1F
1.03
0.90
0.94
1.74
1.99
2.00
2.14
2.11
2.11
f1 (ppm)

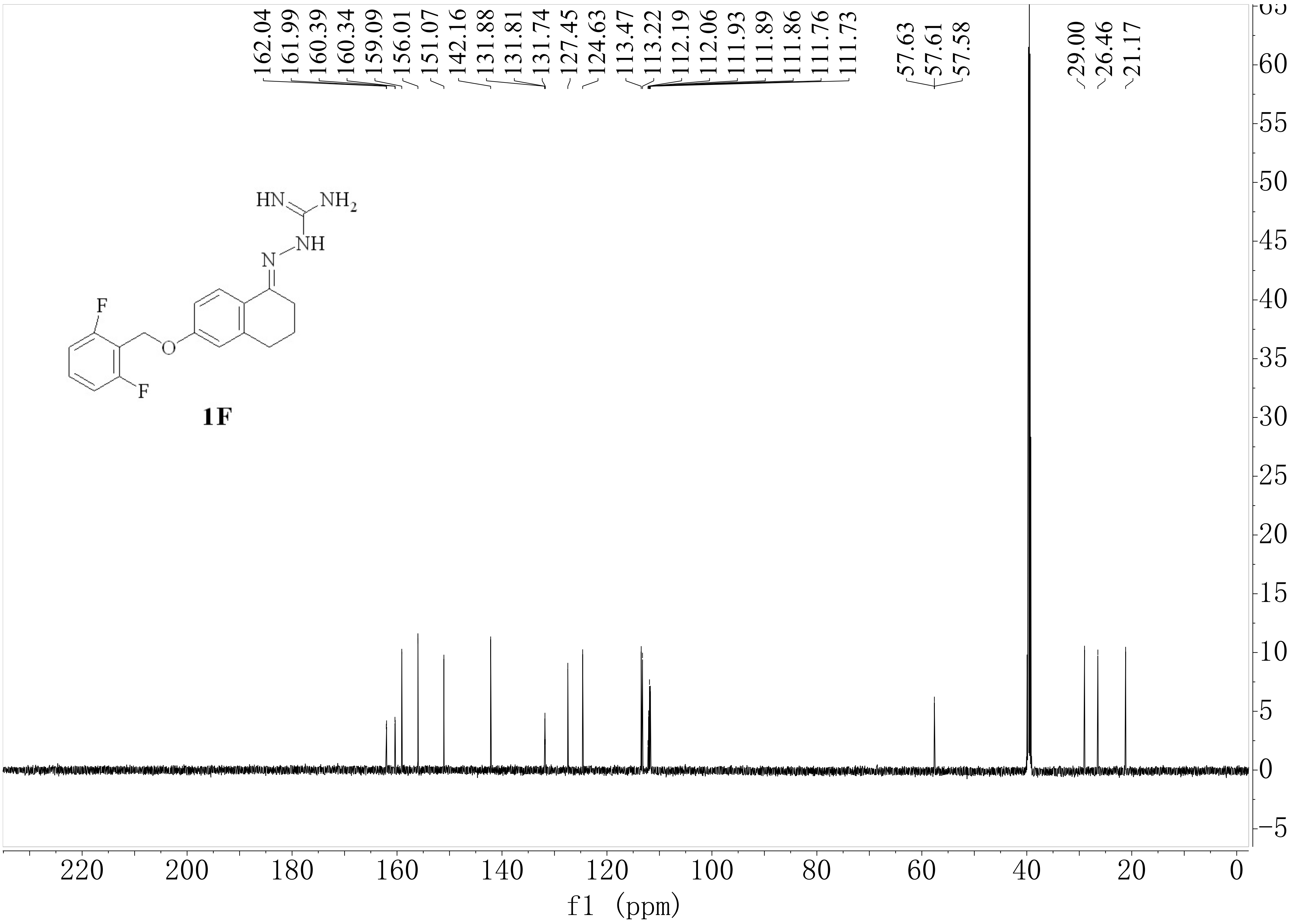
162.04
161.99
160.39
160.34
159.09
156.01
151.07
142.16
131.88
131.81
131.74
127.45
124.63
113.47
113.22
112.19
112.06
111.93
111.89
111.86
111.76
111.73
57.63
57.61
57.58
29.00
26.46
21.17
HN
NH2
NH
N
F
O
F
1F
220 200 180 160 140 120 100 80 60 40 20 0
f1 (ppm)
60 55 50 45 40 35 30 25 20 15 10 5 0 −5

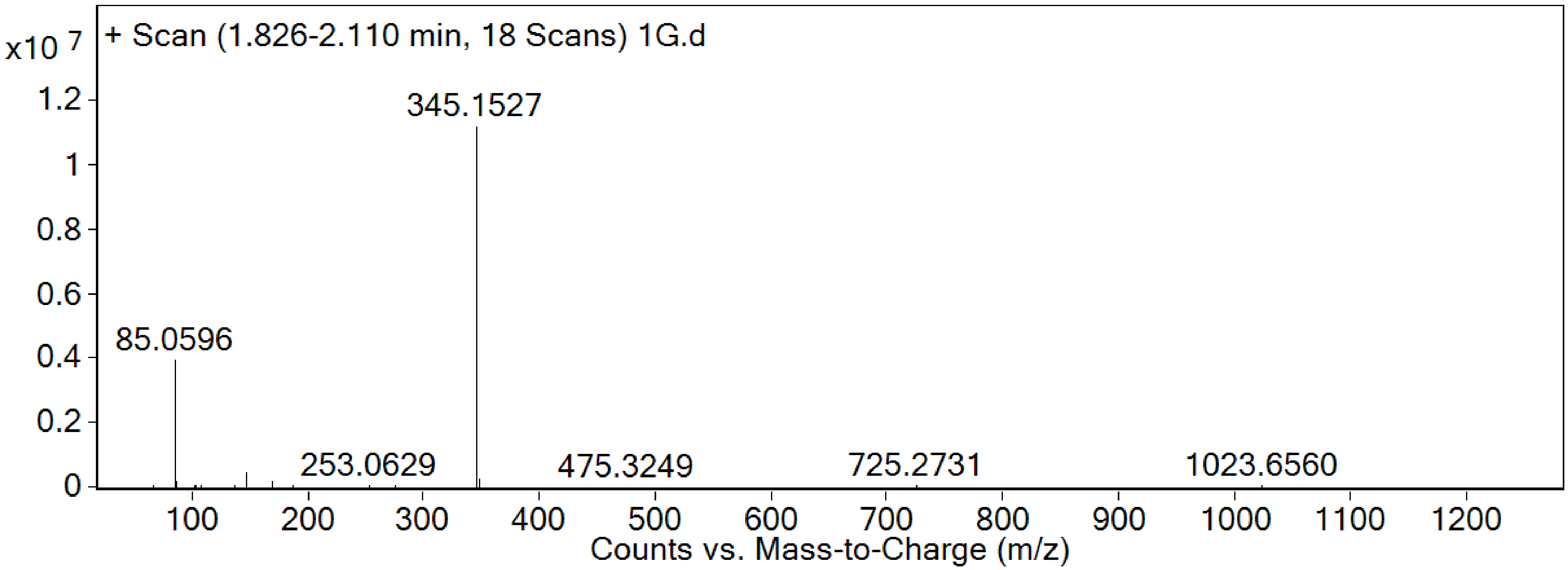
x10 7
+ Scan (1.826-2.110 min, 18 Scans) 1G.d
1.2
1
0.8
0.6
0.4
0.2
0
345.1527
85.0596
253.0629
475.3249
725.2731
1023.6560
100
200
300
400
500
600
700
800
900
1000
1100
1200
Counts vs. Mass-to-Charge (m/z)

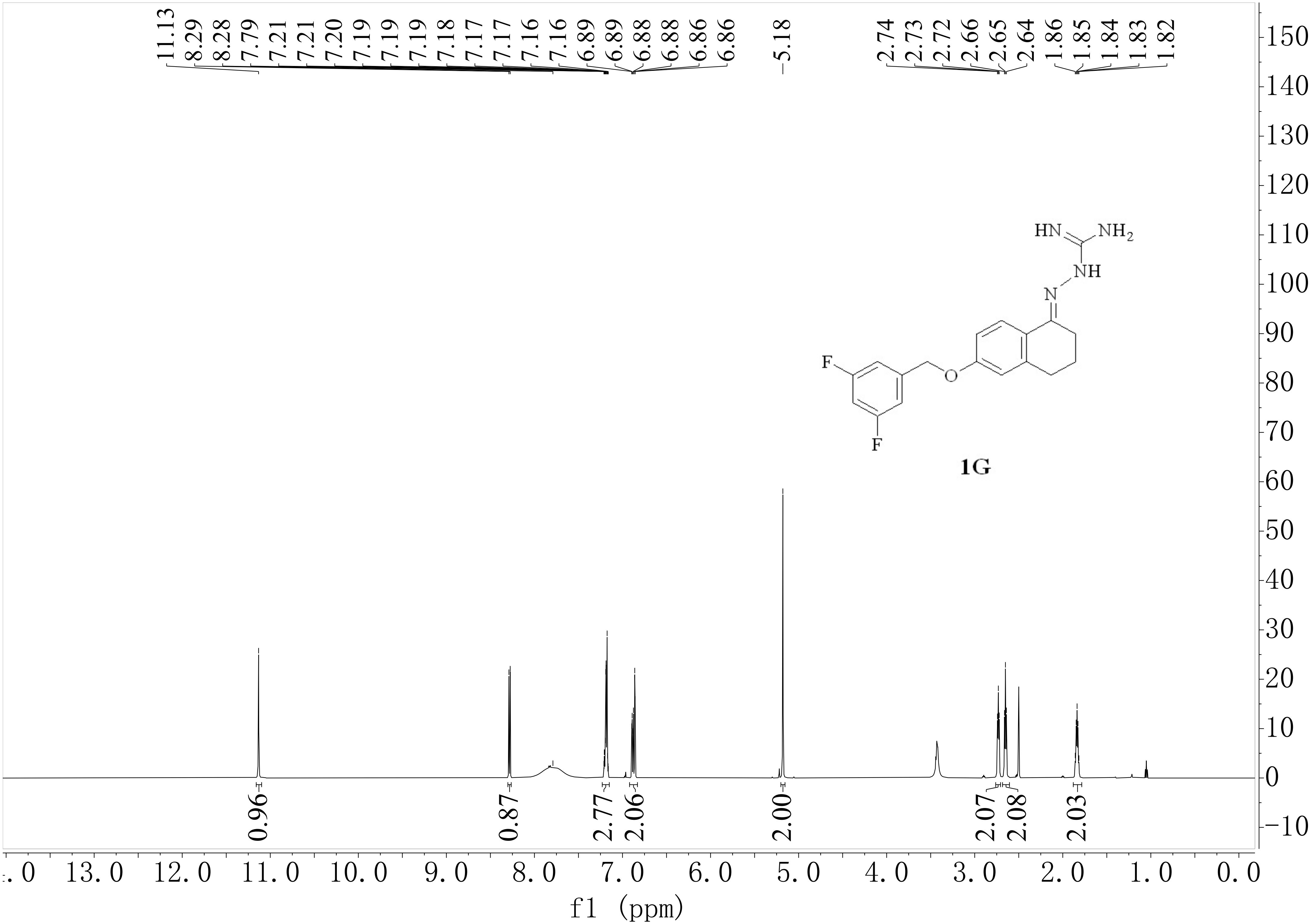
11.13
8.29
8.28
7.79
7.21
7.21
7.20
7.19
7.19
7.19
7.18
7.17
7.17
7.16
7.16
6.89
6.89
6.88
6.88
6.86
6.86
5.18
2.74
2.73
2.72
2.66
2.65
2.64
1.86
1.85
1.84
1.83
1.82
HN
NH2
NH
N
F
O
F
0.96
0.87
2.77
2.06
2.00
2.07
2.08
2.03
f1 (ppm)

1G

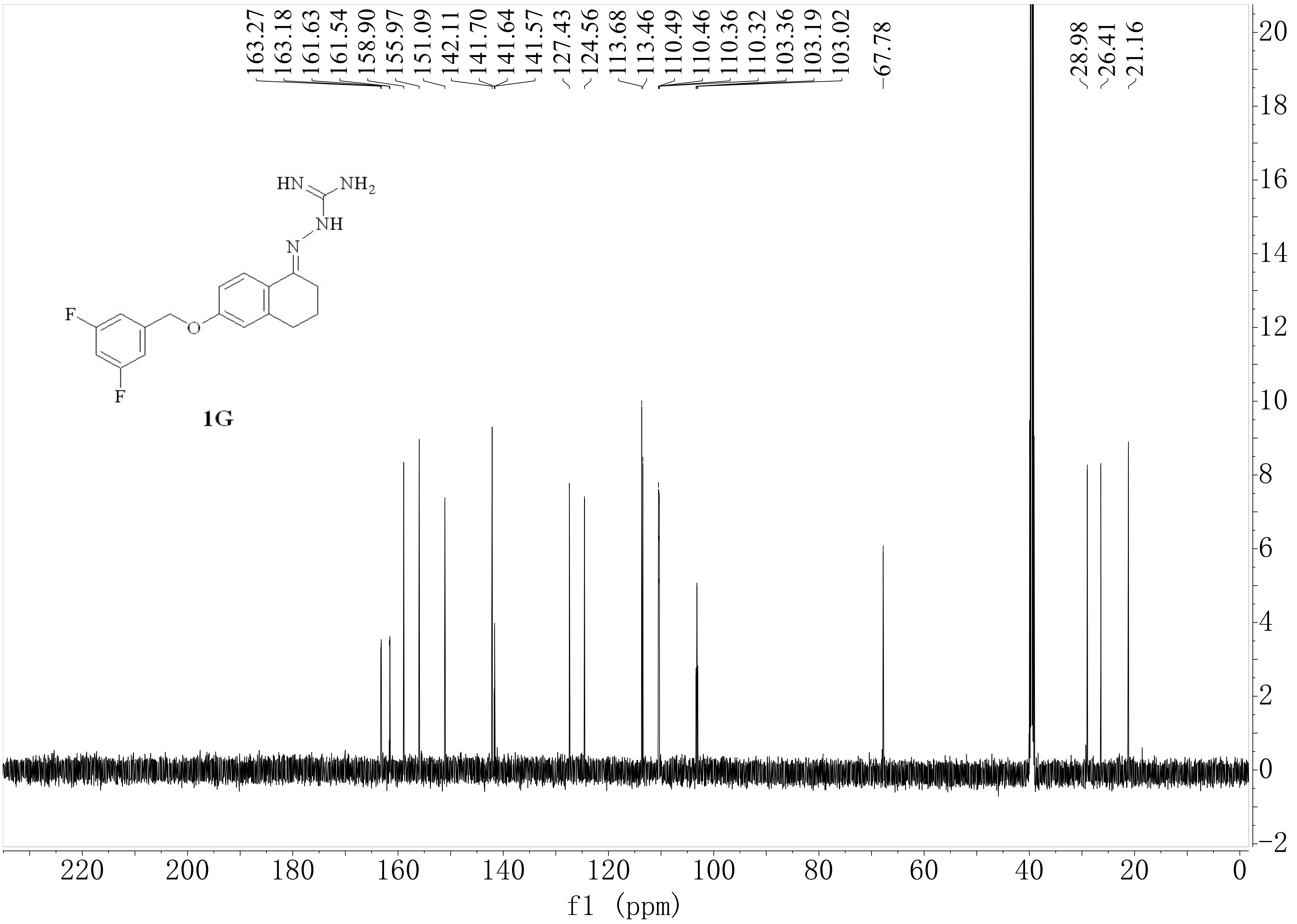
163.27
163.18
161.63
161.54
158.90
155.97
151.09
142.11
141.70
141.64
141.57
127.43
124.56
113.68
113.46
110.49
110.46
110.36
110.32
103.36
103.19
103.02
67.78
28.98
26.41
21.16
HN
NH_2
NH
N
O
F
F
1G
220
200
180
160
140
120
100
80
60
40
20
0
f1 (ppm)
20
18
16
14
12
10
8
6
4
2
0
−2

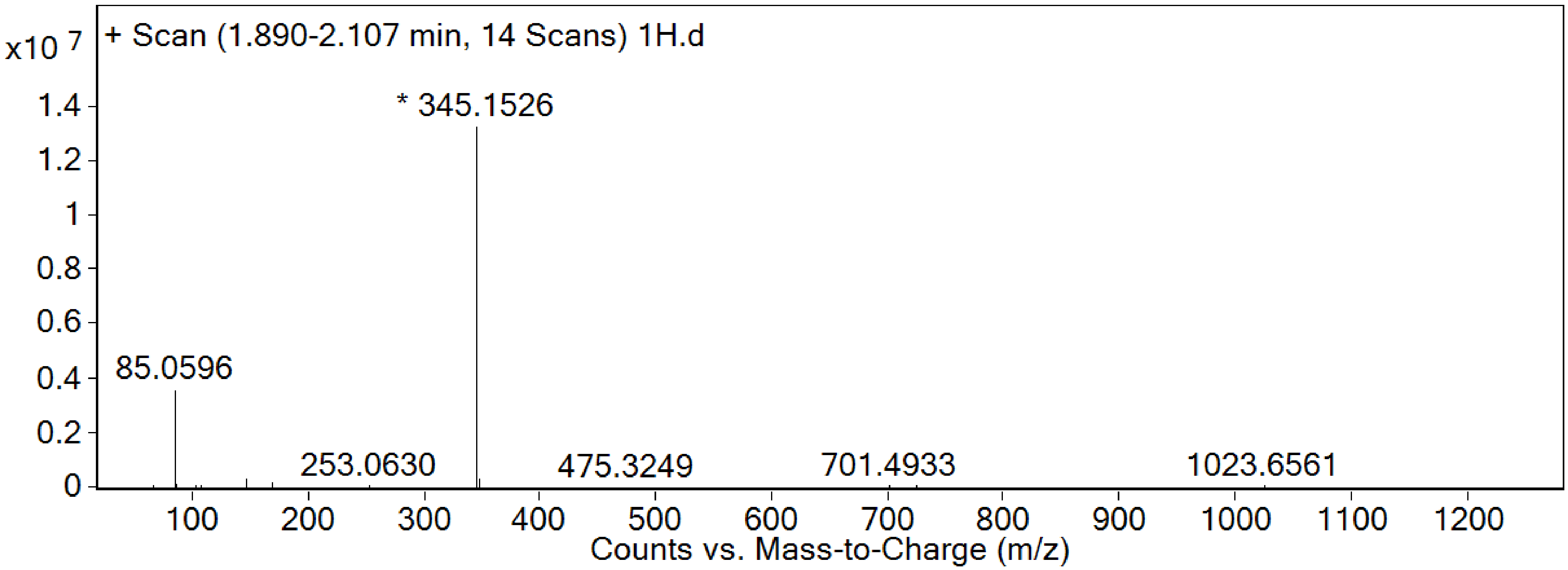

x10 7
+ Scan (1.890-2.107 min, 14 Scans) 1H.d
1.4
1.2
1
0.8
0.6
0.4
0.2
0
* 345.1526
85.0596
253.0630
475.3249
701.4933
1023.6561
100
200
300
400
500
600
700
800
900
1000
1100
1200
Counts vs. Mass-to-Charge (m/z)

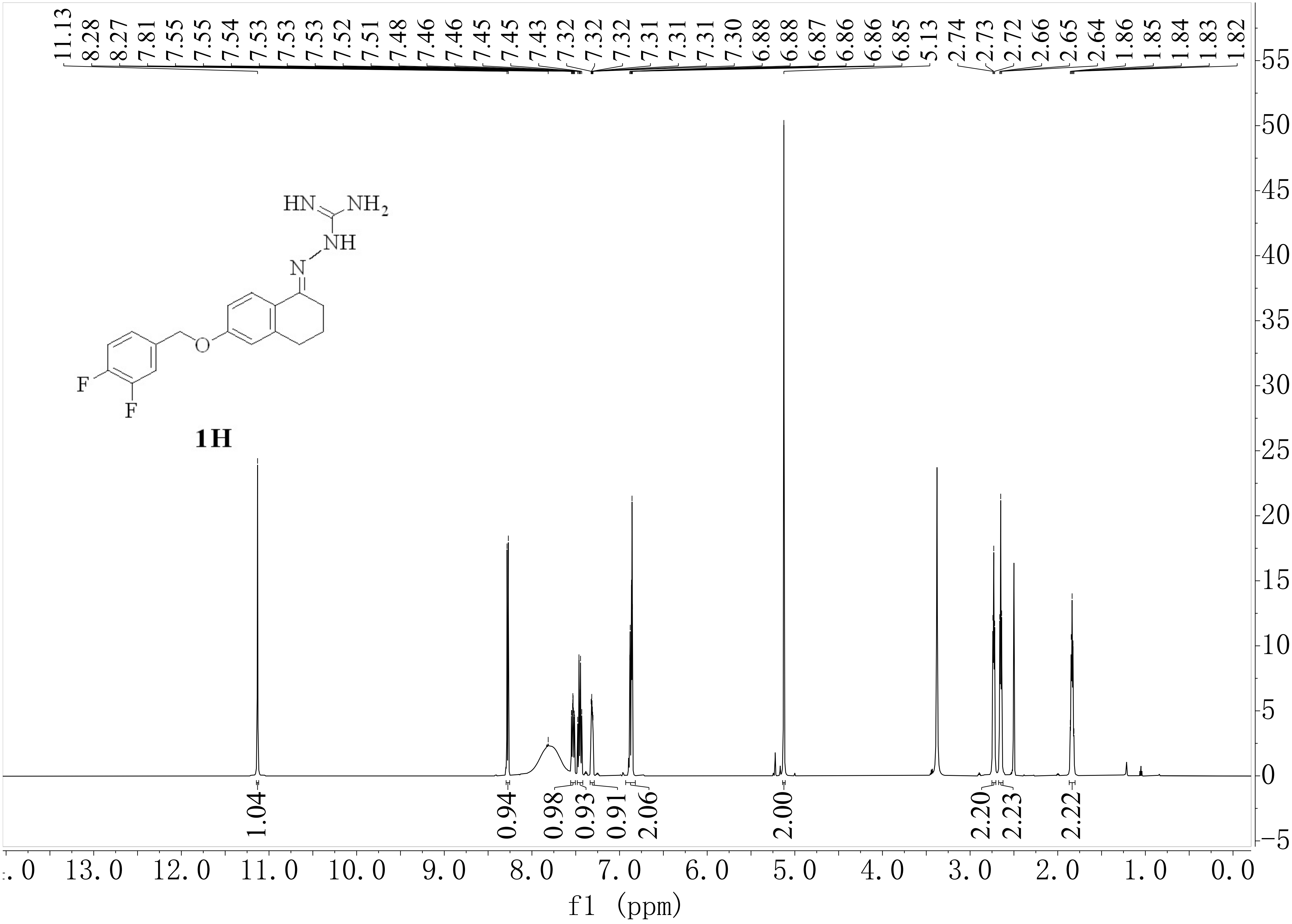

11.13 8.28 8.27 7.81 7.55 7.55 7.54 7.53 7.53 7.53 7.52 7.51 7.48 7.46 7.46 7.45 7.45 7.43 7.32 7.32 7.32 7.31 7.31 7.31 7.30 6.88 6.88 6.87 6.86 6.86 6.85 5.13 2.74 2.73 2.72 2.66 2.65 2.64 1.86 1.85 1.84 1.83 1.82
HN NH2
NH
N
O
F
F
1H
1.04
0.94
0.98
0.93
0.91
2.06
2.00
2.20
2.23
2.22
13.0 12.0 11.0 10.0 9.0 8.0 7.0 6.0 5.0 4.0 3.0 2.0 1.0 0.0
f1 (ppm)
55 50 45 40 35 30 25 20 15 10 5 0 −5

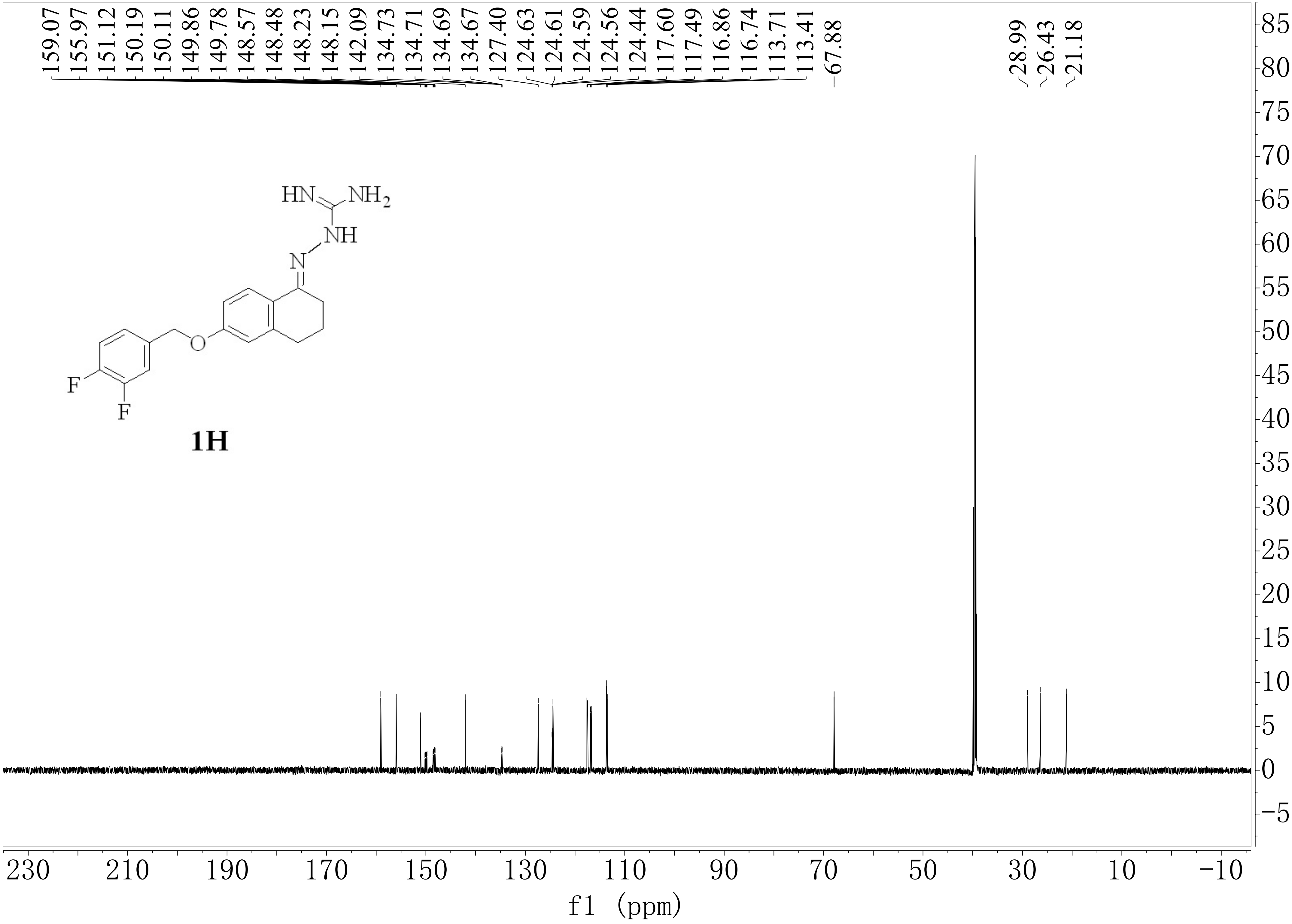
159.07
155.97
151.12
150.19
150.11
149.86
149.78
148.57
148.48
148.23
148.15
142.09
134.73
134.71
134.69
134.67
127.40
124.63
124.61
124.59
124.56
124.44
117.60
117.49
116.86
116.74
113.71
113.41
67.88
28.99
26.43
21.18
HN
NH2
NH
N
O
F
F
1H
f1 (ppm)

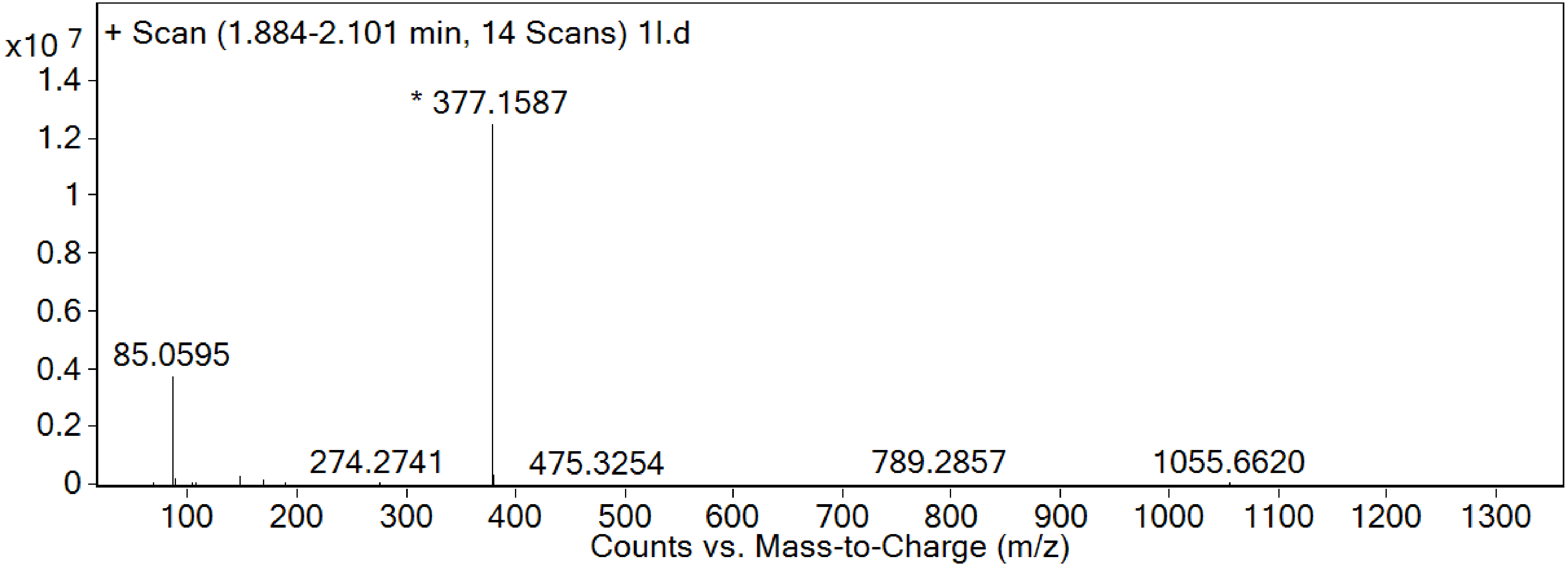

x10 7
+ Scan (1.884-2.101 min, 14 Scans) 1l.d
1.4
1.2
1
0.8
0.6
0.4
0.2
0
* 377.1587
85.0595
274.2741
475.3254
789.2857
1055.6620
100
200
300
400
500
600
700
800
900
1000
1100
1200
1300
Counts vs. Mass-to-Charge (m/z)

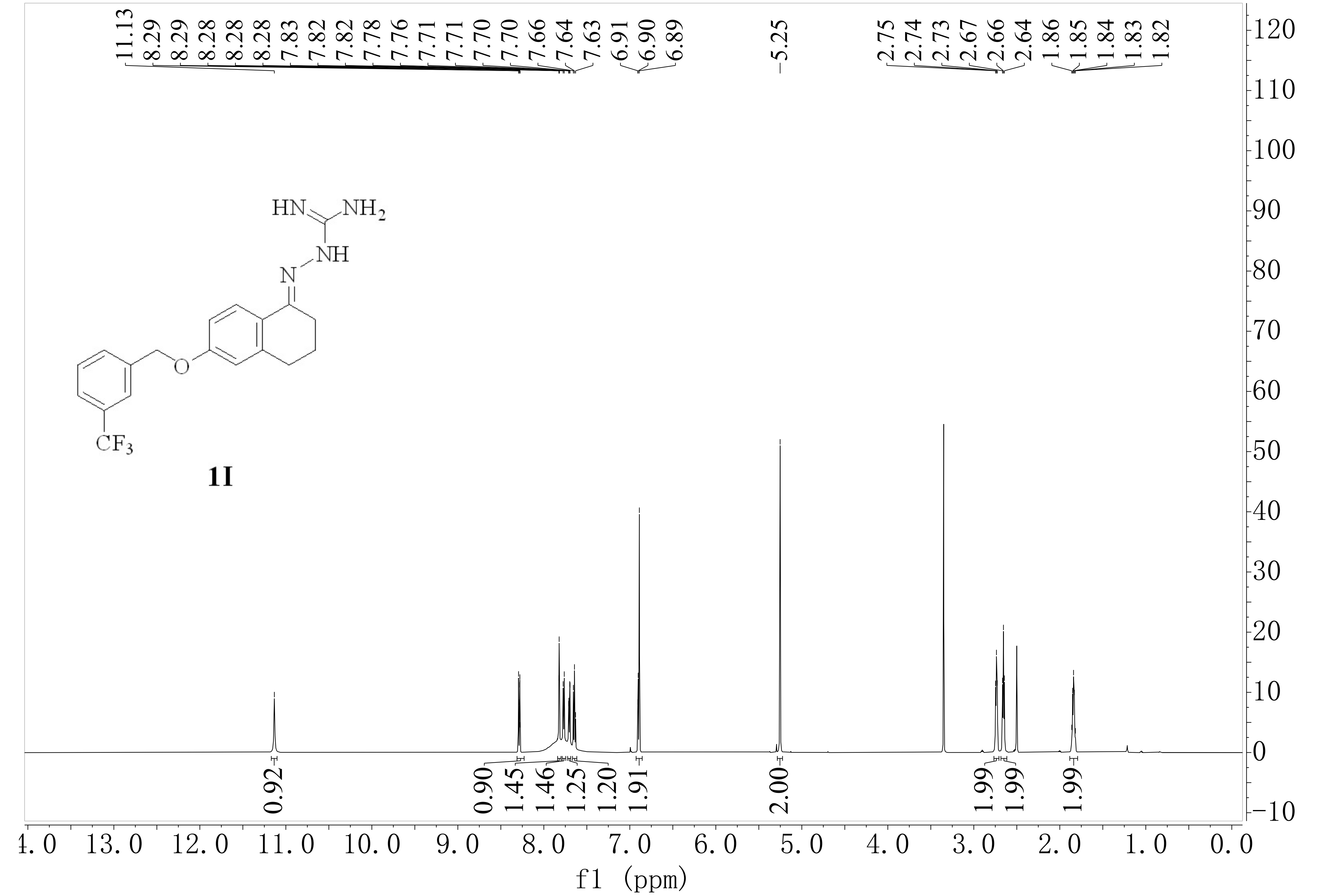

11.13
8.29
8.29
8.28
8.28
8.28
7.83
7.82
7.82
7.78
7.76
7.71
7.71
7.70
7.70
7.66
7.64
7.63
6.91
6.90
6.89
5.25
2.75
2.74
2.73
2.67
2.66
2.64
1.86
1.85
1.84
1.83
1.82
HN
NH2
NH
N
O
CF3
1I
0.92
0.90
1.45
1.46
1.25
1.20
1.91
2.00
1.99
1.99
1.99
f1 (ppm)

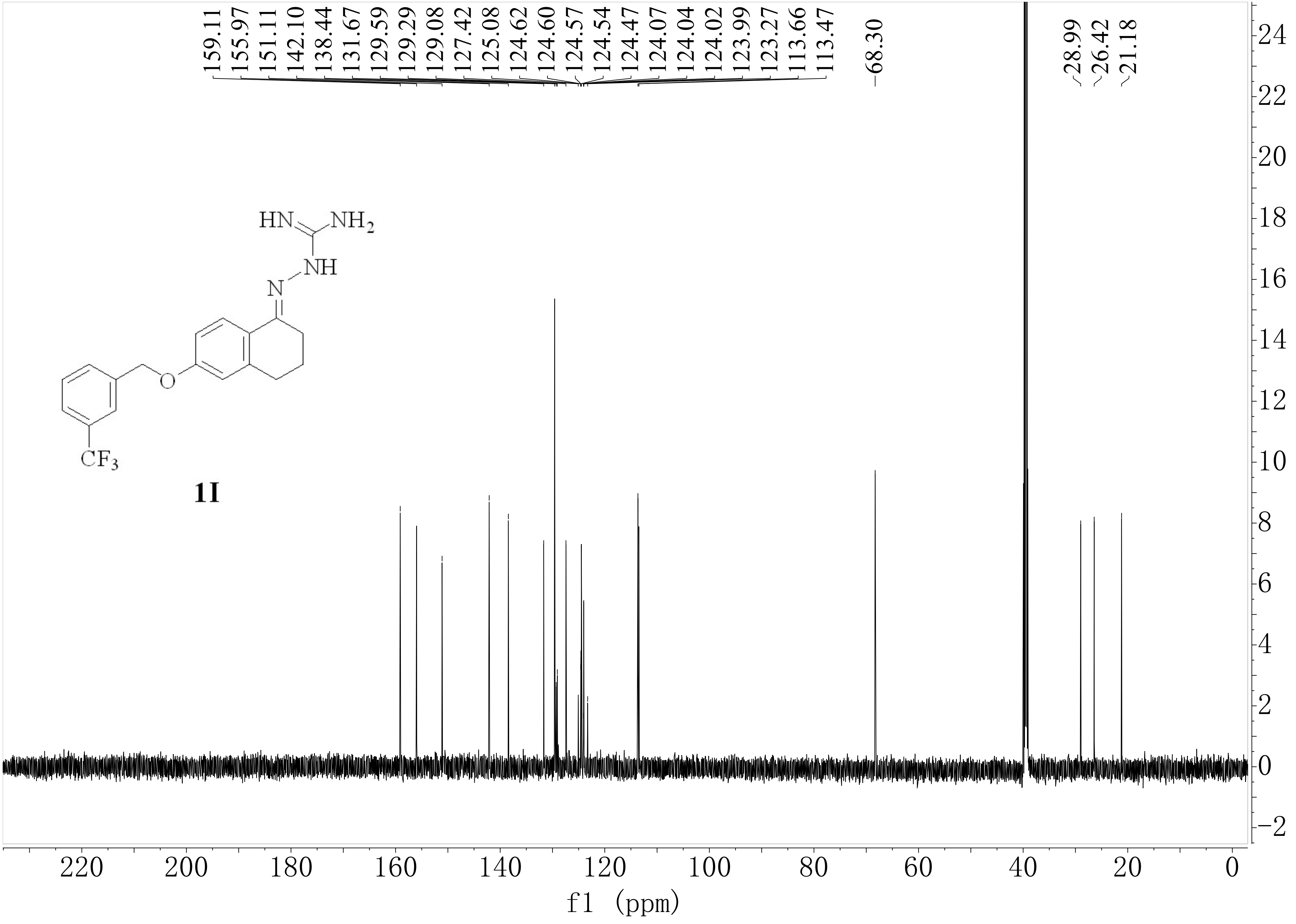

159.11
155.97
151.11
142.10
138.44
131.67
129.59
129.29
129.08
127.42
125.08
124.62
124.60
124.57
124.54
124.47
124.07
124.04
124.02
123.99
123.27
113.66
113.47
68.30
28.99
26.42
21.18
HN
NH2
NH
N
O
CF3
1I
f1 (ppm)

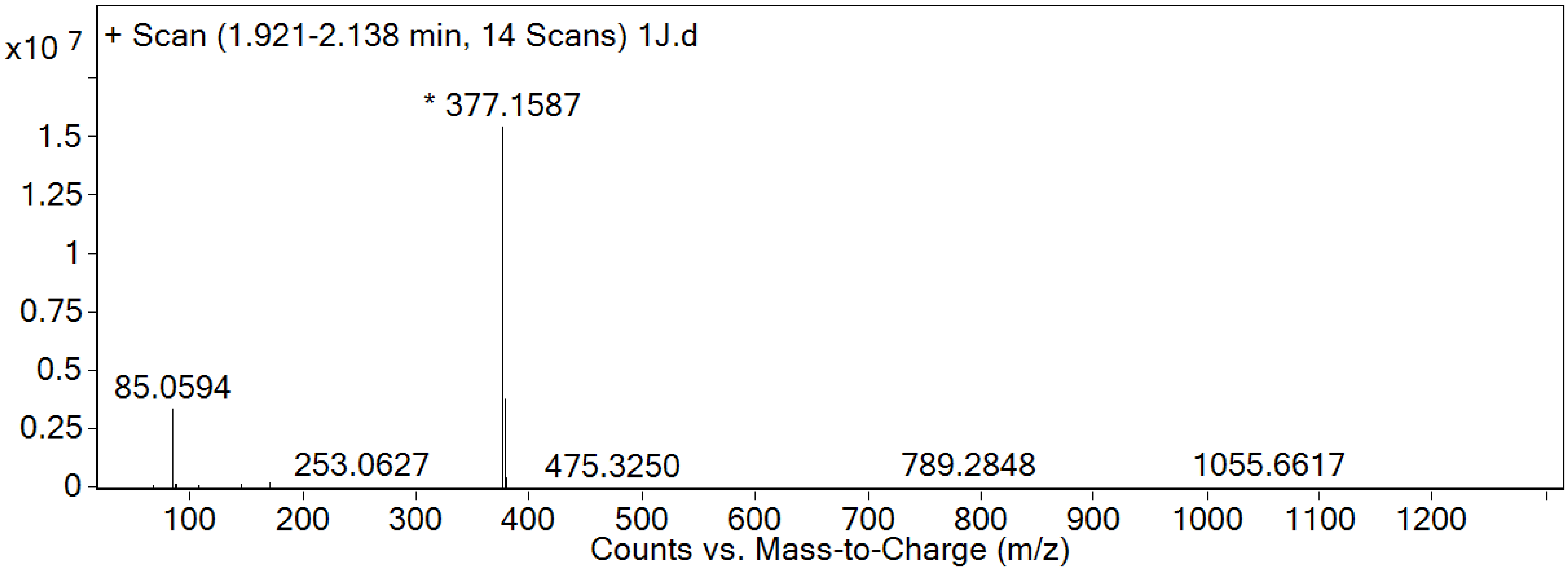

x10 7
+ Scan (1.921-2.138 min, 14 Scans) 1J.d
* 377.1587
1.5
1.25
1
0.75
0.5
0.25
0
85.0594
253.0627
475.3250
789.2848
1055.6617
100
200
300
400
500
600
700
800
900
1000
1100
1200
Counts vs. Mass-to-Charge (m/z)

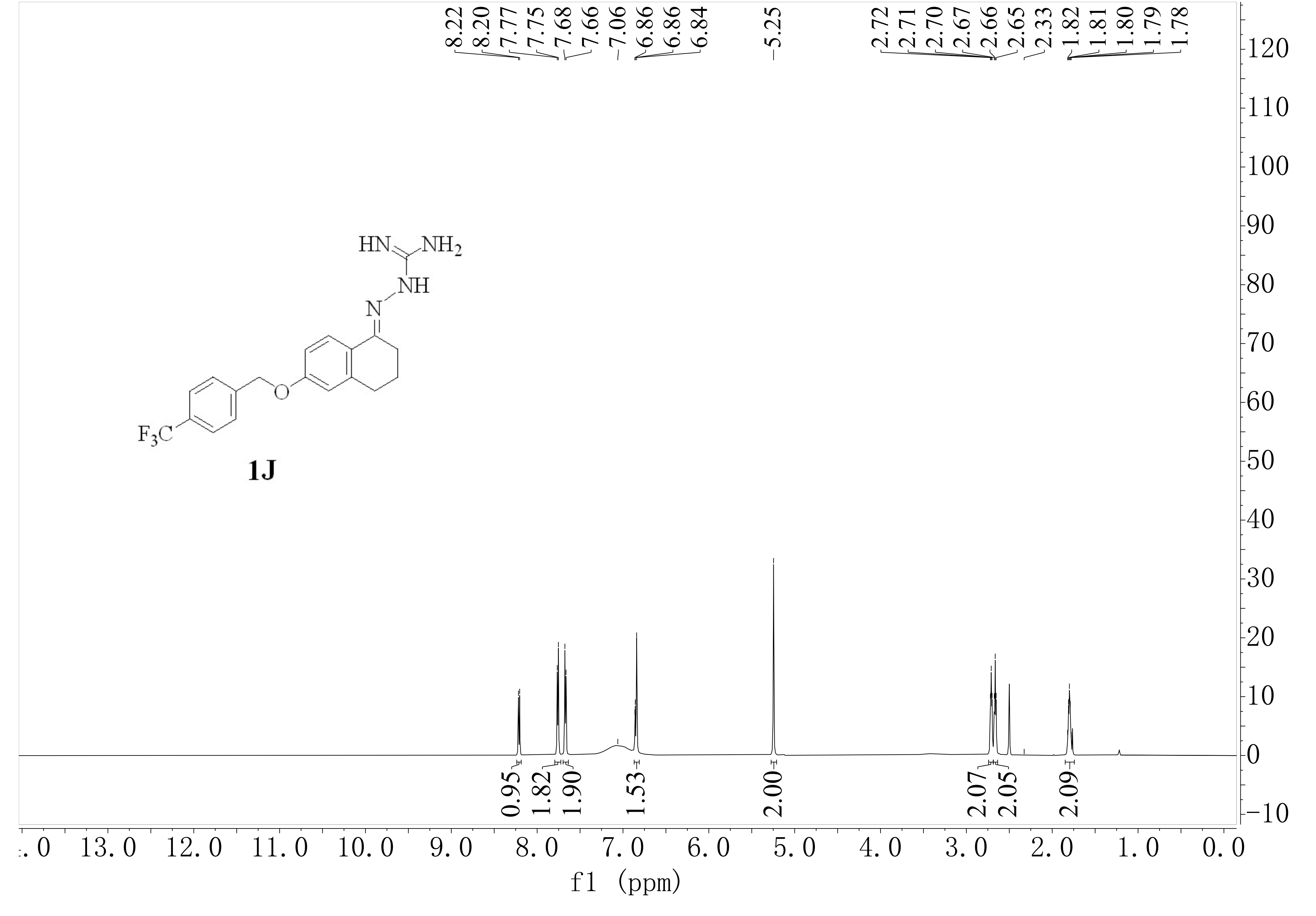

8.22
8.20
7.77
7.75
7.68
7.66
7.06
6.86
6.86
6.84
5.25
2.72
2.71
2.70
2.67
2.66
2.65
2.33
1.82
1.81
1.80
1.79
1.78
HN
NH2
NH
N
O
F3C
1J
0.95
1.82
1.90
1.53
2.00
2.07
2.05
2.09
f1 (ppm)

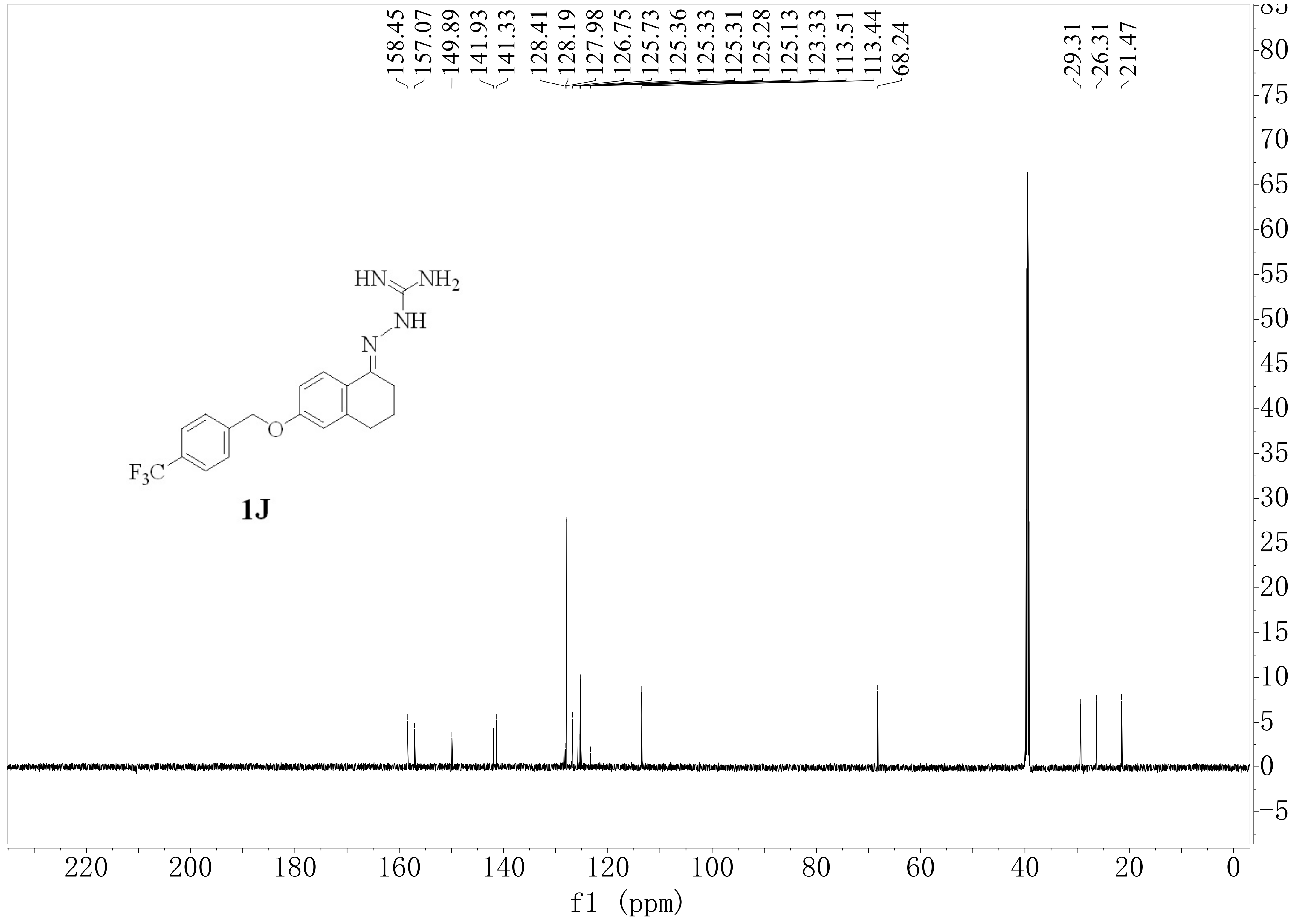
158.45
157.07
149.89
141.93
141.33
128.41
128.19
127.98
126.75
125.73
125.36
125.33
125.31
125.28
125.13
123.33
113.51
113.44
68.24
29.31
26.31
21.47
HN
NH2
NH
N
O
F3C
1J
f1 (ppm)

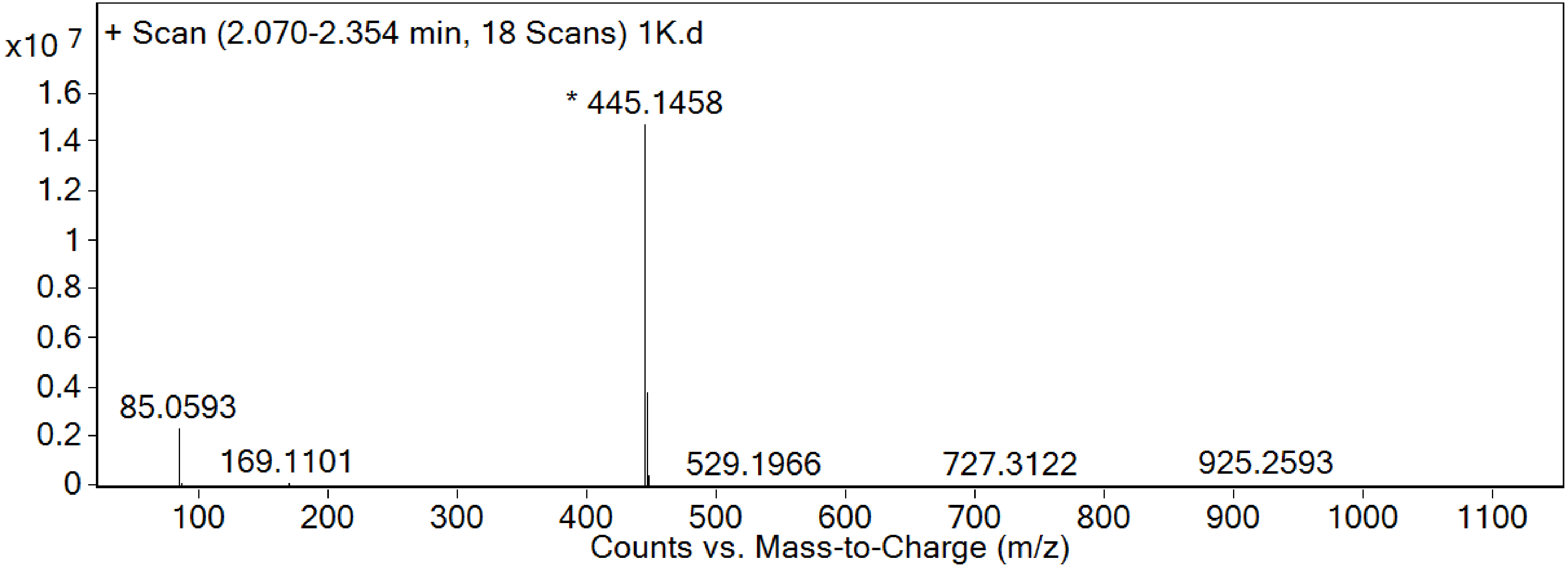
x10 7
+ Scan (2.070-2.354 min, 18 Scans) 1K.d
1.6
1.4
1.2
1
0.8
0.6
0.4
0.2
0
* 445.1458
85.0593
169.1101
529.1966
727.3122
925.2593
100
200
300
400
500
600
700
800
900
1000
1100
Counts vs. Mass-to-Charge (m/z)

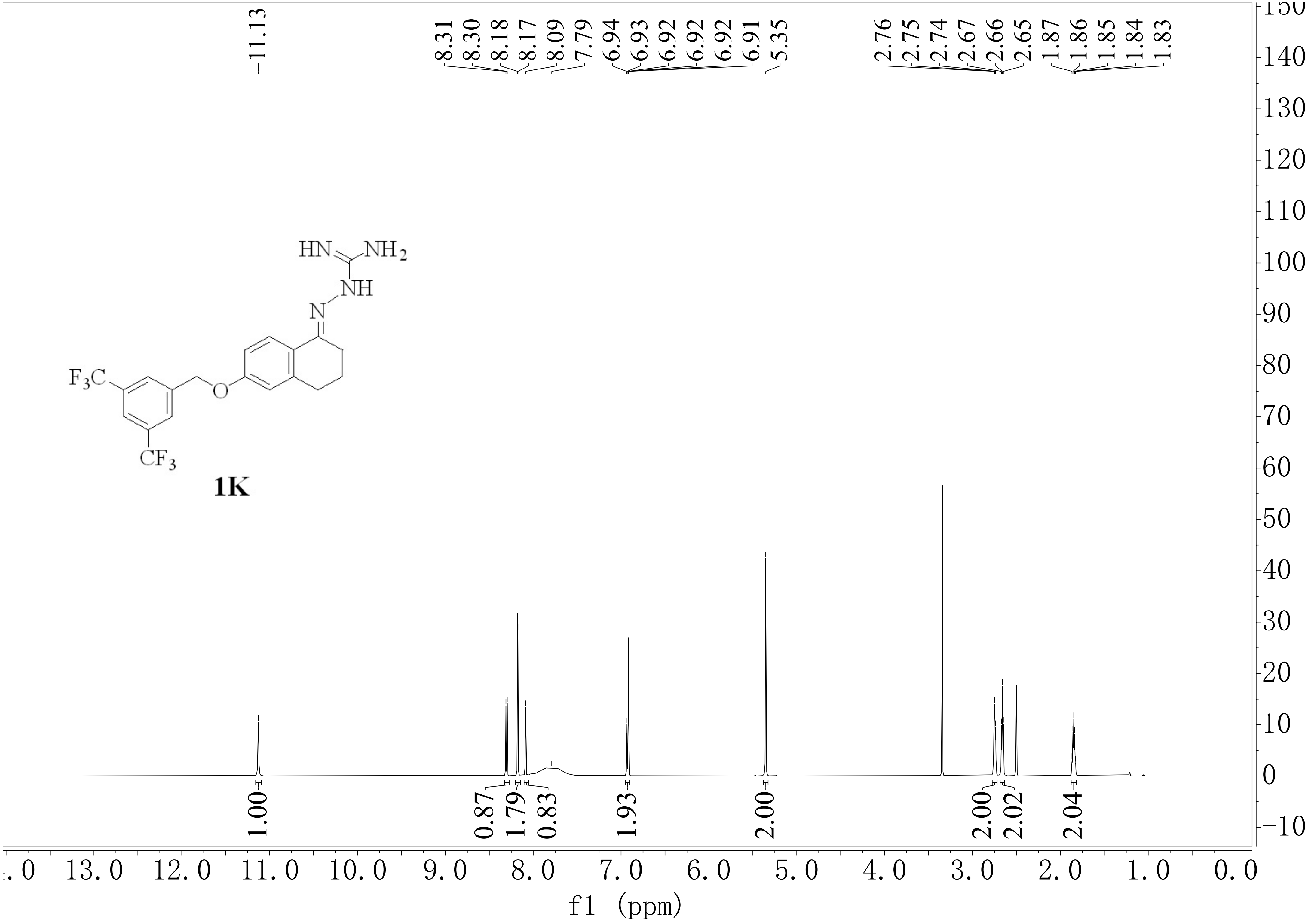

11.13
8.31
8.30
8.18
8.17
8.09
7.79
6.94
6.93
6.92
6.92
6.92
6.91
5.35
2.76
2.75
2.74
2.67
2.66
2.65
1.87
1.86
1.85
1.84
1.83
1.00
0.87
1.79
0.83
1.93
2.00
2.00
2.02
2.04
HN
NH2
NH
N
O
F3C
CF3
f1 (ppm)

1K

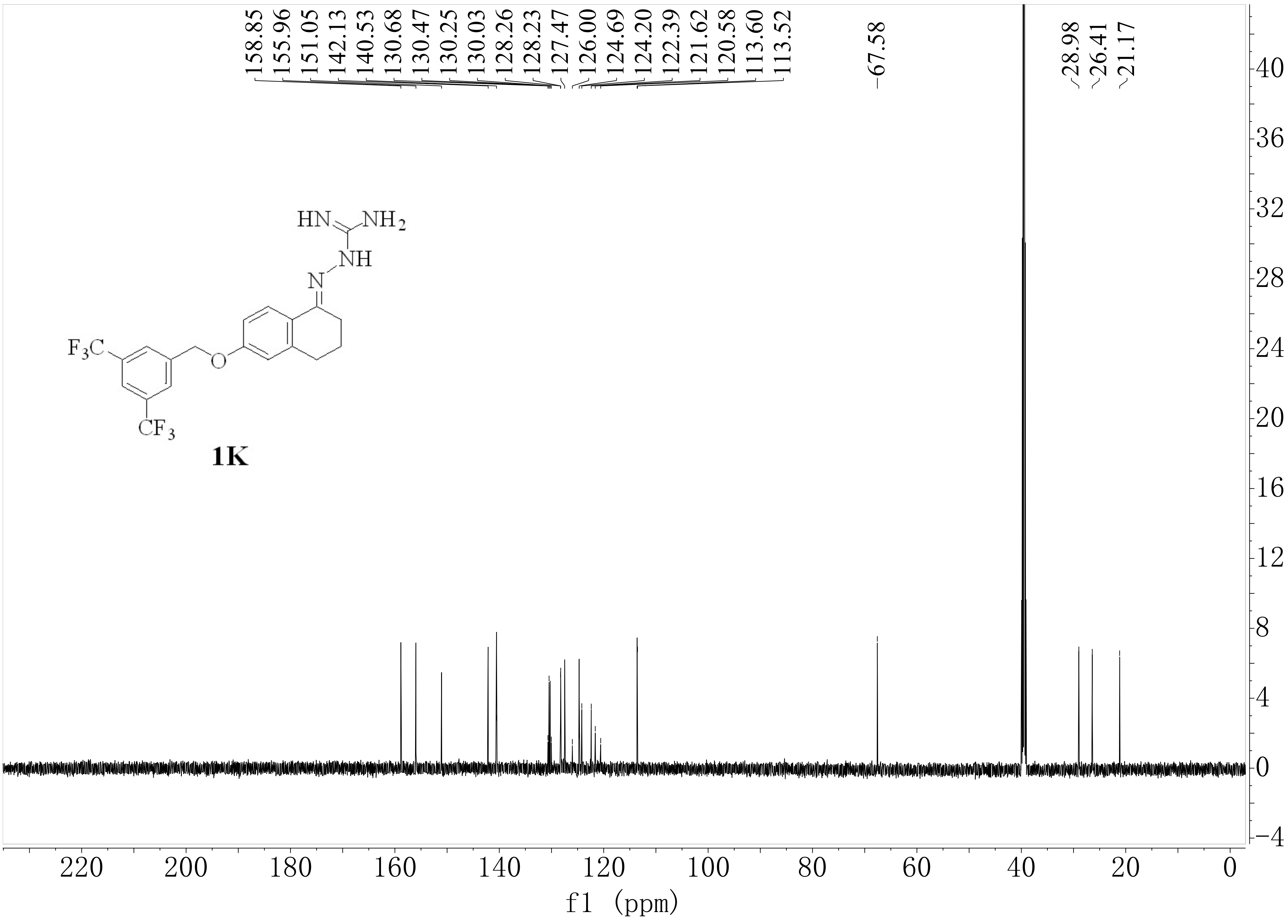

158.85
155.96
151.05
142.13
140.53
130.68
130.47
130.25
130.03
128.26
128.23
127.47
126.00
124.69
124.20
122.39
121.62
120.58
113.60
113.52
67.58
28.98
26.41
21.17
HN
NH2
NH
N
F3C
O
CF3
1K
f1 (ppm)

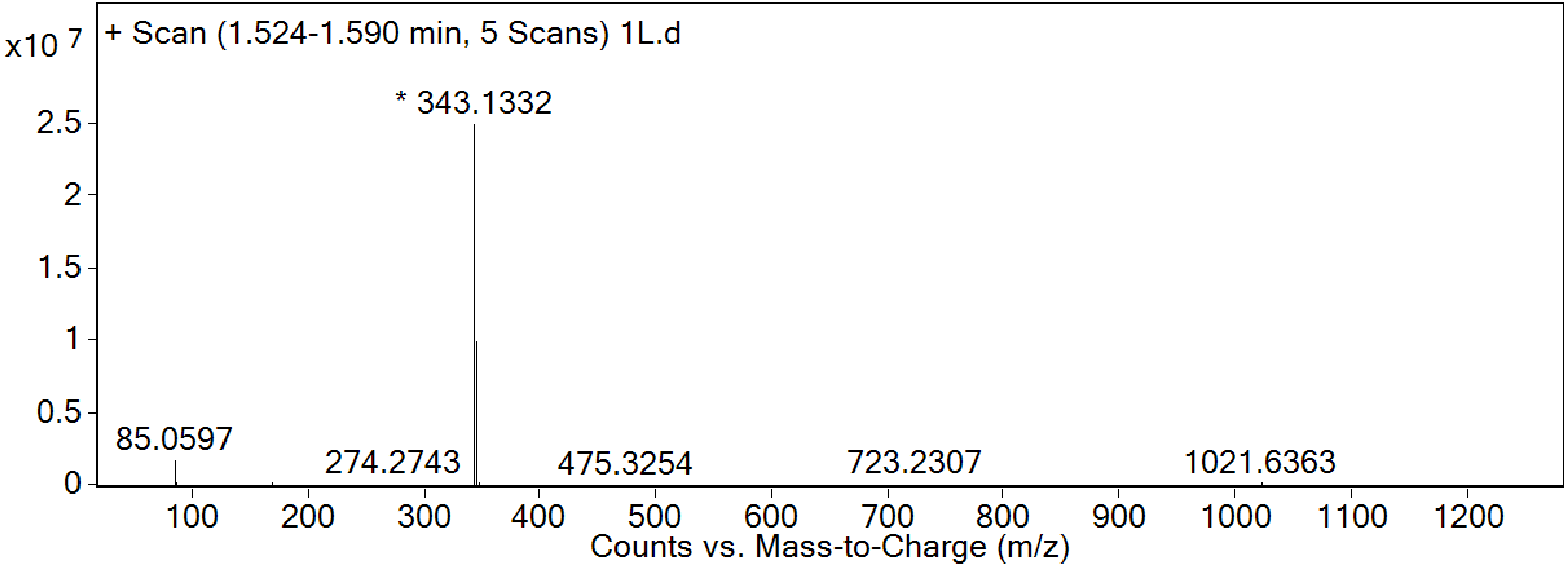
x10 7
+ Scan (1.524-1.590 min, 5 Scans) 1L.d
* 343.1332
2.5
2
1.5
1
0.5
0
85.0597
274.2743
475.3254
723.2307
1021.6363
100
200
300
400
500
600
700
800
900
1000
1100
1200
Counts vs. Mass-to-Charge (m/z)

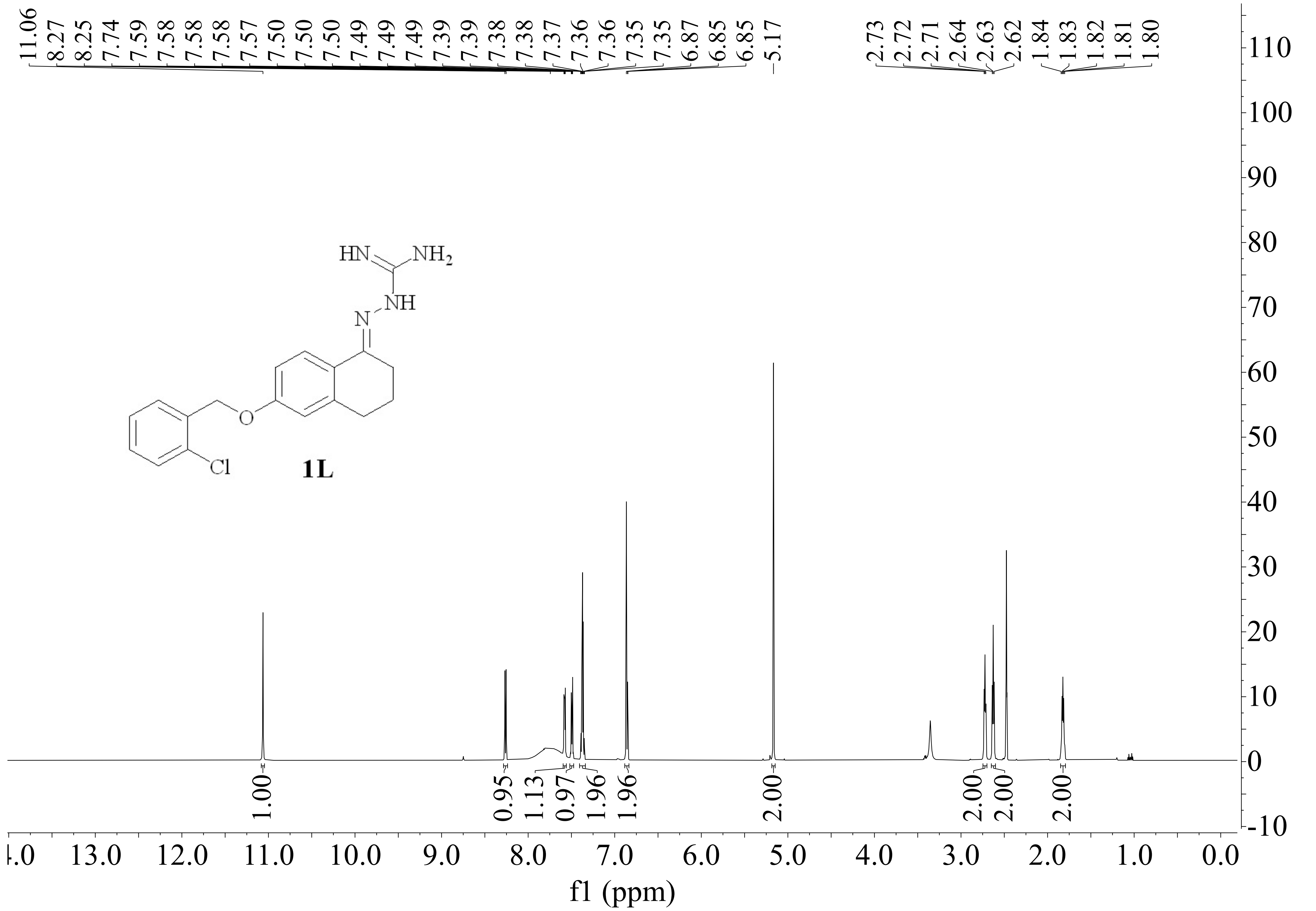

11.06
8.27
8.25
7.74
7.59
7.58
7.58
7.58
7.57
7.50
7.50
7.50
7.49
7.49
7.49
7.39
7.39
7.38
7.38
7.37
7.36
7.36
7.35
7.35
6.87
6.85
6.85
5.17
2.73
2.72
2.71
2.64
2.63
2.62
1.84
1.83
1.82
1.81
1.80
HN
NH2
NH
N
O
Cl
1L
1.00
0.95
1.13
0.97
1.96
1.96
2.00
2.00
2.00
2.00
f1 (ppm)

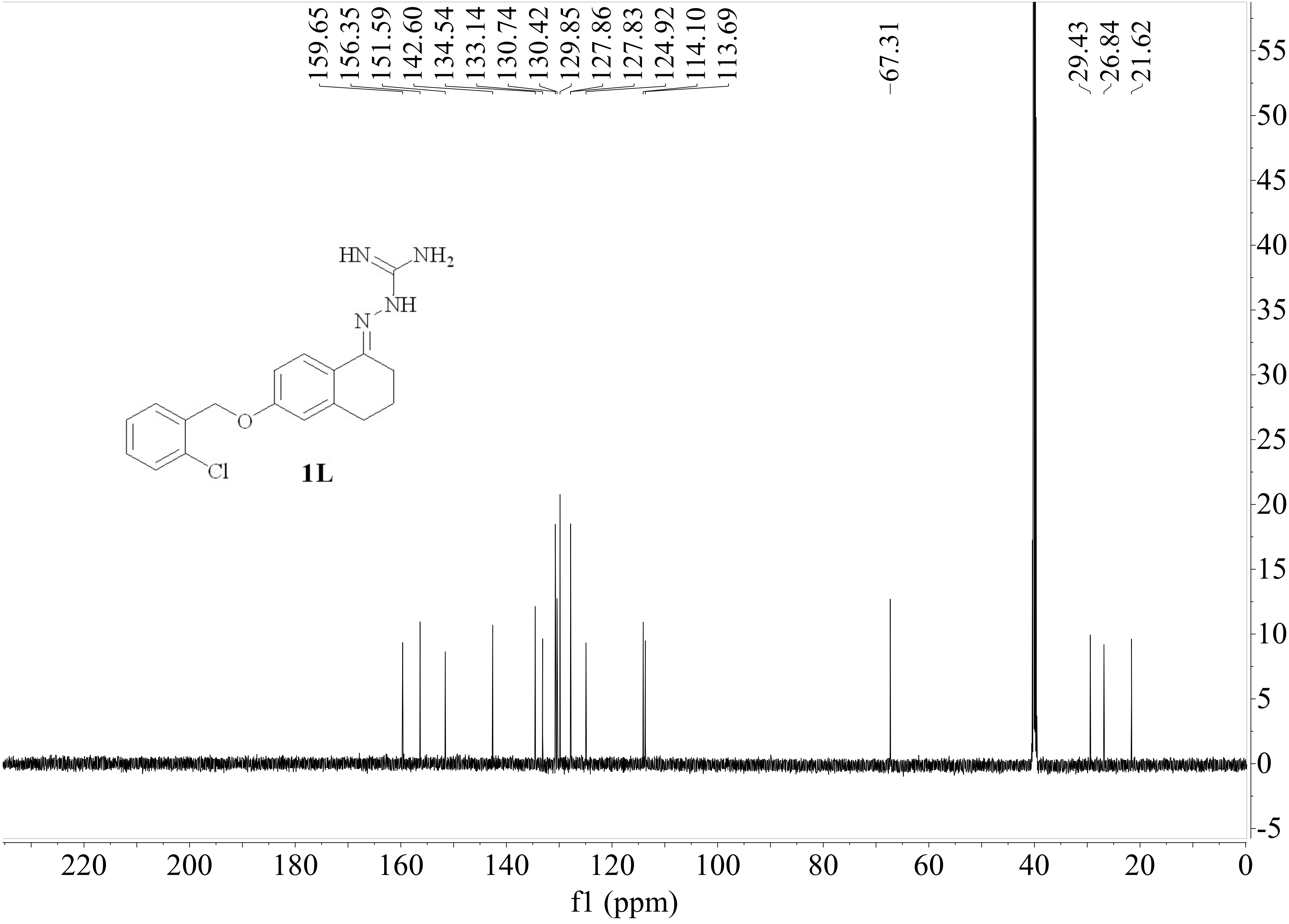

159.65
156.35
151.59
142.60
134.54
133.14
130.74
130.42
129.85
127.86
127.83
124.92
114.10
113.69
67.31
29.43
26.84
21.62
HN
NH2
NH
N
O
Cl
1L
55
50
45
40
35
30
25
20
15
10
5
0
-5
220
200
180
160
140
120
100
80
60
40
20
0
f1 (ppm)

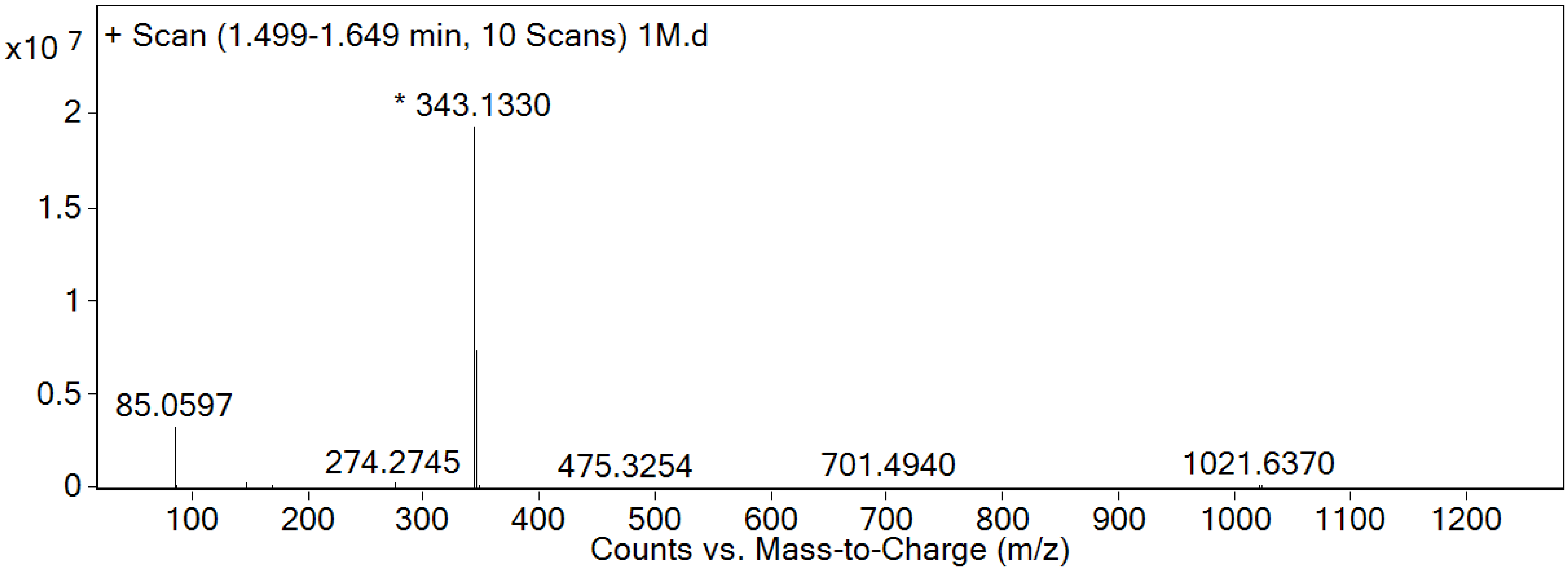
x10 7
+ Scan (1.499-1.649 min, 10 Scans) 1M.d
* 343.1330
85.0597
274.2745
475.3254
701.4940
1021.6370
2
1.5
1
0.5
0
100
200
300
400
500
600
700
800
900
1000
1100
1200
Counts vs. Mass-to-Charge (m/z)

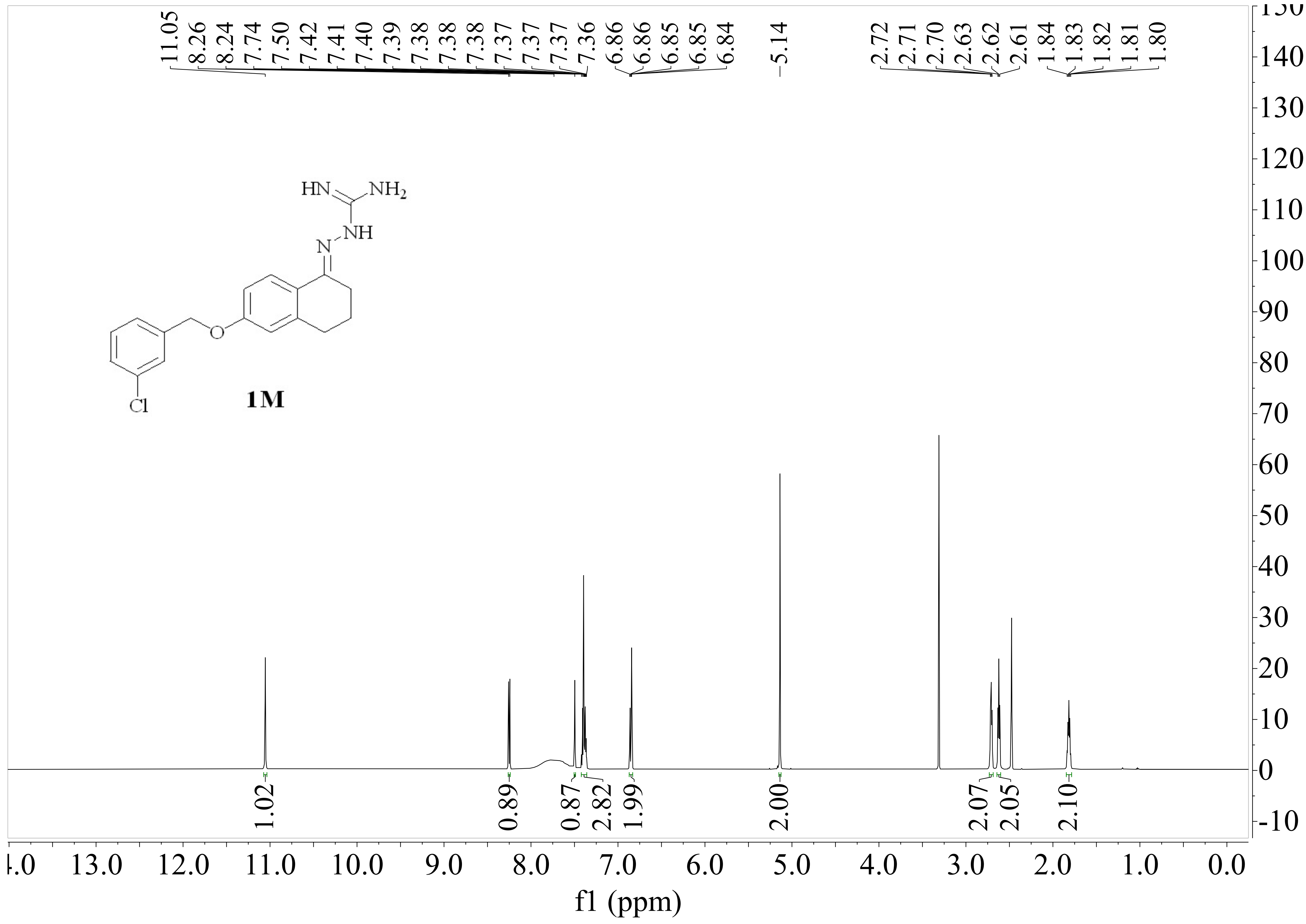

11.05
8.26
8.24
7.74
7.50
7.42
7.41
7.40
7.39
7.38
7.38
7.38
7.37
7.37
7.37
7.36
6.86
6.86
6.85
6.85
6.84
5.14
2.72
2.71
2.70
2.63
2.62
2.61
1.84
1.83
1.82
1.81
1.80
HN
NH2
NH
N
O
Cl
1M
1.02
0.89
0.87
2.82
1.99
2.00
2.07
2.05
2.10
f1 (ppm)

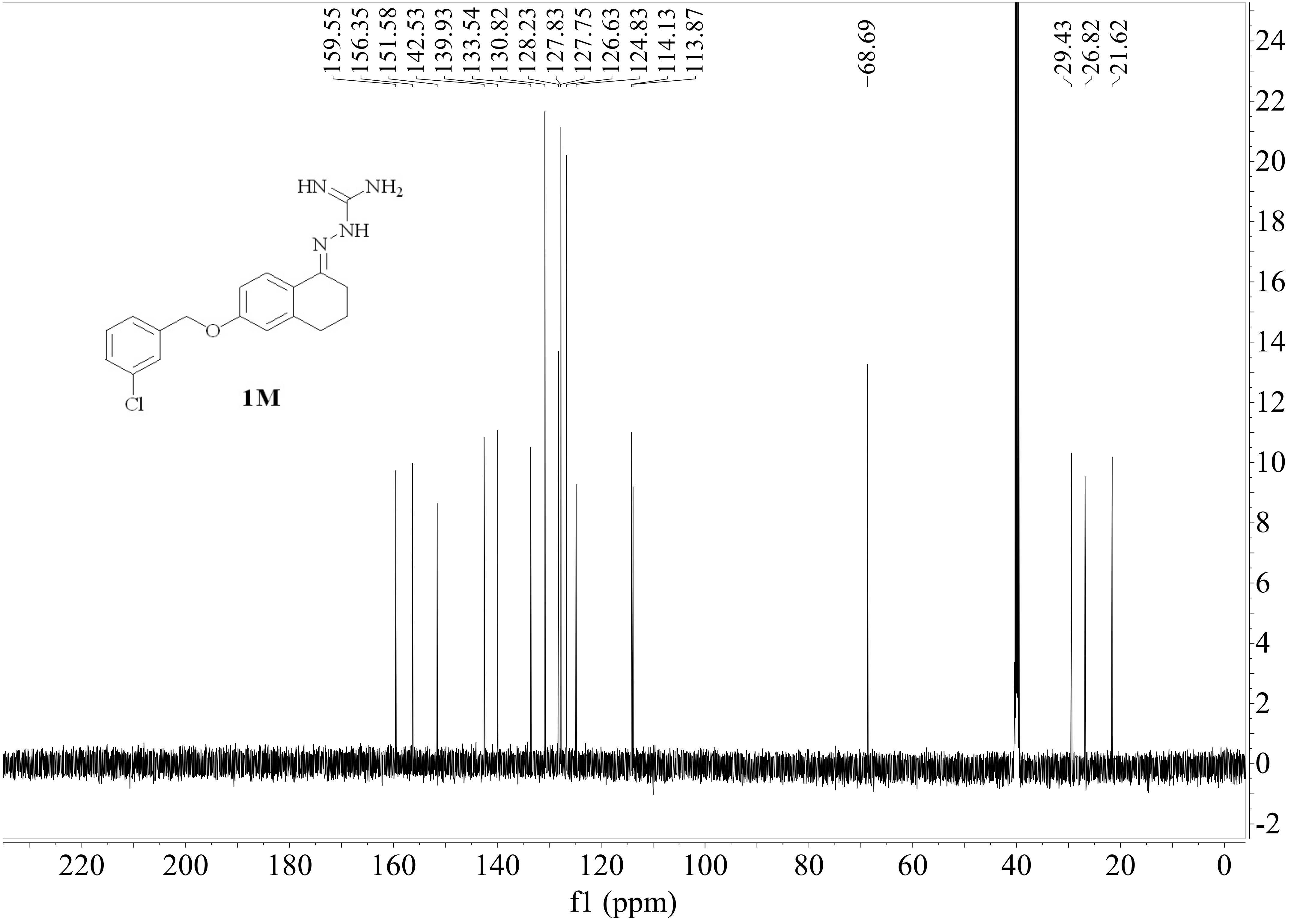

159.55
156.35
151.58
142.53
139.93
133.54
130.82
128.23
127.83
127.75
126.63
124.83
114.13
113.87
68.69
29.43
26.82
21.62
HN
NH2
NH
N
O
Cl
1M
24
22
20
18
16
14
12
10
8
6
4
2
0
-2
220
200
180
160
140
120
100
80
60
40
20
0
f1 (ppm)

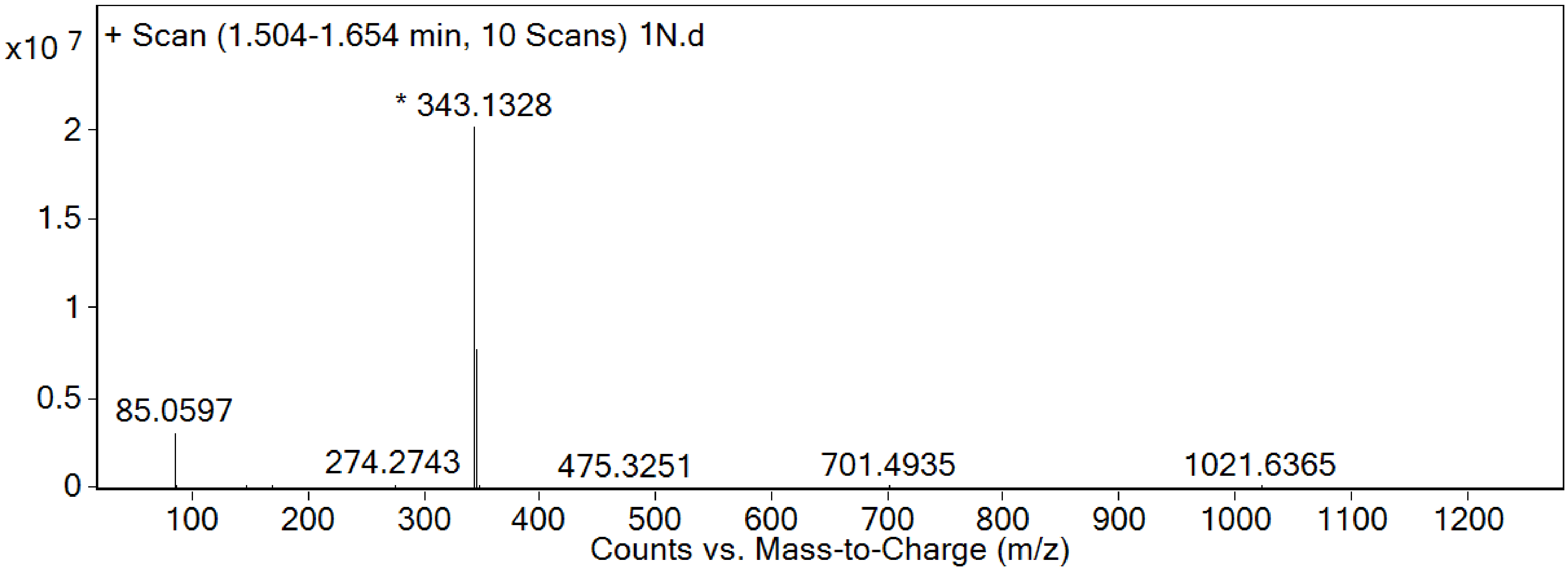

x10 7
+ Scan (1.504-1.654 min, 10 Scans) 1N.d
* 343.1328
85.0597
274.2743
475.3251
701.4935
1021.6365
0
0.5
1
1.5
2
100
200
300
400
500
600
700
800
900
1000
1100
1200
Counts vs. Mass-to-Charge (m/z)

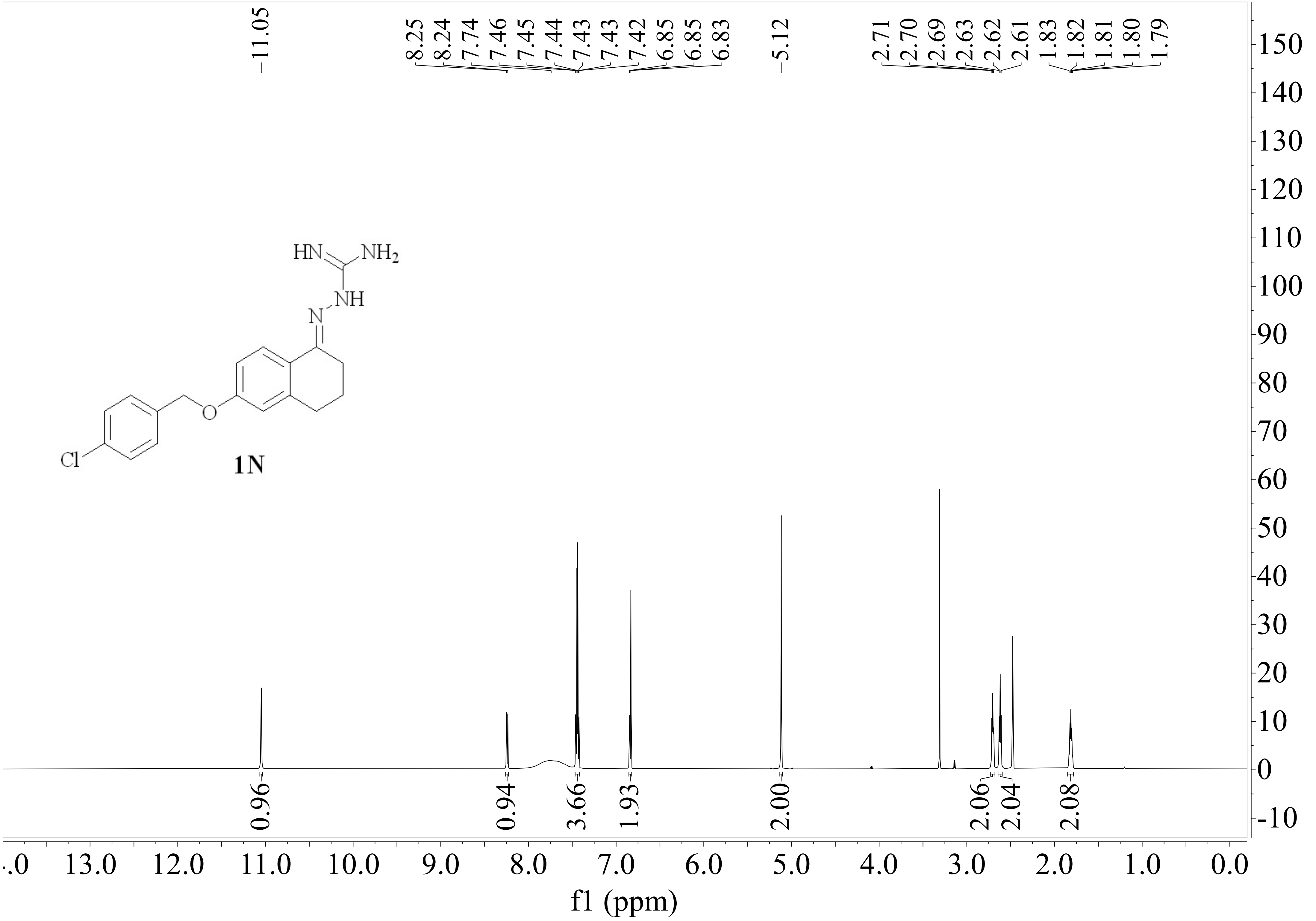

11.05
8.25 8.24 7.74 7.46 7.45 7.44 7.43 7.43 7.42 6.85 6.85 6.83
5.12
2.71 2.70 2.69 2.63 2.62 2.61 1.83 1.82 1.81 1.80 1.79
HN NH2
NH
N
O
Cl
1N
0.96
0.94
3.66
1.93
2.00
2.06
2.04
2.08
150 140 130 120 110 100 90 80 70 60 50 40 30 20 10 0 -10
.0 13.0 12.0 11.0 10.0 9.0 8.0 7.0 6.0 5.0 4.0 3.0 2.0 1.0 0.0
f1 (ppm)

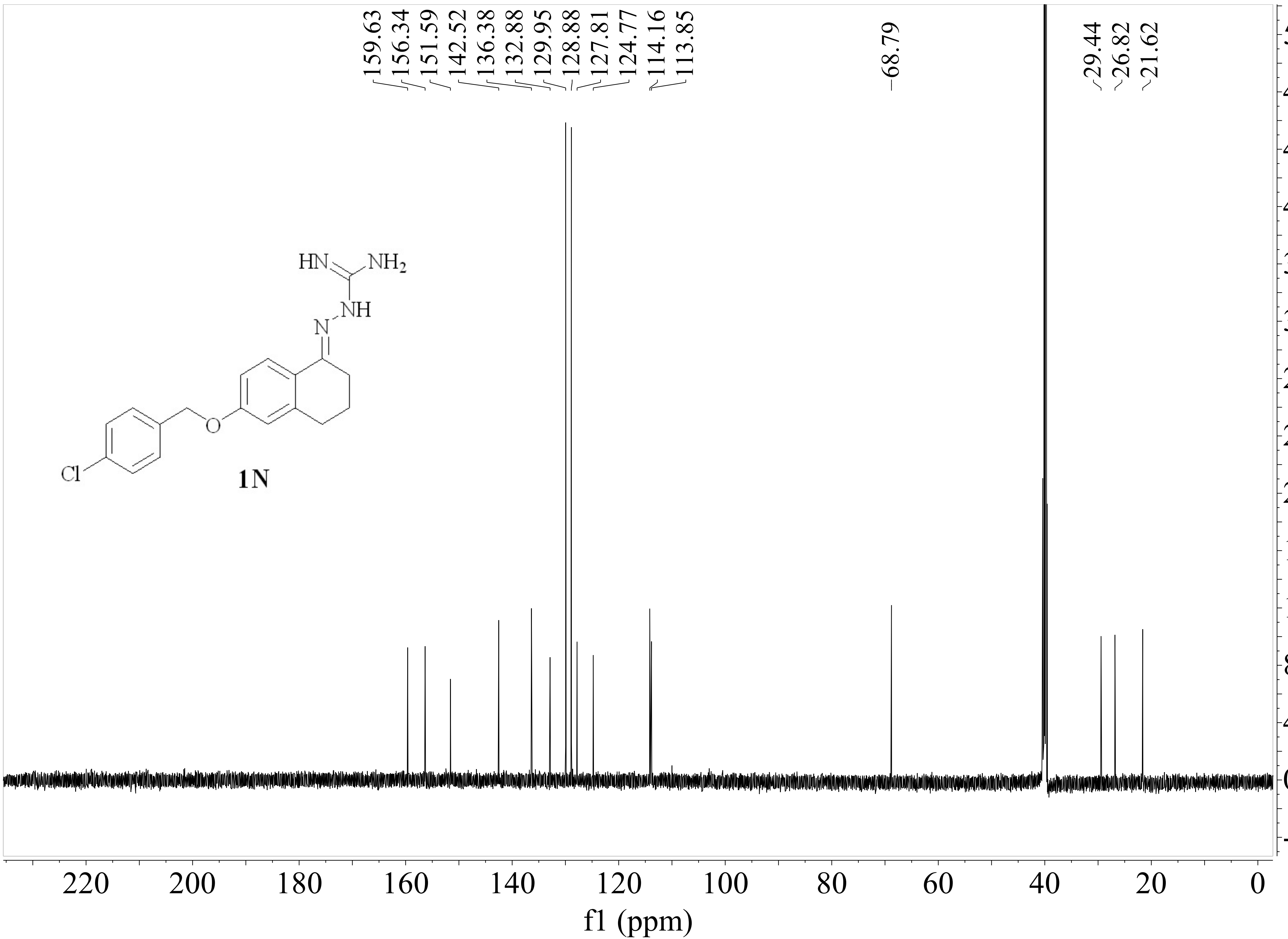

159.63
156.34
151.59
142.52
136.38
132.88
129.95
128.88
127.81
124.77
114.16
113.85
68.79
29.44
26.82
21.62
HN
NH2
NH
N
O
Cl
1N
220
200
180
160
140
120
100
80
60
40
20
0
f1 (ppm)
52
48
44
40
36
32
28
24
20
16
12
8
4
0
-4

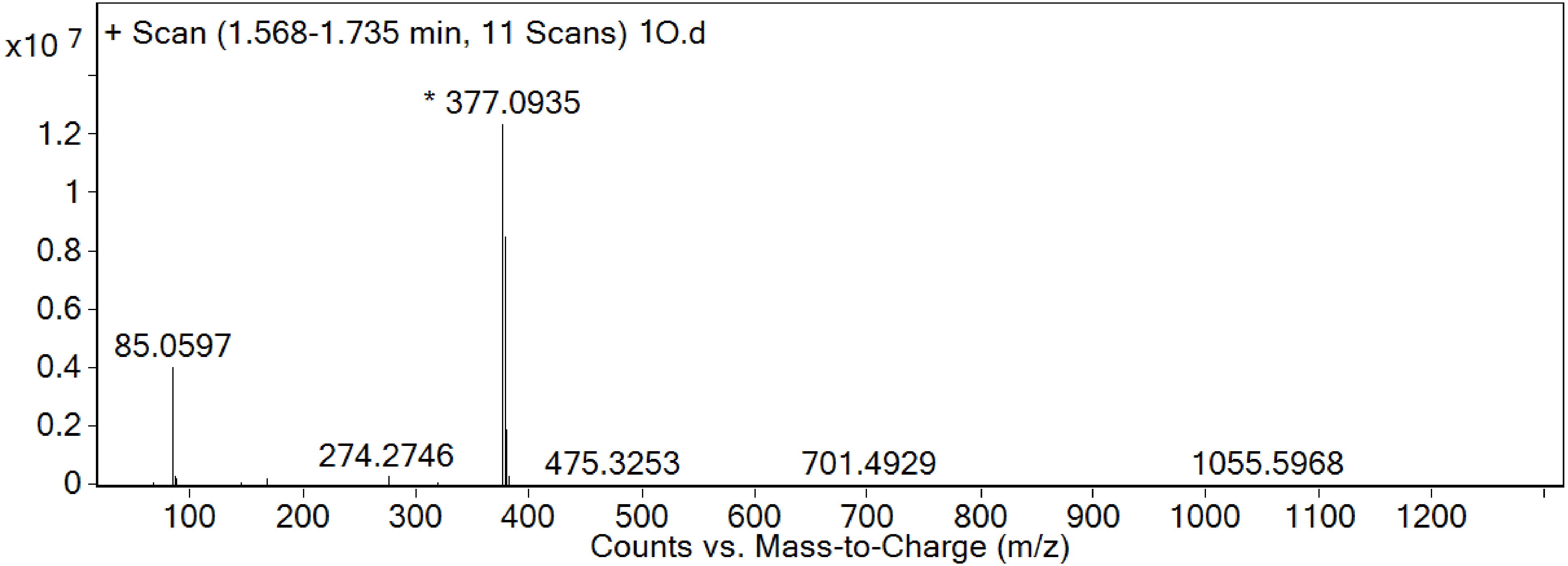
x10 7
+ Scan (1.568-1.735 min, 11 Scans) 1O.d
* 377.0935
85.0597
274.2746
475.3253
701.4929
1055.5968
1.2
1
0.8
0.6
0.4
0.2
0
100
200
300
400
500
600
700
800
900
1000
1100
1200
Counts vs. Mass-to-Charge (m/z)

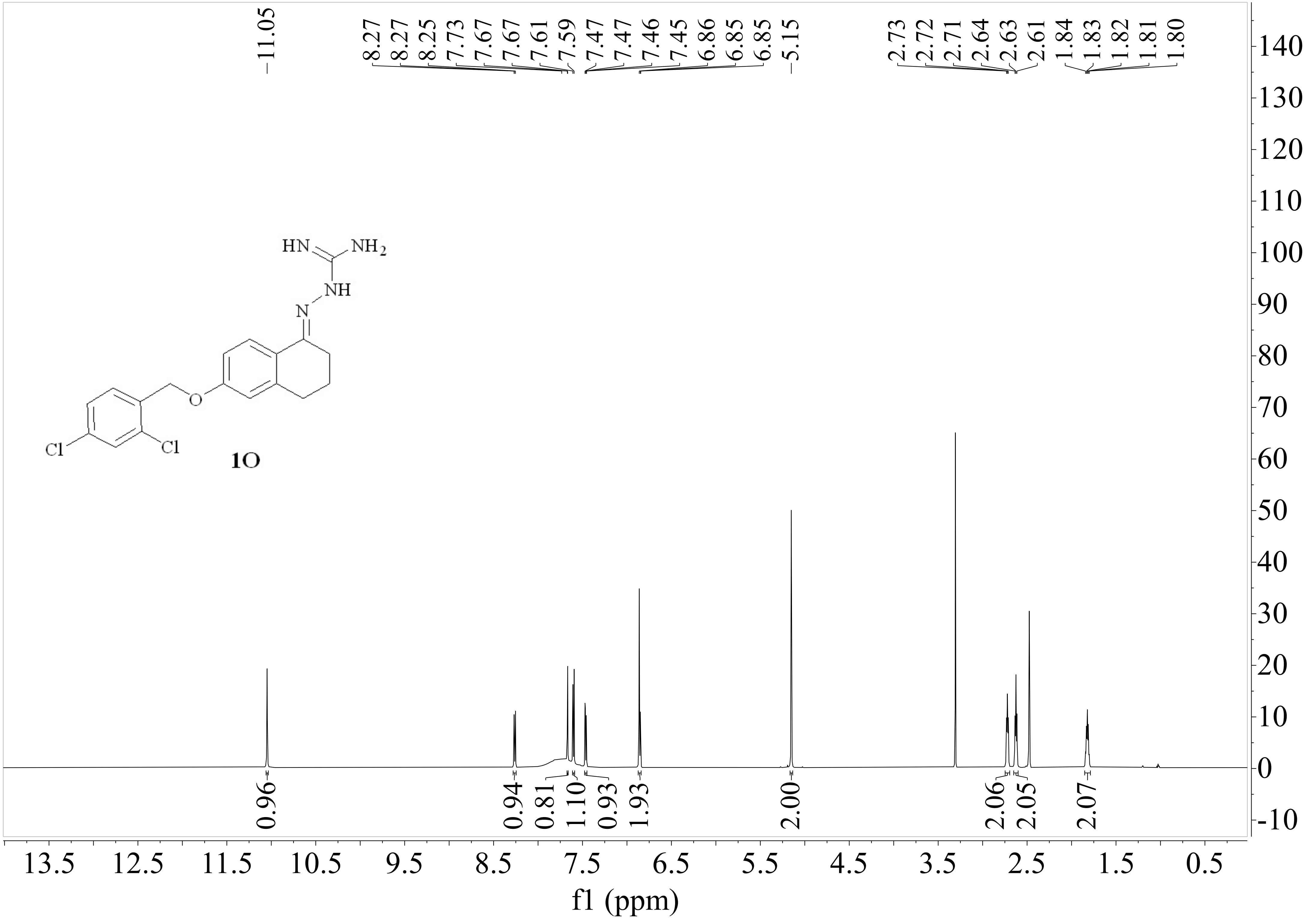

11.05
8.27 8.27 8.25 7.73 7.67 7.67 7.61 7.59 7.47 7.47 7.46 7.45 6.86 6.85 6.85
5.15
2.73 2.72 2.71 2.64 2.63 2.61 1.84 1.83 1.82 1.81 1.80
HN
NH2
NH
N
O
Cl
Cl
1O
0.96
0.94
0.81
1.10
0.93
1.93
2.00
2.06
2.05
2.07
140
130
120
110
100
90
80
70
60
50
40
30
20
10
0
-10
13.5
12.5
11.5
10.5
9.5
8.5
7.5
6.5
5.5
4.5
3.5
2.5
1.5
0.5
f1 (ppm)

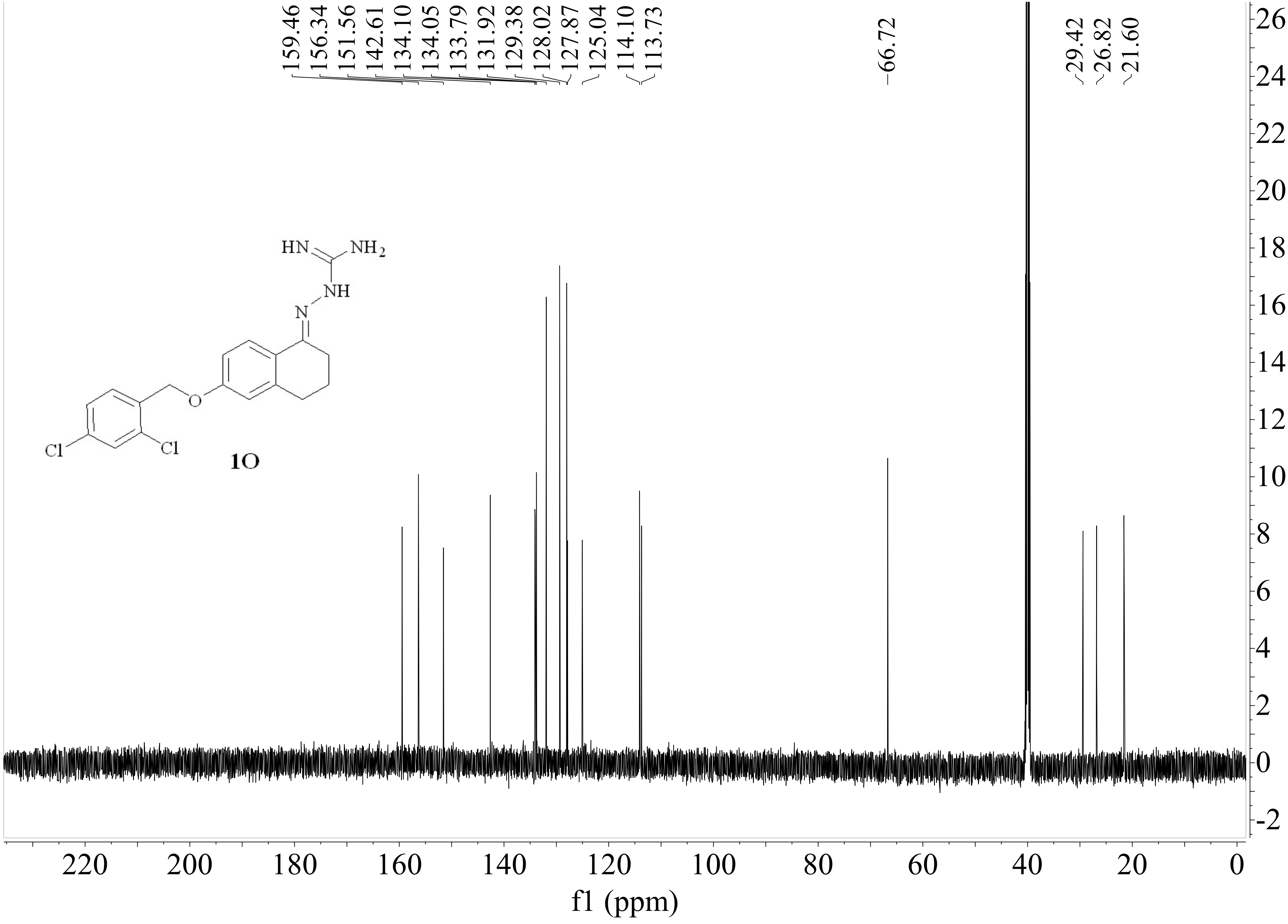
159.46
156.34
151.56
142.61
134.10
134.05
133.79
131.92
129.38
128.02
127.87
125.04
114.10
113.73
66.72
29.42
26.82
21.60
HN
NH2
NH
N
O
Cl
Cl
1O
220
200
180
160
140
120
100
80
60
40
20
0
f1 (ppm)
26
24
22
20
18
16
14
12
10
8
6
4
2
0
-2

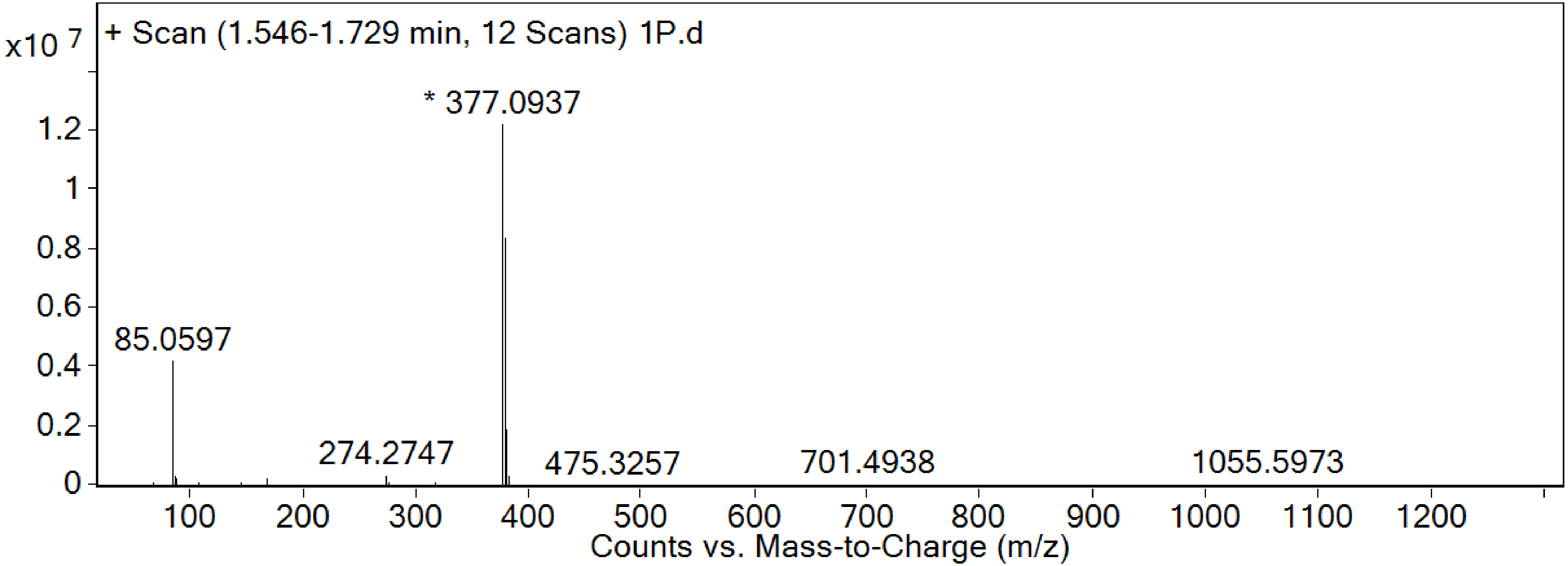

+ Scan (1.546-1.729 min, 12 Scans) 1P.d
x10 7
1.2
1
0.8
0.6
0.4
0.2
0
* 377.0937
85.0597
274.2747
475.3257
701.4938
1055.5973
100
200
300
400
500
600
700
800
900
1000
1100
1200
Counts vs. Mass-to-Charge (m/z)

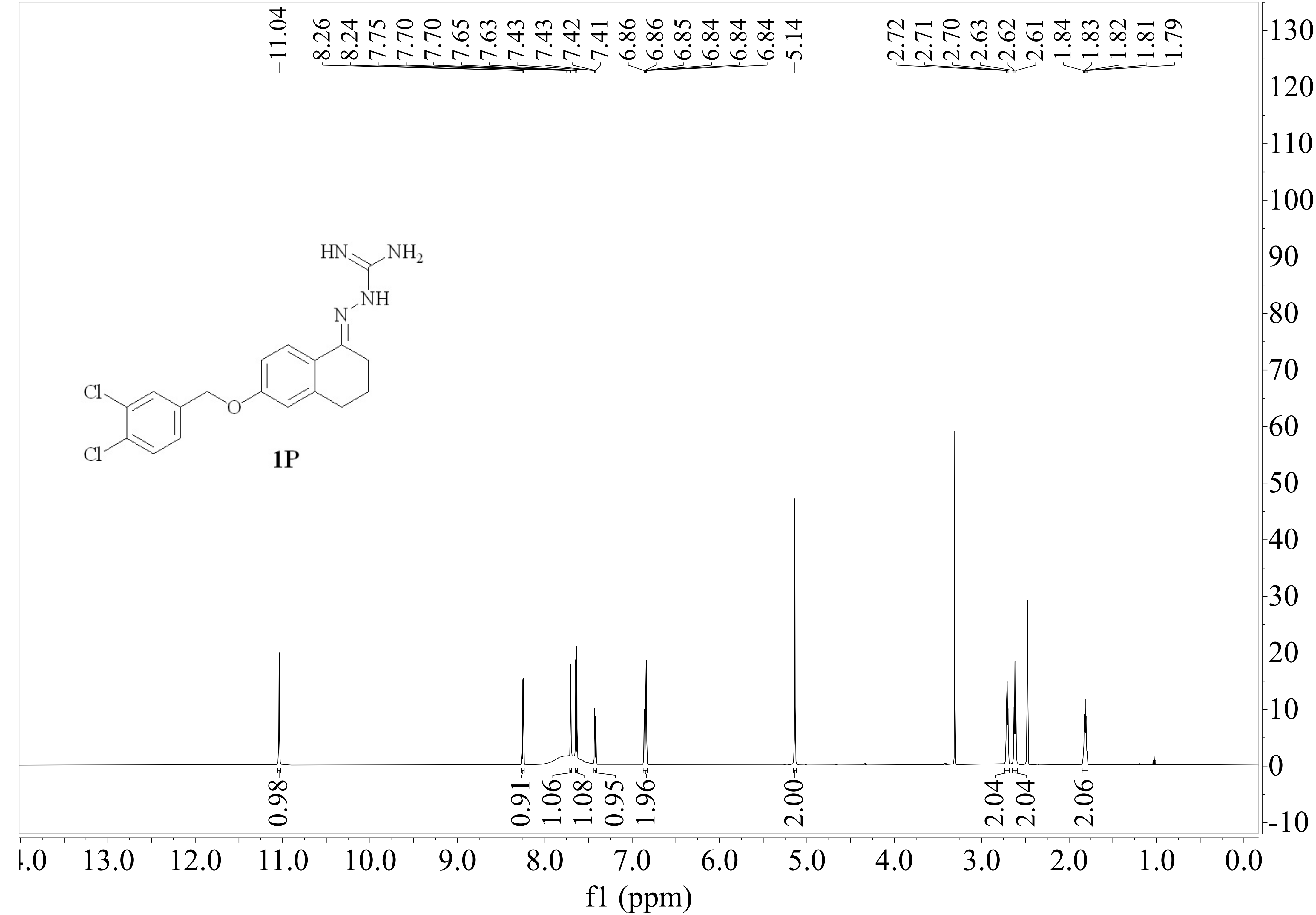

11.04
8.26
8.24
7.75
7.70
7.70
7.65
7.63
7.43
7.43
7.42
7.41
6.86
6.86
6.85
6.84
6.84
6.84
5.14
2.72
2.71
2.70
2.63
2.62
2.61
1.84
1.83
1.82
1.81
1.79
HN
NH2
NH
N
Cl
Cl
O
1P
0.98
0.91
1.06
1.08
0.95
1.96
2.00
2.04
2.04
2.06
f1 (ppm)

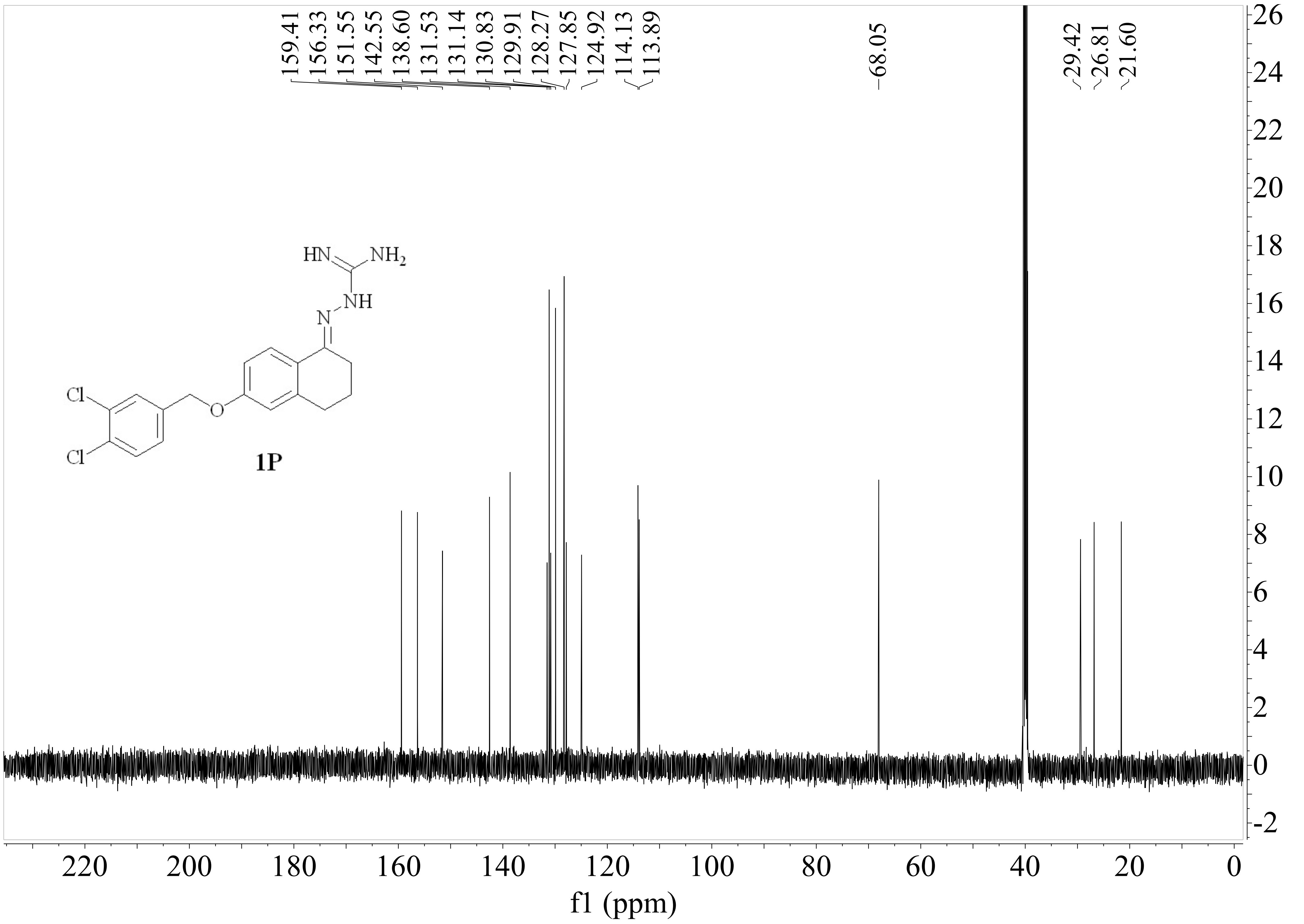

159.41
156.33
151.55
142.55
138.60
131.53
131.14
130.83
129.91
128.27
127.85
124.92
114.13
113.89
68.05
29.42
26.81
21.60
HN
NH2
NH
N
Cl
Cl
O
1P
f1 (ppm)

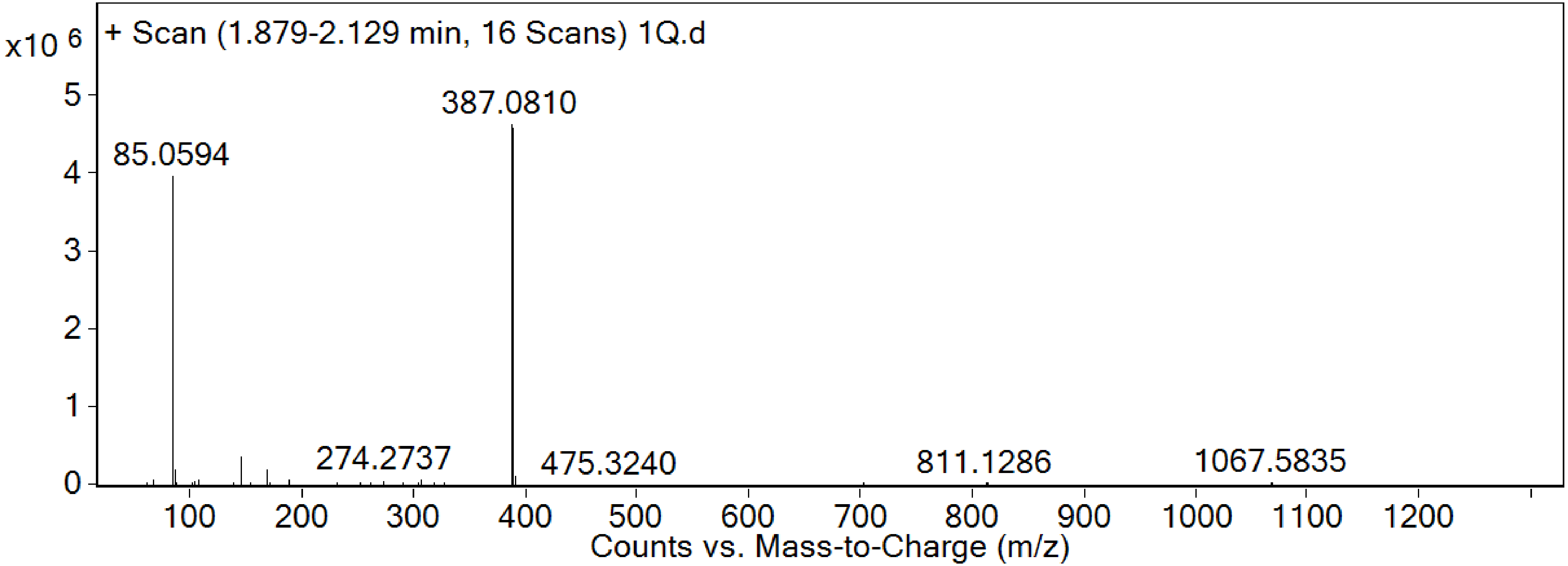

x10 6
+ Scan (1.879-2.129 min, 16 Scans) 1Q.d
5
4
3
2
1
0
387.0810
85.0594
274.2737
475.3240
811.1286
1067.5835
100
200
300
400
500
600
700
800
900
1000
1100
1200
Counts vs. Mass-to-Charge (m/z)

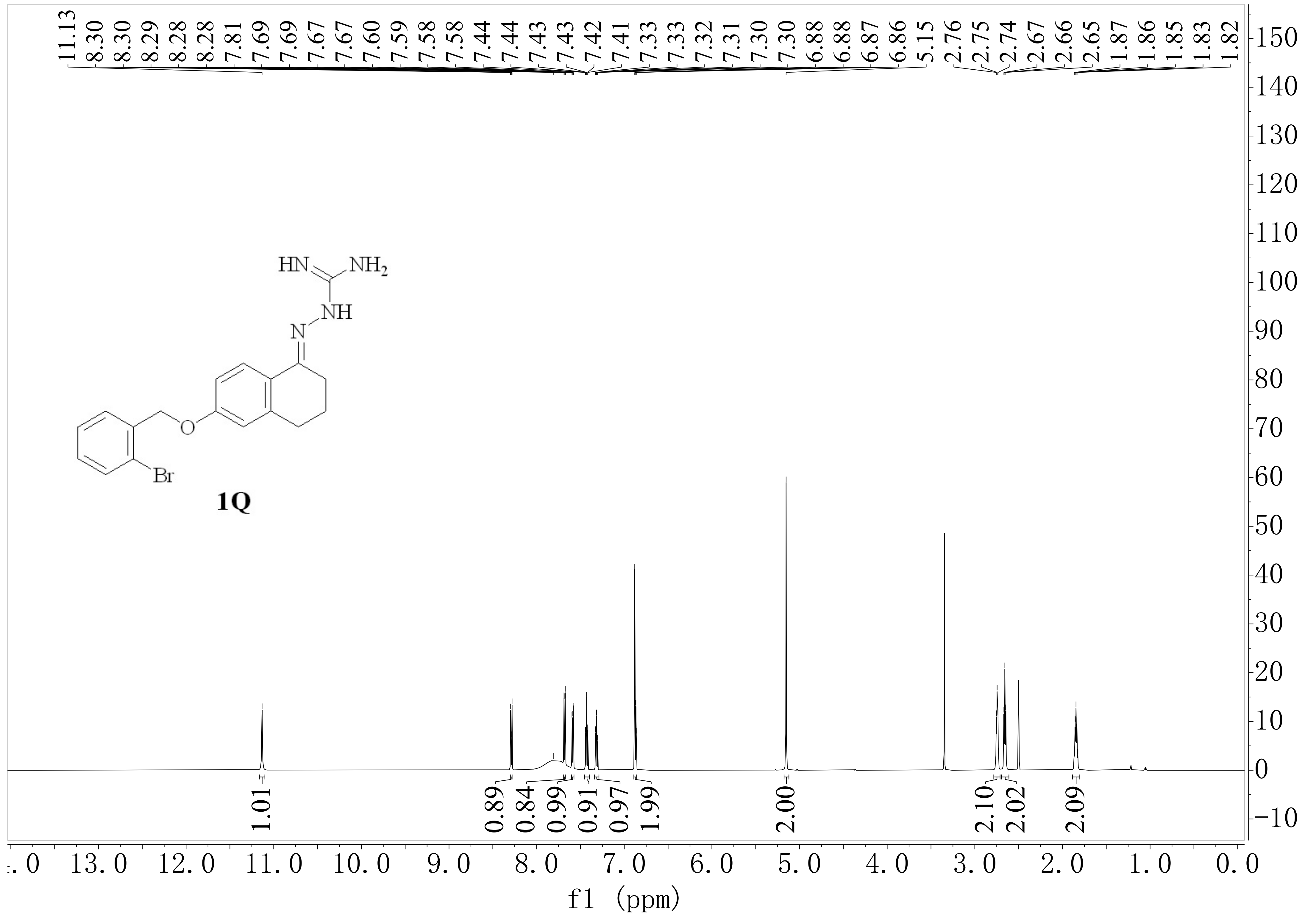
11.13 8.30 8.30 8.29 8.28 8.28 7.81 7.69 7.69 7.67 7.67 7.60 7.59 7.58 7.58 7.44 7.44 7.43 7.43 7.42 7.41 7.33 7.33 7.32 7.31 7.30 7.30 6.88 6.88 6.87 6.86 5.15 2.76 2.75 2.74 2.67 2.66 2.65 1.87 1.86 1.85 1.83 1.82
HN NH2
NH
N
O
Br
1Q
1.01
0.89
0.84
0.99
0.91
0.97
1.99
2.00
2.10
2.02
2.09
f1 (ppm)

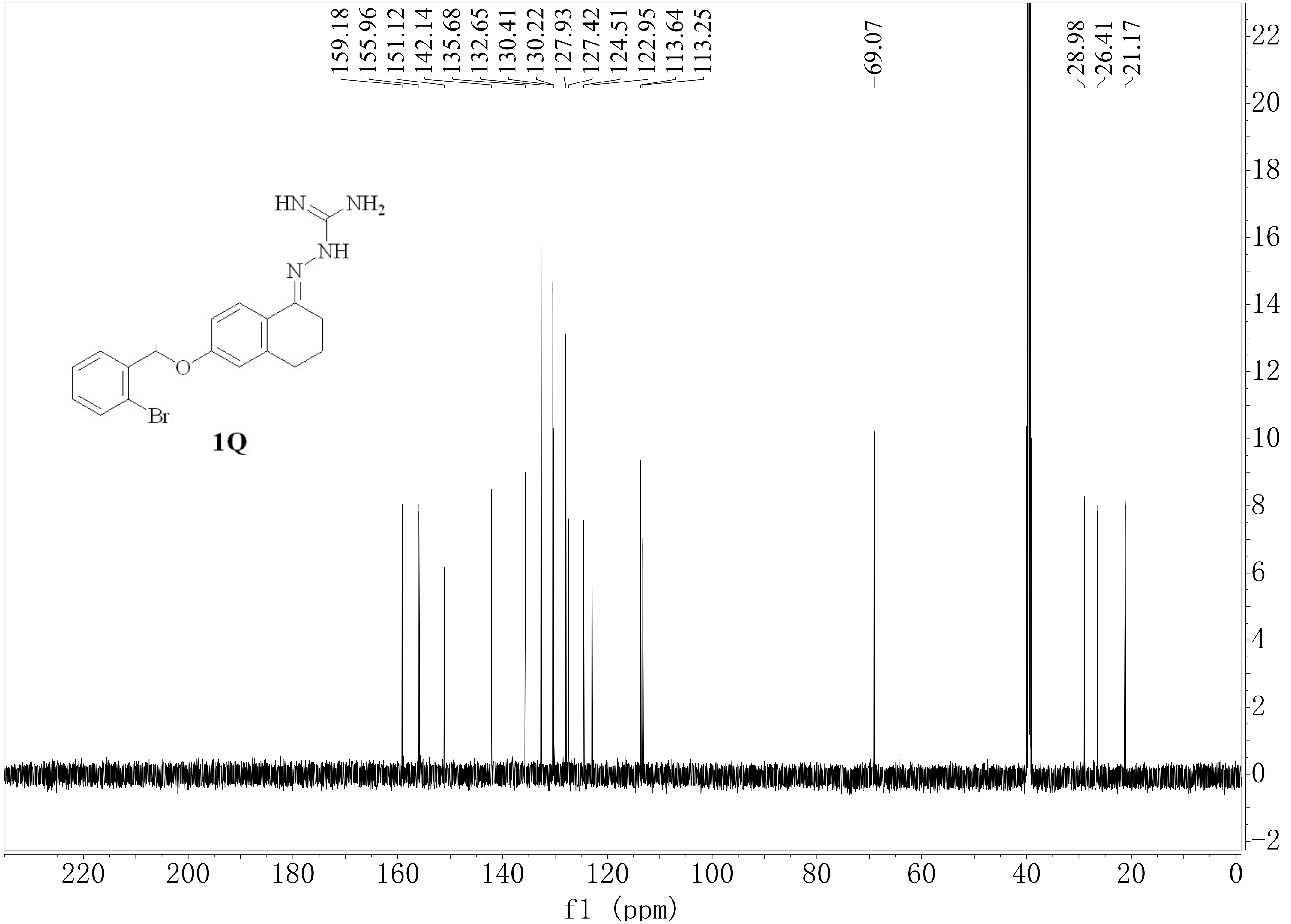
159.18
155.96
151.12
142.14
135.68
132.65
130.41
130.22
127.93
127.42
124.51
122.95
113.64
113.25
69.07
28.98
26.41
21.17
HN
NH2
NH
N
O
Br
1Q
f1 (ppm)

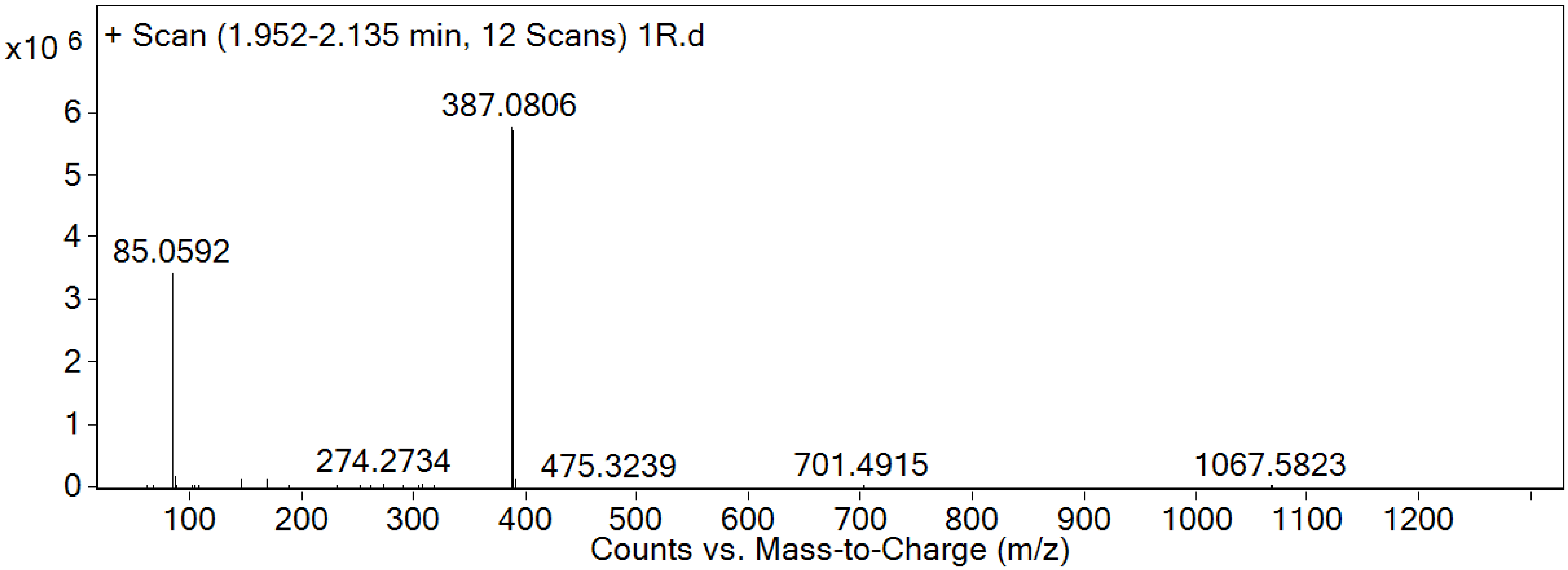
x10 6
+ Scan (1.952-2.135 min, 12 Scans) 1R.d
387.0806
85.0592
274.2734
475.3239
701.4915
1067.5823
0
1
2
3
4
5
6
100
200
300
400
500
600
700
800
900
1000
1100
1200
Counts vs. Mass-to-Charge (m/z)

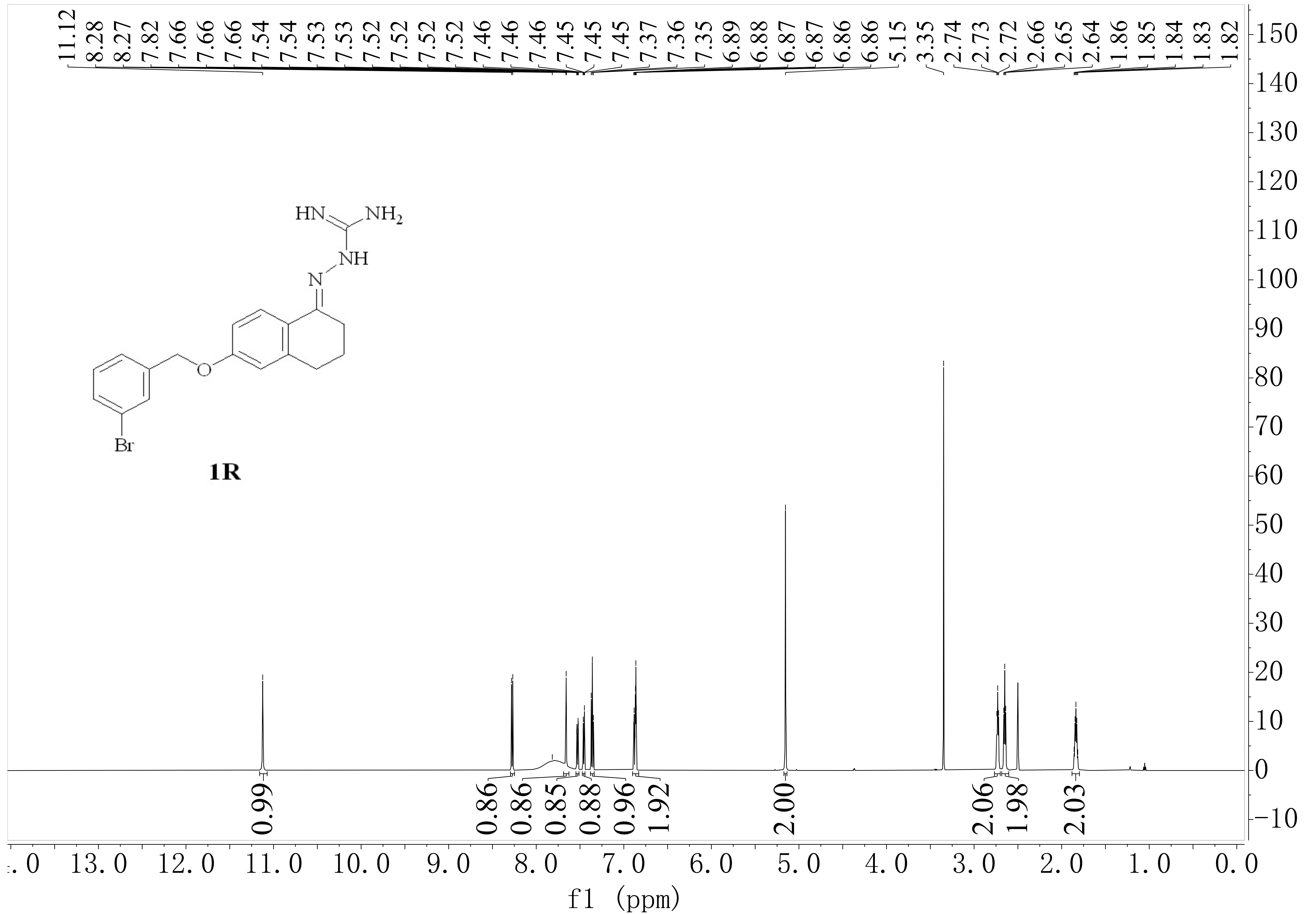

11.12 8.28 8.27 7.82 7.66 7.66 7.66 7.54 7.54 7.53 7.53 7.52 7.52 7.52 7.52 7.46 7.46 7.45 7.45 7.45 7.37 7.36 7.35 6.89 6.88 6.87 6.87 6.86 6.86 5.15 3.35 2.74 2.73 2.72 2.66 2.65 2.64 1.86 1.85 1.84 1.83 1.82
HN
NH2
NH
N
O
Br
1R
0.99
0.86
0.86
0.85
0.88
0.96
1.92
2.00
2.06
1.98
2.03
f1 (ppm)

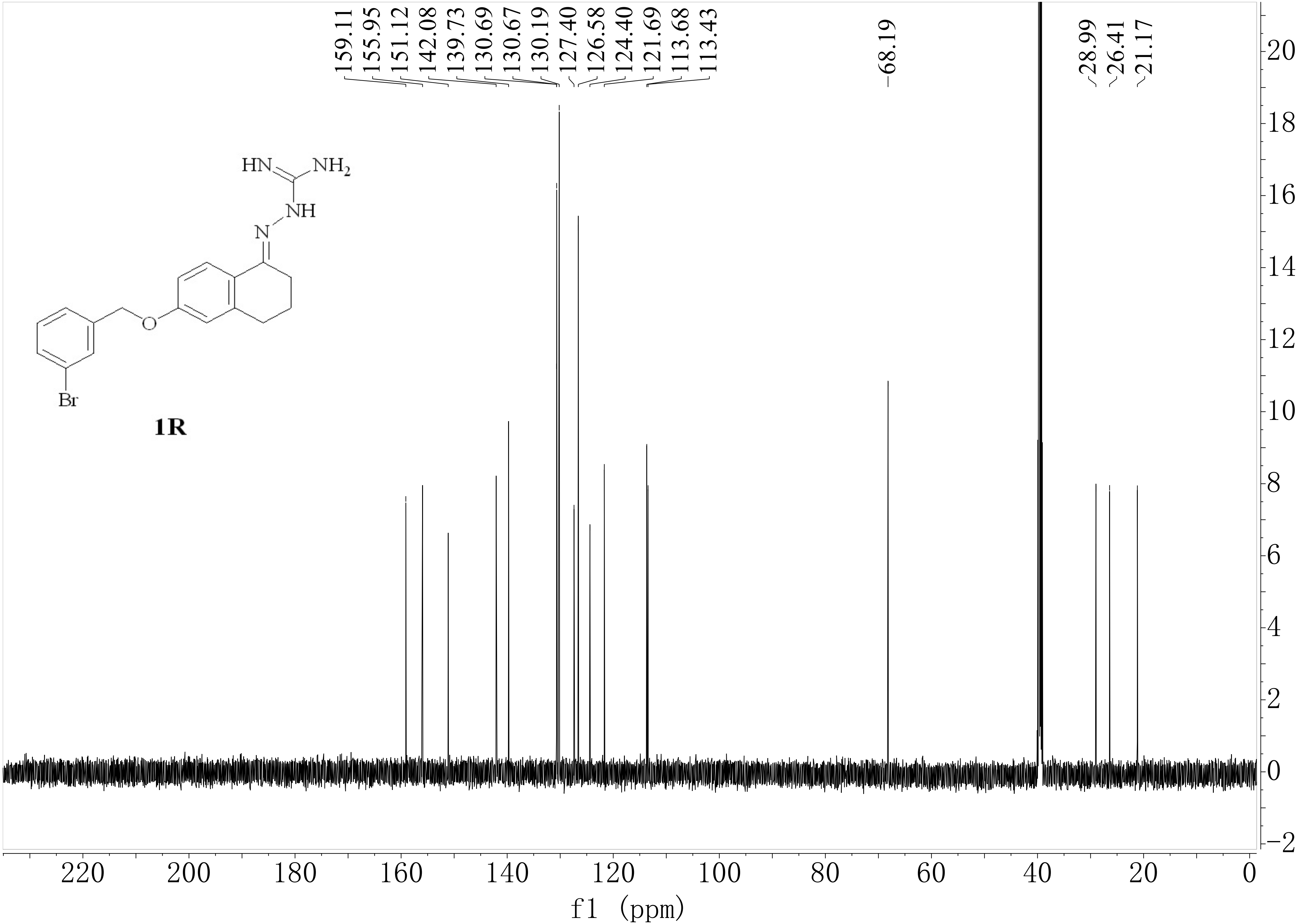

159.11
155.95
151.12
142.08
139.73
130.69
130.67
130.19
127.40
126.58
124.40
121.69
113.68
113.43
68.19
28.99
26.41
21.17
HN
NH2
NH
N
O
Br
1R
f1 (ppm)

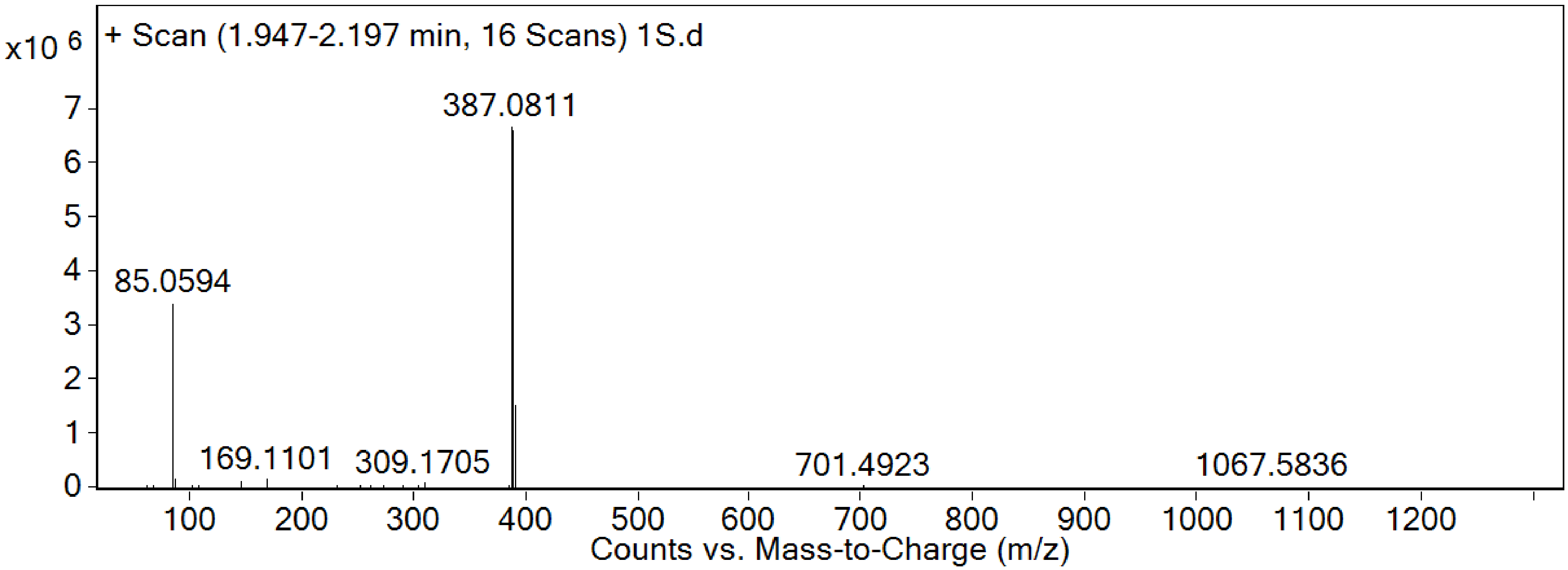
x10 6
+ Scan (1.947-2.197 min, 16 Scans) 1S.d
7
6
5
4
3
2
1
0
387.0811
85.0594
169.1101
309.1705
701.4923
1067.5836
100
200
300
400
500
600
700
800
900
1000
1100
1200
Counts vs. Mass-to-Charge (m/z)

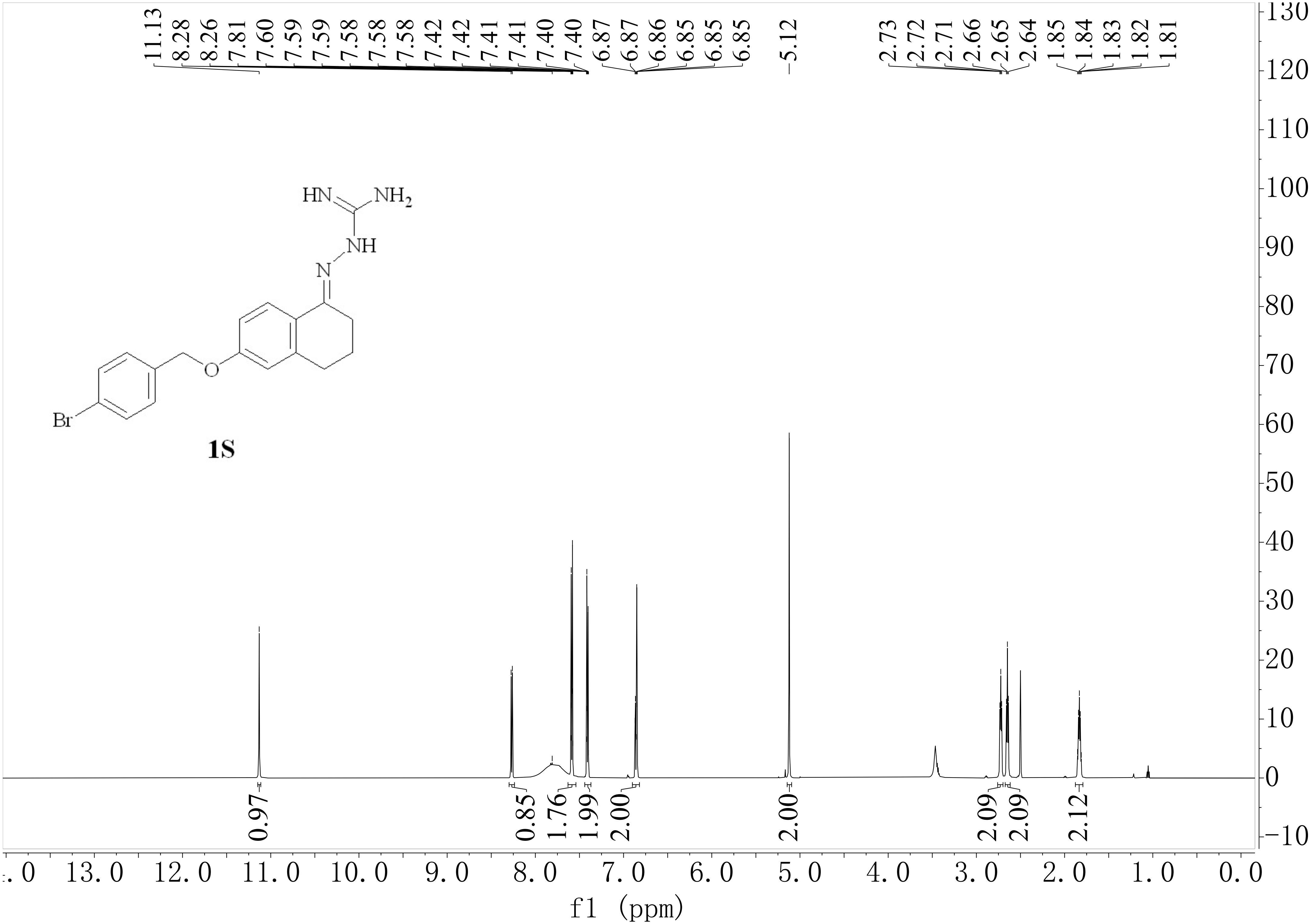
11.13 8.28 8.26 7.81 7.60 7.59 7.59 7.58 7.58 7.58 7.42 7.42 7.41 7.41 7.40 7.40 6.87 6.87 6.86 6.85 6.85 6.85
5.12
2.73 2.72 2.71 2.66 2.65 2.64 1.85 1.84 1.83 1.82 1.81
HN
NH2
NH
N
O
Br
1S
0.97
0.85
1.76
1.99
2.00
2.00
2.09
2.09
2.12
f1 (ppm)

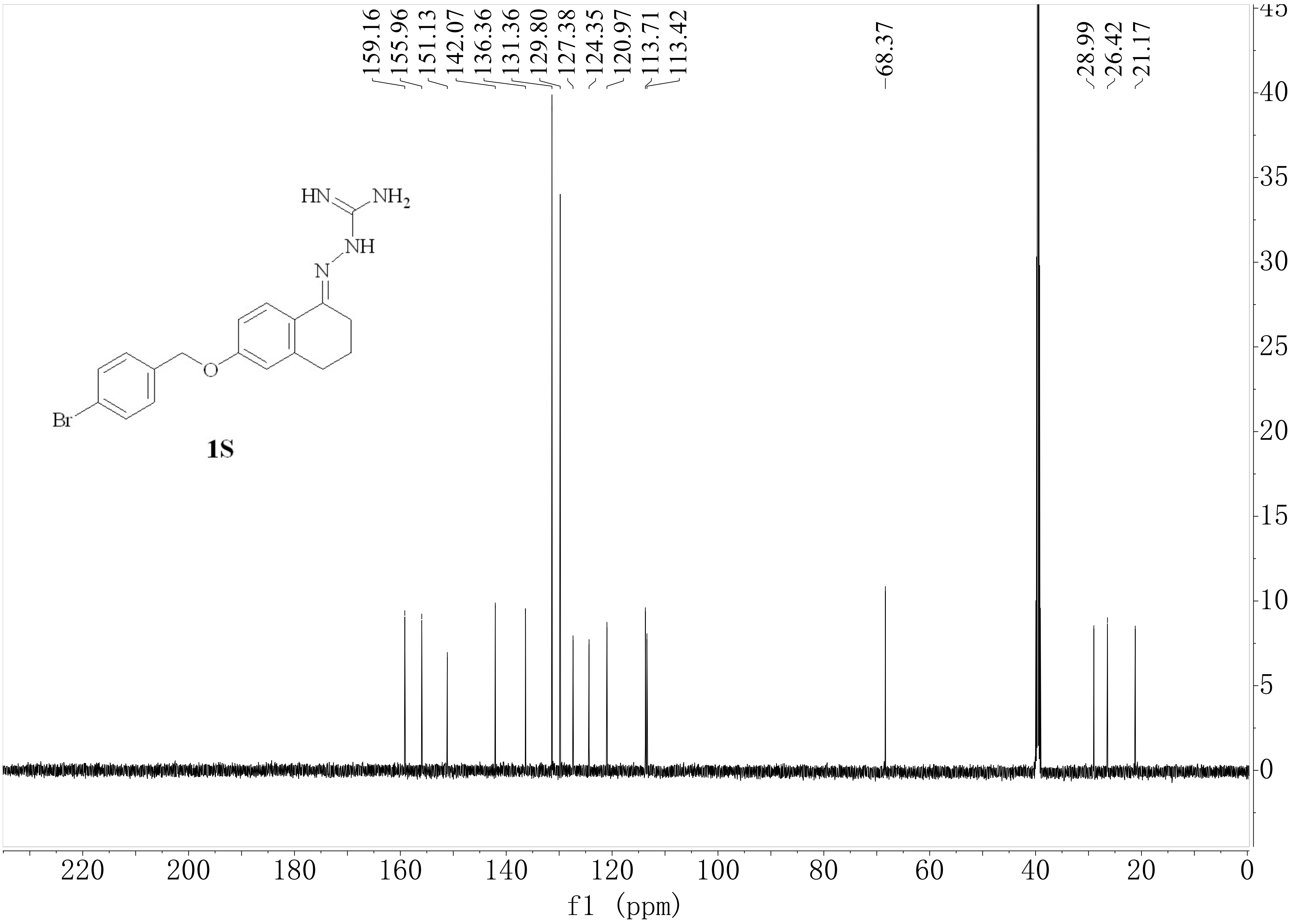

159.16
155.96
151.13
142.07
136.36
131.36
129.80
127.38
124.35
120.97
113.71
113.42
68.37
28.99
26.42
21.17
HN
NH2
NH
N
O
Br
1S
f1 (ppm)
220
200
180
160
140
120
100
80
60
40
20
0
45
40
35
30
25
20
15
10
5
0

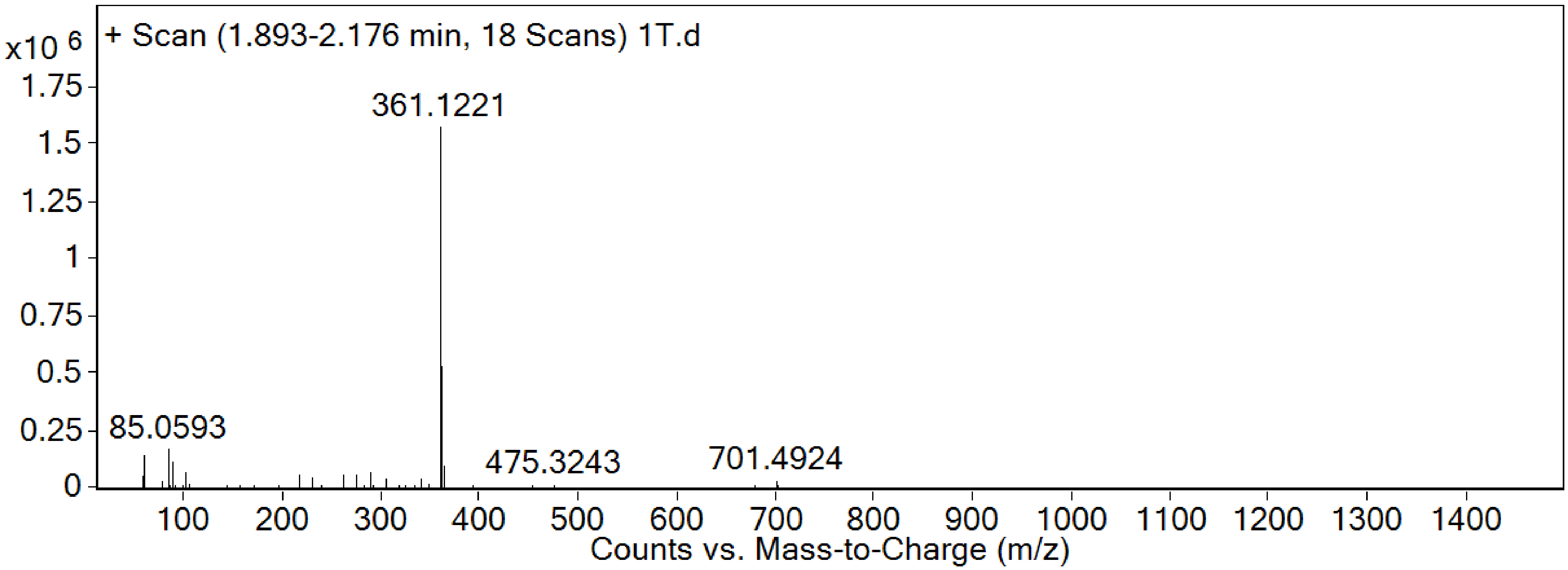
+ Scan (1.893-2.176 min, 18 Scans) 1T.d
x10 6
1.75
1.5
1.25
1
0.75
0.5
0.25
0
361.1221
85.0593
475.3243
701.4924
100
200
300
400
500
600
700
800
900
1000
1100
1200
1300
1400
Counts vs. Mass-to-Charge (m/z)

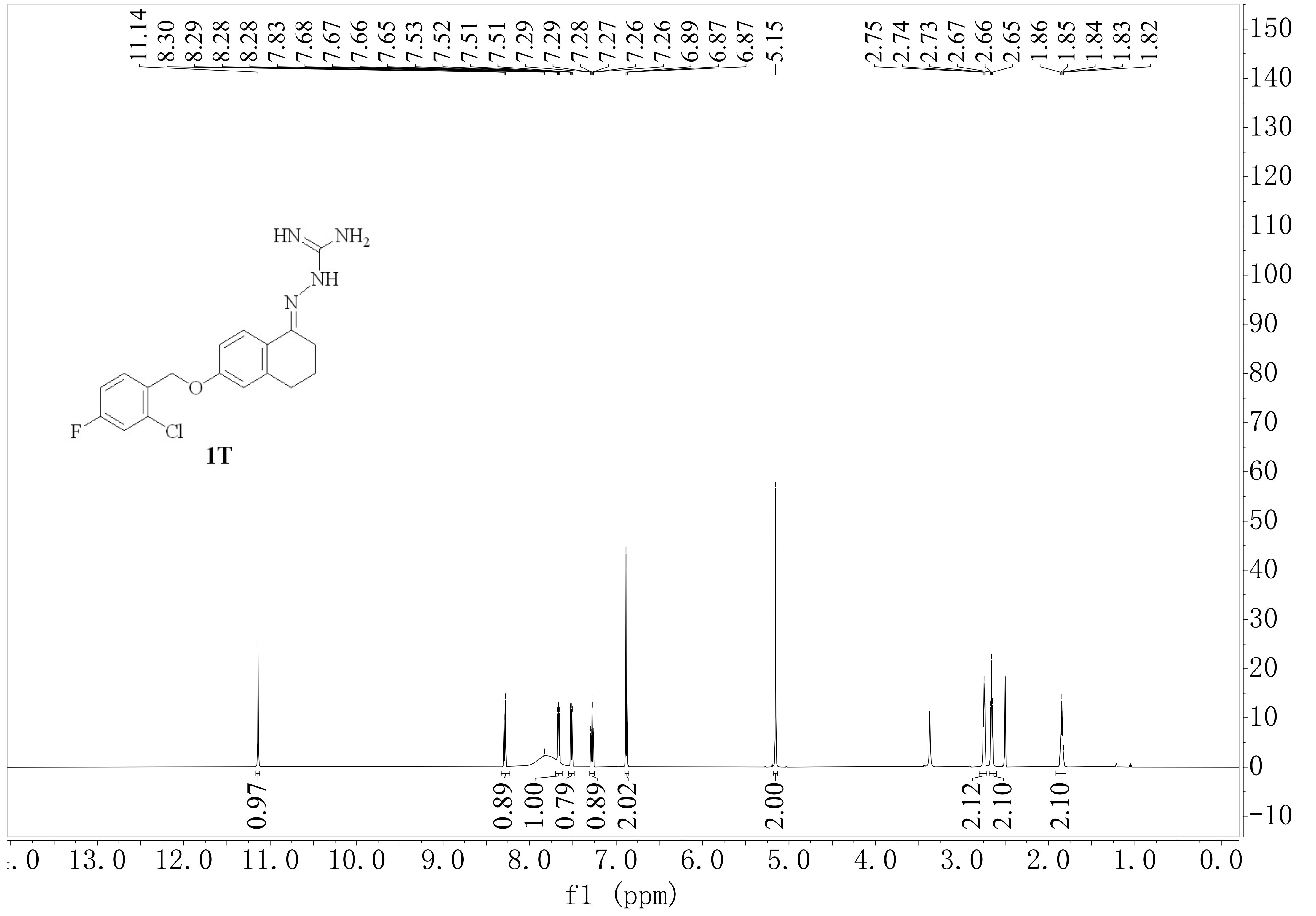
11.14 8.30 8.29 8.28 8.28 7.83 7.68 7.67 7.66 7.65 7.53 7.52 7.51 7.51 7.29 7.29 7.28 7.27 7.26 7.26 6.89 6.87 6.87
5.15
2.75 2.74 2.73 2.67 2.66 2.65 1.86 1.85 1.84 1.83 1.82
HN
NH2
NH
N
O
F
Cl
1T
0.97
0.89
1.00
0.79
0.89
2.02
2.00
2.12
2.10
2.10
13.0 12.0 11.0 10.0 9.0 8.0 7.0 6.0 5.0 4.0 3.0 2.0 1.0 0.0
f1 (ppm)
150 140 130 120 110 100 90 80 70 60 50 40 30 20 10 0 −10

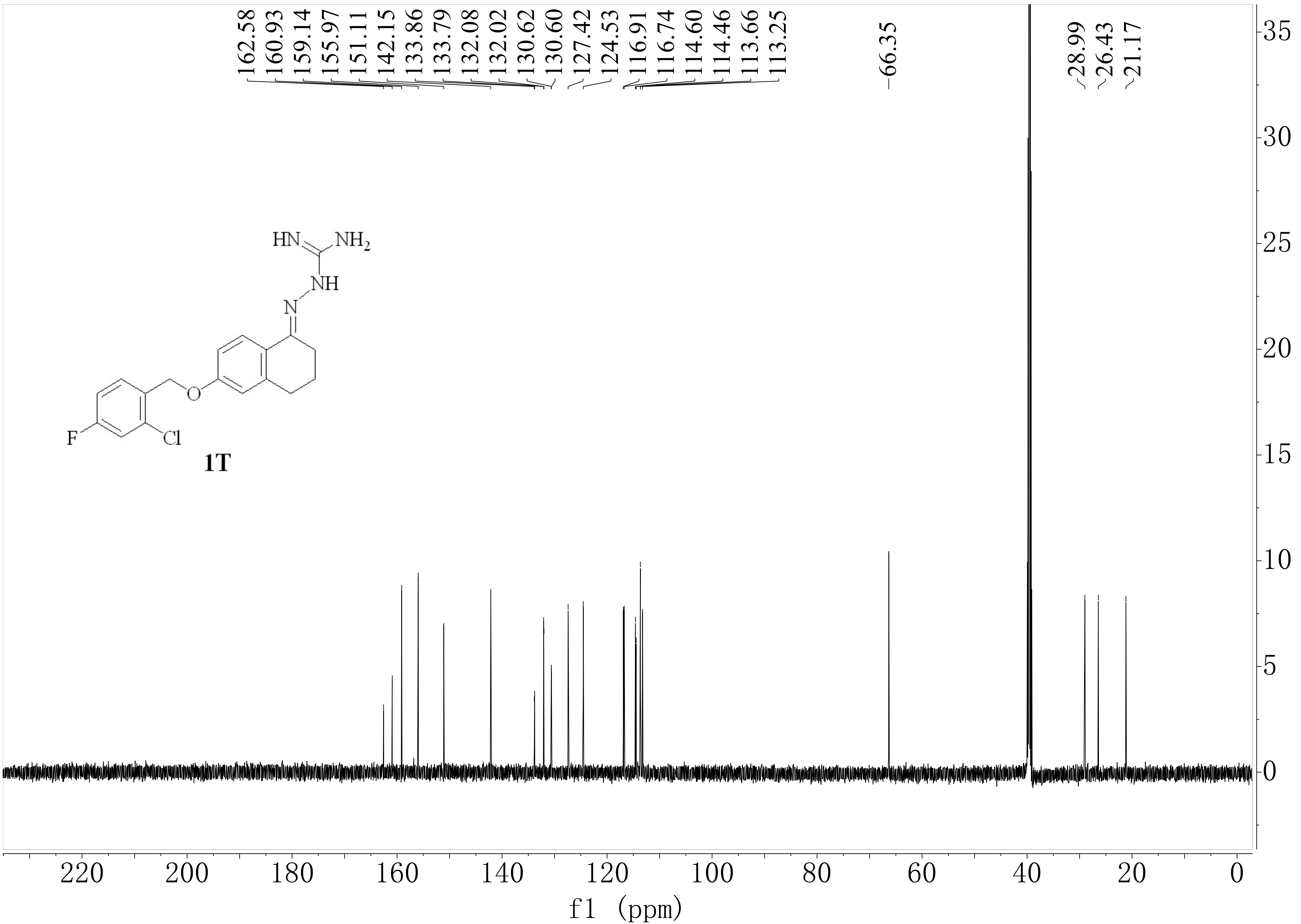

162.58
160.93
159.14
155.97
151.11
142.15
133.86
133.79
132.08
132.02
130.62
130.60
127.42
124.53
116.91
116.74
114.60
114.46
113.66
113.25
66.35
28.99
26.43
21.17
HN
NH2
NH
N
O
F
Cl
1T
220
200
180
160
140
120
100
80
60
40
20
0
f1 (ppm)
35
30
25
20
15
10
5
0

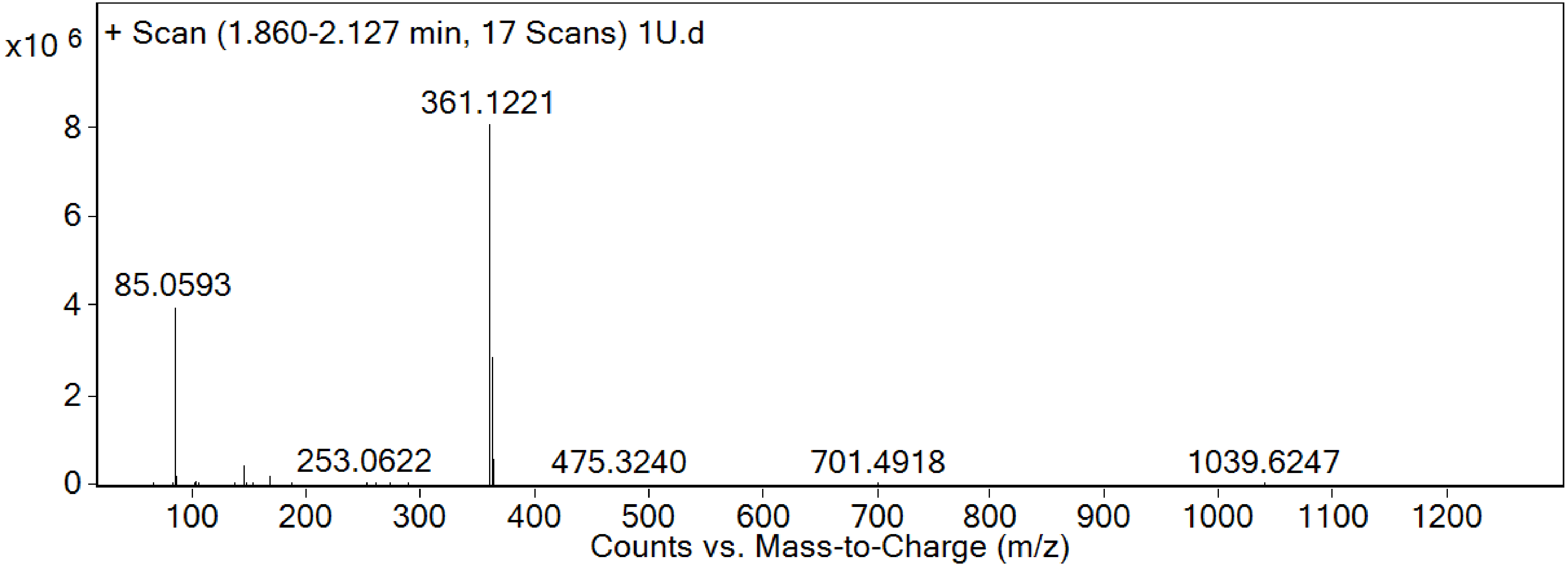
x10 6
+ Scan (1.860-2.127 min, 17 Scans) 1U.d
361.1221
85.0593
253.0622
475.3240
701.4918
1039.6247
0
2
4
6
8
100
200
300
400
500
600
700
800
900
1000
1100
1200
Counts vs. Mass-to-Charge (m/z)

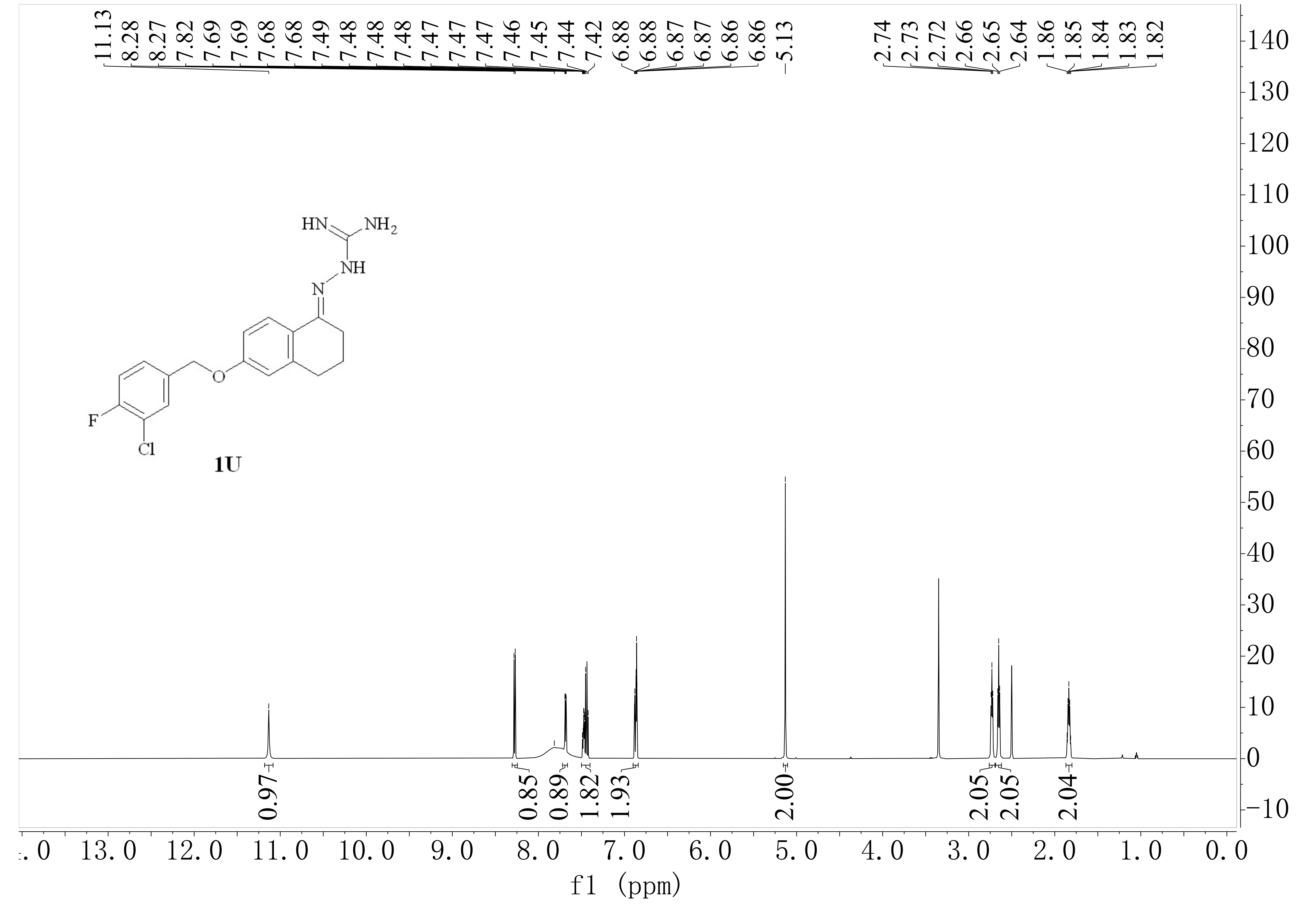

11.13
8.28
8.27
7.82
7.69
7.69
7.68
7.68
7.49
7.48
7.48
7.48
7.47
7.47
7.47
7.46
7.45
7.44
7.42
6.88
6.88
6.87
6.87
6.86
6.86
5.13
2.74
2.73
2.72
2.66
2.65
2.64
1.86
1.85
1.84
1.83
1.82
HN
NH2
NH
N
O
F
Cl
1U
0.97
0.85
0.89
1.82
1.93
2.00
2.05
2.05
2.04
f1 (ppm)

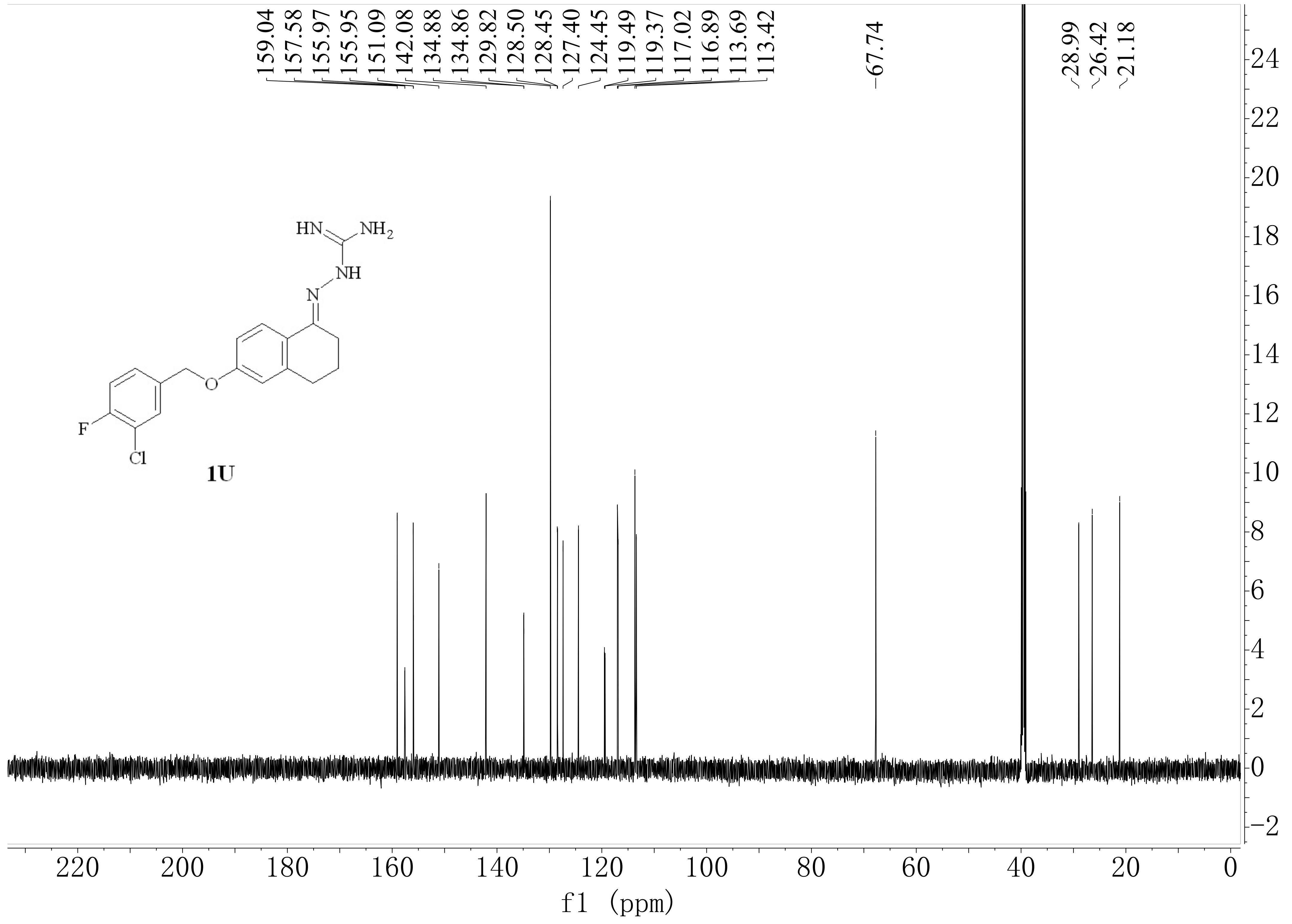
159.04
157.58
155.97
155.95
151.09
142.08
134.88
134.86
129.82
128.50
128.45
127.40
124.45
119.49
119.37
117.02
116.89
113.69
113.42
67.74
28.99
26.42
21.18
HN
NH2
NH
N
O
F
Cl
1U
f1 (ppm)

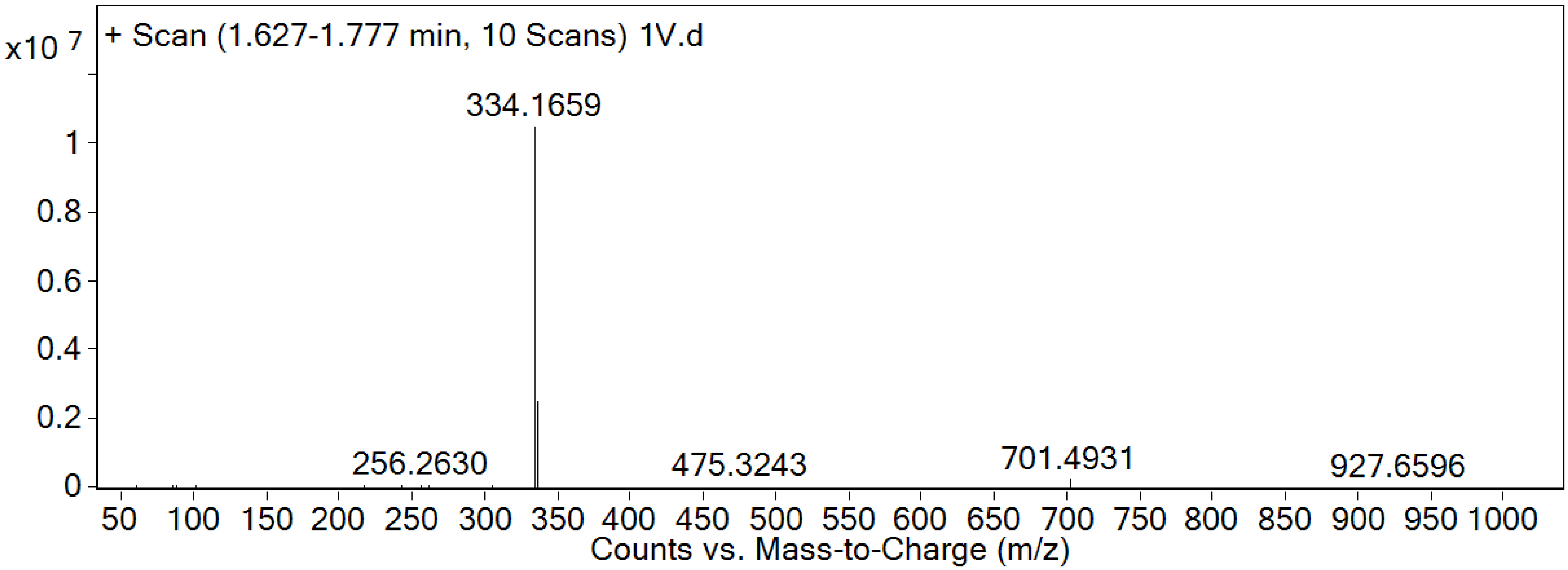
+ Scan (1.627-1.777 min, 10 Scans) 1V.d
x10 7
334.1659
256.2630
475.3243
701.4931
927.6596
0
0.2
0.4
0.6
0.8
1
50 100 150 200 250 300 350 400 450 500 550 600 650 700 750 800 850 900 950 1000
Counts vs. Mass-to-Charge (m/z)

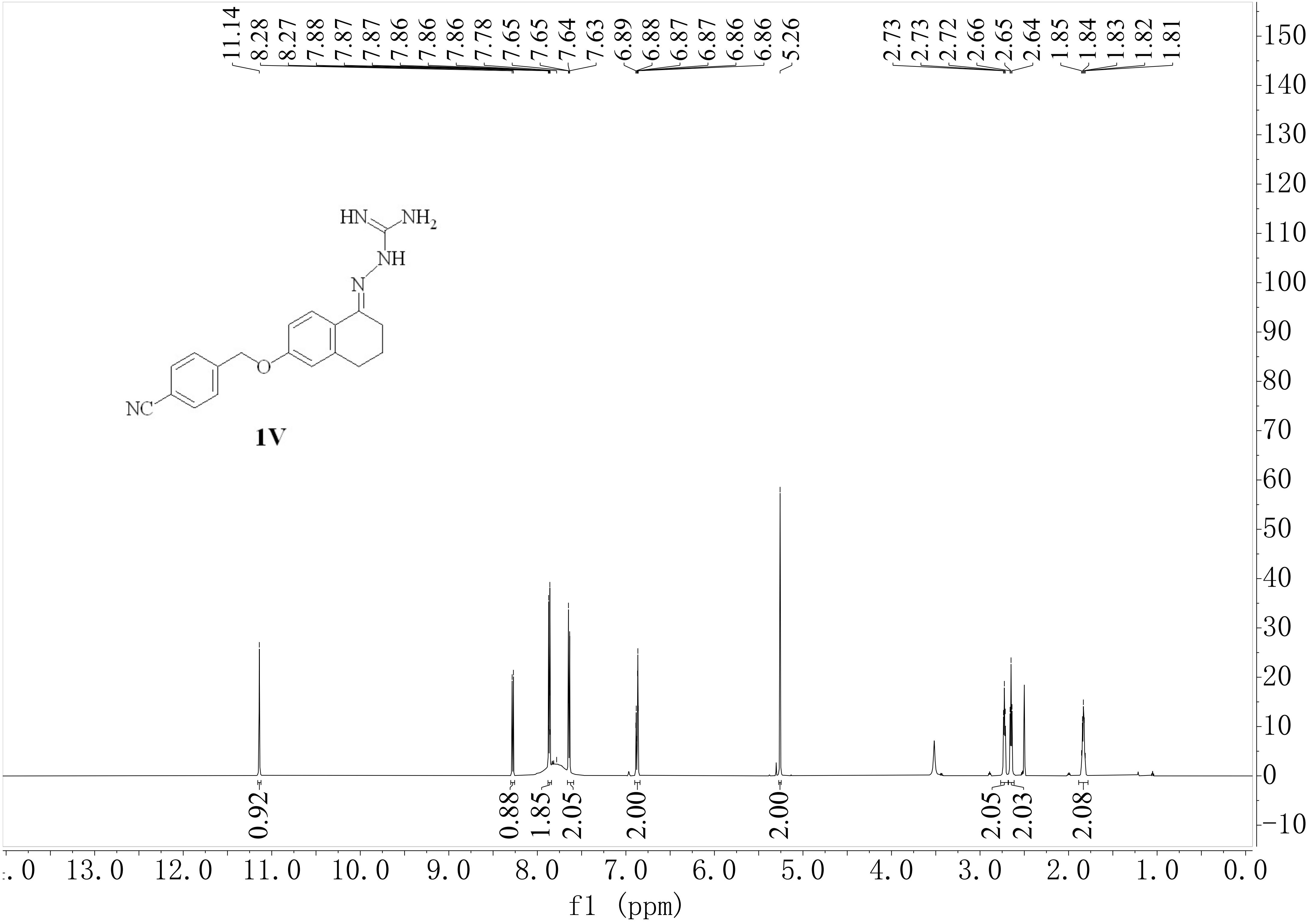

11.14
8.28
8.27
7.88
7.87
7.87
7.86
7.86
7.86
7.78
7.65
7.65
7.64
7.63
6.89
6.88
6.87
6.87
6.86
6.86
5.26
2.73
2.73
2.72
2.66
2.65
2.64
1.85
1.84
1.83
1.82
1.81
HN
NH2
NH
N
O
NC
1V
0.92
0.88
1.85
2.05
2.00
2.00
2.05
2.03
2.08
f1 (ppm)

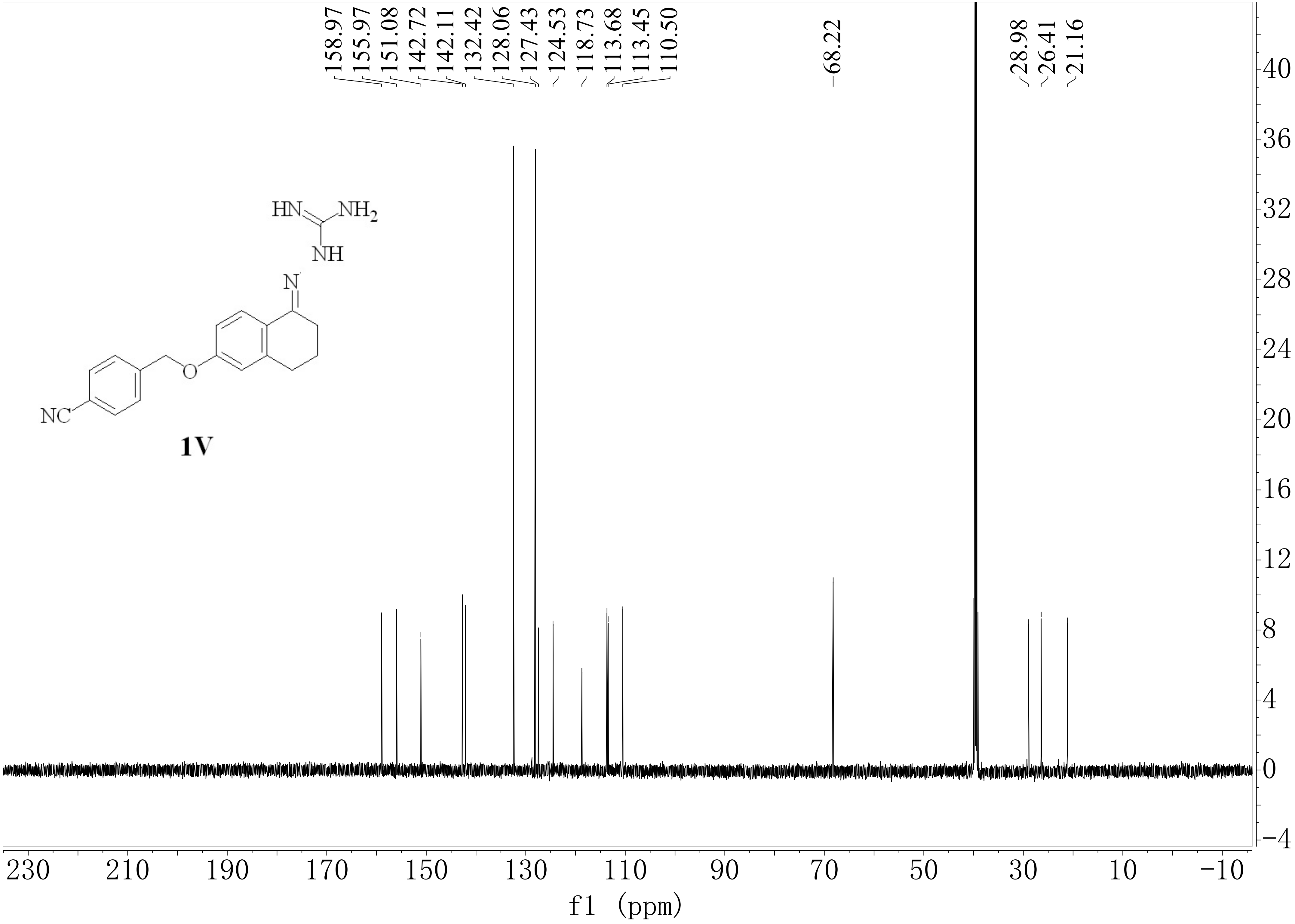

158.97
155.97
151.08
142.72
142.11
132.42
128.06
127.43
124.53
118.73
113.68
113.45
110.50
68.22
28.98
26.41
21.16
HN
NH2
NH
N
O
NC
1V
f1 (ppm)

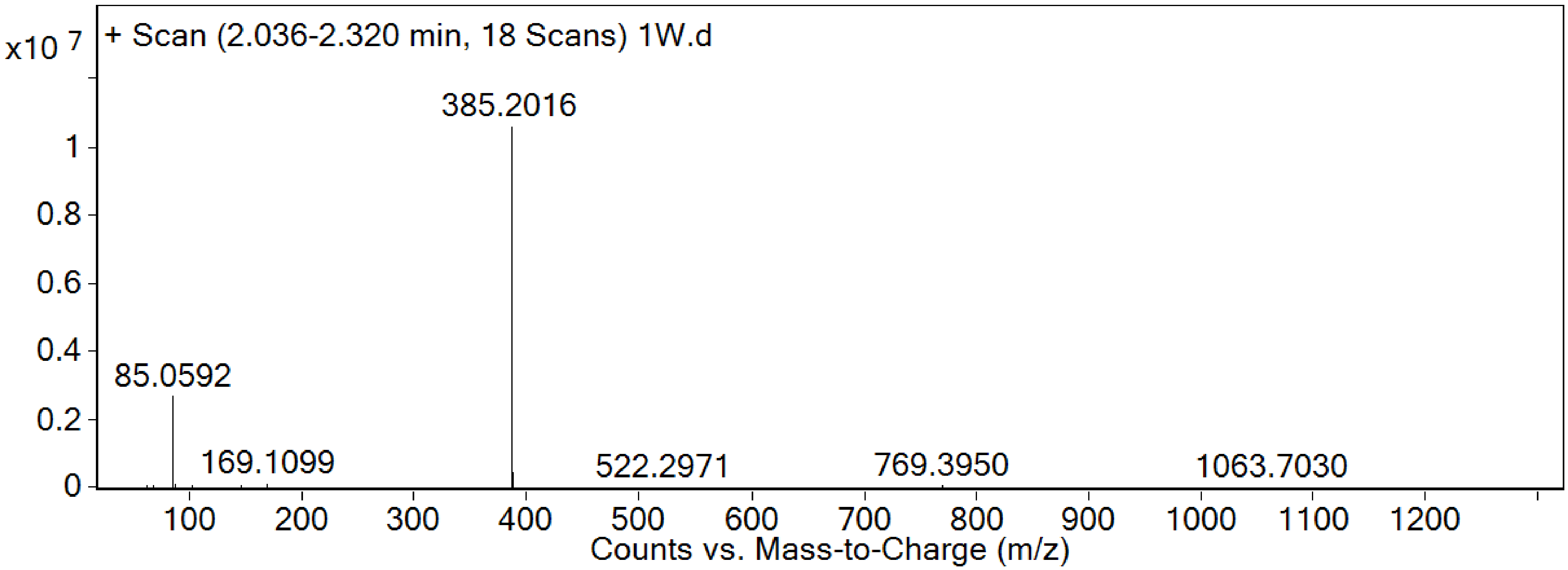

x10 7
+ Scan (2.036-2.320 min, 18 Scans) 1W.d
385.2016
85.0592
169.1099
522.2971
769.3950
1063.7030
1
0.8
0.6
0.4
0.2
0
100
200
300
400
500
600
700
800
900
1000
1100
1200
Counts vs. Mass-to-Charge (m/z)

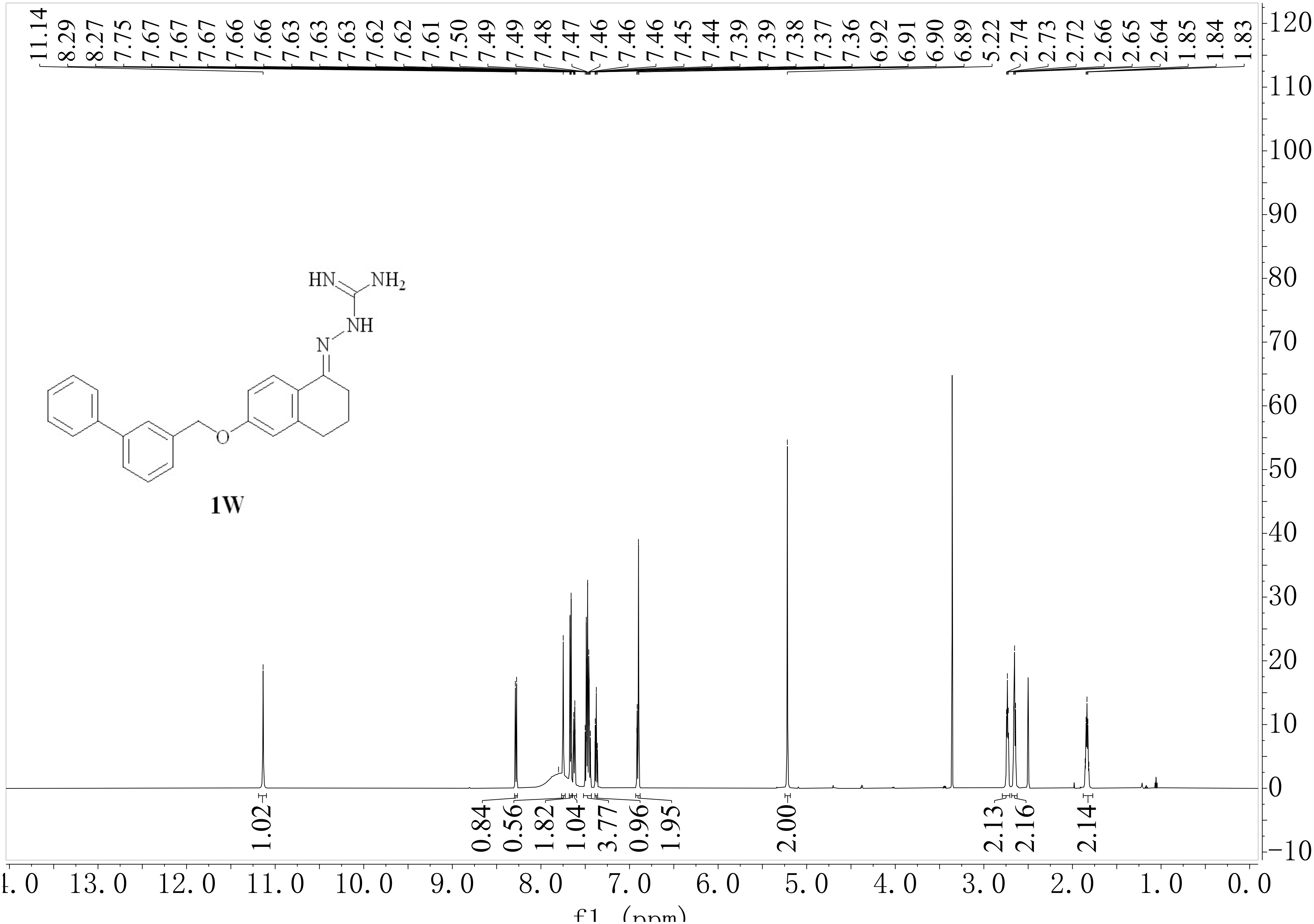
11.14 8.29 8.27 7.75 7.67 7.67 7.67 7.66 7.66 7.63 7.63 7.63 7.62 7.62 7.61 7.50 7.49 7.49 7.48 7.47 7.46 7.46 7.46 7.45 7.44 7.39 7.39 7.38 7.37 7.36 6.92 6.91 6.90 6.89 5.22 2.74 2.73 2.72 2.66 2.65 2.64 1.85 1.84 1.83
HN NH2 NH N O
1W
1.02 0.84 0.56 1.82 1.04 3.77 0.96 1.95 2.00 2.13 2.16 2.14
120 110 100 90 80 70 60 50 40 30 20 10 0 −10
13.0 12.0 11.0 10.0 9.0 8.0 7.0 6.0 5.0 4.0 3.0 2.0 1.0 0.0
f1 (ppm)

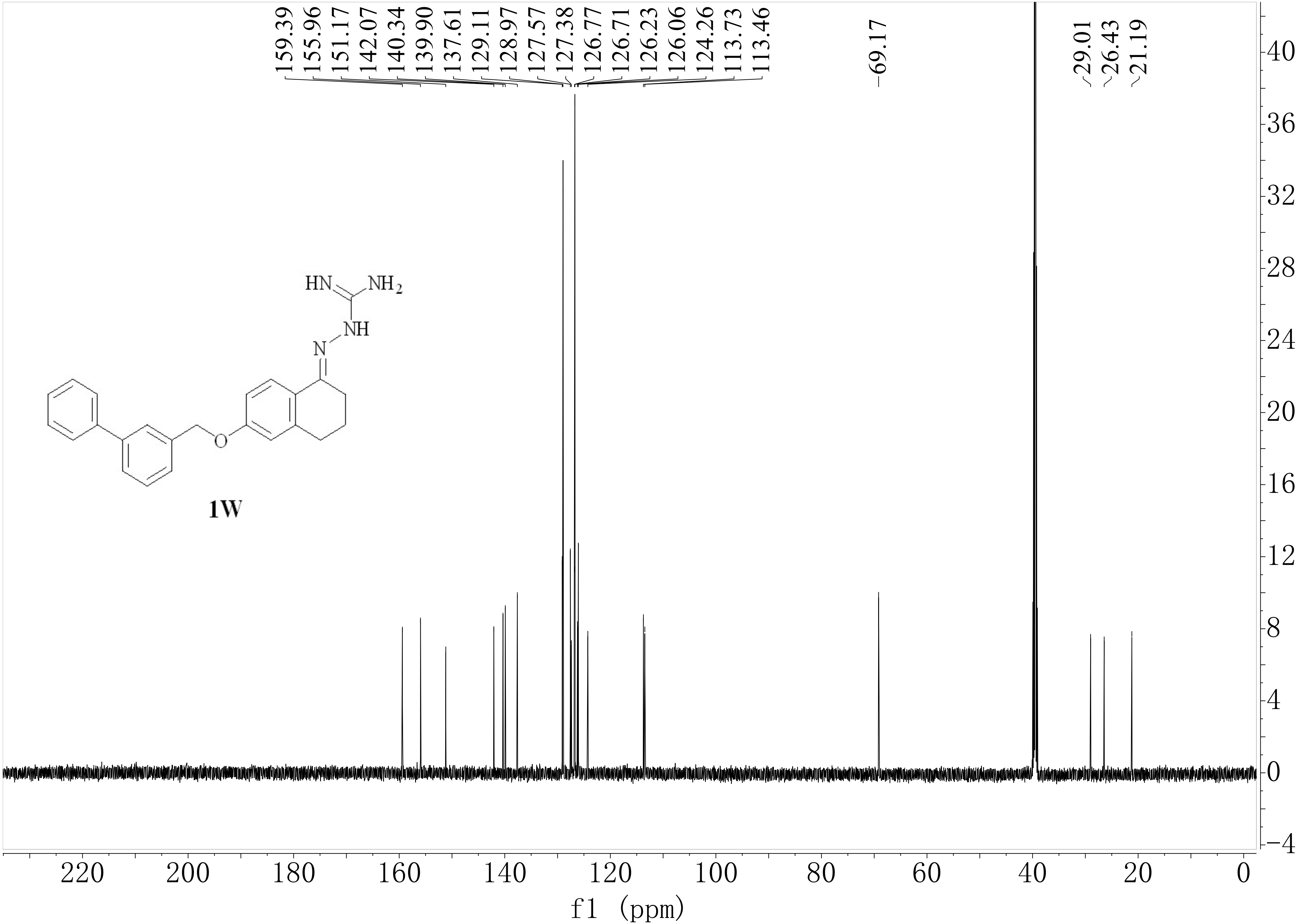
159.39
155.96
151.17
142.07
140.34
139.90
137.61
129.11
128.97
127.57
127.38
126.77
126.71
126.23
126.06
124.26
113.73
113.46
69.17
29.01
26.43
21.19
HN
NH2
NH
N
O
1W
220
200
180
160
140
120
100
80
60
40
20
0
f1 (ppm)
40
36
32
28
24
20
16
12
8
4
0
−4

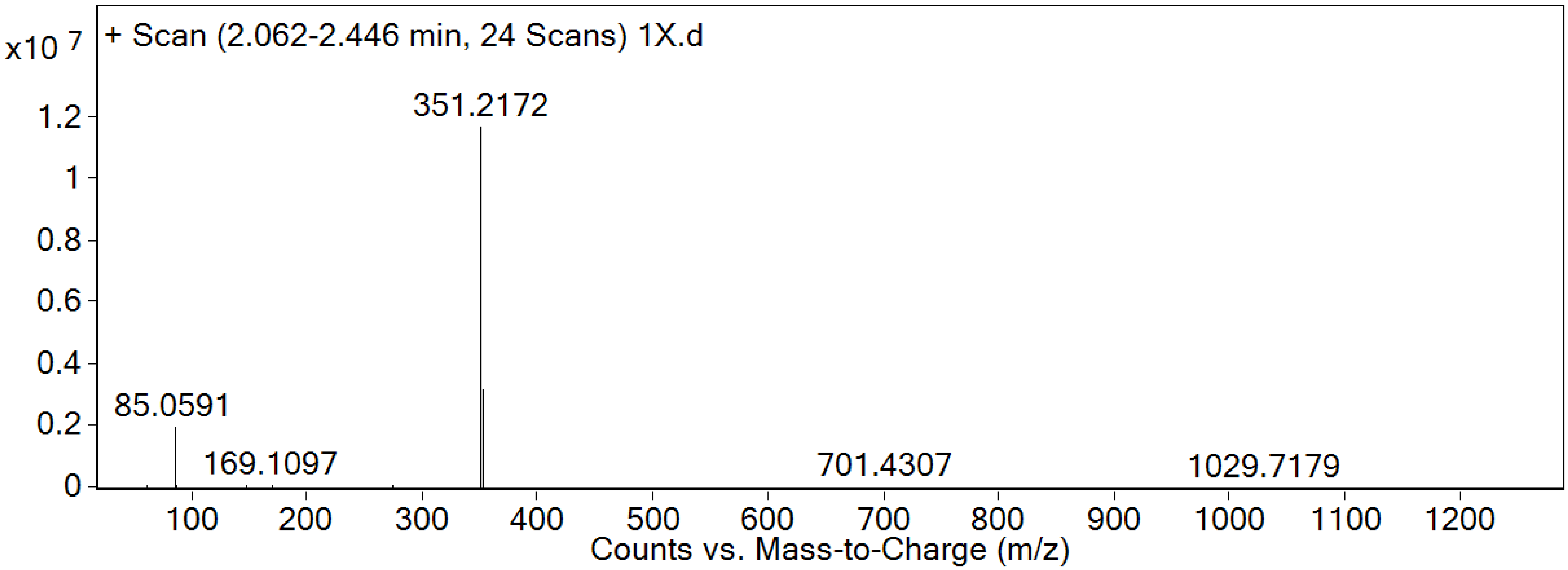
x10 7
+ Scan (2.062-2.446 min, 24 Scans) 1X.d
351.2172
85.0591
169.1097
701.4307
1029.7179
1.2
1
0.8
0.6
0.4
0.2
0
100
200
300
400
500
600
700
800
900
1000
1100
1200
Counts vs. Mass-to-Charge (m/z)

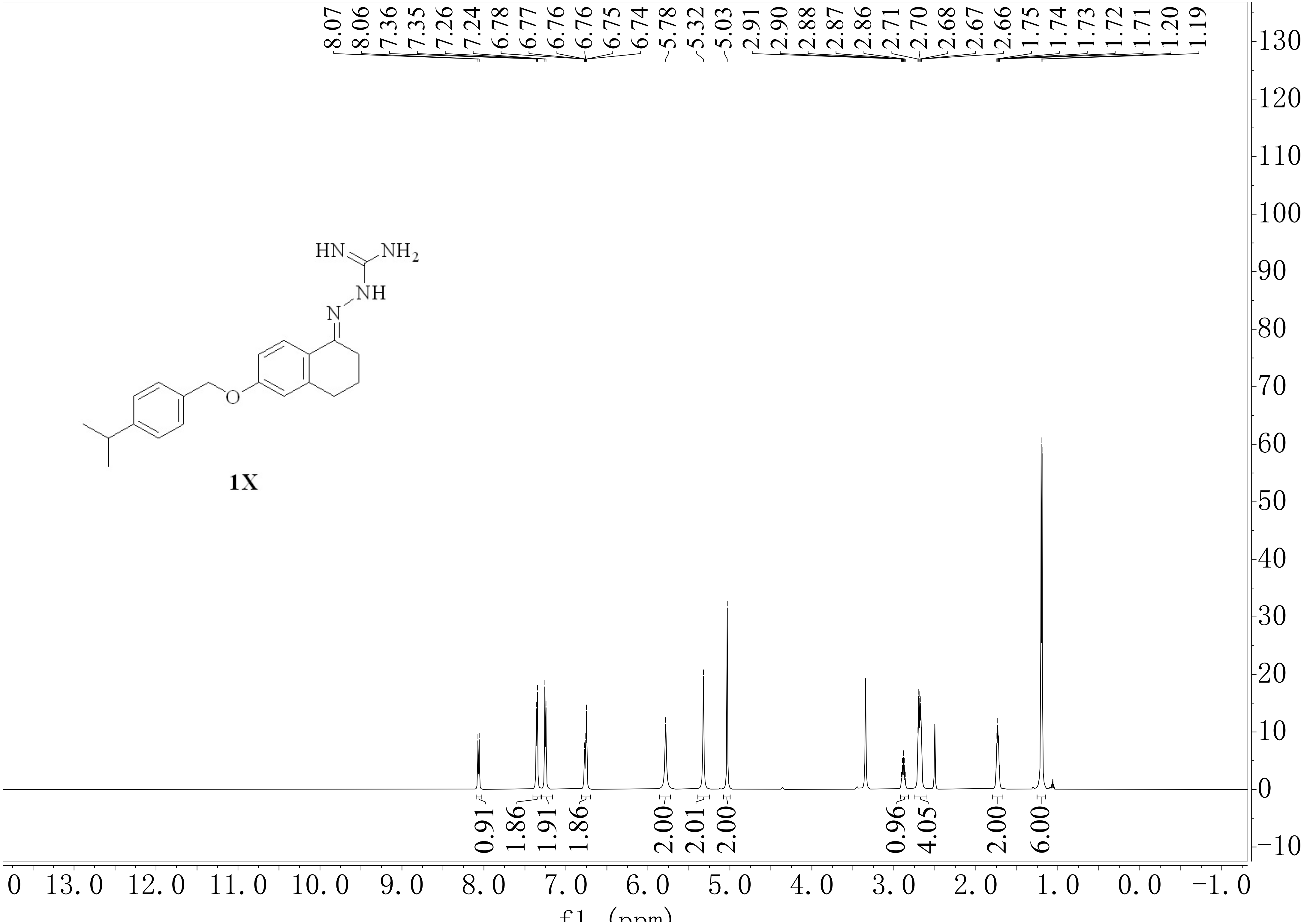
8.07 8.06 7.36 7.35 7.26 7.24 6.78 6.77 6.76 6.76 6.75 6.74 5.78 5.32 5.03 2.91 2.90 2.88 2.87 2.86 2.71 2.70 2.68 2.67 2.66 1.75 1.74 1.73 1.72 1.71 1.20 1.19
HN
NH2
NH
N
O
1X
0.91 1.86 1.91 1.86 2.00 2.01 2.00 0.96 4.05 2.00 6.00
f1 (ppm)

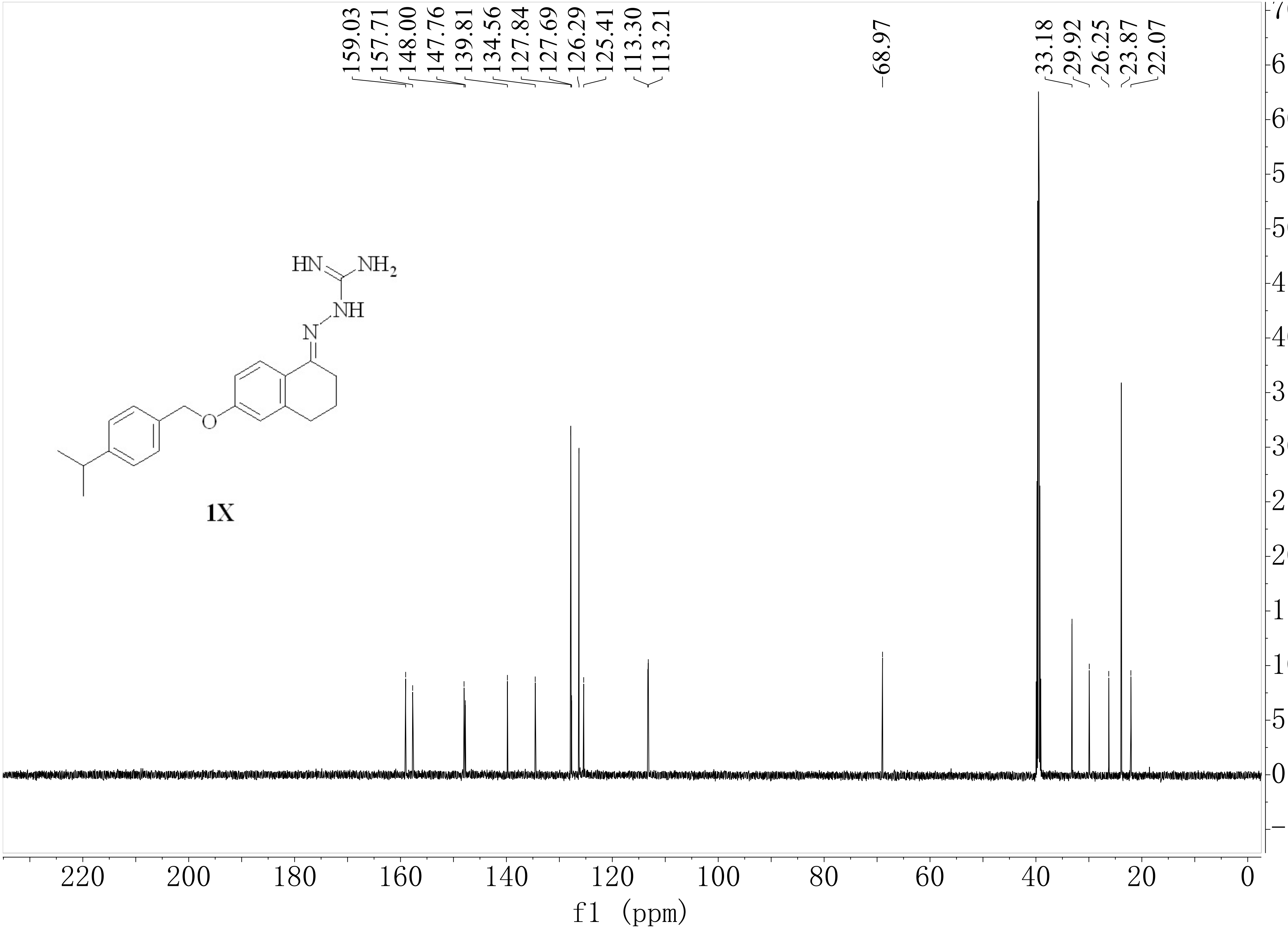
159.03
157.71
148.00
147.76
139.81
134.56
127.84
127.69
126.29
125.41
113.30
113.21
68.97
33.18
29.92
26.25
23.87
22.07
HN
NH2
NH
N
O
1X
f1 (ppm)

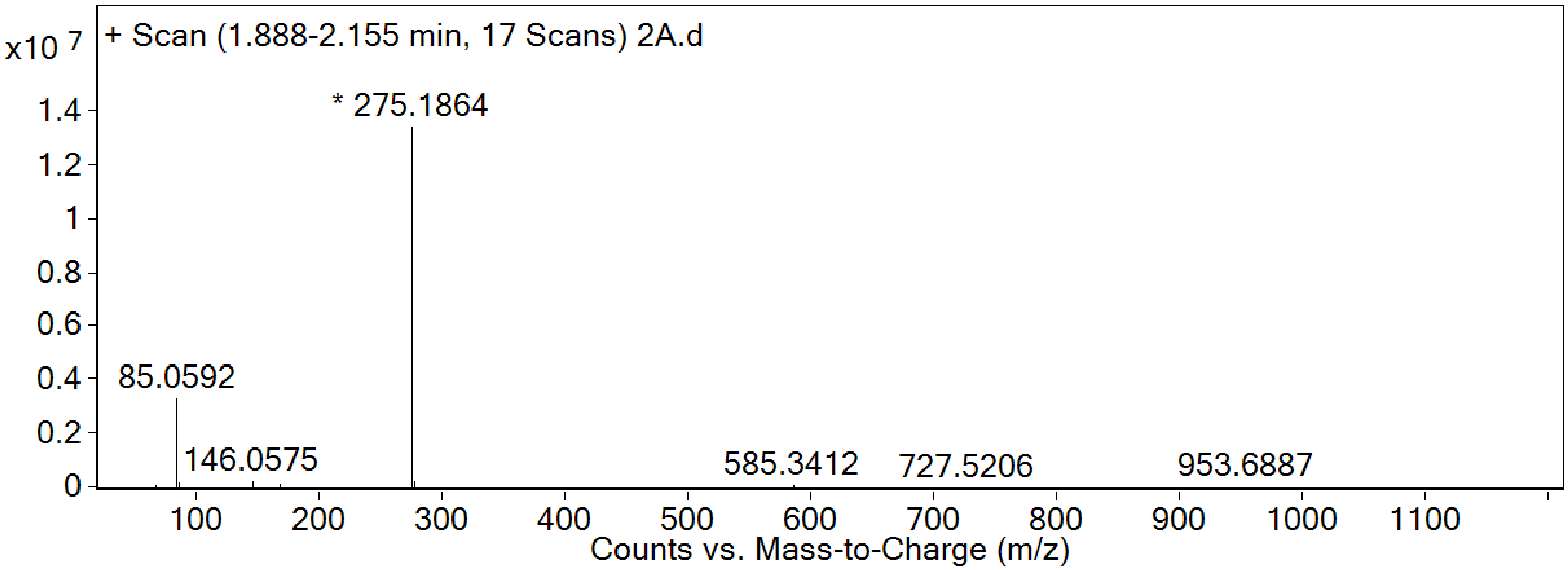

x10 7
+ Scan (1.888-2.155 min, 17 Scans) 2A.d
* 275.1864
85.0592
146.0575
585.3412
727.5206
953.6887
1.4
1.2
1
0.8
0.6
0.4
0.2
0
100
200
300
400
500
600
700
800
900
1000
1100
Counts vs. Mass-to-Charge (m/z)

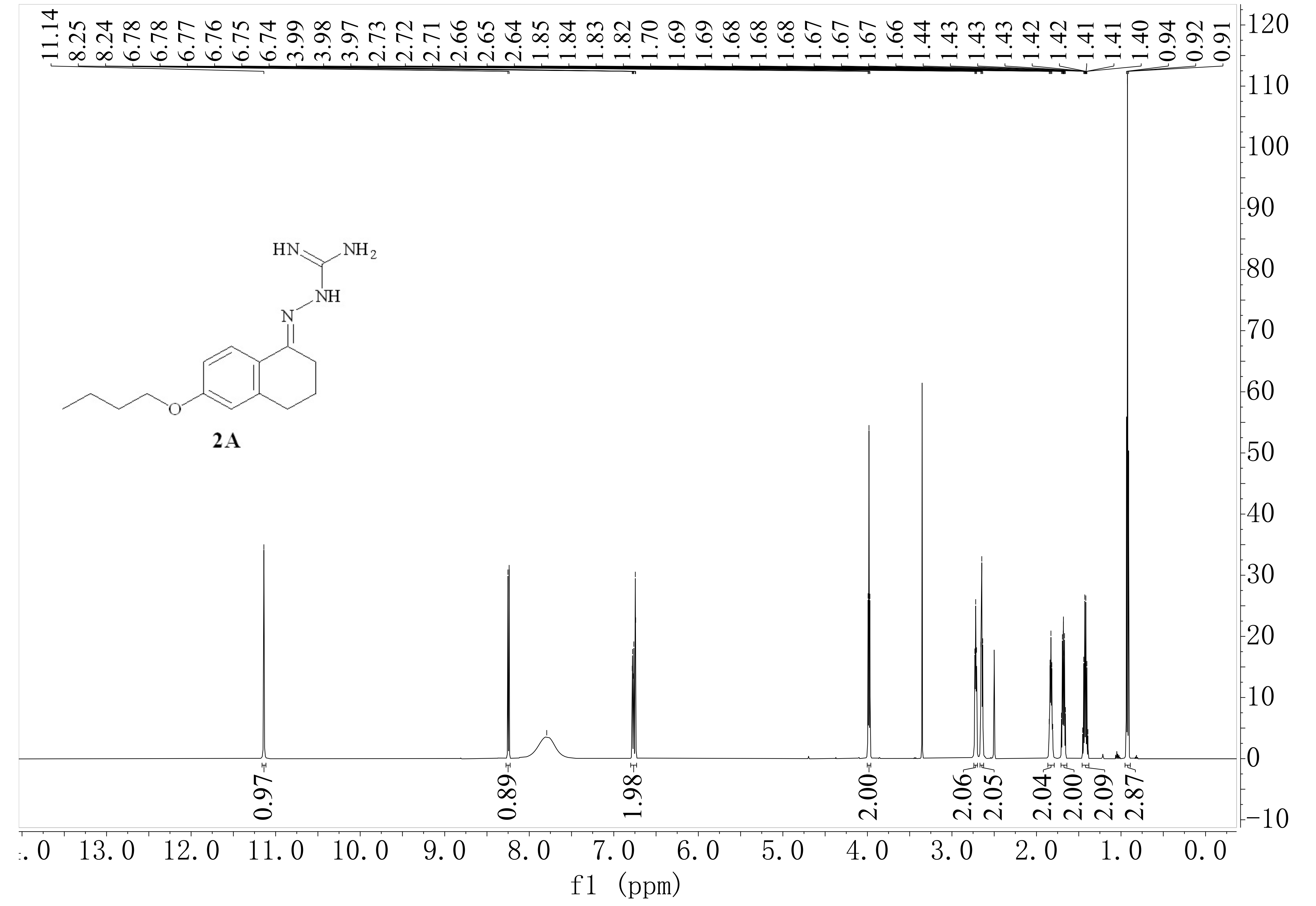

11.14
8.25
8.24
6.78
6.78
6.77
6.76
6.75
6.74
3.99
3.98
3.97
2.73
2.72
2.71
2.66
2.65
2.64
1.85
1.84
1.83
1.82
1.70
1.69
1.69
1.68
1.68
1.68
1.67
1.67
1.67
1.66
1.44
1.43
1.43
1.43
1.42
1.42
1.41
1.41
1.40
0.94
0.92
0.91
HN
NH2
NH
N
O
2A
0.97
0.89
1.98
2.00
2.06
2.05
2.04
2.00
2.09
2.87
13.0
12.0
11.0
10.0
9.0
8.0
7.0
6.0
5.0
4.0
3.0
2.0
1.0
0.0
f1 (ppm)
120
110
100
90
80
70
60
50
40
30
20
10
0
−10

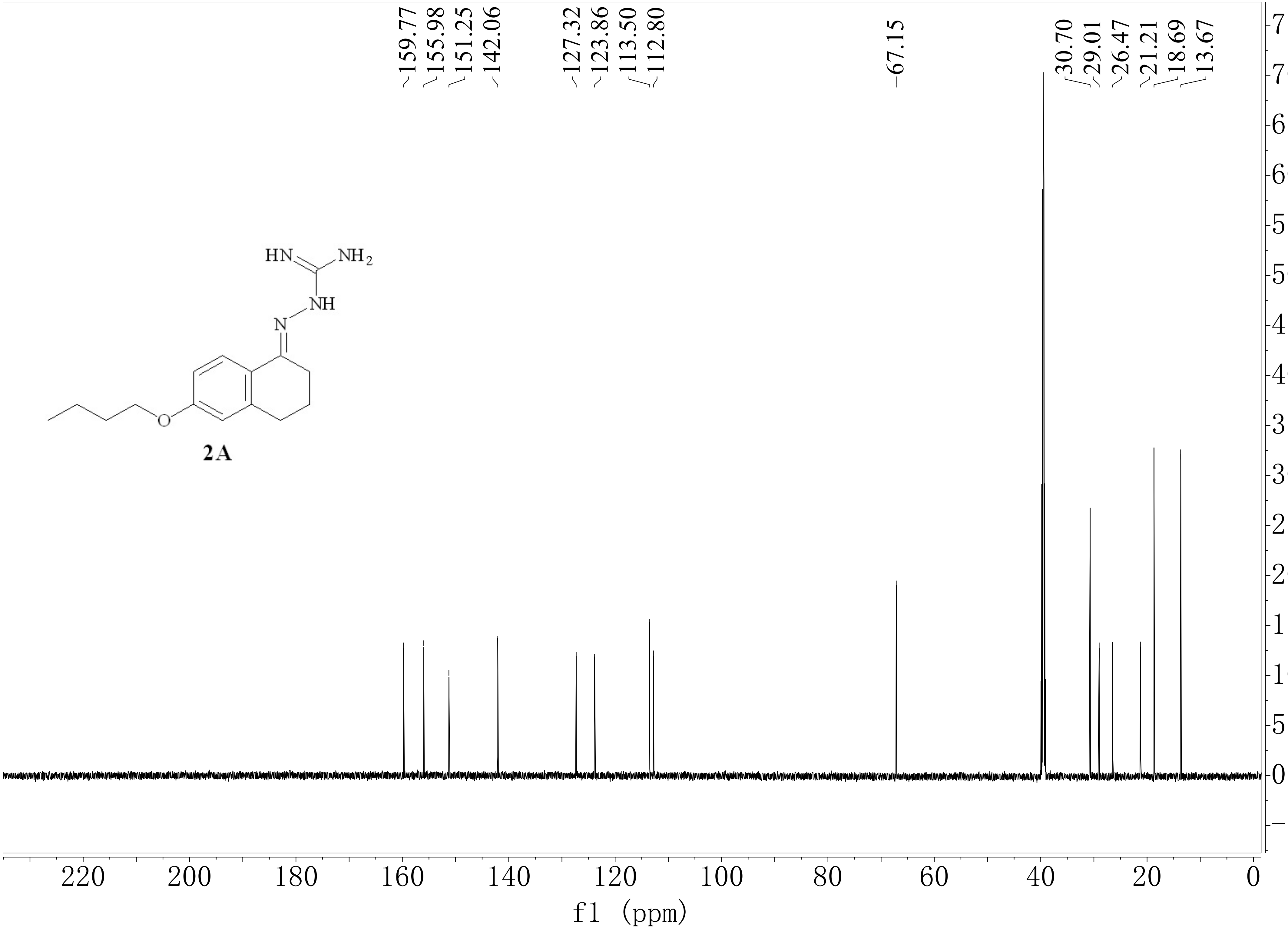
159.77
155.98
151.25
142.06
127.32
123.86
113.50
112.80
67.15
30.70
29.01
26.47
21.21
18.69
13.67
HN
NH2
NH
N
O
2A
220 200 180 160 140 120 100 80 60 40 20 0
f1 (ppm)
75 70 65 60 55 50 45 40 35 30 25 20 15 10 5 0 −5

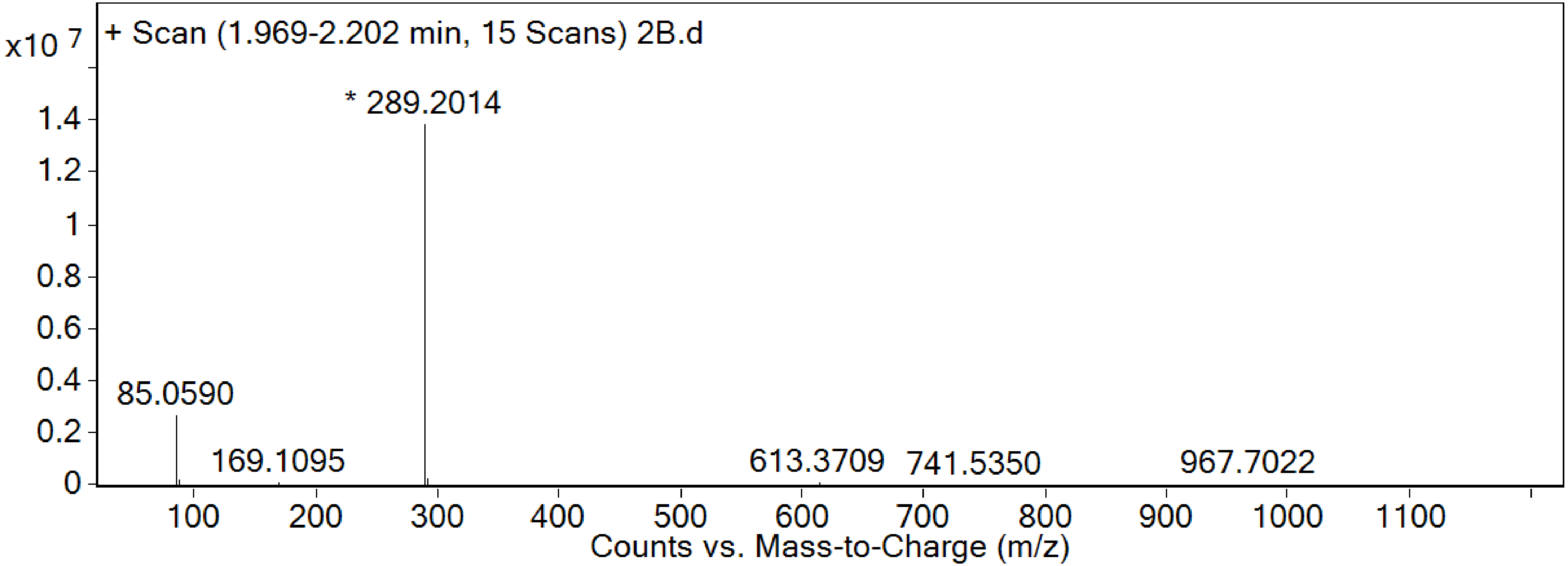
x10 7
+ Scan (1.969-2.202 min, 15 Scans) 2B.d
1.4
1.2
1
0.8
0.6
0.4
0.2
0
* 289.2014
85.0590
169.1095
613.3709
741.5350
967.7022
100
200
300
400
500
600
700
800
900
1000
1100
Counts vs. Mass-to-Charge (m/z)

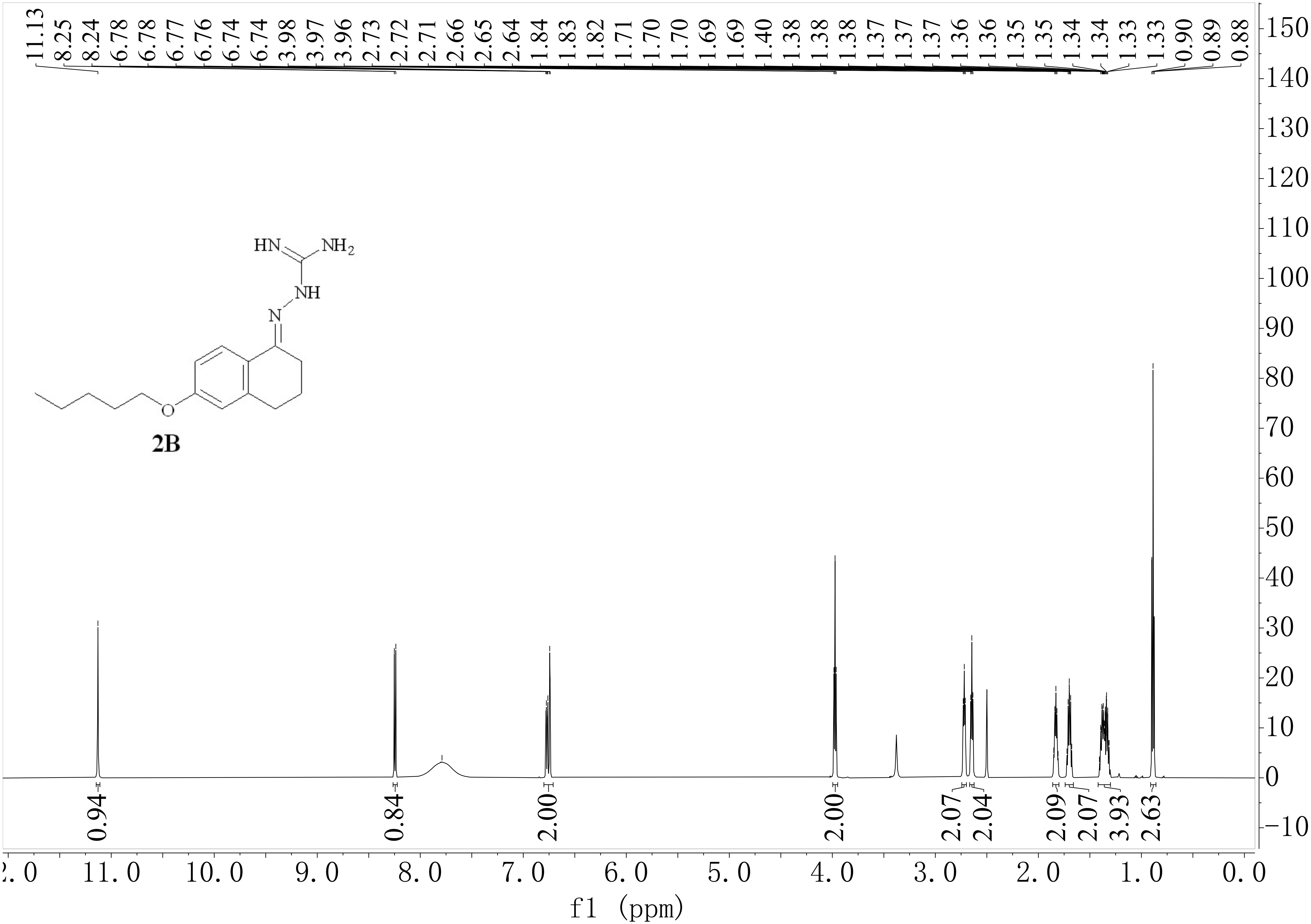
11.13 8.25 8.24 6.78 6.78 6.77 6.76 6.74 6.74 3.98 3.97 3.96 2.73 2.72 2.71 2.66 2.65 2.64 1.84 1.83 1.82 1.71 1.70 1.70 1.69 1.69 1.40 1.38 1.38 1.38 1.37 1.37 1.37 1.36 1.36 1.35 1.35 1.34 1.34 1.33 1.33 0.90 0.89 0.88
HN
NH2
NH
N
O
2B
0.94
0.84
2.00
2.00
2.07
2.04
2.09
2.07
3.93
2.63
f1 (ppm)

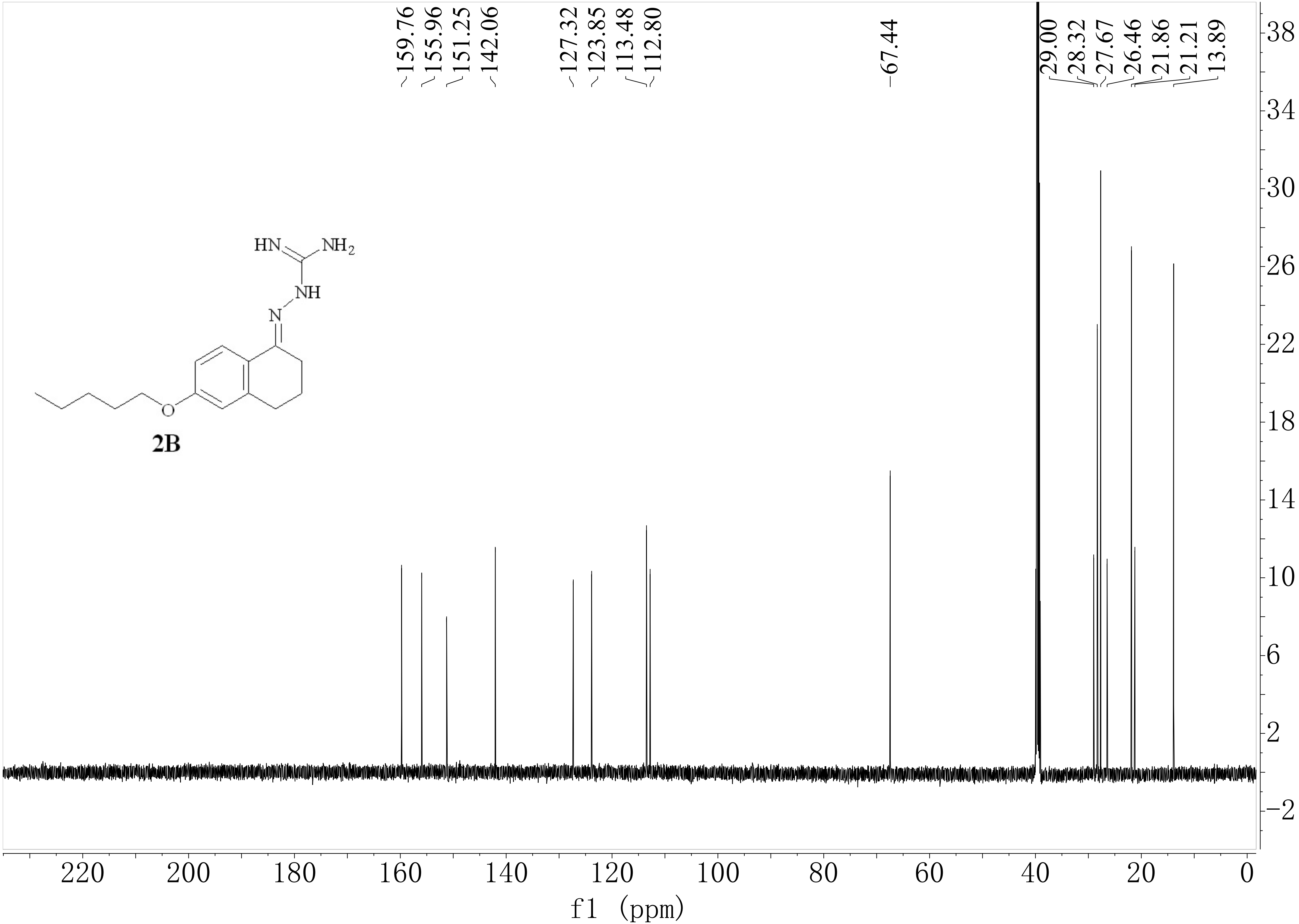

159.76
155.96
151.25
142.06
127.32
123.85
113.48
112.80
67.44
29.00
28.32
27.67
26.46
21.86
21.21
13.89
HN
NH2
NH
N
O
2B
f1 (ppm)

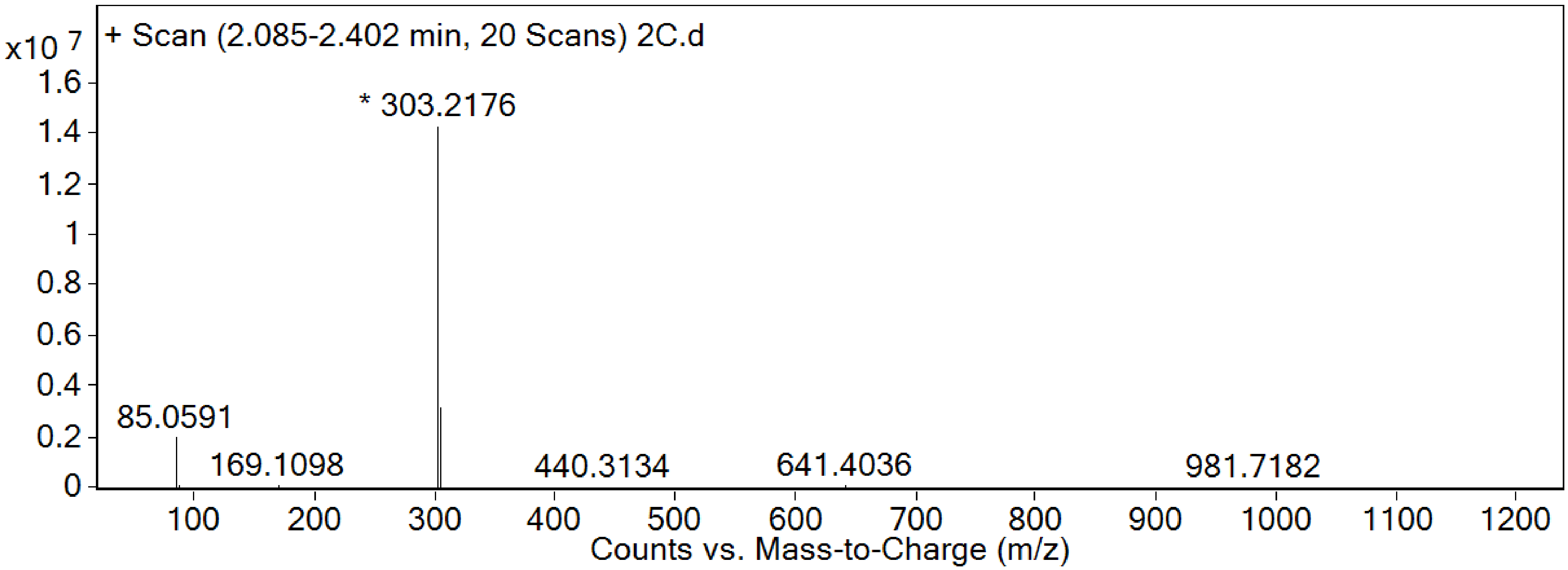

x10 7
+ Scan (2.085-2.402 min, 20 Scans) 2C.d
1.6
1.4
1.2
1
0.8
0.6
0.4
0.2
0
* 303.2176
85.0591
169.1098
440.3134
641.4036
981.7182
100
200
300
400
500
600
700
800
900
1000
1100
1200
Counts vs. Mass-to-Charge (m/z)

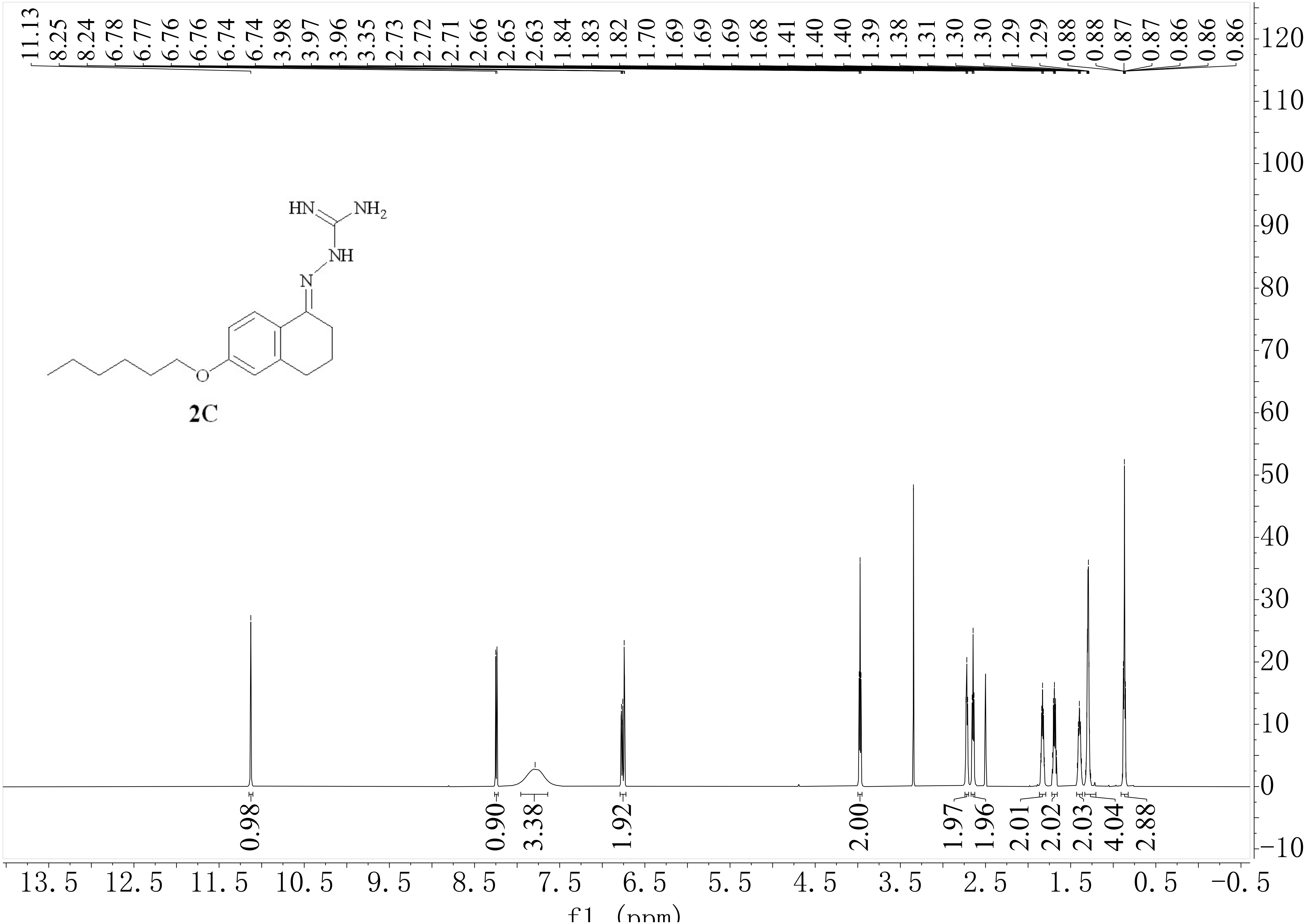
11.13
8.25
8.24
6.78
6.77
6.76
6.76
6.74
6.74
3.98
3.97
3.96
3.35
2.73
2.72
2.71
2.66
2.65
2.63
1.84
1.83
1.82
1.70
1.69
1.69
1.69
1.69
1.68
1.41
1.40
1.40
1.39
1.38
1.31
1.30
1.30
1.29
1.29
0.88
0.88
0.87
0.87
0.86
0.86
0.86
HN
NH2
NH
N
O
2C
0.98
0.90
3.38
1.92
2.00
1.97
1.96
2.01
2.02
2.03
4.04
2.88
13.5
12.5
11.5
10.5
9.5
8.5
7.5
6.5
5.5
4.5
3.5
2.5
1.5
0.5
-0.5
f1 (ppm)
120
110
100
90
80
70
60
50
40
30
20
10
0
-10

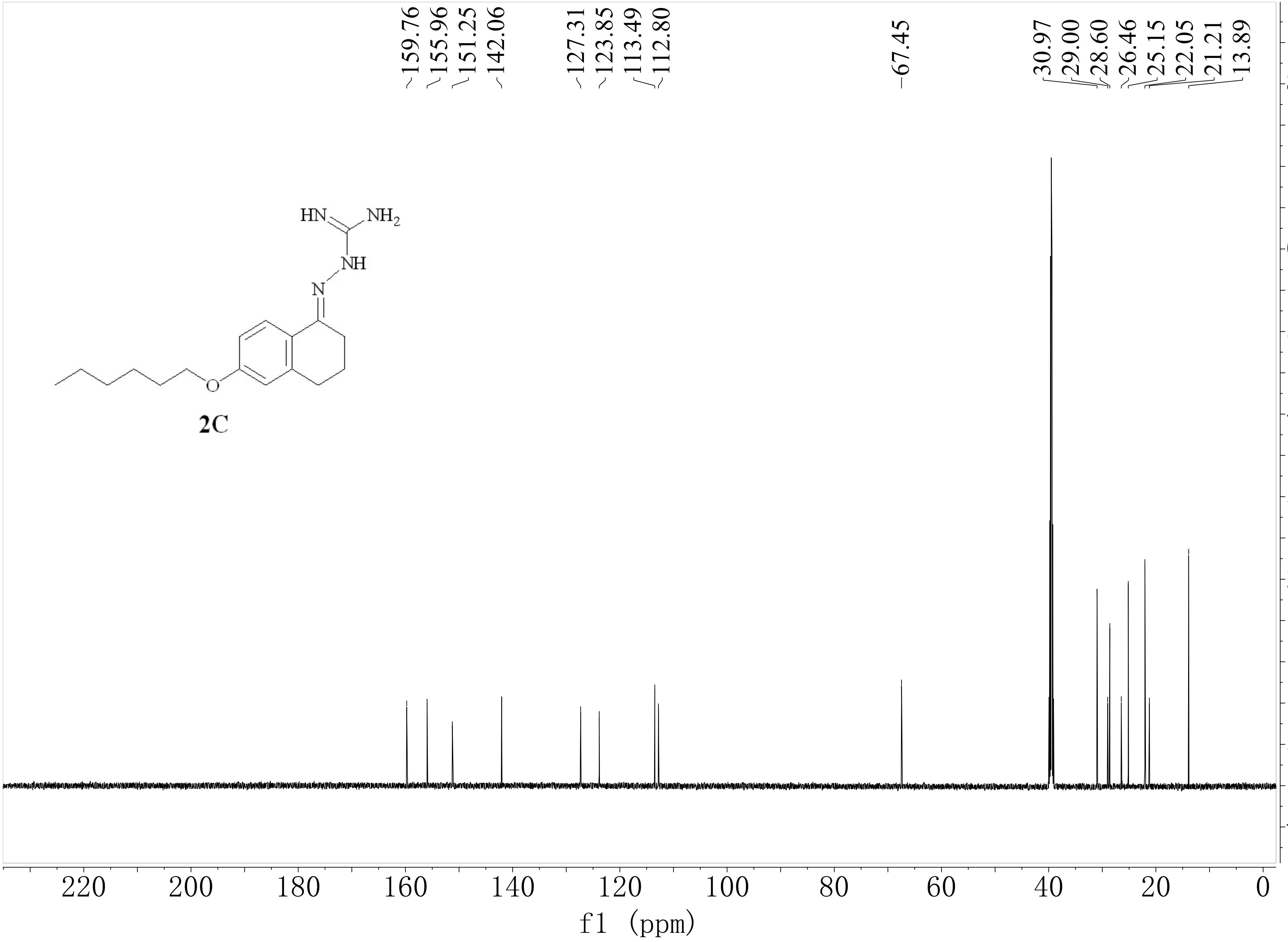
159.76
155.96
151.25
142.06
127.31
123.85
113.49
112.80
67.45
30.97
29.00
28.60
26.46
25.15
22.05
21.21
13.89
HN
NH2
NH
N
O
2C
85
75
65
55
45
35
25
15
5
−5
220
200
180
160
140
120
100
80
60
40
20
0
f1 (ppm)

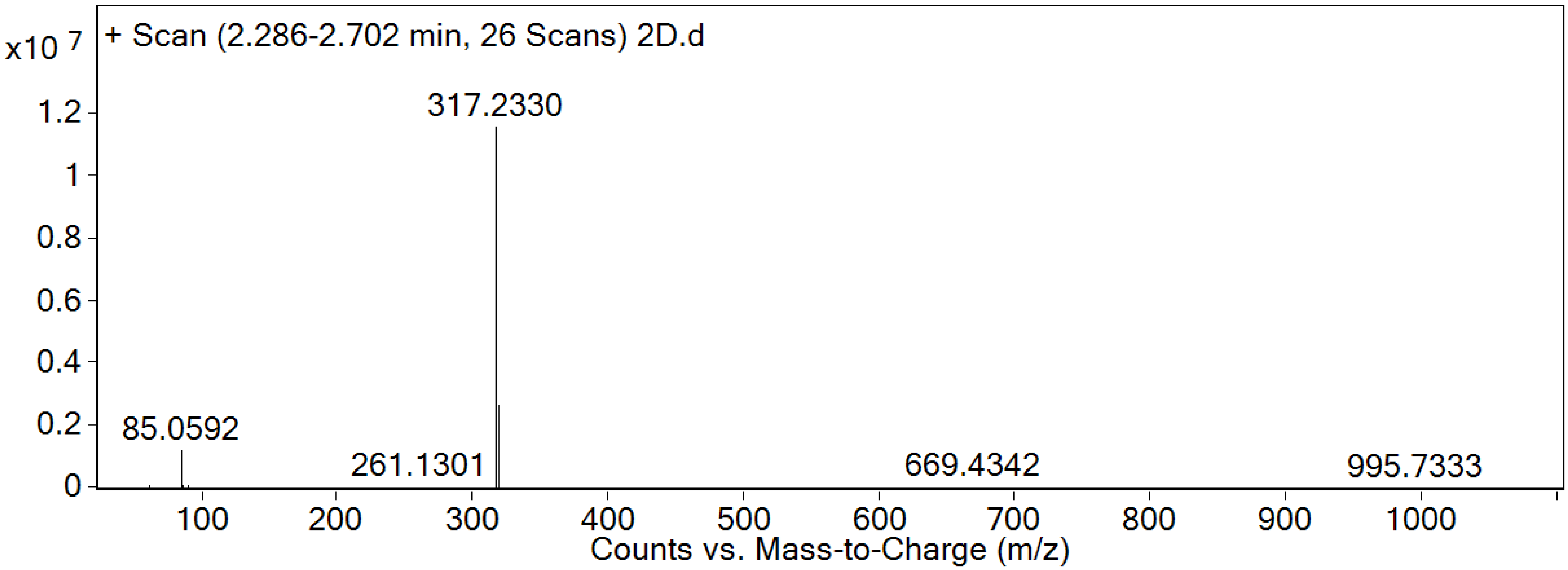

x10 7
+ Scan (2.286-2.702 min, 26 Scans) 2D.d
1.2
1
0.8
0.6
0.4
0.2
0
317.2330
85.0592
261.1301
669.4342
995.7333
100
200
300
400
500
600
700
800
900
1000
Counts vs. Mass-to-Charge (m/z)

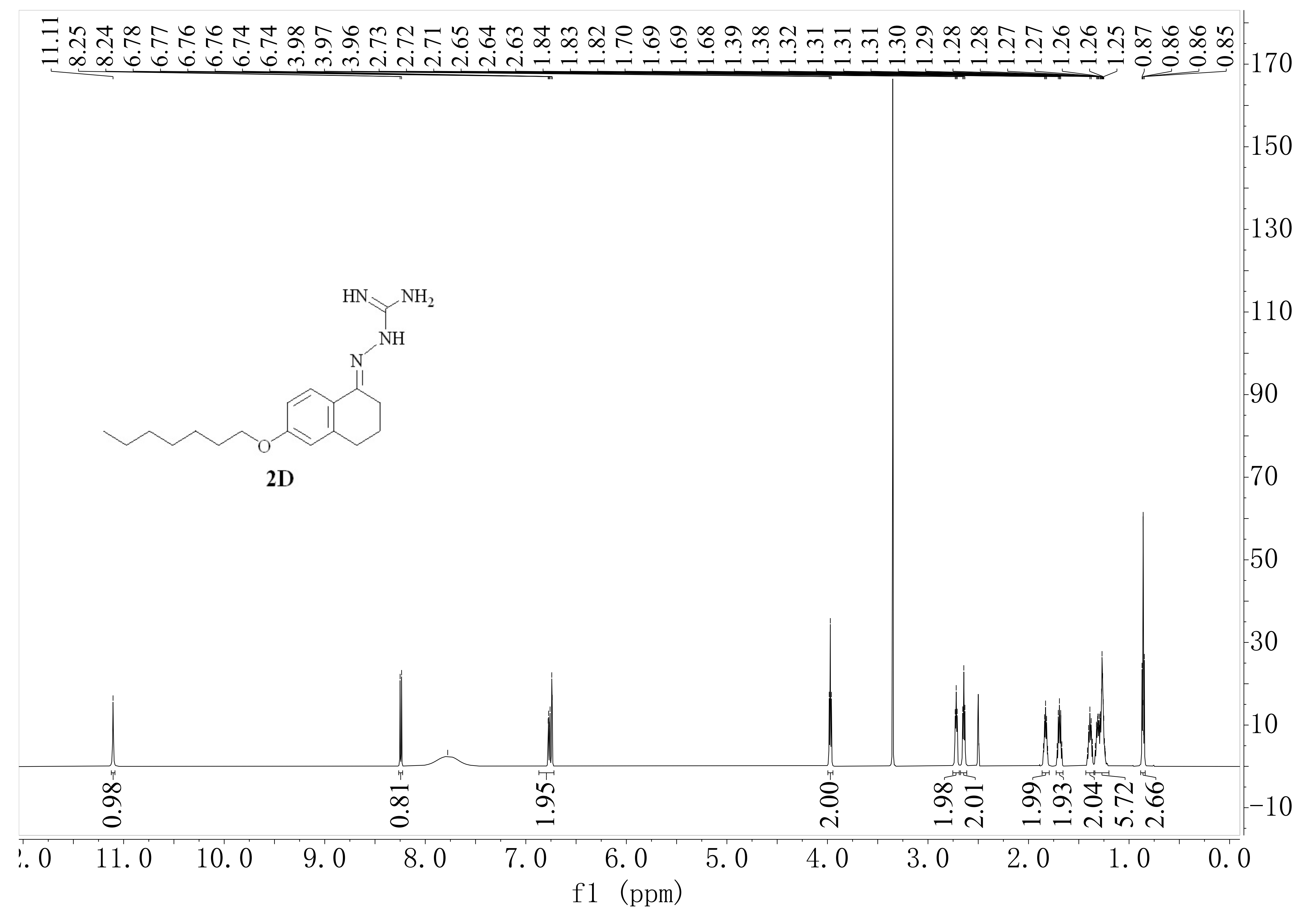

11.11
8.25
8.24
6.78
6.77
6.76
6.76
6.74
6.74
3.98
3.97
3.96
2.73
2.72
2.71
2.65
2.64
2.63
1.84
1.83
1.82
1.70
1.69
1.69
1.68
1.39
1.38
1.32
1.31
1.31
1.31
1.30
1.29
1.28
1.28
1.27
1.27
1.26
1.26
1.25
0.87
0.86
0.86
0.85
HN
NH2
NH
N
O
2D
0.98
0.81
1.95
2.00
1.98
2.01
1.99
1.93
2.04
5.72
2.66
f1 (ppm)

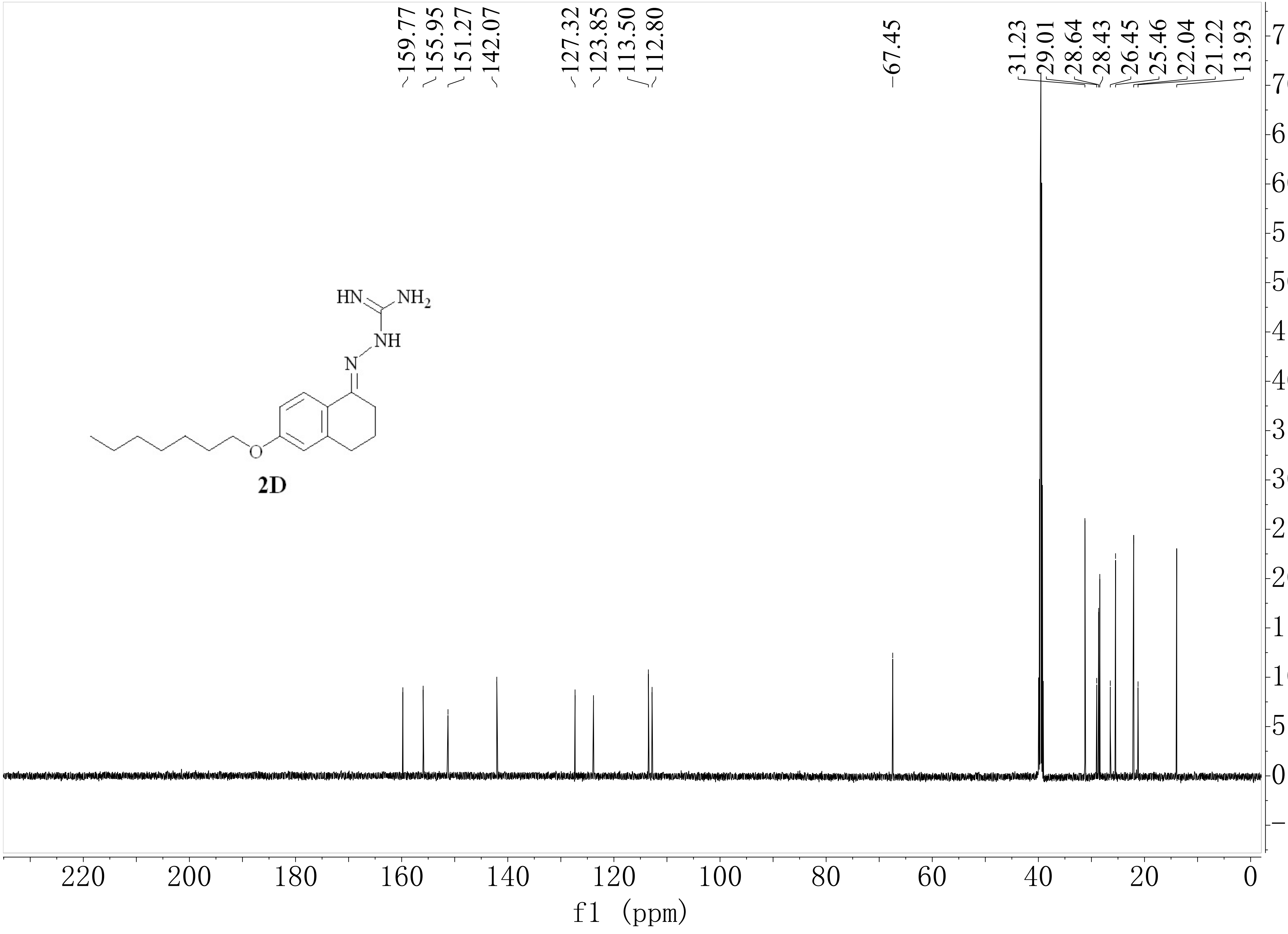
159.77
155.95
151.27
142.07
127.32
123.85
113.50
112.80
67.45
31.23
29.01
28.64
28.43
26.45
25.46
22.04
21.22
13.93
HN
NH2
NH
N
O
2D
75
70
65
60
55
50
45
40
35
30
25
20
15
10
5
0
−5
220
200
180
160
140
120
100
80
60
40
20
0
f1 (ppm)

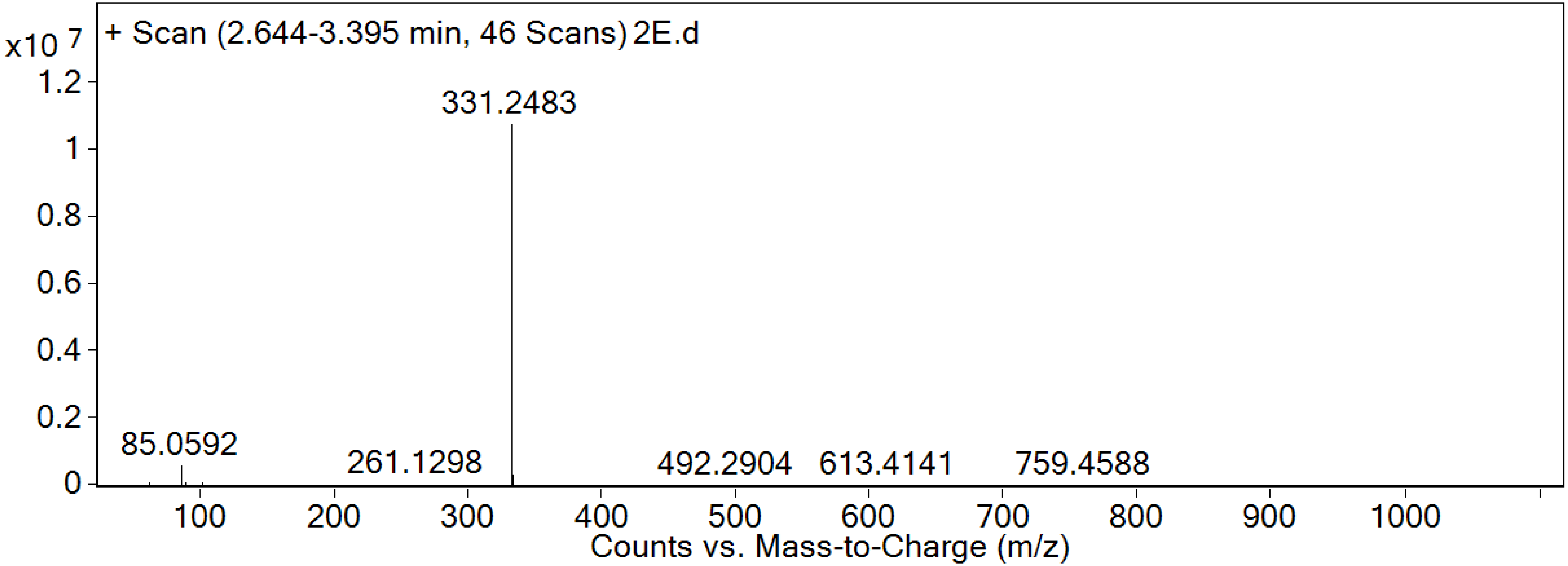

x10 7
+ Scan (2.644-3.395 min, 46 Scans) 2E.d
1.2
1
0.8
0.6
0.4
0.2
0
331.2483
85.0592
261.1298
492.2904
613.4141
759.4588
100
200
300
400
500
600
700
800
900
1000
Counts vs. Mass-to-Charge (m/z)

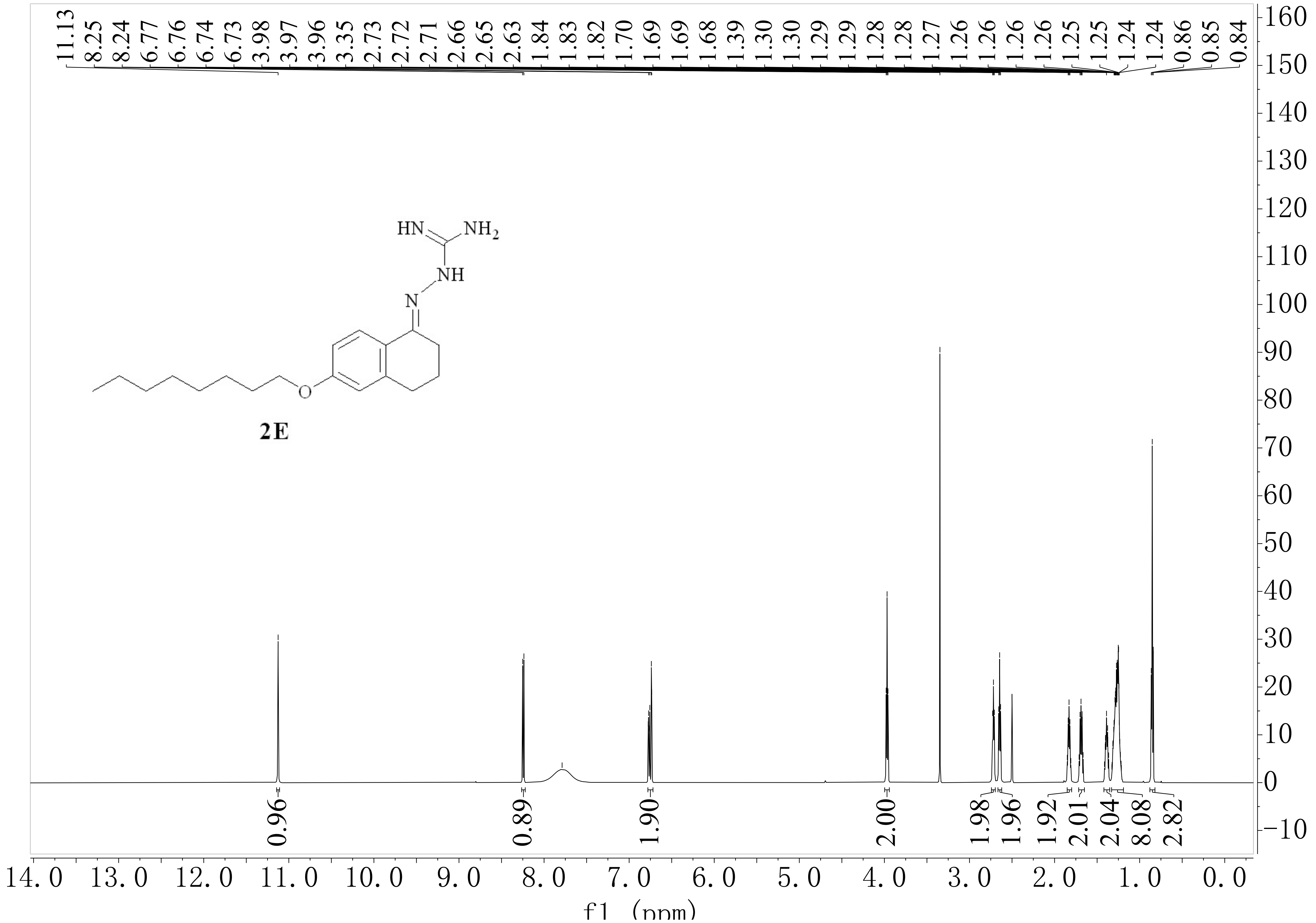
11.13 8.25 8.24 6.77 6.76 6.74 6.73 3.98 3.97 3.96 3.35 2.73 2.72 2.71 2.66 2.65 2.63 1.84 1.83 1.82 1.70 1.69 1.69 1.68 1.39 1.30 1.30 1.29 1.29 1.28 1.28 1.27 1.26 1.26 1.26 1.26 1.25 1.25 1.24 1.24 0.86 0.85 0.84
HN
NH2
NH
N
O
2E
0.96 0.89 1.90 2.00 1.98 1.96 1.92 2.01 2.04 8.08 2.82
14.0 13.0 12.0 11.0 10.0 9.0 8.0 7.0 6.0 5.0 4.0 3.0 2.0 1.0 0.0
f1 (ppm)
160 150 140 130 120 110 100 90 80 70 60 50 40 30 20 10 0 −10

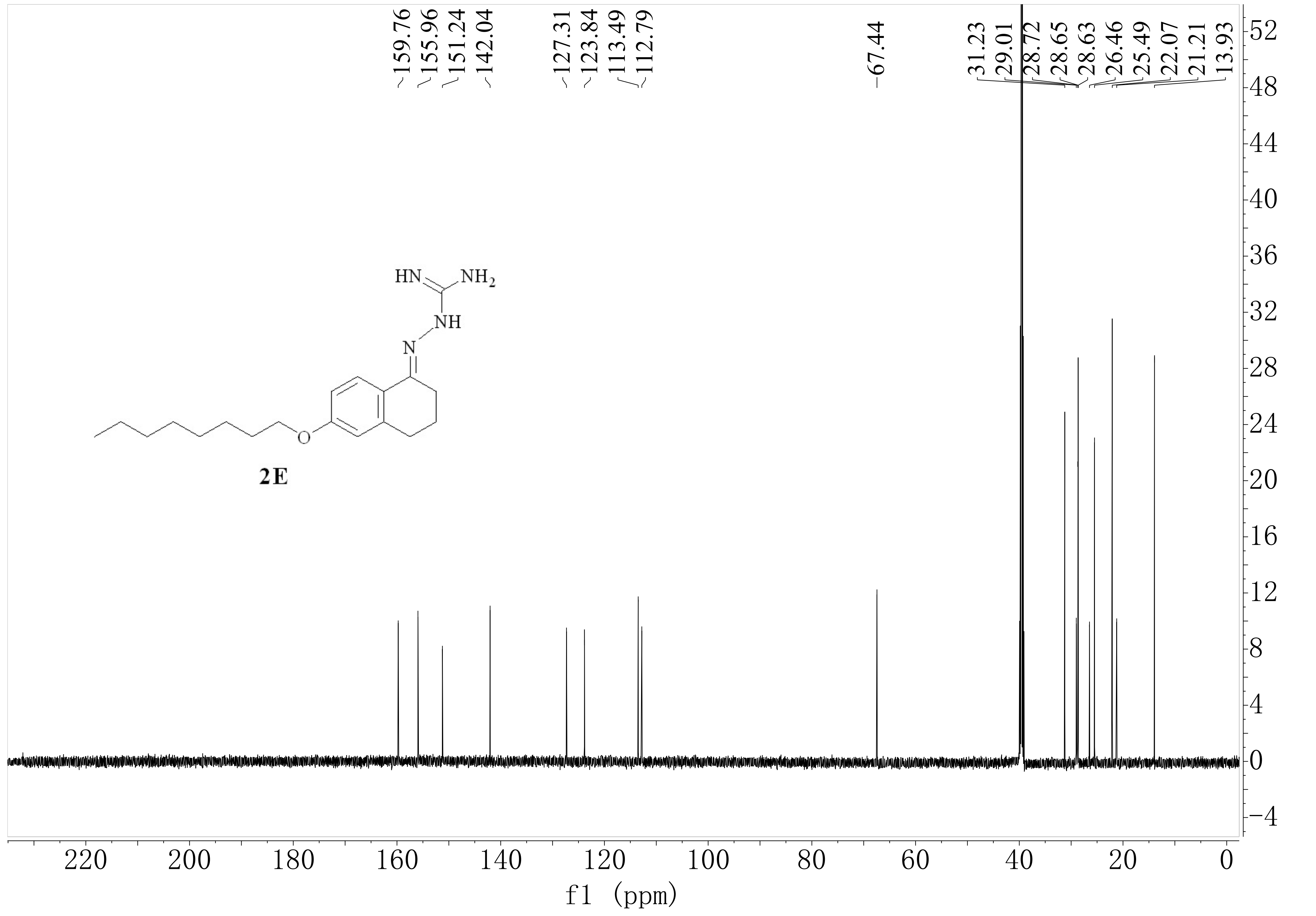

159.76
155.96
151.24
142.04
127.31
123.84
113.49
112.79
67.44
31.23
29.01
28.72
28.65
28.63
26.46
25.49
22.07
21.21
13.93
HN
NH2
NH
N
O
2E
f1 (ppm)

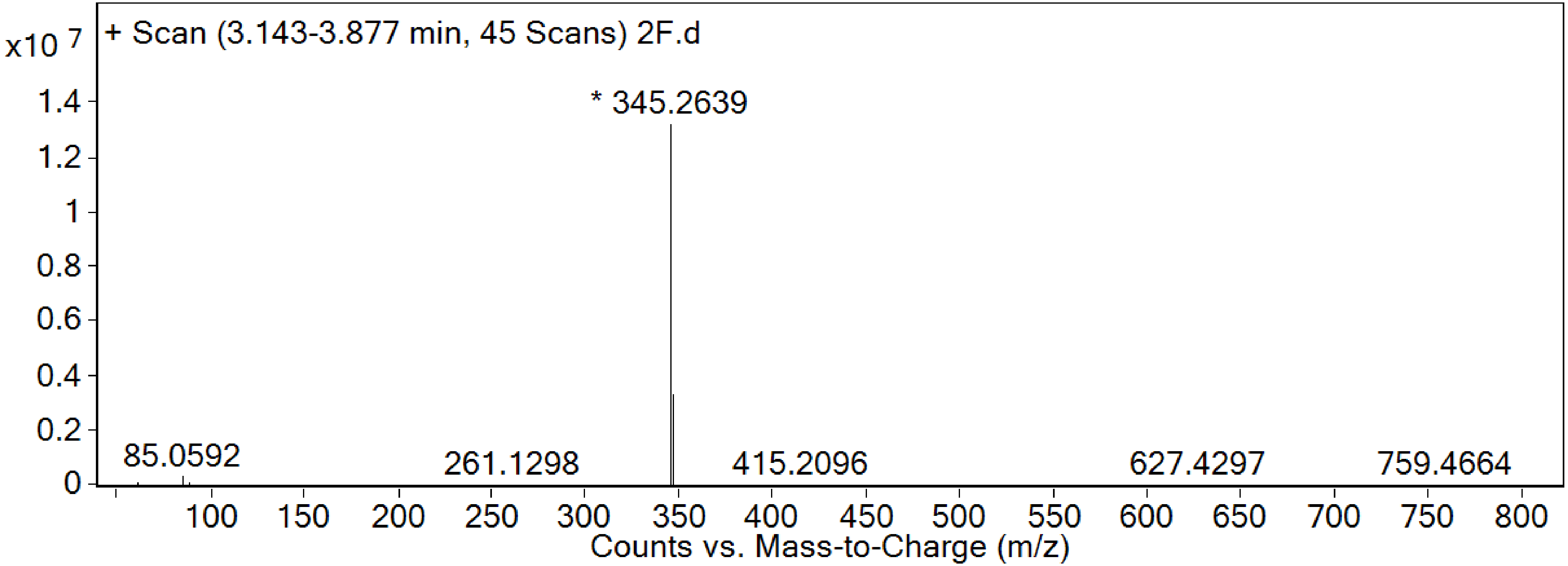

x10 7
+ Scan (3.143-3.877 min, 45 Scans) 2F.d
* 345.2639
85.0592
261.1298
415.2096
627.4297
759.4664
1.4
1.2
1
0.8
0.6
0.4
0.2
0
100 150 200 250 300 350 400 450 500 550 600 650 700 750 800
Counts vs. Mass-to-Charge (m/z)

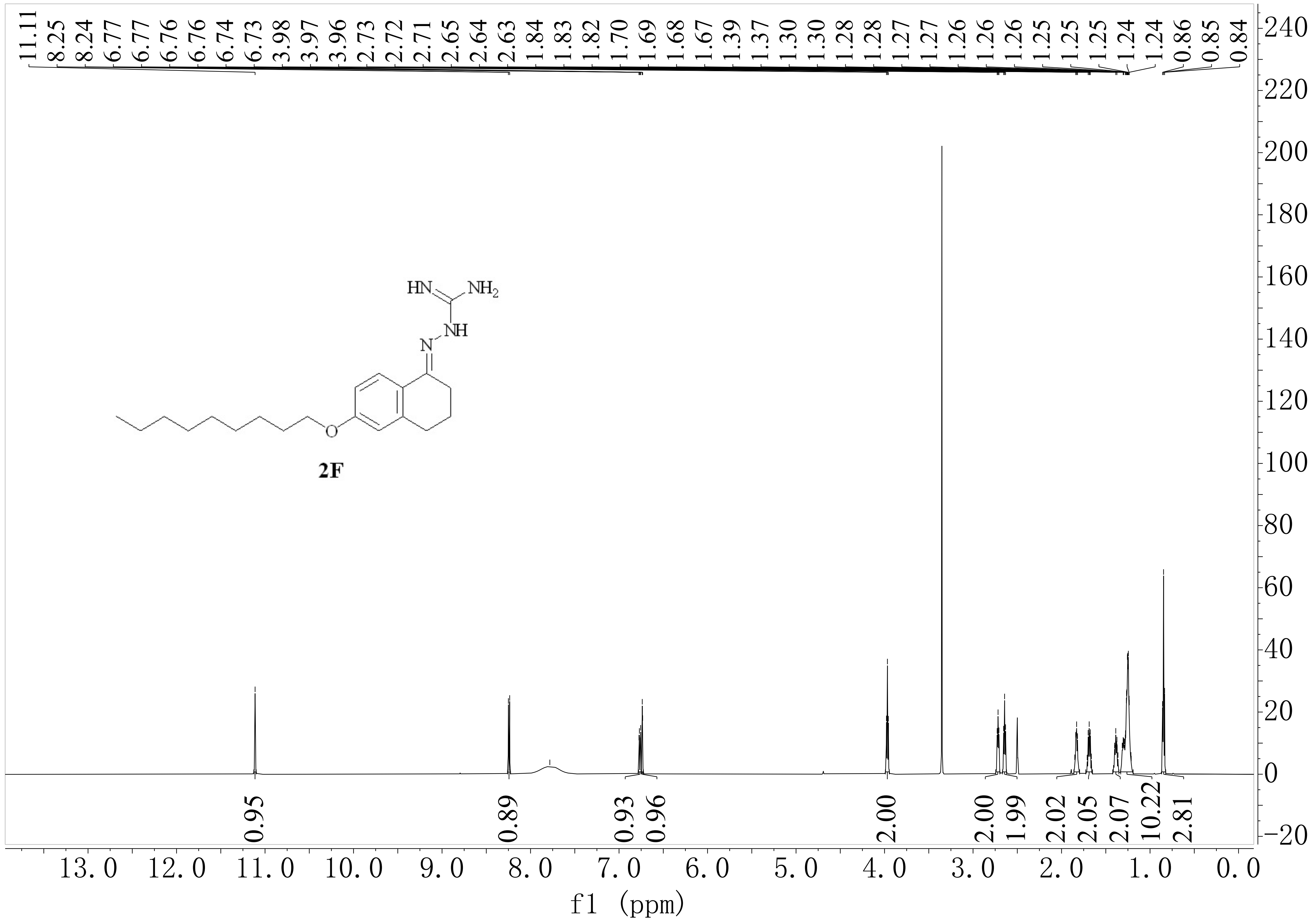
11.11 8.25 8.24 6.77 6.77 6.76 6.76 6.74 6.73 3.98 3.97 3.96 2.73 2.72 2.71 2.65 2.64 2.63 1.84 1.83 1.82 1.70 1.69 1.68 1.67 1.39 1.37 1.30 1.30 1.28 1.28 1.27 1.27 1.26 1.26 1.26 1.25 1.25 1.25 1.24 1.24 0.86 0.85 0.84
HN
NH2
NH
N
O
2F
0.95 0.89 0.93 0.96 2.00 2.00 1.99 2.02 2.05 2.07 10.22 2.81
13.0 12.0 11.0 10.0 9.0 8.0 7.0 6.0 5.0 4.0 3.0 2.0 1.0 0.0
f1 (ppm)
240 220 200 180 160 140 120 100 80 60 40 20 0 -20

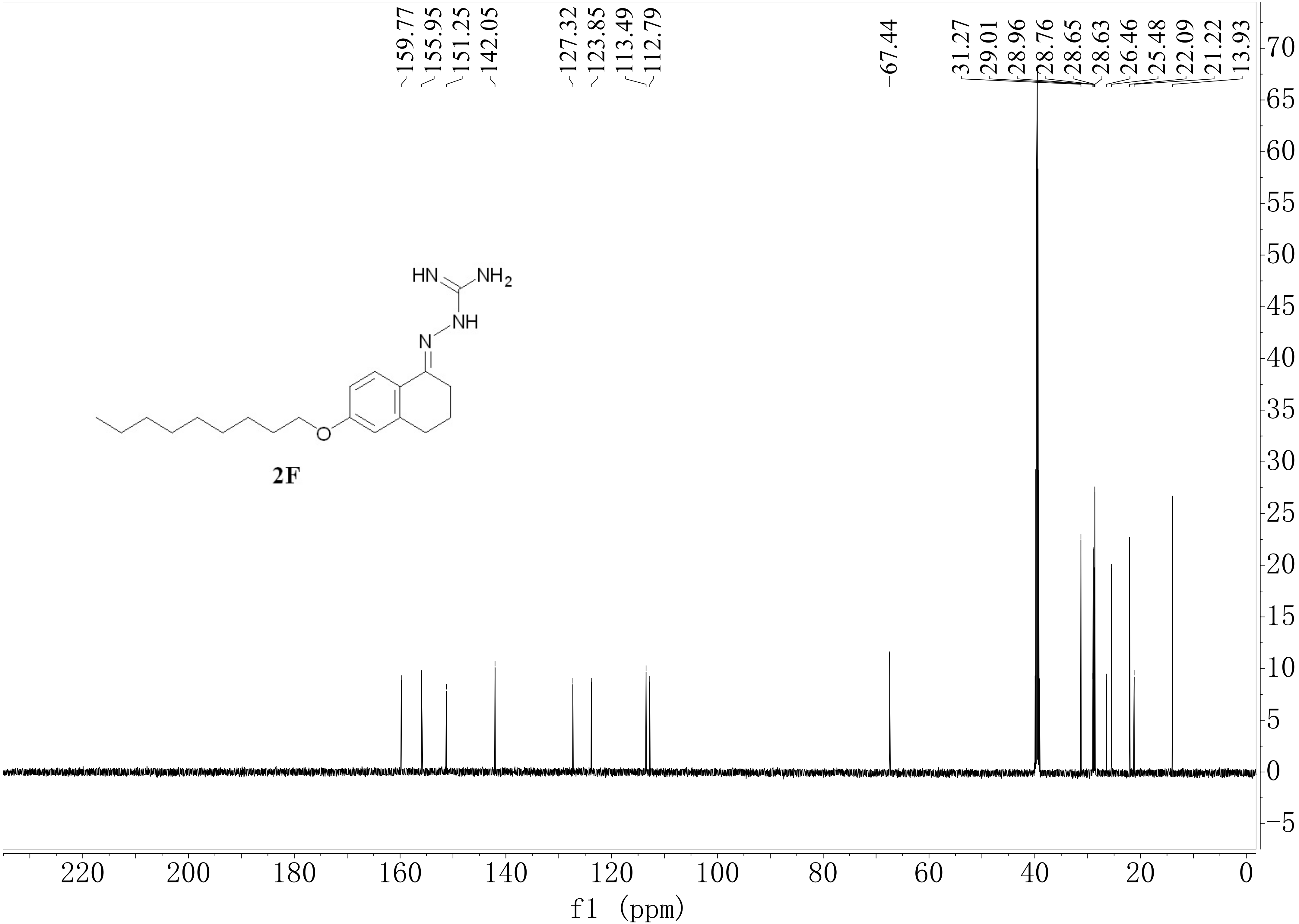

159.77
155.95
151.25
142.05
127.32
123.85
113.49
112.79
67.44
31.27
29.01
28.96
28.76
28.65
28.63
26.46
25.48
22.09
21.22
13.93
HN
NH2
NH
N
O
2F
70
65
60
55
50
45
40
35
30
25
20
15
10
5
0
−5
220
200
180
160
140
120
100
80
60
40
20
0
f1 (ppm)

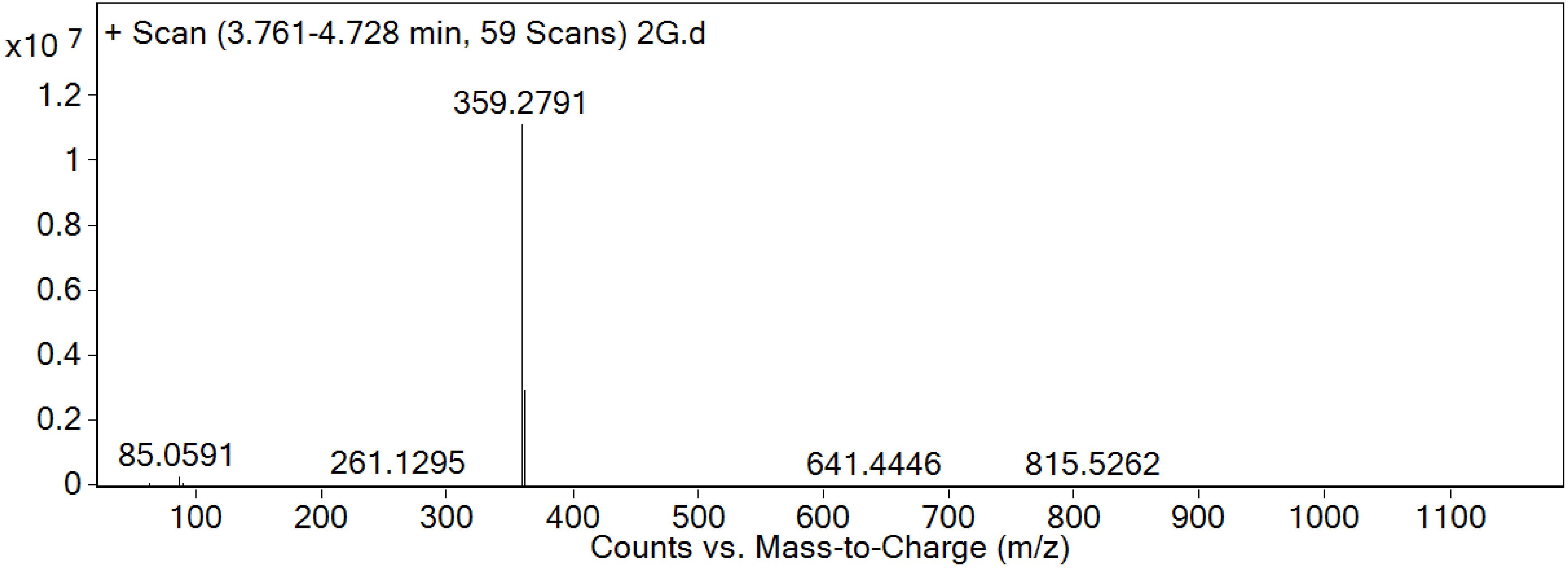

x10 7
+ Scan (3.761-4.728 min, 59 Scans) 2G.d
359.2791
85.0591
261.1295
641.4446
815.5262
1.2
1
0.8
0.6
0.4
0.2
0
100
200
300
400
500
600
700
800
900
1000
1100
Counts vs. Mass-to-Charge (m/z)

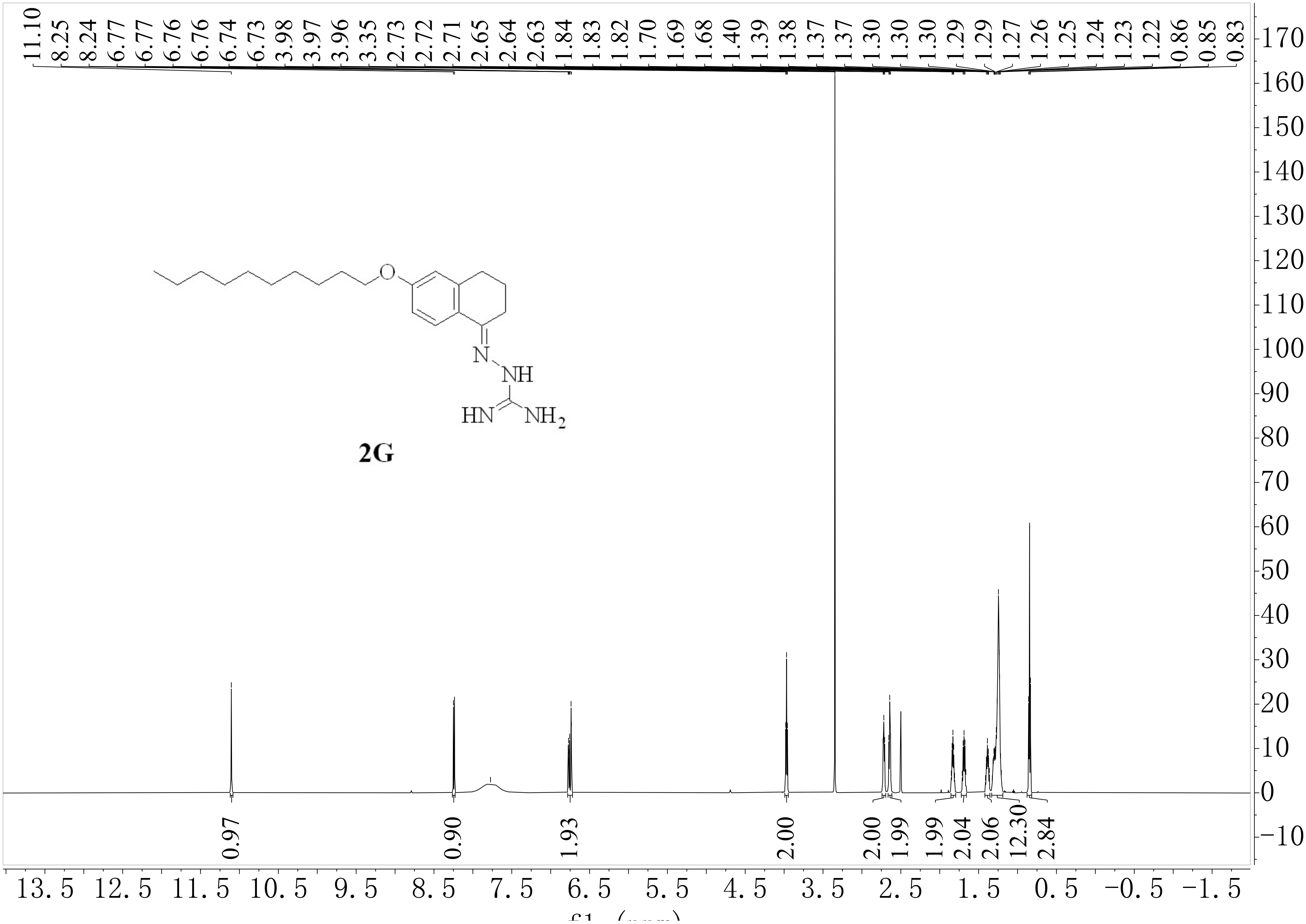
11.10 8.25 8.24 6.77 6.77 6.76 6.76 6.74 6.73 3.98 3.97 3.96 3.35 2.73 2.72 2.71 2.65 2.64 2.63 1.84 1.83 1.82 1.70 1.69 1.68 1.40 1.39 1.38 1.37 1.37 1.30 1.30 1.30 1.29 1.29 1.27 1.26 1.25 1.24 1.23 1.22 0.86 0.85 0.83
O
N
NH
HN
NH2
2G
0.97
0.90
1.93
2.00
2.00
1.99
1.99
2.04
2.06
12.30
2.84
13.5 12.5 11.5 10.5 9.5 8.5 7.5 6.5 5.5 4.5 3.5 2.5 1.5 0.5 −0.5 −1.5
170 160 150 140 130 120 110 100 90 80 70 60 50 40 30 20 10 0 −10

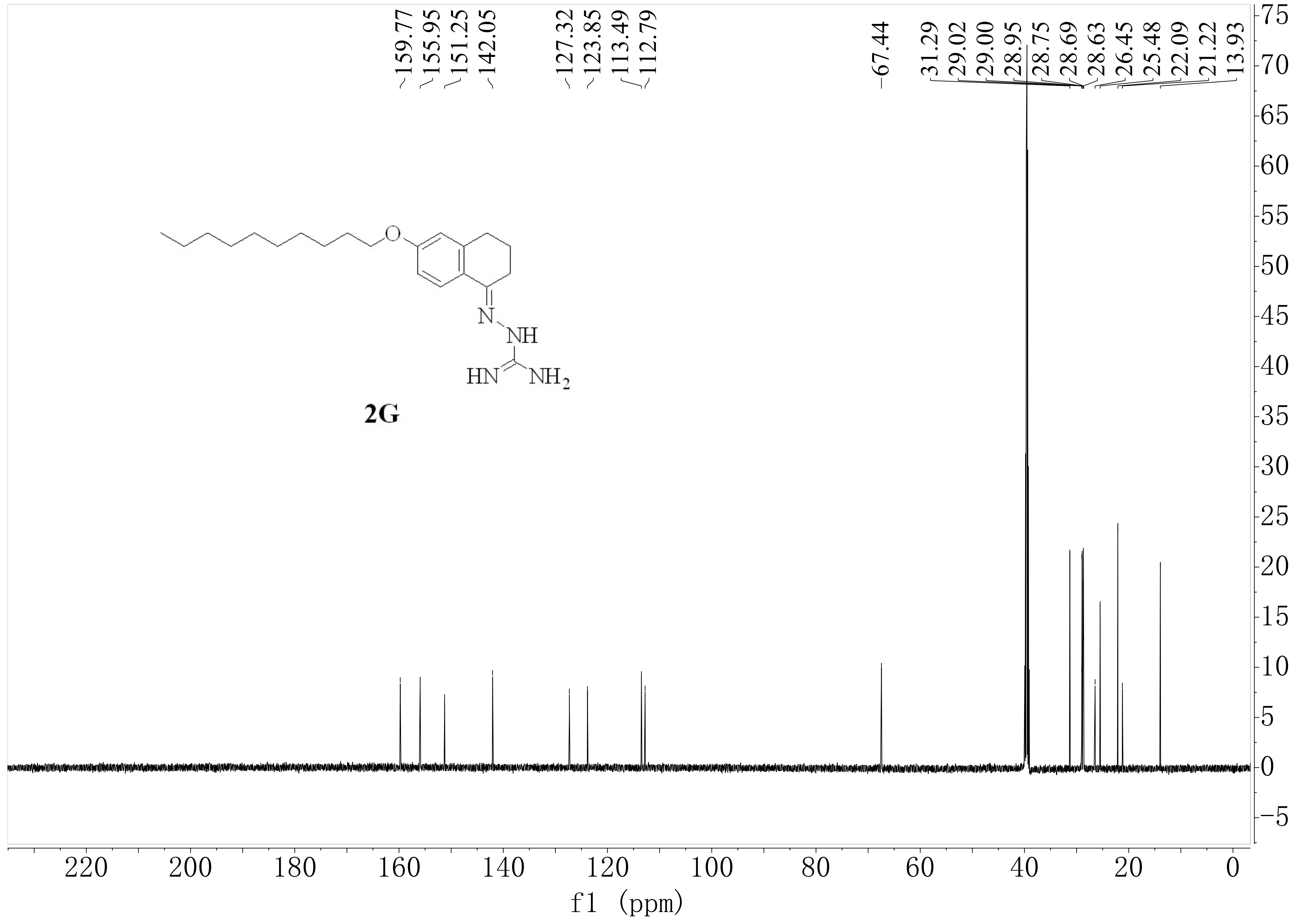
159.77
155.95
151.25
142.05
127.32
123.85
113.49
112.79
67.44
31.29
29.02
29.00
28.95
28.75
28.69
28.63
26.45
25.48
22.09
21.22
13.93
O
N
NH
HN
NH2
2G
f1 (ppm)

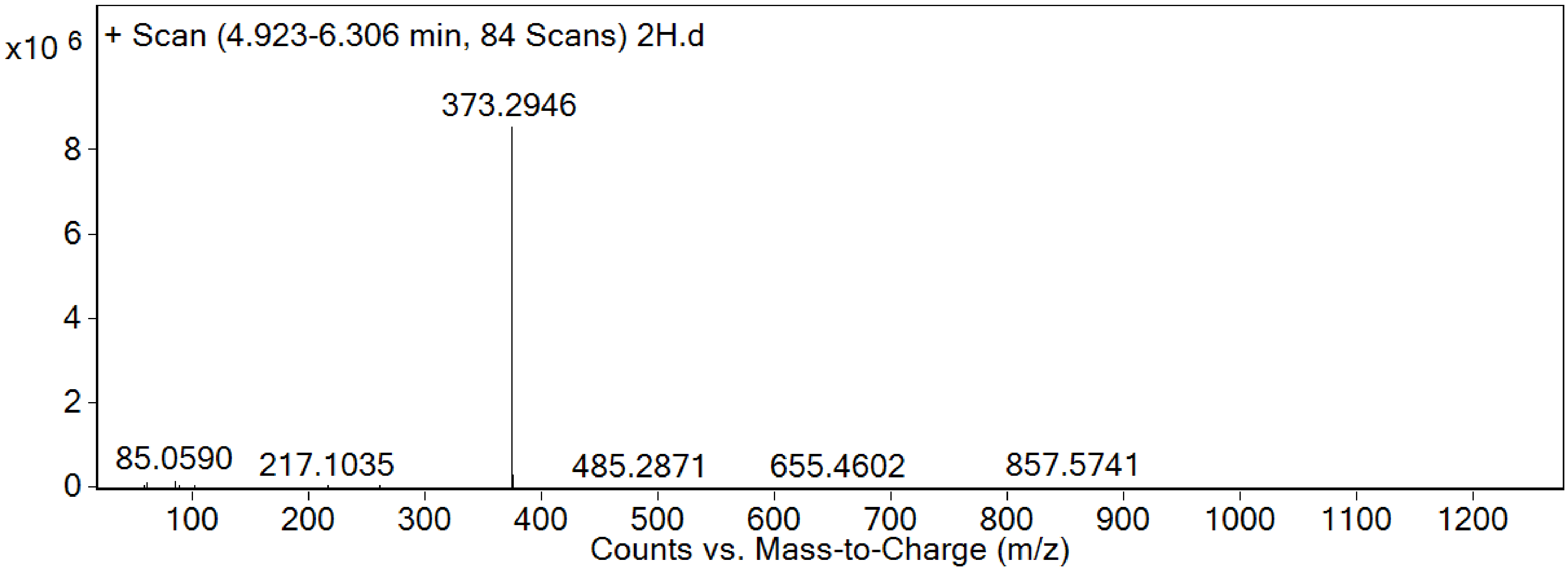
x10 6
+ Scan (4.923-6.306 min, 84 Scans) 2H.d
373.2946
85.0590
217.1035
485.2871
655.4602
857.5741
0
2
4
6
8
100
200
300
400
500
600
700
800
900
1000
1100
1200
Counts vs. Mass-to-Charge (m/z)

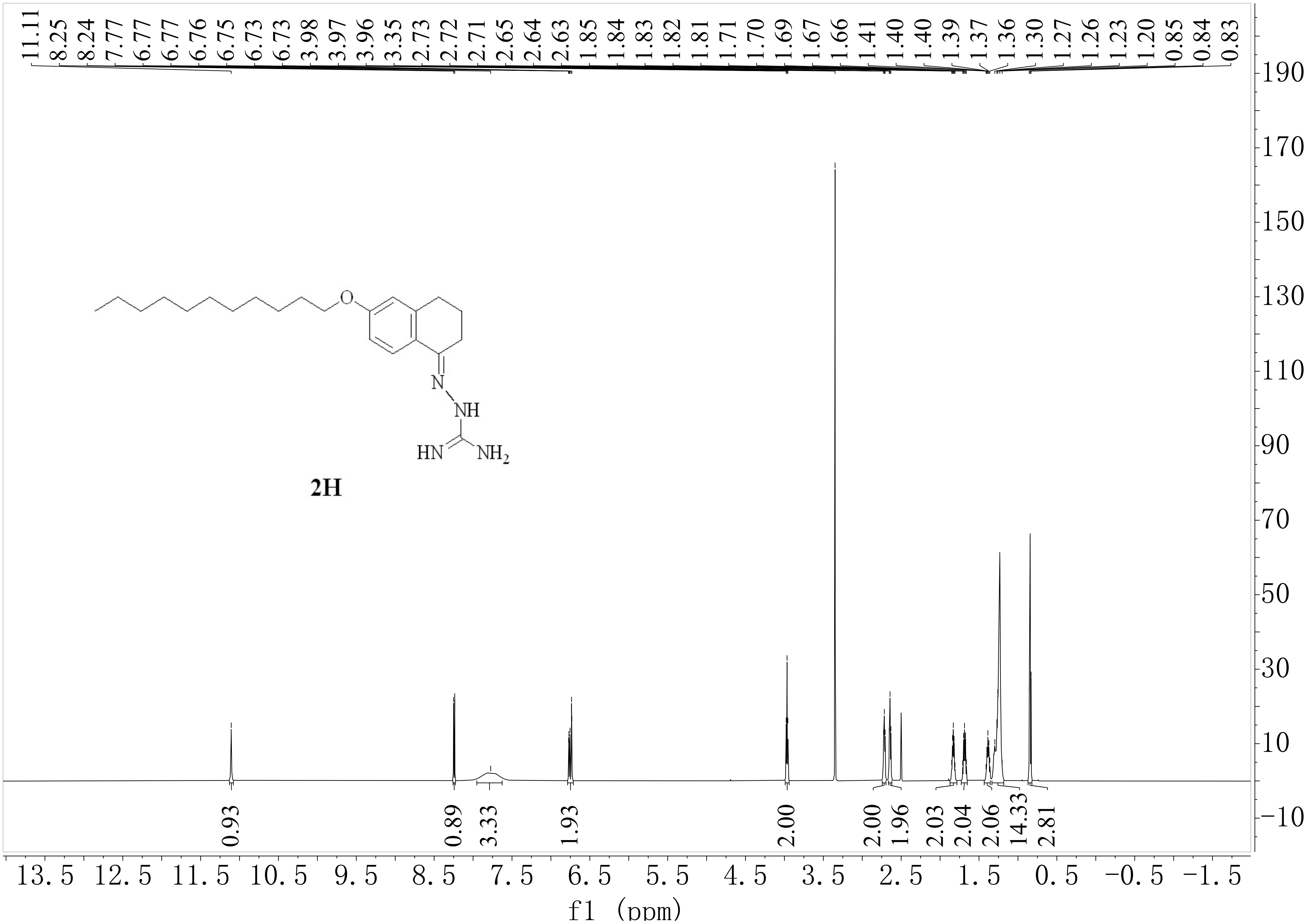
11.11 8.25 8.24 7.77 6.77 6.77 6.76 6.75 6.73 6.73 3.98 3.97 3.96 3.35 2.73 2.72 2.71 2.65 2.64 2.63 1.85 1.84 1.83 1.82 1.81 1.71 1.70 1.69 1.67 1.66 1.41 1.40 1.40 1.39 1.37 1.36 1.30 1.27 1.26 1.23 1.20 0.85 0.84 0.83
O
N
NH
HN
NH2
2H
0.93 0.89 3.33 1.93 2.00 2.00 1.96 2.03 2.04 2.06 14.33 2.81
13.5 12.5 11.5 10.5 9.5 8.5 7.5 6.5 5.5 4.5 3.5 2.5 1.5 0.5 -0.5 -1.5
f1 (ppm)
190 170 150 130 110 90 70 50 30 10 -10

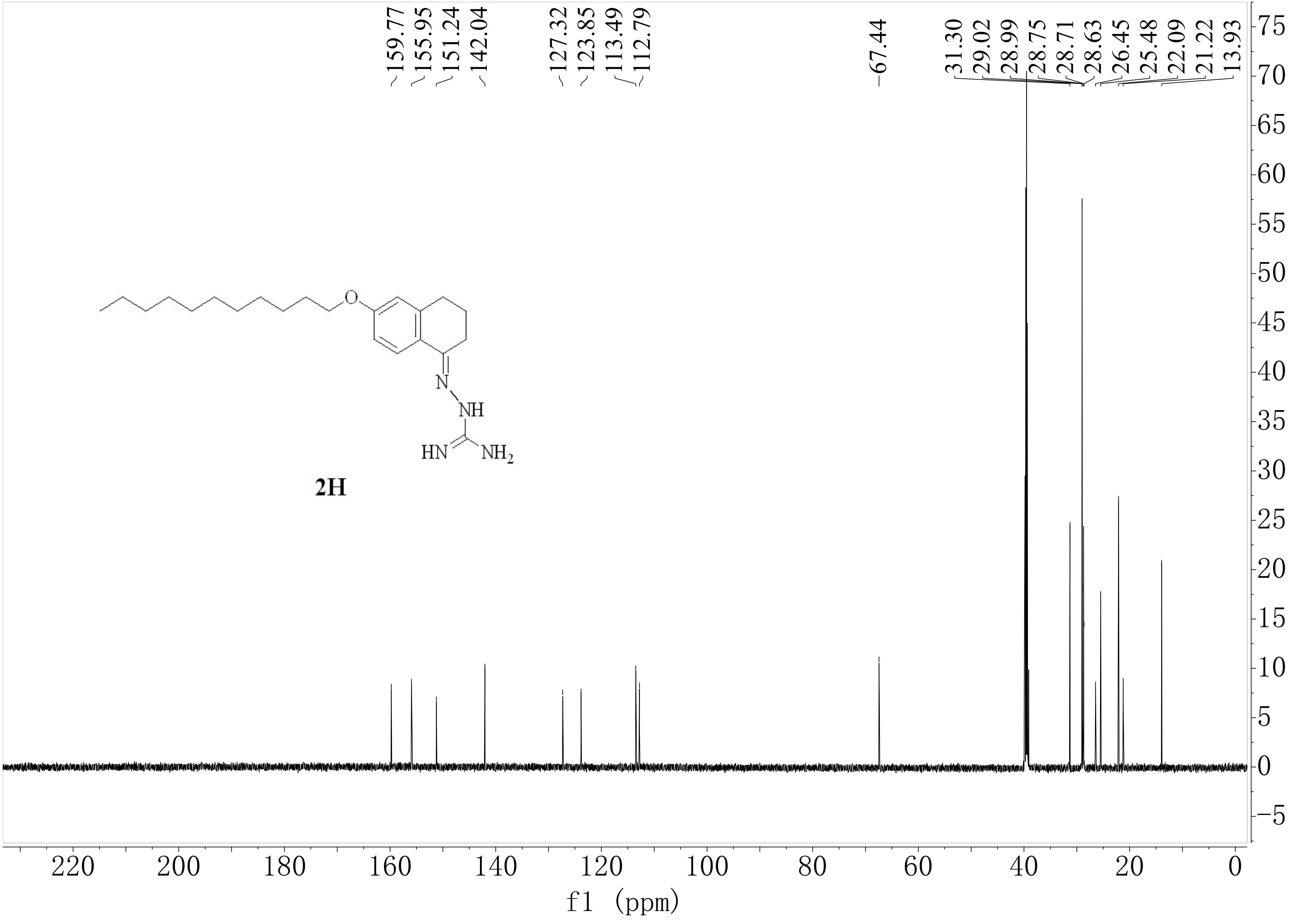
159.77
155.95
151.24
142.04
127.32
123.85
113.49
112.79
67.44
31.30
29.02
28.99
28.75
28.71
28.63
26.45
25.48
22.09
21.22
13.93
O
N
NH
HN
NH2
2H
220
200
180
160
140
120
100
80
60
40
20
0
f1 (ppm)
75
70
65
60
55
50
45
40
35
30
25
20
15
10
5
0
−5

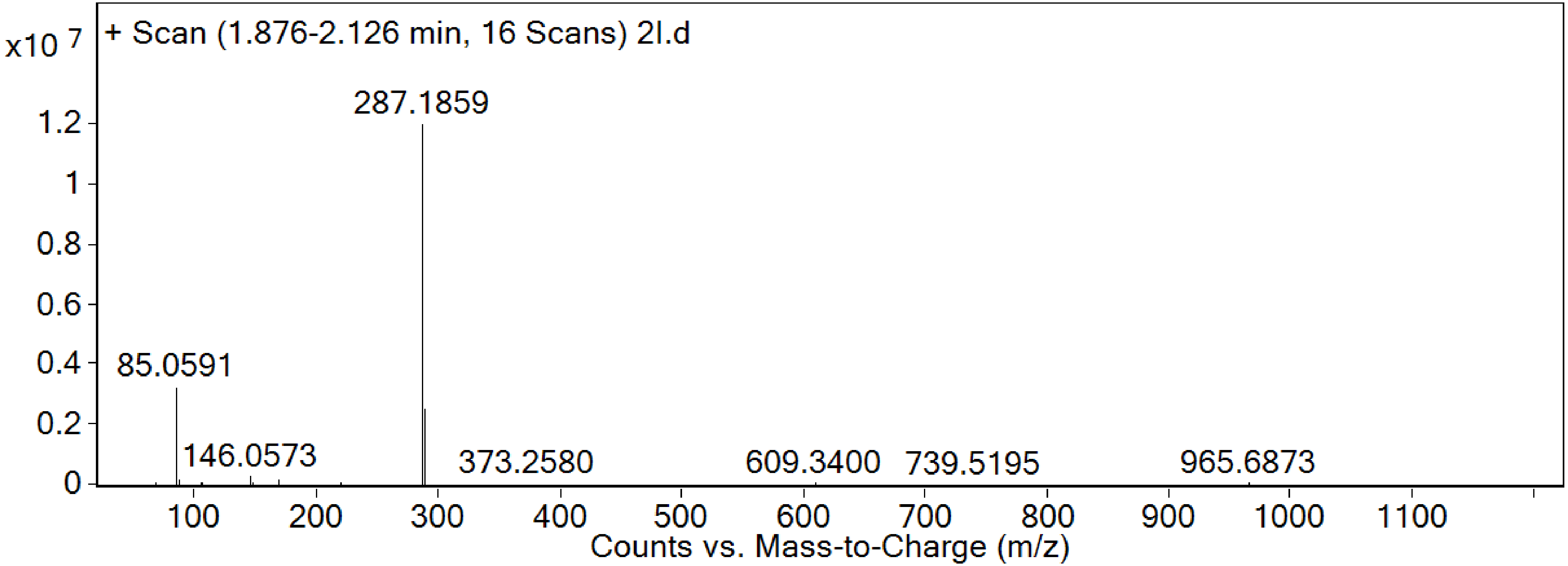

x10 7
+ Scan (1.876-2.126 min, 16 Scans) 2l.d
287.1859
85.0591
146.0573
373.2580
609.3400
739.5195
965.6873
1.2
1
0.8
0.6
0.4
0.2
0
100
200
300
400
500
600
700
800
900
1000
1100
Counts vs. Mass-to-Charge (m/z)

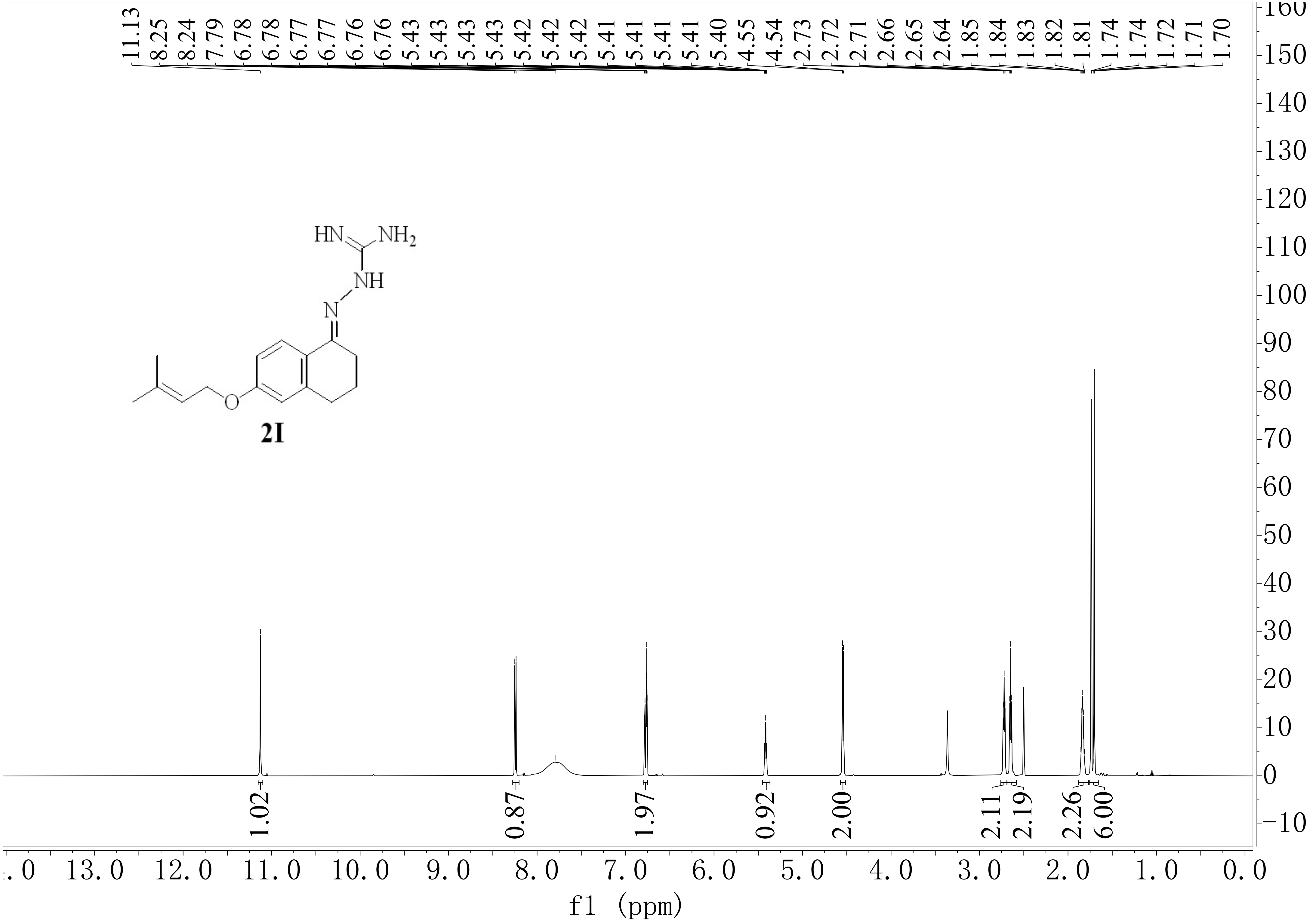
11.13
8.25
8.24
7.79
6.78
6.78
6.77
6.77
6.76
6.76
5.43
5.43
5.43
5.43
5.42
5.42
5.42
5.41
5.41
5.41
5.41
5.40
4.55
4.54
2.73
2.72
2.71
2.66
2.65
2.64
1.85
1.84
1.83
1.82
1.81
1.74
1.74
1.72
1.71
1.70
HN
NH2
NH
N
O
2I
1.02
0.87
1.97
0.92
2.00
2.11
2.19
2.26
6.00
13.0
12.0
11.0
10.0
9.0
8.0
7.0
6.0
5.0
4.0
3.0
2.0
1.0
0.0
f1 (ppm)
160
150
140
130
120
110
100
90
80
70
60
50
40
30
20
10
0
−10

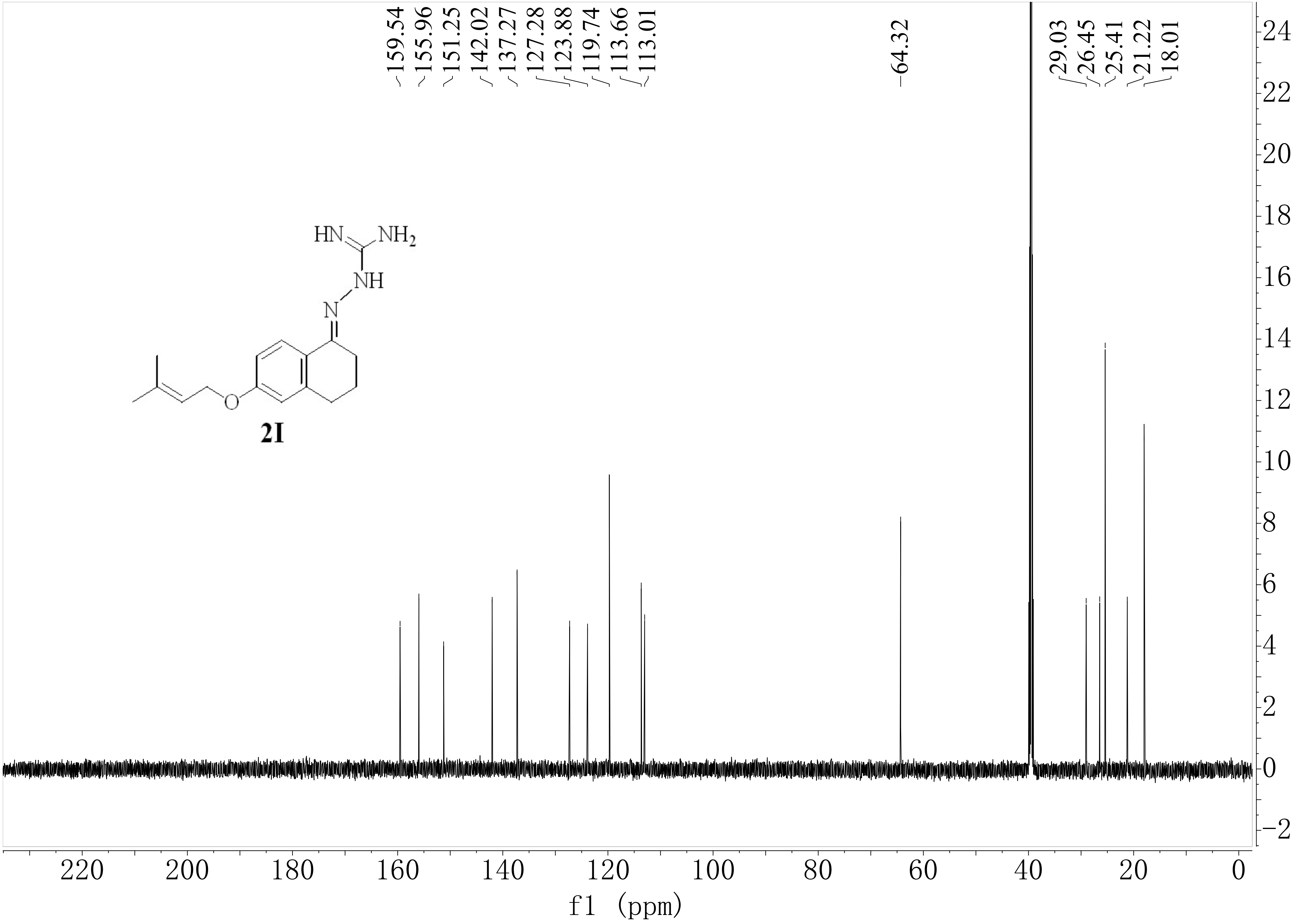

159.54
155.96
151.25
142.02
137.27
127.28
123.88
119.74
113.66
113.01
64.32
29.03
26.45
25.41
21.22
18.01
HN
NH2
NH
N
O
2I
24
22
20
18
16
14
12
10
8
6
4
2
0
−2
220
200
180
160
140
120
100
80
60
40
20
0
f1 (ppm)

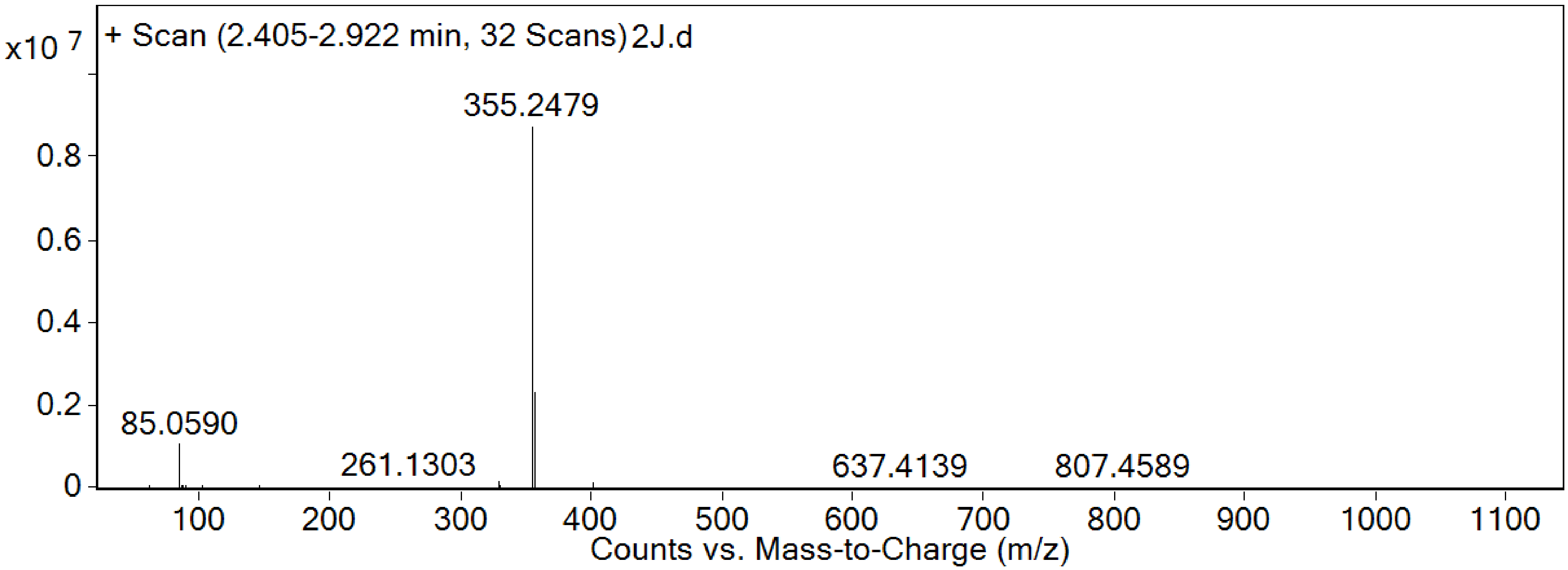
x10 7
+ Scan (2.405-2.922 min, 32 Scans) 2J.d
355.2479
85.0590
261.1303
637.4139
807.4589
0.8
0.6
0.4
0.2
0
100
200
300
400
500
600
700
800
900
1000
1100
Counts vs. Mass-to-Charge (m/z)

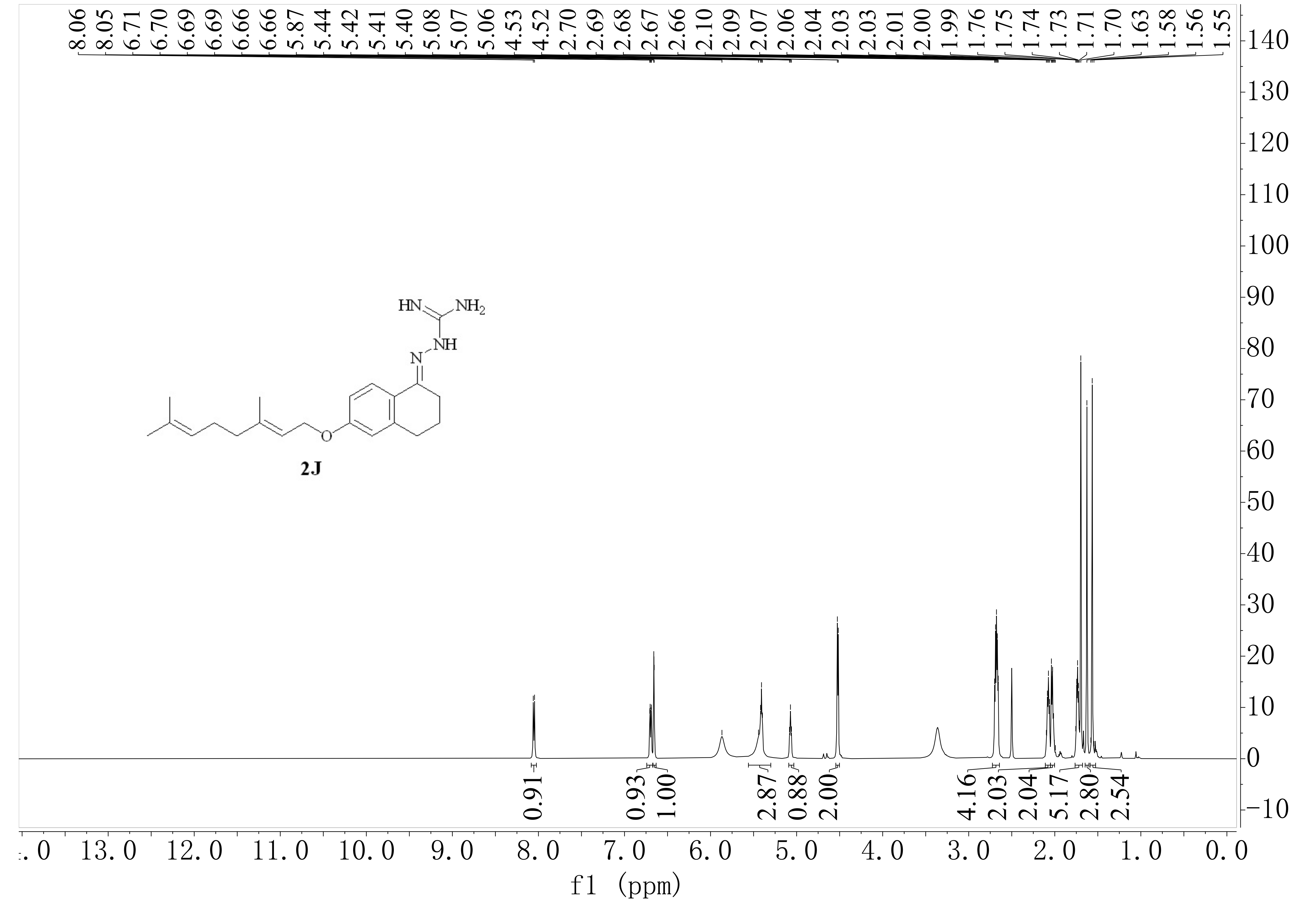

8.06 8.05 6.71 6.70 6.69 6.69 6.66 6.66 5.87 5.44 5.42 5.41 5.40 5.08 5.07 5.06 4.53 4.52 2.70 2.69 2.68 2.67 2.66 2.10 2.09 2.07 2.06 2.04 2.03 2.03 2.01 2.00 1.99 1.76 1.75 1.74 1.73 1.71 1.70 1.63 1.58 1.56 1.55
HN
NH2
N
NH
O
2J
0.91
0.93
1.00
2.87
0.88
2.00
4.16
2.03
2.04
5.17
2.80
2.54
13.0 12.0 11.0 10.0 9.0 8.0 7.0 6.0 5.0 4.0 3.0 2.0 1.0 0.0
f1 (ppm)
140 130 120 110 100 90 80 70 60 50 40 30 20 10 0 −10

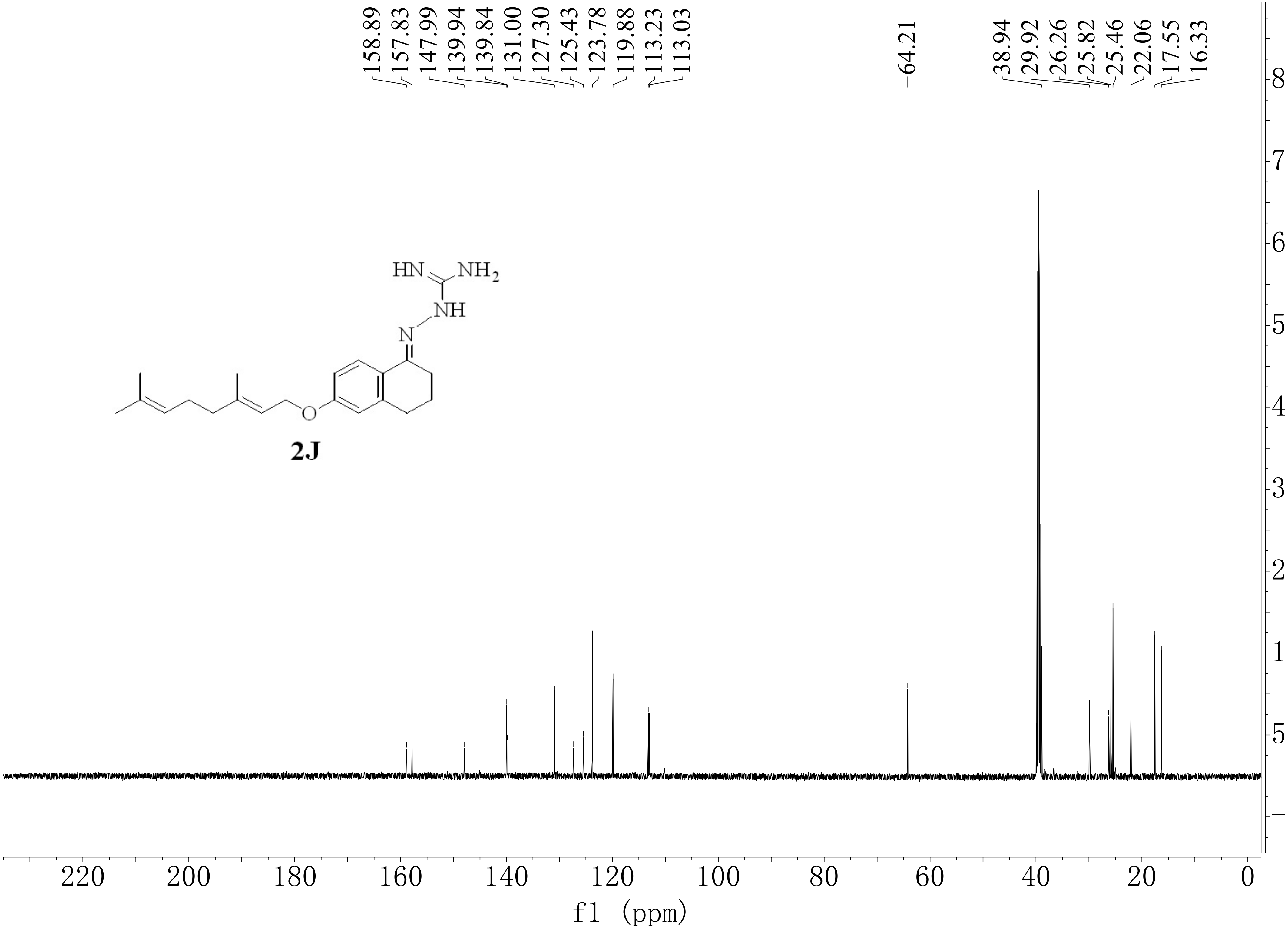

158.89
157.83
147.99
139.94
139.84
131.00
127.30
125.43
123.78
119.88
113.23
113.03
64.21
38.94
29.92
26.26
25.82
25.46
22.06
17.55
16.33
HN
NH2
NH
N
O
2J
f1 (ppm)
